# A Retrotransposon Insertion in *GhMML3_D12* Is Likely Responsible for the Lintless Locus *li_3_* of Tetraploid Cotton

**DOI:** 10.3389/fpls.2020.593679

**Published:** 2020-11-26

**Authors:** Wei Chen, Yan Li, Shouhong Zhu, Shengtao Fang, Lanjie Zhao, Yan Guo, Junyi Wang, Li Yuan, Youjun Lu, Fang Liu, Jinbo Yao, Yongshan Zhang

**Affiliations:** ^1^State Key Laboratory of Cotton Biology, Institute of Cotton Research, Chinese Academy of Agricultural Sciences, Anyang, China; ^2^School of Biological Science and Food Engineering, Anyang Institute of Technology, Anyang, China

**Keywords:** fiber initiation, fiberless mutants, fuzzless mutants, MYBMIXTA-like transcription factors, cotton (*Gossypium* spp.)

## Abstract

Cotton (*Gossypium*) seed fibers can be divided into lint (long) or fuzz (very short). Using fiberless (fuzzless-lintless) mutants, the lint initiation gene *Li_3_* was identified by map-based cloning. The gene is an R2R3-MYB transcription factor located on chromosome D12 (*GhMML3_D12*). Sequence analysis revealed that *li_3_* is a loss-of-function allele containing a retrotransposon insertion in the second exon that completely blocks the gene’s expression. The genetic loci *n_2_* and *n_3_* underlying the recessive fuzzless phenotype in *Gossypium hirsutum* were also mapped. The genomic location of *n_3_* overlapped with that of the dominant fuzzless locus *N_1_*, and *n_3_* appeared to be a loss-of-function allele caused by a single nucleotide polymorphism (SNP) mutation in the coding region of *GhMML3_A12*. The *n_2_* allele was found to be co-located with *li_3_* and originated from *G. babardense*. *n_2_* and *li_3_* are possibly the multiple alleles of the *GhMML3_D12* gene. Genetic analysis showed that *Li_3_* and *N_3_* are a pair of homologs with additive effects for the initiation of fibers (fuzz or lint). In addition, the presence of another locus was speculated, and it appeared to show an inhibitory effect on the expression of *GhMML3*. These findings provide new information about the genetic factors affecting the initiation of fibers in cotton.

## Introduction

The cotton genus (*Gossypium*) includes approximately 50 species, and the diploid (*n* = 13) species can be divided into eight diploid genome groups (A–G and K; Wendel and Cronn, 2003). Allotetraploid cotton resulted from the merger of two formerly isolated diploid genomes (A- and D-genome ancestors; Wendel and Cronn, 2003). Nearly all cotton species have epidermal seed trichomes (fibers), though in some D-genome species, the fibers are relatively sparse. Seed fibers are usually divided into lint and fuzz according to mature fiber length. Lint fibers are spinnable (generally <25 mm) and are initiated −1 to 0 days post-anthesis (DPA), while fuzz fibers are much shorter (<5 mm) and are initiated 3–5 DPA (Lang, 1938; Stewart, 1975).

Upland cotton (*Gossypium hirsutum*, AD_1_) is the most widely cultivated allotetraploid species. Many “naked seed” (fuzzless-linted) mutants have been identified in *G. hirsutum* (Gh) cultivars, and these can be divided into dominant and recessive types based on genetic analyses (Kearney and Harrison, 1927; Carver, 1929; Ware, 1929; Ware et al., 1947). A dominant allele *N_1_* and a recessive allele *n_2_* have been proposed to be responsible for the fuzzless phenotype in some mutant lines (Endrizzi et al., 1985). Recently, two new recessive loci (*n_3_* and *n_4_*^t^) were identified, and an *N_3_* allele was thought to have epistatic effects on the expression of the *n_2_* locus (Turley and Kloth, 2002; Bechere et al., 2012). In addition, the *N_1_* and *n_2_* alleles were found to significantly reduce lint percentage (Turley and Kloth, 2002, 2008). All *Gossypium barbadense* (Gb; another cultivated allotetraploid species, AD_2_) lines display the recessive fuzzless phenotype, although the amount of fuzz is affected by environmental factors (Kearney and Harrison, 1928).

Various fiberless (fuzzless-lintless) mutant lines have also been reported in Gh. These include Line L40 (Musaev and Abzalov, 1972), Mcu5 (Nadarajan and Rangasamy, 1988), Xu142 *fl* (Zhang and Pan, 1991), and SL1-7-1 (Turley and Ferguson, 1996). However, the genotypes of these lines and the relationships among them remained to be clearly elucidated (Turley and Kloth, 2008). For example, three genotypes have been proposed for Xu142 *fl*: a three-locus type (*n_1_n_1_n_2_n_2_li_3_li_3_*) proposed by Zhang and Pan (1991), a four-locus type (*n_1_n_1_n_2_n_2_li_3_li_3_li_4_li_4_*) proposed by Du et al. (2001), and another four-locus type (*n_1_n_1_n_2_n_2_li_3_li_3_n_3_n_3_*) proposed by Turley and Kloth (2008). Xu142 *fl* was thought to differ from the original wild-type Xu142 (*n_1_n_1_N_2_N_2_li_3_li_3_*) at the *N_2_* locus (Zhang and Pan, 1991). Interestingly, a fiberless line, MD17 (*N_1_N_1_n_2_n_2_n_3_n_3_*), was developed from the cross between dominant (*N_1_N_1_N_2_N_2_n_3_n_3_*) and recessive (*n_1_n_1_n_2_n_2_n_3_n_3_*) naked seed lines, meaning that the interaction of fuzzless loci (*N_1_*, *n_2_*, and *n_3_*) could also produce the fiberless phenotype (Turley, 2002). Besides these major loci, there should be other genes modifying the amount of fuzz or lint on the seed, because considerable variation in the fuzzless/fiberless phenotype has been observed in many uncharacterized fiber mutants (Turley and Kloth, 2008).

The *N_1_* and the *n_2_* loci were mapped to chromosomes 12 (A12) and 26 (D12), respectively (Endrizzi and Ramsay, 1980; Samora et al., 1994). However, in a later study, both loci were genetically mapped to A12 by molecular markers (Rong et al., 2005). Recently, the *N_1_* gene was identified by map-based cloning and found to be an *MYBMIXTA*-like (MML) transcription factor 3 on chromosome A12 (*GhMML3_A12*; Wan et al., 2016). *MML3_A12* promotes the initiation of fibers, but its expression is suppressed by a natural antisense transcript (NAT) in dominant naked seed mutants. *MML3* was also named *GhMYB25-like* in an earlier study, and its reduced expression was also found in Xu142 *fl* (Walford et al., 2011). Suppression of *GhMYB25-like* resulted in fiberless seeds (Walford et al., 2011). Another MML transcription factor, *GhMML4_D12*, was thought to be the *li_3_* locus responsible for the initiation of lint fibers in Xu142 *fl* (Wu et al., 2018). Interestingly, *MML3* and *MML4* are coterminous on chromosomes A12 and D12. A recent study proposed that *MML3_D12* also is a major contributing locus for the recessive fuzzless trait of Gb (Zhu et al., 2018). Interestingly, besides these protein-coding genes, some long noncoding RNAs were found to be associated with fiber initiation in the comparison of the transcriptomic differences between Xu142 *fl* and normal lines (Wang et al., 2015; Hu et al., 2018). Huang et al. (2013) identified an R2R3-MYB gene *GbMYB2* involved in fiber initiation and elongation, and this gene also showed a higher expression level in Xu142 compared with Xu142 *fl*. Another R2R3-MYB gene, *GhMYB212*, was also found to regulate fiber initiation and elongation (Sun et al., 2019). In *Gossypium arboreum* (A2-genome), many fuzzless mutants have been found, and they share a common dominant repressor that has been speculated to be the *GLABRA2*-*interacting repressor* (*GIR1*) gene on chromosome 8 (Du et al., 2018; Feng et al., 2019). Recently, a homeodomain-leucine zipper gene *GaHD-1* on Chr06 was found to be the likely causative gene for the lintless trait in *G. arboreum* (Ding et al., 2020; Liu et al., 2020).

In this study, the *li3* locus of Xu142 *fl* was identified by map-based cloning, and the possible variations of DNA sequences in this locus likely responsible for the lintless phenotype were detected. Primary mapping of *n_2_* and *n_3_* loci in *n_2_* was conducted, and a single nucleotide polymorphism (SNP) potentially underlying the *n_3_* mutation was also revealed. Based on the genotyping of F_2_ plants derived from the cross TM-1 × Xu142 *fl*, a new genotype was proposed for the fiberless phenotype of Xu142 *fl*.

## Materials and Methods

### Plant Materials

**Figure 1:**
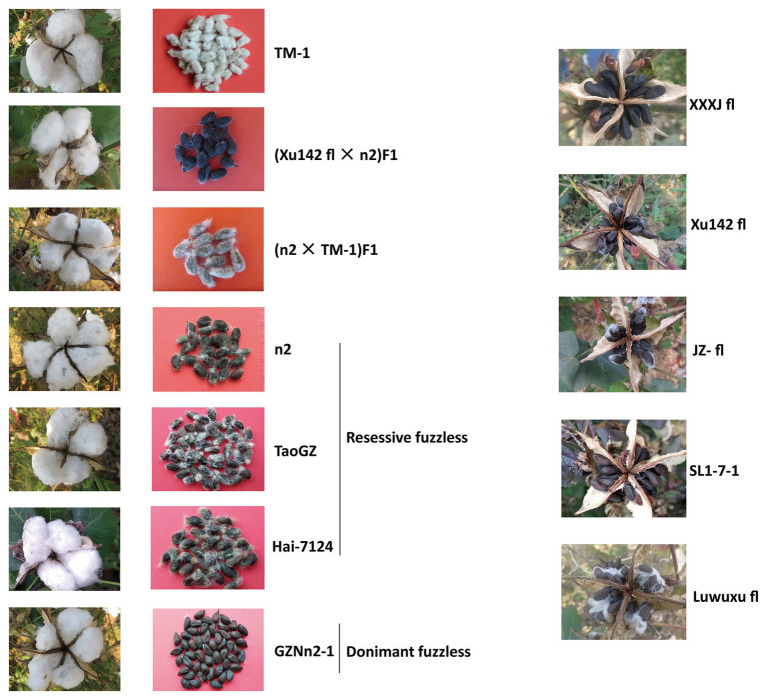
Fuzz and lint phenotypes of TM-1[wild type (WT)] and representative fiber mutants. For linted materials, the **left panel** show seeds with lint (before ginning); the **right panel** show seeds with fuzz (after ginning). Very sparse lint could be found for some fiberless (lintless-fuzzless) mutants.

An F_2_ population derived from Xu142 *fl* × n2 was used for segregation analysis and mapping of the *Li_3_* locus. A BC_1_ mapping population was also developed for fine mapping. Xu142 *fl* and *n_2_* are naturally occurring fiberless and fuzzless-linted mutants, respectively (Figure 1). Xu142 *fl* was found in a commercial Gh variety Xu142 (with normal fuzz and lint) in 1987. For mapping of the *N_2_* and *N_3_* loci, an F_2_ population derived from *n_2_* × TM-1 was used. TM-1 is a standard *G. hirsutum* line with a normal seed phenotype (Kohel et al., 1970). For mapping of the fuzzless loci of Gb, an F_2_ population derived from TM-1 × Hai7124 was used. An F_2_ population derived from TM-1 × Xu142 *fl* was used for the segregation analysis of the *Li_3_* and *N_3_* loci. In addition, 6 fiberless, 25 dominant fuzzless, and 26 recessive fuzzless lines were used for genotyping of *Li_3_* and *N_3_* loci (Supplementary Table S1). For association analysis of *Li_3_* and *N_3_* loci, 387 Gh accessions with normal fiber phenotype were used (including Xu142). All cotton plants were grown in the field at the Experimental Station of the Cotton Research Institute of the Chinese Academy of Agricultural Sciences (Anyang, China). Seed phenotypes were scored based on visual inspection at maturity of seeds, and at least five bolls in a single plant were investigated for each material. Lint percentage data were collected and evaluated following the conventional cotton breeding methods using at least 1,000 seeds and the formula (weight of ginned fibers/weight of seeds before ginning).

### Bulk Segregant Analysis and Mapping-by-Sequencing

For bulk construction, genomic DNA was isolated from young leaves of the 30 selected plants (assumed to have homozygous recessive genotype) and bulked in an equal ratio to form the pool for each segregating population. Pools and DNA of corresponding parents were subjected to sequencing on the Illumina HiSeq 2,500 platform with 100-bp paired-end reads generated. Identification of SNPs was performed according to the procedure described by Zhu et al. (2017). Briefly, after quality check using FastQC, the clean reads were aligned to the TM-1 reference genome (NAU-NBI Assembly v1.1) using BWA software (Li et al., 2009). The GATK Toolkit was used for calling of SNPs (McKenna et al., 2010), and the SNP filtering was performed according to the methods reported by Reumers et al. (2012). Minimum read coverage of SNPs was set to 5 and 10 for parent and pool, respectively. The sequencing depth and coverage for each mapping population are summarized in Supplementary Table S2. Association mapping was conducted using the method proposed by Abe et al. (2012) and Zhu et al. (2018). Only homozygous SNPs between the parents of the population were used for association mapping. For each SNP site, the SNP index was calculated as follows: SNP index = *R/T*, where *R* is the depth of reads of recessive parental types in the pool, and *T* is the total depth of reads in the pool. For the SNP responsible for the recessive genotype or its closely linked SNPs, the SNP indices should be or close to 1. Average SNP indices were calculated using a sliding-window-based approach (1.0 Mb window with 100 kb increments) and plotted for each chromosome. If there are less than five SNPs in a given 1.0 Mb window, the average SNP index value for this window will be ignored.

### Genotyping and Linkage Analysis

The SNPs in the association region of *Li_3_* were converted to PCR-based markers using the modified allele-specific PCR method (Hayashi et al., 2006; Chen et al., 2015), and these markers were used to genotype the plants from the F_2_ population of Xu142 *fl* × *n_2_*. The linkage map was constructed with MapMaker v 3.0 (Lander et al., 1987). Recombination frequency was transformed into the genetic distance (centimorgans, cM) using Kosambi’s mapping function (Kosambi, 1944).

### Sequence Comparison

The candidate gene was amplified from the genomic DNA of the TM-1 and other materials using the primers described in Supplementary Table S3. In addition, the full-length coding fragments of MML3 were also amplified from the −1 DPA-ovule cDNA of TM-1 and Xu142 *fl*. PrimeSTAR GXL high fidelity DNA polymerase (Takara, Dalian, China) was used for PCR. The PCR products were subcloned into a pEASY-Blunt cloning vector (Transgen, Beijing, China), and no fewer than eight clones were sequenced for each PCR product. The cloned sequences were assigned to the At- or Dt-subgenome of the reference genome sequence (Yuan et al., 2015; Zhang et al., 2015). Sequence alignment was performed using ClustalW2.^1^

### Gene Expression Analysis

The expression levels of *MML3_D12* and *MML4_D12* were analyzed by quantitative real-time PCR (qRT-PCR) in ABI Prism 7,500 system according to the manufacturer’s protocol. Developing ovules at −3, −1, 0, 1, 3, and 5 DPA were harvested and immediately frozen in liquid nitrogen. Total RNA was isolated from the whole ovules using the RNAprep Pure Plant Kit (Tiangen, Beijing, China). The total RNA was reverse transcribed to cDNA using a PrimeScript® RT reagent kit with a gDNA Eraser (Takara, Dalian, China). Quantitative PCR (qPCR) was performed using SYBR® Premix Ex Taq™ (Tli RNaseH Plus; Takara, Dalian, China). Cotton *ACTIN14* (GenBank accession number: AY305733) was used as an internal control. Relative expression levels were determined by the ∆*^Ct^* method. For each set of materials, three biological and two technical replicates were used. RT-PCR primers are listed in Supplementary Table S3. PCR efficiency of these primers was determined by LinRegPCR (Ruijter et al., 2009). All qPCR primer pairs had a similar amplification efficiency (91.7–97.3%).

## Results

### Map-Based Cloning of *li_3_*

**Figure 2:**
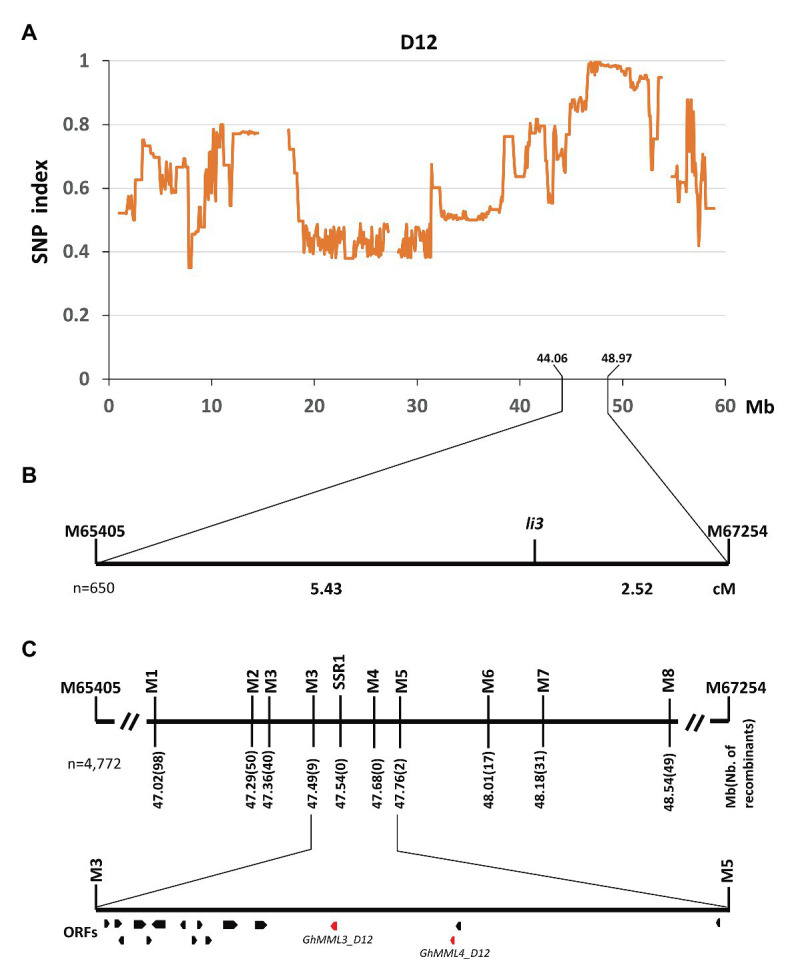
Fine mapping of the *Li_3_* gene. **(A)** The SNP index plot on chromosome D12 (*GhMML3_D12*). Averaged SNP index values in a moving window of 1.0 Mb with 100 kb increments were calculated and plotted. **(B)** Primary mapping of *Li_3_*. cM, centiMorgan. **(C)** Fine mapping of *Li_3_*. Open reading frames (ORFs) are indicated by boxes.

Previous studies concerning the inheritance of the fuzzless/fiberless seed phenotypes showed that Xu142 *fl* differed from n_2_ only at the *Li_3_* locus (Turley and Kloth, 2008). Thus, the cross Xu142 *fl* × *n_2_* was made to generate a segregating population for the mapping of the *Li_3_* locus. Seeds of F_1_ plants were fuzzless but linted, and segregation of linted and lintless seed phenotypes in the F_2_ and BC_1_ populations both fit the expected ratios (3:1 in F_2_; 1:1 in BC_1_; Supplementary Table S4). The DNA pool was generated by bulking 30 lintless individuals in the F_2_ population, and these were subsequently subjected to high-throughput sequencing. A single unique genomic region was found on chromosome D12 (*GhMML3_D12*; Figure 2A). Linkage analysis using two flanking SNP markers showed that the *li_3_* locus was located in the region of 44.06–48.97 Mb (Figure 2B). Further fine-mapping using 1,805 F_2_ and 2,967 BC_1_ plants narrowed down the *li_3_* locus to a ~270 kb region that contained 16 predicted open reading frames (ORFs; Figure 2C, Supplementary Table S5). Out of these ORFs, two genes coding *AtMYB16*/*AmMIXTA* orthologs (*GhMML3_D12* and *GhMML4_D12*) appear to be the candidates for *Li_3_*, as they are the only genes shown to be preferentially expressed in ovules during the fiber initiation stage (−3 to 5 DPA) in the previous analyses (Bedon et al., 2014; Zhang et al., 2015; Wu et al., 2018; Supplementary Table S6). However, for *GhMML4_D12*, sequence alignment did not find any likely causal variation for the *li_3_* mutation (please see detailed description in the later section “*li_3_* and *n_2_* might be the multiple alleles of *GhMML3_D12*”).

**Figure 3:**
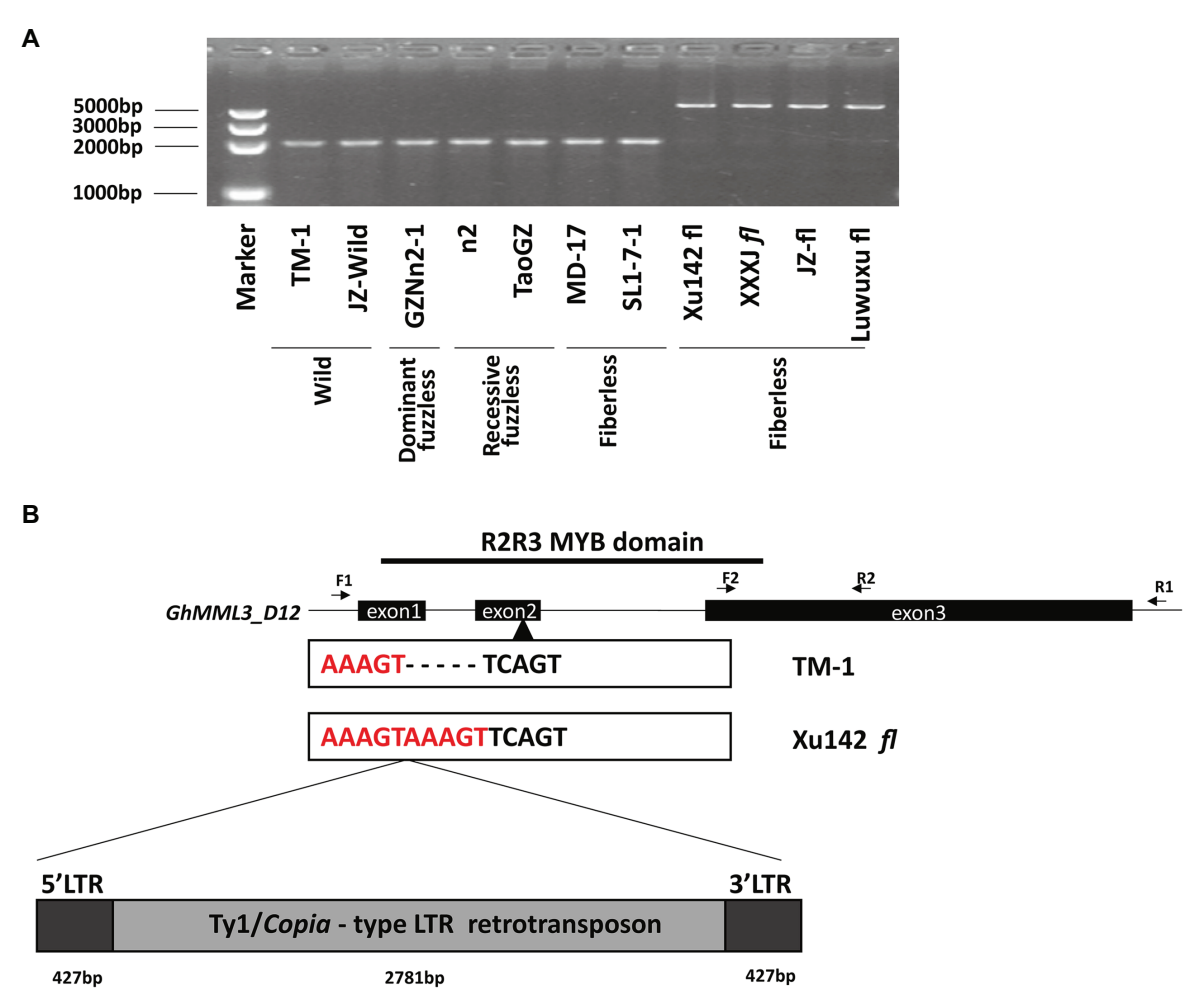
The sequence variation between TM-1 and Xu142 *fl*. **(A)** Detection of retrotransposon insertion using F1/R1 primer pair. **(B)** Structure of the *GhMML3_D12*. F1/R1 primer pair was used for the amplification of the full DNA sequence of *GhMML3_D12*. The F2/R2 primer pair was used for quantitative real-time PCR (qRT-PCR).

When amplified by subgenome-specific primers for *GhMML3_D12*, genomic DNA of Xu142 *fl* displayed a longer PCR product compared with those of expected length in normal and fuzzless-mutant lines, and the same size products were also found in three other fiberless mutants (Figure 3A). However, the other two fiberless lines, MD17 and SL1-7-1, displayed a normal-length amplification. The full-length genomic sequences of *GhMML3_D12* of Xu142 *fl* and three other fiberless mutants showed that they all carried a 3,635-bp insertion fragment in the second exon (Figure 3B). Further sequence analyses indicated that the insertion fragment is a putative Ty1/*copia* long terminal repeat (LTR) retrotransposon (named *Ghli3_ret* hereafter). The components of a typical Ty1/*copia* element, such as LTRs, gag, and pol, could be found in *Ghli3_ret* (Supplementary Figure S1). The retrotransposon consists of a 427-bp 5′-LTR, a 2,781-bp internal region, and a 427-bp 3′-LTR. The sequences of the two LTRs are entirely the same, which suggests that this insertion event is relatively recent. A further screening using *li_3_*-specific primers (forward primer in Intron 1 and reverse primer in 5′LTR; Supplementary Table S3) in 387 Gh and 373 Gb cultivars showed that the *Ghli3_ret* insertion only existed in these four fiberless mutants (Supplementary Tables S7, S8). The marker assay also showed that this insertion was absent in Xu142.

**Figure 4:**
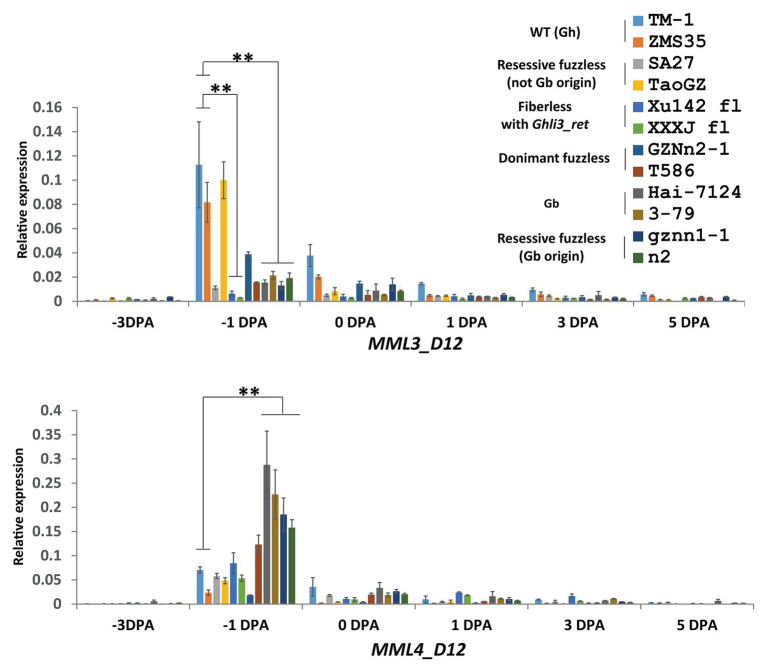
Quantitative real-time PCR measurements of *MML3_D12* and *MML4_D12* in ovule development. Error bars indicate the standard deviation of three biological replicates. Significant differences between groups are indicated by an asterisk (^**^p < 0.01) according to Student’s *t*-tests.

The *Ghli3_ret* insertion in *GhMML3_D12* might block the expression of the gene. qRT-PCR was used to analyze the expression level of the *GhMML3_D12* transcript at different development stages of ovules. Compared with TM-1, the transcript abundance of *GhMML3_D12* was significantly reduced in Xu142 *fl* at −1 and 0 DPA stages (Figure 4). Sequencing of cDNA using primers universal to the At- and Dt-subgenome alleles also indicated that *GhMML3_D12* in Xu142 *fl* might not be expressed, because all sequenced clones were *GhMML3_A12* type (15 out of 15). Actually, Walford et al. (2011) also reported the absence of *GhMML3_D12* mRNA in Xu142 *fl*. These results indicated that the insertion of *Ghli3_ret* interrupted the transcription of *GhMML3_D12*, the best candidate for *Li_3_*.

### Primary Mapping of *n_2_* and *n_3_*

**Figure 5:**
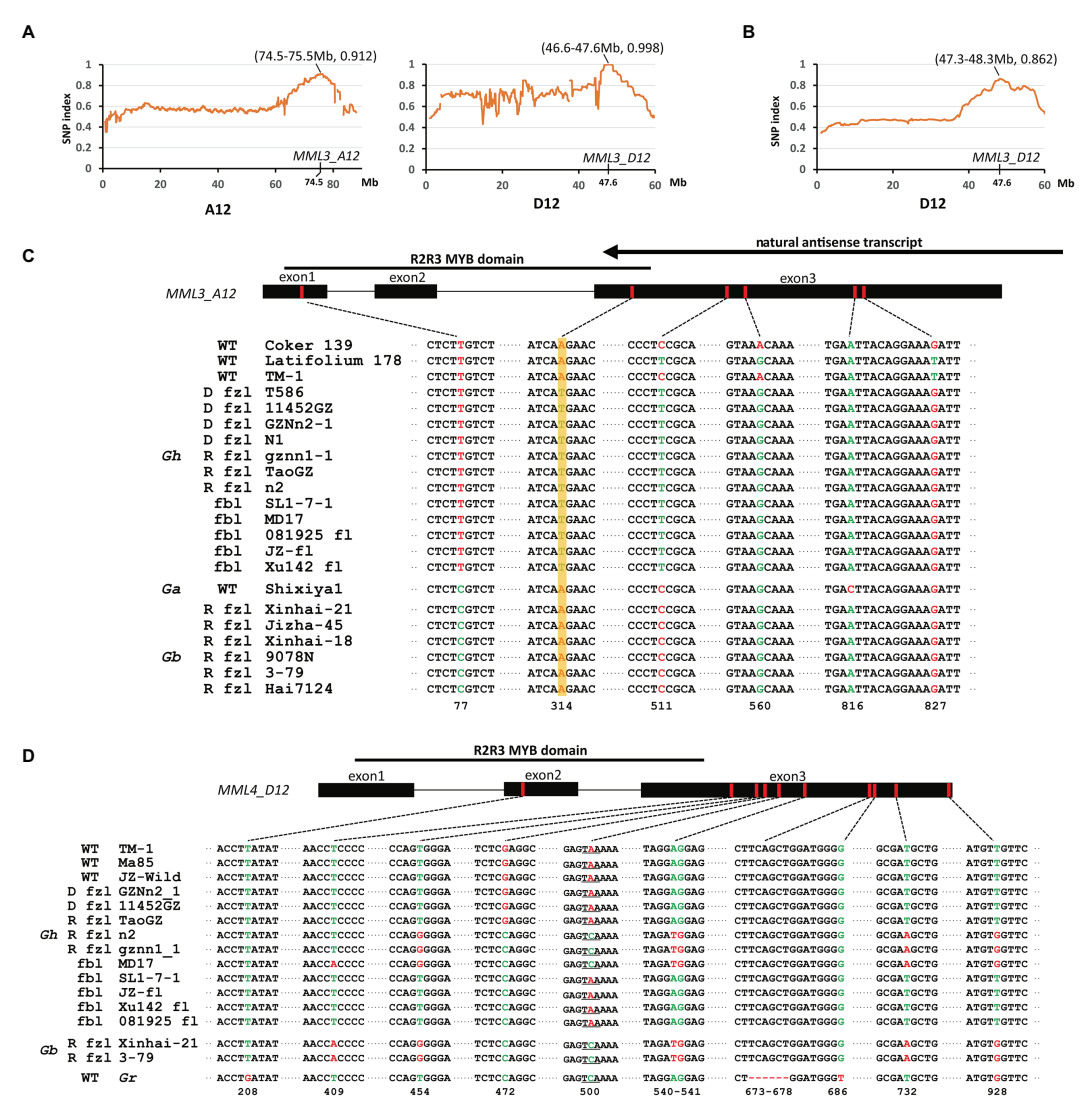
Mapping of the recessive fuzzless genes. **(A)** The SNP index plot on chromosomes A12 and D12 using the F_2_ population of n2 × TM-1. **(B)** The SNP index plot on *GhMML3_D12* using the F_2_ population of TM-1 × Hai7124. Averaged SNP index values in a moving window of 1.0 Mb with 100 kb increments were calculated and plotted. The window with the largest index value is shown in brackets. **(C)** Non-synonymous mutations in *MML3_A12.* D fzl, dominant fuzzless; R fzl, recessive fuzzless; and fbl, fiberless. The arrow indicates the NAT of *GhMML3_A12* found by Wan et al. (2016). **(D)** Non-synonymous mutations in *MML4_D12*.

Previous studies showed that the recessive fuzzless mutant *n_2_* might differ from normal cotton at the *N_2_* and *N_3_* loci (Turley and Kloth, 2002). Thus, the cross *n_2_* (*n_1_n_1_n_2_n_2_n_3_n_3_*) × TM-1 (*n_1_n_1_N_2_N_2_N_3_N_3_*) was made to generate a segregating population for mapping of these two loci. F_1_ plants show the fuzzy phenotype, though the fuzz density of seeds is lower compared with that of TM-1 (Figure 1). The fuzz phenotype of F_2_ plants was investigated by visual inspection. The results showed that segregation of fuzzy and fuzzless phenotypes fit the expected ratio (15:1; Supplementary Table S4). However, it should be noted that there was a broad variation in fuzz density for those fuzzy plants, and only a fraction showed similar density with that of TM-1. Again, the strategy of bulked segregant analysis (BSA) combined with whole-genome resequencing was used. The DNA pool was generated by bulking 30 fuzzless individuals (with genotype assumed to be *n_1_n_1_n_2_n_2_n_3_n_3_*) in the F_2_ population with subsequent high-throughput sequencing (Illumina HiSeq × Ten platform). Two candidate genomic regions for *n_2_* and *n_3_* were found in BSA analysis: ~47–48 Mb on chromosome D12 and ~74–75 Mb on chromosome A12 (Figure 5A). Interestingly, the region on D12 overlapped with the location of *Li_3_*, and the region on A12 overlapped with that of *N_1_*. *GhMML3* or *GhMML4* again was considered to be a good candidate for *N_2_* or *N_3_*, because in these regions, no other gene showed preferential expression in ovules during the fiber initiation stage (−3 to 5 DPA) in the previous RNA-Seq data (Zhang et al., 2015; Supplementary Table S6).

### *n_3_* Is One of the *GhMML3_A12* Alleles

*GhMML4_A12* was found to maintain a very low expression level in ovules throughout different developmental stages in previous and the present studies (Zhang et al., 2015; Supplementary Figure S2), and no sequence variation in the coding sequence (CDS) region was found between mutants and the wild type (WT; Supplementary Figure S3), so it is unlikely to be the candidate gene for these two loci (*N_2_* and *N_3_*). In *GhMML3_A12*, sequence alignment found an SNP (A314T) common among n2, Xu142 *fl*, and GZNn2-1 that caused a non-synonymous coding mutation (K105M) in the DNA-binding domain of this gene (Figure 5C; Supplementary Figures S4, S5). The screening of 387 normal Gh lines and 57 fiber mutants using a diagnostic marker based on this SNP showed that this mutation was found in all fiber mutant lines, no matter whether they were fuzzless (dominant or recessive) or fiberless (Supplementary Tables S1, S7). In previous studies (Turley and Kloth, 2002, 2008), the dominant fuzzless mutants have been shown to contain the *N_2_* allele, so this SNP should be the underlying mutation for the *n_3_* locus. That is, if this SNP is the causal mutation for the *n_2_* locus, those dominant fuzzless mutants would contain the *n_2_* allele, which is contradictory to the results of the previous studies. Interestingly, Wan et al. (2016) proposed that the extremely low expression of *GhMML3_A12* in *N_1_* is associated with NAT production (Figure 5C). This NAT is highly active in the dominant fuzzless mutants and suppresses many MML genes besides *MML3*. In normal and recessive fuzzless lines, this NAT is weak or absent (Wan et al., 2016). Thus, the *n_3_* and *N_1_* loci are actually different mutation sites in and around *GhMML3_A12*, and both disrupt the normal function of *GhMML3_A12*.

The marker assay showed that only six normal lines contained the *n_3_* allele (Supplementary Table S9), so it appears to be a rare allele in normal lines (allele frequency was 0.015). The screening in 373 Gb accessions showed that this allele was also missing in Gb lines (Supplementary Table S8). Thus, all Gb lines seem to contain the *N_3_* locus, though they are all fuzzless.

Another SNP (C511T) in *GhMML3_A12* was also common among n_2_, Xu142 *fl*, and GZNn2-1, and this SNP also caused a non-synonymous coding mutation (P171S; Figure 5C; Supplementary Figures S4, S5). However, this SNP was found in many normal lines in the previous genome-wide association study (GWAS) analyses, and no significant association was found with any surveyed traits (Wang et al., 2017; Ma et al., 2018). Furthermore, this SNP is located outside the MYB domain of *GhMML3_A12*, so it should be excluded as the causal mutation for *n_3_*.

To trace the origin of the *n_3_* locus, 51 fuzzless and 405 normal Gh wild accessions were surveyed using the diagnostic marker based on the A314T mutation (Supplementary Table S10). The *n_3_* mutation was found in only two fuzzless accessions, marie-galante 38 (TX-881) and marie-galante 39A (TX-882), and it was absent in all normal wild accessions.

Interestingly, Xu142 was found to contain the *N_3_* allele in the marker assay. That is, it contained wild-type genotypes (*Li_3_Li_3_N_3_N_3_*) for both *li_3_* and *n_3_* loci. Thus, it is possible that Xu142 used in the present study is not the original line (Xu142 *fl* derived). Actually, Xu142 was also found to differ from Xu42 *fl* at more than two loci in another study (Hu et al., 2018). In future study, attention should be called to the truth of Xu142, considering that the original line might be lost because of the low probability of opening pollination during germplasm conservation.

### *li_3_* and *n_2_* Might Be the Multiple Alleles of *GhMML3_D12*

Because *n_3_* is located in *GhMML3_A12*, the *n_2_* locus should be in the other candidate genomic region on D12 that overlaps with the location of *li_3_* (Figure 5A). The sequence alignment showed that genomic sequences of *GhMML3_D12* and *GhMML4_D12* of n2, gznn1-1, and MD17 were the most similar to those of Gb reference genomes (Liu et al., 2015; Yuan et al., 2015). Considering that nearly all Gb accessions in nature are fuzzless, the *n_2_* allele of these mutants might have been transferred from fuzzless Gb lines. For *GhMML3_D12*, no sequence variation in CDS was found to be specific for mutants proposed to contain the *n_2_* allele (Supplementary Figure S6). In the promoter region of *GhMML3_D12*, a 480-bp insertion at the −252 site (the star codon set as the zero site) was found when compared with *GhMML3_A12* and other homologs in *Gossypium raimondii* (D5-genome) and *G. arboreum* (Supplementary Figure S7). This insertion exists in both Gh and Gb. A 7 bp target site duplication sequence (ACATAGT) was found beside the insertion. Alignment analyses showed that sequences similar to this insertion widely distributed in the genomes of *Gossypium* species, but were missing in other organisms (Supplementary Table S11). Thus, this insertion appears to be a transposon specific to cotton. Interestingly, in the insertion, an 80-bp deletion was found in some Gh lines, differing from the 22-bp deletion in Gb, n2, gznn1-1, and MD17. Several other sequence variants were found between Gh and Gb in the promoter region. When using a diagnostic marker based on the differences in the promoter region between Gh and Gb lines, 9 out of 24 recessive fuzzless lines were found to be of Gb origin (Supplementary Table S1).

For *GhMML4_D12*, sequence alignment found that a non-synonymous coding mutation (G472C cause E158Q) was common for *n_2_*, gznn1-1, Xu142 *fl*, MD17, and 2 Gb reference lines (Figure 5D; Supplementary Figure S8), so this appears to be a candidate variation for the *n_2_* locus, considering that all these lines were proposed to contain the *n_2_n_2_* genotype in previous studies (Turley and Kloth, 2002, 2008). However, this variation was also found in some of the dominant fuzzless lines (14 out of 23) that were thought to contain the *N_2_* allele (Supplementary Table S1). This SNP variation was also found in the GWAS analysis, and it appeared to be arranged randomly in the 352 cotton accessions (Wang et al., 2017). Another SNP (C500A) that resulted in the early termination of *GhMML4_D12* translation because of the formation of a TAA stop codon was found in all Gh lines surveyed, except for *n_2_*, gznn1-1, MD17, and other Gb origin recessive fuzzless lines. Thus, this SNP should not be the causal mutation of the *n_2_* locus, because it was also found in the normal Gh lines that were thought to contain the *N_2_* allele. In addition, this mutation was located outside the MYB domain of *GhMML4_D12*, and it might not affect its DNA-binding function. Interestingly, the expression level of *GhMML4_D12* was much higher for −1 DPA-ovules in Gb and Gb origin recessive fuzzless lines compared with those with the TAA mutation (Figure 4), further suggesting that this gene might not be the candidate for *N_2_*.

Overall, no mutation was found common among lines containing the *n_2_* allele (based on previously proposed genetic models) in the genomic sequences of *GhMML3_D12* and *GhMML4_D12* in the present study. However, the expression level of *GhMML3_D12* was much lower for −1 DPA-ovules in lines assumed to contain the *n_2_* allele compared with the WT (Figure 4). Considering that transcription of *GhMML3_D12* was also completely interrupted in the *li_3_* mutants, *li_3_* and *n_2_* might be multiple alleles of the *GhMML3_D12* gene (so *li_3_* was renamed as *n_2_^Xu^*).

### Mapping of Fuzzless Loci in Gb

Considering that all Gb cultivars are fuzzless and that some of the recessive fuzzless Gh lines were of Gb origin, it was essential to map the fuzzless loci in Gb. An F_2_ population derived from the cross TM-1 × Hai7124 was developed, and the fuzz phenotype was investigated by visual inspection. Fuzz density of seeds in the population seemed to display a continuous distribution, from TM-1-like to Hai7124-like. Of the 1,117 F_2_ plants, only 39 plants displayed naked seeds and the fuzz density of those seeds was even lower than that of Hai7124, indicating that the fuzzless seed trait of Hai7124 might be regulated by multiple loci. The DNA pool was generated by bulking these fuzzless individuals and was subsequently subjected to high-throughput sequencing (Illumina HiSeq × Ten platform). Only a single genomic region on D12 was found significant in association mapping, and this region overlapped with those of the *Li_3_*/*N_2_* loci (Figure 5B). This location is also the same as the major locus mapped in Gb using another cross (Zhu et al., 2018), though another four minor loci were also identified in their study. These results indicate that the *n_2_* allele originated from Gb.

### Genetic Analysis of Lintless/Fuzzless Loci in Xu142 *fl*

**Figure 6:**
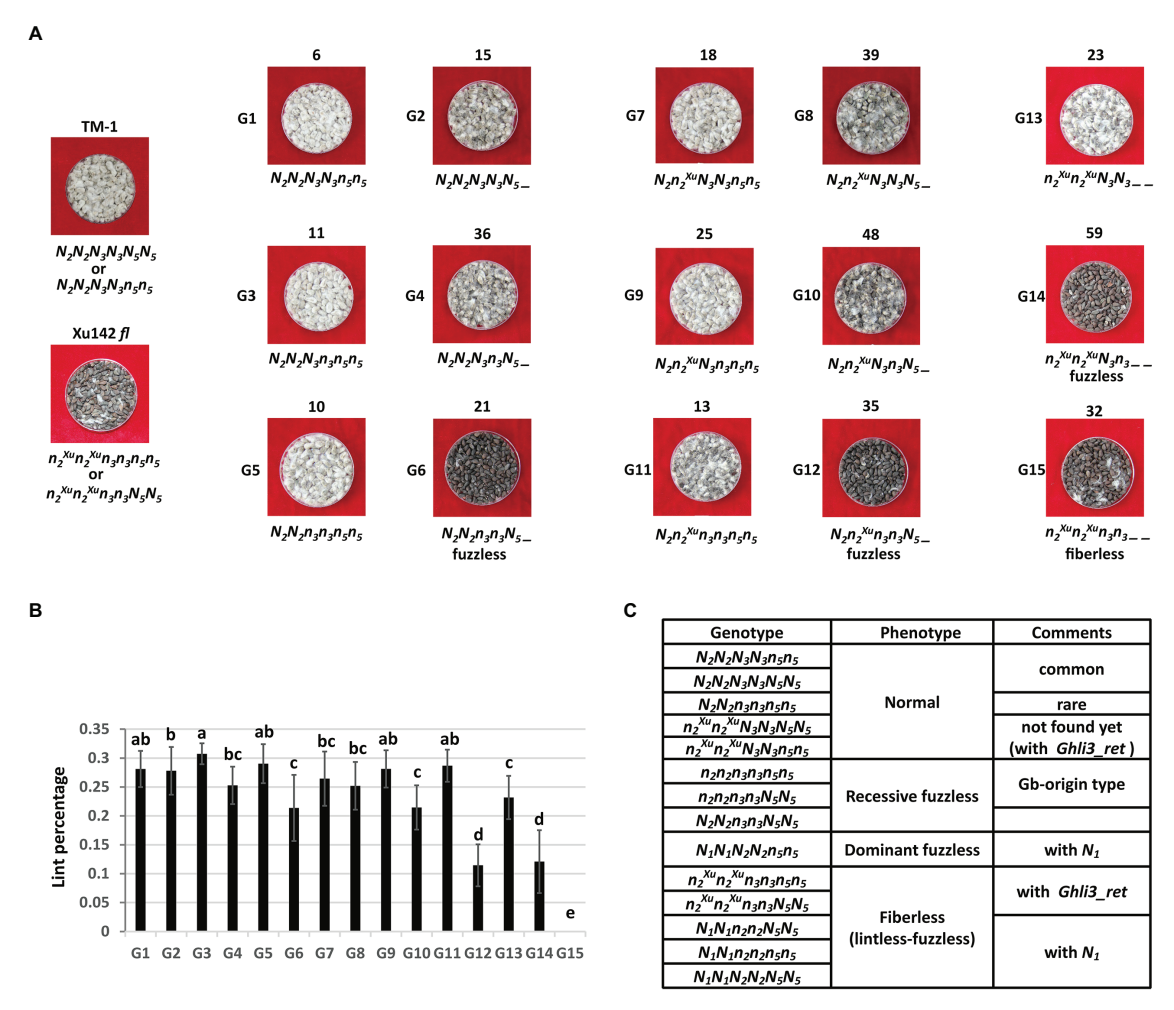
Segregation of the fiber traits by *N_2_*/*N_3_*/*N_5_* combination in the F_2_ population of TM-1 × Xu142 *fl*. **(A)** Fuzz phenotype of parents and representative F_2_ plants. The number on the top is the number of plants in each group. The proposed genotype is on the bottom. *N_2_*, *n_2_^Xu^* are WT and Xu142 *fl* alleles in the *N_2_* locus, respectively. **(B)** The lint percentage of each group. Error bars indicate standard deviations. Multiple comparisons were conducted by Duncan’s new multiple comparisons test. **(C)** Proposed genotypes for WT cotton and fiber mutants by *N_1_*/*N_2_*/*N_3_*/*N_5_* combination.

To further dissect the inheritance model for the fiberless trait of Xu142 *fl*, the genotypes of *Li_3_/N2* and *N_3_* loci of F_2_ plants from the cross TM-1 × Xu142 *fl* were surveyed by PCR markers based on the mutations responsible for these two loci in *GhMML3_D12* (*Ghli3_ret* insertion) and *GhMML3_A12* (A314T), respectively (Figure 6A). In the background of *N_2_N_2_* (homozygous *Li3* allele), two groups of fuzz density were observed in each genotype of the *N_3_* locus. It seems that there was a third locus (named *N_5_*) displaying an inhibitory effect on the expression of *Li_3_* (*GhMML3_D12*) because of the ratio of lower fuzz density plants: higher fuzz density plants was close to 3:1 in each genotype of the *N_3_* locus. The combination of *N_2_N_2_n_3_n_3_N_5__* resulted in the fuzzless phenotype. In the background of *N_2_n_2_^Xu^*, the *N_5_* locus still displayed a suppression effect on the *Li_3_* locus, because two groups of fuzz density were still observed in each genotype of the *N_3_* locus. The combination of *N_2_n_2_^Xu^n_3_n_3_N_5__* resulted in the fuzzless phenotype. In the background of *n_2_^Xu^n_2_^Xu^*, only a single fuzz density was observed for each genotype of the *N_3_* locus. Thus, the *N_5_* locus seems to show no inhibitory effect on the expression of *N_3_* (*GhMML3_A12*). The combination of *n_2_^Xu^n_2_^Xu^N_3_n_3__ _* resulted in the fuzzless phenotype, and the double homozygous recessive genotype (*n^li3^n^li3^n_3_n_3_*_ _) displayed the fiberless phenotype.

The *N_5_* locus might also repress the initiation of lint. The average lint percentage of those plants with the dominant *N_5__* genotype was usually lower than that of plants with *n_5_n_5_* in the same genotype combination of *Li_3_/N_2_* and *N_3_* loci (Figure 6B). Interestingly, the fuzzless *N_2_N_2_n_3_n_3_N_5__* plants showed a much higher lint percentage than the other two types of fuzzless plants (*N_2_n_2_^Xu^n_3_n_3_N_5__* and *n_2_^Xu^n_2_^Xu^N_3_n_3__ _*).

Based on these results, a four-locus genetic model could be proposed for Xu142 *fl* and other fiber mutants when taking into account the *N_1_* locus (Figure 6C). In this model, only dominant fuzzless mutants have a single genotype.

## Discussion

For the fine-mapping of the *li_3_* (*n_2_^Xu^*) locus, the same parents and types of mapping population were used between this study and that of Wu et al. (2018), and the same mapping region was also obtained. The SNP site (C500A) that resulted in the early termination of *GhMML4_D12* was regarded as the causative mutation for *li_3_* in their study. However, our results showed that this mutation was universal in Gh lines, and native *MML4_D12* was only found in Gb and recessive fuzzless Gh lines with *n_2_* allele (Gb origin). Like Xu142 *fl*, the wild-type Gh lines also displayed a much lower expression level of *GhMML4_D12* compared with Gb and recessive fuzzless lines (with *n_2_* allele). Furthermore, the RNA interference suppression of *GhMML4* did not result in the lintless phenotype in their study. These results indicated that *GhMML4_D12* should not be the candidate gene for *li_3_*. In this study, a retrotransposon insertion in *GhMML3_D12* was found, and the expression of this transcript was accordingly disrupted. The insertion was only found in four fiberless mutants. Furthermore, Walford et al. (2011) found that the reduced expression of *GhMML3* resulted in a fiberless phenotype. Thus, the insertion in *GhMML3_D12* is likely the real underlying mutation for *li_3_*.

In the present research, the fiberless mutant Xu142 *fl* was found to be a double mutant line (*n_2_^Xu^n_2_^Xu^n_3_n_3_*), and the mutations in the copies of *GhMML3* on A12 and D12 were sufficient to explain the fiberless phenotype in the F_2_ population. *GhMML3_D12* was found to be a good candidate for the recessive fuzzless *n_2_* locus in Gh, and it also appeared to be a major locus underlying the fuzzless seed trait in Gb. Furthermore, the *n_3_* locus appeared to be a prerequisite for all the naked or fiberless seed mutants in cultivated Gh lines. The RNA interference suppression of *MML3* resulted in fiberless seed phenotypes similar to that of Xu142 *fl* and reduced expression of other fiber-expressed MYBs (such as *GhMML7*/*GhMYB25*, *GhMML8*, and *GhMML9*) was also found in these silenced plants (Walford et al., 2011; Bedon et al., 2014). In a transcriptome profiling study of five fiber mutants, a number of transcription factor families (such as MYB, WRKY, bZIP, and bHLH) were found to be differently modulated between WT and mutants (Wan et al., 2014). Thus, the present and previous results imply that *GhMML3* is a key factor having a prominent effect on fiber initiation and that it may function as an upstream gene in a regulatory cascade for fiber initiation (Hu et al., 2016). However, the discovery of the *N_1_* and *N_5_* locus shows that there are some upstream regulatory factors modulating the expression of *GhMML3*.

The K105M in the DNA-binding domain of *MML3_A12* appeared to be the best candidate for *n_3_* in the present study. This residue (K105) is located in the DNA-binding motif of the MYB domain, and no variation of this site has been found among different plant species (Supplementary Figure S5). The mutation might disrupt the DNA-binding activity of *MML3*, so *n_3_* should be a loss-of-function allele. The genetic analyses in the F_2_ population of the cross TM-1 × Xu142 *fl* also indicated that *n_3_* was a recessive allele. A recent GWAS (using 352 cotton accessions) showed that this SNP allele was missing in all of the normal cotton except for one line, 611Bo (Wang et al., 2017). In another GWAS (using 419 cotton accessions), only 10 out of 335 normal-seed lines were found to contain this allele (Ma et al., 2018; Supplementary Table S9). In both GWAS, all of the fiber mutants contained this SNP site. Thus, this mutation appears to be a prerequisite for the naked or fiberless seed in Gh. It appears to be a rare allele in normal lines, which is also reasonable based on the four-locus genetic model deduced in the present study. In the background of *N_2_N_2_n_5_n_5_*, the plants with the *n_3_n_3_* genotype could still display normal fuzz phenotype. In addition, this SNP site was also found to be significantly associated with lint percentage in the GWAS analysis of Gh lines (Ma et al., 2018).

There are different mapping results for the *n_2_* locus in previous studies. In Endrizzi and Ramsay (1980) *n_2_* was mapped to D12, but in later studies, it was mapped to A12 (Rong et al., 2005; Li et al., 2019). Song et al. (2010) found that the *n_2_* locus could be mapped to A12 or D12, depending on the mapping population used. Actually, some of these studies may have ignored the segregation of the *n_3_* locus in mapping populations, thus leading to the confusing mapping results for *n_2_*. Because of the broad variation in the fuzz phenotype in some mapping populations, some fuzzy plants with low fuzz density would be classified into the fuzzless group. Thus, at times, the fuzz phenotype from some mapping populations would fit the expected ratios based on monogenic inheritance in segregation analyses, even though *n_2_* and *n_3_* were both segregating.

In this study, all four fiberless mutants from China were found to contain the retrotransposon *Ghli3_ret* in *GhMML3_D12*, and the sequences of these *Ghli3_ret* were all the same. The *Ghli3_ret* insertion in these mutants might have the same origin, though the pedigree of those lines is missing. However, the *n_3_* allele was found in all fiber mutants of cultivated Gh, and it would be a low probability event if all of these mutations happened independently. The genetic analyses in previous and the present studies indicated that the fuzzless/fiberless traits of all fiber mutants in Gh have polygenic inheritance. Thus, it is possible that these mutants actually are natural combinations of some rare mutations of different gene loci existing in normal lines. Actually, for the cross TM-1 × Xu142 *fl* used in the present study, all F_2_ plants with *n_2_^Xu^n_2_^Xu^N_3_N_3_* and some plants with *N_2_N_2_n_3_n_3_* genotypes displayed the normal seed phenotype. Although the *n_2_^Xu^* allele was not found outside the fiberless mutants used in the present study, it might exist in some normal lines currently not surveyed.

The locations of loci mapped in the present study indicated that the initiation of fuzz and lint seems to be under the same (or partly overlapping) gene regulatory network. The genetic analysis using the F_2_ plants of TM-1 × Xu142 *fl* indicated that the lintless phenotype resulted from the additive effects of the two fuzzless loci. The fiberless phenotype of the synthetic line MD17 is an example of this kind of additive effect (Turley, 2002). Actually, the lower lint percentage had been observed in most of the fuzzless mutants (Supplementary Table S1). These results are not surprising considering *MML3_A12* and *MML3_D12* affecting the initiation of fiber with an additive manner. However, no fuzz-lintless mutant has been found in Gh or Gb so far. Thus, some key genetic loci (currently unknown) responsible for lint initiation might be at the upstream position of the regulatory network that also controls the initiation of fuzz.

The variation responsible for the *n_2_* allele still needs to be examined in further studies. Only one SNP (C76T) in the CDS region of *MML3_D12* was found to be specific to materials containing the *n_2_* allele. However, this SNP is a synonymous mutation and is located outside the DNA-binding motif. There are several sequence variants in the promoter region of *MML3_D12* between Gh and Gb, and some of them might be responsible for the lower expression level of *GbMML3_D12*. A promoter activity assay with different combinations of promoter sequences is necessary to find the key region regulating the expression of *MML3_D12*.

## Data Availability Statement

The sequences of MML4_A12 (GenBank accession number: MN510689–MN510696), MML3_D12 (MN510697–MN510712), MML3_A12 (MN510713–MN510729), MML4_D12 (MN510730–MN510742), MML3_D12 promoters (MN510743–MN510757), and Ghli3_ret (MN486221) have been deposited in the GenBank database.

## Author Contributions

WC, YLi, SZ, JY, and YZ designed the research, performed most of experiments, and analyzed the data. SF, LZ, YG, JW, LY, FL, and YLu performed part of the experiments. WC and YZ interpreted the results and wrote the manuscript. All authors contributed to the article and approved the submitted version.

### Conflict of Interest

The authors declare that the research was conducted in the absence of any commercial or financial relationships that could be construed as a potential conflict of interest.

## Footnotes

^1^http://www.ebi.ac.uk/Tools/msa/clustalw2/
