# Supplementary material for: A Retrotransposon Insertion in GhMML3_D12 Is Likely Responsible for the Lintless Locus li3 of Tetraploid Cotton
Source: Front Plant Sci. 2020 Nov 26;11:593679. doi: 10.3389/fpls.2020.593679 (PMC7725795; doi:10.3389/fpls.2020.593679)
Supplement: Supplementary file 1 [file Data_Sheet_1.zip › Fig S1-Fig S8 and Table S1-S11/Fig S1.pdf]

Fig. S1. Sequence of Ty1/*copia* LTR retrotransposon *Ghli3_ret*. The conserved domains were identified by searching NCBI's conserved domain database (CDD).

AAAGTTTGTTGCGGAAGCGACGATAATATTAATAACAATATTATCAGAAATATTTAGATA
ATTATTATGCCAACAATTATACTAAGAAAATTCCCAGTTAAATTGGAGGGGTCACAATG
GTCCCGCTTAAAACCCAACAACTAGACAGAACTCACTTAGATATTTATAGCAATAATAA
CTAAATAAATATAACCAACATAAAGCTATTAAATAAAAGCAAACAAATAAGAAATAAAA
TACCAGAGTTTTAACGAGGTTCGGCCAATTTAGCTTACGTCCTCGGACACTACCAAATT
AATATTTCCTCTAGAAATATTACAAAGGAGAAGATTTTGGGATAATACAACCAAAGGGG
GAGGGGTTCTATATATAACAAACCAAACCCCCTCCATTTCTATCTTTTCCTAATGTGGGA
TATTGCCAATATTCAACAAATCTCCACCTTGGCGATATTCCACATCTTCGAACATCTTCT
CATAACACAAACTTCAATAGTGTCTTCAATTTTGCAACCAATCGCCTTCTTCCCTTTTGG
TAGTTGTGCCAGCTTCCACATCTTGTTTTTCTCTAGAGATTGCATTTCCTCTTCCATTGC
ACCTAACCATCTATAATTCTCTAAACTCTGAATGGCTTCGGTGTAAGTGGATGGAATATT
ATCAACAACTGGAAGTGCATAAGCTACCATGTCAGTGAAACGAGCAGGTTTCTGAATT
TCTCGTCTCTGCCTTCTGACTGCAATTGGCTCTGATTGCTGTTGAGGTTCTTGGGTAGA
AACCTCTTCCTCATCTGACTCTGCATCTGCCAATGAAGAATCACTAGTGGTACCTTTTG
CTGGAATTACCACACTCTGCTCAAACTCCACCTGCTGCGGAGTACACTCCACCTGCTG
CGAAGTACTACTCACTCCTTCTGGATTTACCTTATTCAACATGGCAGATTCATGAAAGG
TAACATCCCTACTGATTATCGTCTTCTTTGCCTCTAGACACCACAAACGATATCCCTTAA
CACCAGCATTGAAGCCCATGAATAGAGCCTTCTTTGCTCTTGGATCTAACTTTGATTCTT
TCACATGATAATATGCAATAGAACCAAAAATGCGCAAGGTGTCATAATCAGTAGCAGGT
TTTCCATACCATTTCTCCAAAGGAGTCTTGCCATTTATAGCAGATGATGGCAAATGATTG
ATGAGATGTTGAGCGTACGTCACAGCCTCAGTCCAAAACTTTCTATCTAATCCAGCATT
AGATAACATACATCGAACTTTCTCCACAAGCGTTCGATTCATACGCTCTGCCACCCCATT
CTGTTGCGGTGTTCCACCTACGGTGAAGTGCCTTACTATGCCACAATCTTGGCATATCTT
CAGGAAAGGATCGCTTTTATATTCTCCACCGTTGTCTGATCGGAGAACCTTAATCTTCCT
TCCTGTCTGATTTTCAACTTTAGTTTTCCACTTAAGGAAAACTTCAAGCACCTCATCTTT
GTTCTTCATAGGAAACACCCAAACTCGTCTGGAAAAATCATCAATAAAGGTGACAAAA
TAACGTCTTCCTCCAAGTGATGGAGTCTTGGACGGTCCCCATACATCAGAATGCACATA
ATCCAGAATACCTTTAGTATTGTGAATCCCAGTGCCAAATTTCACCCTTCGTTGTTTTCC
CAGAACACAATGCTCGCAAAATTCAAGTTTGCAAGTCTTTGTACCTTTCAATAATCCTT
GCTTGGCTAGAGTTTGCAAGGATTTTCACCAGCATGGCCCAAACGCATATGCCACAG
CTGTGTTGCCTCAGCGTCCTTCTTGGTACTCGTAATTGCTGCTGCTGTTGTCCCGACCA
CTGTACTACCTTGGTAGTAATACAGATTGTTCTTTCTTATGCCTTTCAACATCACCAATG
CTCCTGAAGTTGCTTTAAGAACTCCATCTCGTATTGTCACAACTAGGCCTTTAGATTCTA
ACGACCCCAAAGAGATGAGATTCTTCTTCAACTTTGGGACATATCGTACATCCCGCAAA
ATTCTAGTAGATCCATCATGACTCCTCAGTTTAATTGAACCTATCCCGGCTATTTTACATG
TGTTATCATTACCCATGTAAACAACCCCTTCATCTAGTTTTTGAAATTCAAAGAACCATT
CCCGAATGGGACACATATGATAGGAACAACCAGAATCCATGATCCACTCATCTGCATAT
AATGTTGAAGATGTCATACTAAGAGAAAAGTCTGACTCTGTTTCACTATTGCATTCTGC
AACATTTGCATCTTGCGGAGTCTTCCCTTTTTTCTTCAATTTTGGACAATCTTTCTTCCA
ATGTCCTTTTTCTTTGCAAAAGAACATTCATCTTTGGCAACAGCTCGACCTTTGGACTT

TCTTCTCTTCCTATTAGACTGACTTTGCTGACGACCTCTTGTTACCAATGCTTCCACTGC
TGCTTCACCTGAACCTTTCAACTTATCCTTTCGGCGTAGTTCATAGCTATACAATGCAGC
AGTTACTTCATTGAAAGTTACTTCCGATTTTCCATGAAGTAGAGTAGTTTCAAGGTACT
CAAACTCCTCAGGAAGCGATCCCAACAACATTAACGCCAAGTCTTCGTCTTCAAAAGT
CACATCCAAATTCAACAAATCAGCCACCAGCTGATTAAAATTGGTAATGTGCTCGTTCA
TCGTGGTACCAGGAACATAACTGAAACGAAACAGTCTTTTCTTCATATGTAACTTATTTT
GACTGTTCTTCTTCAGAAATTTCTCCTCCAATGCTTTCCACAATTTACTTGCAGAAGTCT
CTTTACTGAATGTATACTTCTGCTCTCTGGAAAGACATGATCGAATTGTACCACATGCCA
ATCGGTTGATGGTCTTCCATTCTTTATCATCAACCCCTTCTGGTTTTTCTTCCTCAATGGC
AATATCTAGACCCTGTTGAAAGAGGGCATCTAGAAGCTCACTTTGCCACATGCCGAAAT
GACCTGTTCCATCAAAAATTTCCACTACAAATCTCGTATTTGCAGTTCCAATCCTCGGC
AAAATGTACGAAGTGGAAGTTGTTGATATGGATGGTTTTTCTTCTGTCATTTTTGCCATA
GCTTCTACTTAATAGCCACCAAATGCAGAATACCCACAAGCTTTAGGAAATGTTTCTGA
TGTGTAAGATCAGACTAAGCTGCAAACACAGAGCATACTACAAAACCTTGGCTCTGAT
ACCAATTGTTGCGGAAGCGACGATAATATTAATAACAATATTATCAGAAATATTTAGATA
ATTATTATGCCAACAATTATACTAAGAAAATTCCCAGTTAAATTGGAGGGGTCACAATG
GTCCCGCTTAAAACCCAACAACTAGACAGAACTCACTTAGATATTTATAGCAATAATAA
CTAAATAAATATAACCAACATAAAGCTATTAAATAAAAGCAAACAAATAAGAAATAAAA
TACCAGAGTTTTAACGAGGTTCGGCCAATTTAGCTTACGTCCTCGGACACTACCAAATT
AATATTTCCTCTAGAAATATTACAAAGGAGAAGATTTTGGGATAATACAACCAAAGGGG
GAGGGGTTCTATATATAACAAACCAAACCCCCTCCATTTCTATCTTTTCCTAATGTGGGA
TATTGCCAATATTCAACAAAGT

Letters in blue: target site duplication
Letters in red: long terminal repeat
Letters with yellow background: gag-polypeptide of LTR *copia*-type
Letters with green background: GAG-pre-integrase domain
Letters with pink background: integrase core domain
