# Supplementary material for: A Retrotransposon Insertion in GhMML3_D12 Is Likely Responsible for the Lintless Locus li3 of Tetraploid Cotton
Source: Front Plant Sci. 2020 Nov 26;11:593679. doi: 10.3389/fpls.2020.593679 (PMC7725795; doi:10.3389/fpls.2020.593679)
Supplement: Supplementary file 1 [file Data_Sheet_1.zip › Fig S1-Fig S8 and Table S1-S11/Fig S3.pdf]

Fig. S3. Alignment of the coding sequences of *MML4_A12* from normal lines and mutants. Normal lines: TM-1, JZ-Wild; fuzzless mutants: n2, 11452GZ; Gb lines: Xinhai21, 3-79; fiberless mutants: 081925 fl, JZ-fl, Xu142 fl, SL1-7-1. Ga lines: Shixiya1.

```
TM-1(this study)                    1   ATGCGGCCACCGTCTCCGAACAGAAAGAAAGAGGTGAGATTGAAGAGAGGGCCATGGACAGCTGAAGAAG
JZ-Wild                             1   ATGCGGCCACCGTCTCCGAACAGAAAGAAAGAGGTGAGATTGAAGAGAGGGCCATGGACAGCTGAAGAAG
TM-1(CotAD_16205 BGI)               1   ATGCGGCCACCGTCTCCGAACAGAAAGAAAGAGGTGAGATTGAAGAGAGGGCCATGGACAGCTGAAGAAG
TM-1(Gh_A12G1504 NAU)               1   ATGCGGCCACCGTCTCCGAACAGAAAGAAAGAGGTGAGATTGAAGAGAGGGCCATGGACAGCTGAAGAAG
n2                                  1   ATGCGGCCACCGTCTCCGAACAGAAAGAAAGAGGTGAGATTGAAGAGAGGGCCATGGACAGCTGAAGAAG
081925 fl                           1   ATGCGGCCACCGTCTCCGAACAGAAAGAAAGAGGTGAGATTGAAGAGAGGGCCATGGACAGCTGAAGAAG
JZ-fl                               1   ATGCGGCCACCGTCTCCGAACAGAAAGAAAGAGGTGAGATTGAAGAGAGGGCCATGGACAGCTGAAGAAG
Xu142 fl                            1   ATGCGGCCACCGTCTCCGAACAGAAAGAAAGAGGTGAGATTGAAGAGAGGGCCATGGACAGCTGAAGAAG
11452GZ                             1   ATGCGGCCACCGTCTCCGAACAGAAAGAAAGAGGTGAGATTGAAGAGAGGGCCATGGACAGCTGAAGAAG
SL1-7-1                             1   ATGCGGCCACCGTCTCCGAACAGAAAGAAAGAGGTGAGATTGAAGAGAGGGCCATGGACAGCTGAAGAAG
Xinhai21(GOBAR_AA37559 NAU)         1   ATGCGGCCACCGTCTCCGAACAGAAAGAAAGAGGTGAGATTGAAGAGAGGGCCATGGACAGCTGAAGAAG
3-79(Gbscaffold10496.11.0 HZAU      1   ATGCGGCCACCGTCTCCGAACAGAAAGAAAGAGGTGAGATTGAAGAGAGGGCCATGGACAGCTGAAGAAG
Shixiya1(Ga12G1198.1 CRI)           1   ATGCGGCCACCGTCTCCGAACAGAAAGAAAGAGGTGAGATTGAAGAGAGGGCCATGGACAGCTGAAGAAG

TM-1(this study)                    71  ACAAATTACTGACGGCTTACATTCAAAAACATGGCTATGGCAGCAGGGGTTCCTTGCCTCACAAAGCTGG
JZ-Wild                             71  ACAAATTACTGACGGCTTACATTCAAAAACATGGCTATGGCAGCAGGGGTTCCTTGCCTCACAAAGCTGG
TM-1(CotAD_16205 BGI)               71  ACAAATTACTGACGGCTTACATTCAAAAACATGGCTATGGCAGCAGGGGTTCCTTGCCTCACAAAGCTGG
TM-1(Gh_A12G1504 NAU)               71  ACAAATTACTGACGGCTTACATTCAAAAACATGGCTATGGTAGCTGGGGTTCCTTGCCTCACAAAGCTGG
n2                                  71  ACAAATTACTGACGGCTTACATTCAAAAACATGGCTATGGCAGCAGGGGTTCCTTGCCTCACAAAGCTGG
081925 fl                           71  ACAAATTACTGACGGCTTACATTCAAAAACATGGCTATGGCAGCAGGGGTTCCTTGCCTCACAAAGCTGG
JZ-fl                               71  ACAAATTACTGACGGCTTACATTCAAAAACATGGCTATGGCAGCAGGGGTTCCTTGCCTCACAAAGCTGG
Xu142 fl                            71  ACAAATTACTGACGGCTTACATTCAAAAACATGGCTATGGCAGCAGGGGTTCCTTGCCTCACAAAGCTGG
11452GZ                             71  ACAAATTACTGACGGCTTACATTCAAAAACATGGCTATGGCAGCAGGGGTTCCTTGCCTCACAAAGCTGG
SL1-7-1                             71  ACAAATTACTGACGGCTTACATTCAAAAACATGGCTATGGCAGCAGGGGTTCCTTGCCTCACAAAGCTGG
Xinhai21(GOBAR_AA37559 NAU)         71  ACAAATTACTGACGGCTTACATTCAAAAACATGGCTATGGCAGCAGGGGTTCCTTGCCTCACAAAGCTGG
3-79(Gbscaffold10496.11.0 HZAU      71  ACAAATTACTGACGGCTTACATTCAAAAACATGGCTATGGCAGCAGGGGTTCCTTGCCTCACAAAGCTGG
Shixiya1(Ga12G1198.1 CRI)           71  ACAAATTACTGACGGCTTACATTCAAAAACATGGCTATGGCAGCTGGGGTTCCTTGCCTCACAAAGCTGG

TM-1(this study)                    141 ACTTGAACGATGTGGAAAGAGCTGCCGACTGAGATGGATTAACTACTTAAGACCTGATATCAAAAGAGGA
JZ-Wild                             141 ACTTGAACGATGTGGAAAGAGCTGCCGACTGAGATGGATTAACTACTTAAGACCTGATATCAAAAGAGGA
TM-1(CotAD_16205 BGI)               141 ACTTGAACGATGTGGAAAGAGCTGCCGACTGAGATGGATTAACTACTTAAGACCTGATATCAAAAGAGGA
TM-1(Gh_A12G1504 NAU)               141 ACTTGAACGATGTGGAAAGAGCTGCCGACTGAGATGGATTAACTACTTAAGACCTGATATCAAAAGAGGA
n2                                  141 ACTTGAACGATGTGGAAAGAGCTGCCGACTGAGATGGATTAACTACTTAAGACCTGATATCAAAAGAGGA
081925 fl                           141 ACTTGAACGATGTGGAAAGAGCTGCCGACTGAGATGGATTAACTACTTAAGACCTGATATCAAAAGAGGA
JZ-fl                               141 ACTTGAACGATGTGGAAAGAGCTGCCGACTGAGATGGATTAACTACTTAAGACCTGATATCAAAAGAGGA
Xu142 fl                            141 ACTTGAACGATGTGGAAAGAGCTGCCGACTGAGATGGATTAACTACTTAAGACCTGATATCAAAAGAGGA
11452GZ                             141 ACTTGAACGATGTGGAAAGAGCTGCCGACTGAGATGGATTAACTACTTAAGACCTGATATCAAAAGAGGA
SL1-7-1                             141 ACTTGAACGATGTGGAAAGAGCTGCCGACTGAGATGGATTAACTACTTAAGACCTGATATCAAAAGAGGA
Xinhai21(GOBAR_AA37559 NAU)         141 ACTTGAACGATGTGGAAAGAGCTGCCGACTGAGATGGATTAACTACTTAAGACCTGATATCAAAAGAGGA
3-79(Gbscaffold10496.11.0 HZAU      141 ACTTGAACGATGTGGAAAGAGCTGCCGACTGAGATGGATTAACTACTTAAGACCTGATATCAAAAGAGGA
Shixiya1(Ga12G1198.1 CRI)           141 ACTTGAACGATGTGGAAAGAGCTGCCGACTGAGATGGATTAACTACTTAAGACCTGATATCAAAAGAGGA

TM-1(this study)                    211 AAGTTTAGTTTAGAGGAAGAACAGACCATCATTCAACTCCATGCCTTTCTTGGAAACAGGTGGTCGGCAA
JZ-Wild                             211 AAGTTTAGTTTAGAGGAAGAACAGACCATCATTCAACTCCATGCCTTTCTTGGAAACAGGTGGTCGGCAA
TM-1(CotAD_16205 BGI)               211 AAGTTTAGTTTAGAGGAAGAACAGACCATCATTCAACTCCATGCCTTTCTTGGAAACAGGTGGTCGGCAA
TM-1(Gh_A12G1504 NAU)               211 AAGTTTAGTTTAGAGGAAGAACAGACCATCATTCAACTCCATGCCTTTCTTGGAAACAGGTGGTCGGCAA
n2                                  211 AAGTTTAGTTTAGAGGAAGAACAGACCATCATTCAACTCCATGCCTTTCTTGGAAACAGGTGGTCGGCAA
081925 fl                           211 AAGTTTAGTTTAGAGGAAGAACAGACCATCATTCAACTCCATGCCTTTCTTGGAAACAGGTGGTCGGCAA
JZ-fl                               211 AAGTTTAGTTTAGAGGAAGAACAGACCATCATTCAACTCCATGCCTTTCTTGGAAACAGGTGGTCGGCAA
```

```
Xu142 fl                              211 AAGTTTAGTTTAGAGGAAGAACAGACCATCATTCAACTCCATGCCTTTCTTGGAAACAGGTGGTCGGCAA
11452GZ                               211 AAGTTTAGTTTAGAGGAAGAACAGACCATCATTCAACTCCATGCCTTTCTTGGAAACAGGTGGTCGGCAA
SL1-7-1                               211 AAGTTTAGTTTAGAGGAAGAACAGACCATCATTCAACTCCATGCCTTTCTTGGAAACAGGTGGTCGGCAA
Xinhai21(GOBAR_AA37559 NAU)           211 AAGTTTAGTTTAGAGGAAGAACAGACCATCATTCAACTCCATGCCTTTCTTGGAAACAGGTGGTCGGCAA
3-79(Gbscaffold10496.11.0 HZAU        211 AAGTTTAGTTTAGAGGAAGAACAGACCATCATTCAACTCCATGCCTTTCTTGGAAACAGGTGGTCGGCAA
Shixiya1(Ga12G1198.1 CRI)             211 AAGTTTAGTTTAGAGGAAGAACAGACCATCATTCAACTCCATGCCTTTCTTGGAAACAGGTGGTCGGCAA

TM-1(this study)                      281 TAGCGGCACGCTTGCCTAAGAGAACAGACAATGAGATCAAGAATCACTGGAACACACATCTAAAGAAAAG
JZ-Wild                               281 TAGCGGCACGCTTGCCTAAGAGAACAGACAATGAGATCAAGAATCACTGGAACACACATCTAAAGAAAAG
TM-1(CotAD_16205 BGI)                 281 TAGCGGCACGCTTGCCTAAGAGAACAGACAATGAGATCAAGAATCACTGGAACACACATCTAAAGAAAAG
TM-1(Gh_A12G1504 NAU)                 281 TAGCGGCACGCTTGCCTAAGAGAACAGACAATGAGATCAAGAATCACTGGAACACACATCTAAAGAAAAG
n2                                    281 TAGCGGCACACTTGCCTAAGAGAACAGACAATGAGATCAAGAATCACTGGAACACACATCTAAAGAAAAG
081925 fl                             281 TAGCGGCACACTTGCCTAAGAGAACAGACAATGAGATCAAGAATCACTGGAACACACATCTAAAGAAAAG
JZ-fl                                 281 TAGCGGCACACTTGCCTAAGAGAACAGACAATGAGATCAAGAATCACTGGAACACACATCTAAAGAAAAG
Xu142 fl                              281 TAGCGGCACACTTGCCTAAGAGAACAGACAATGAGATCAAGAATCACTGGAACACACATCTAAAGAAAAG
11452GZ                               281 TAGCGGCACACTTGCCTAAGAGAACAGACAATGAGATCAAGAATCACTGGAACACACATCTAAAGAAAAG
SL1-7-1                               281 TAGCGGCACACTTGCCTAAGAGAACAGACAATGAGATCAAGAATCACTGGAACACACATCTAAAGAAAAG
Xinhai21(GOBAR_AA37559 NAU)           281 TAGCGGCACACTTGCCTAAGAGAACAGACAATGAGATCAAGAATCACTGGAACACACATCTAAAGAAAAG
3-79(Gbscaffold10496.11.0 HZAU        281 TAGCGGCACACTTGCCTAAGAGAACAGACAATGAGATCAAGAATCACTGGAACACACATCTAAAGAAAAG
Shixiya1(Ga12G1198.1 CRI)             281 TAGCGGCACACTTGCCTAAGAGAACAGACAATGAGATCAAGAATCACTGGAACACACATCTAAAGAAAAG

TM-1(this study)                      351 GCTAATCAAAATGGGTATTGATCCCATGACTCACAAGCCCTCAACCACCCCATCACCCAAAAATGGTTCA
JZ-Wild                               351 GCTAATCAAAATGGGTATTGATCCCATGACTCACAAGCCCTCAACCACCCCATCACCCAAAAATGGTTCA
TM-1(CotAD_16205 BGI)                 351 GCTAATCAAAATGGGTATTGATCCCATGACTCACAAGCCCTCAACCACCCCATCACCCAAAAATGGTTCA
TM-1(Gh_A12G1504 NAU)                 351 GCTAATCAAAATGGGTATTGATCCCATGACTCACAAGCCCTCAACCACCCCATCACCCAAAAATGGTTCA
n2                                    351 GCTAATCAAAATGGGTATTGATCCCATGACTCACAAGCCCTCAACCACCCCATCACCCAAAAATGGTTCA
081925 fl                             351 GCTAATCAAAATGGGTATTGATCCCATGACTCACAAGCCCTCAACCACCCCATCACCCAAAAATGGTTCA
JZ-fl                                 351 GCTAATCAAAATGGGTATTGATCCCATGACTCACAAGCCCTCAACCACCCCATCACCCAAAAATGGTTCA
Xu142 fl                              351 GCTAATCAAAATGGGTATTGATCCCATGACTCACAAGCCCTCAACCACCCCATCACCCAAAAATGGTTCA
11452GZ                               351 GCTAATCAAAATGGGTATTGATCCCATGACTCACAAGCCCTCAACCACCCCATCACCCAAAAATGGTTCA
SL1-7-1                               351 GCTAATCAAAATGGGTATTGATCCCATGACTCACAAGCCCTCAACCACCCCATCACCCAAAAATGGTTCA
Xinhai21(GOBAR_AA37559 NAU)           351 GCTAATCAAAATGGGTATTGATCCCATGACTCACAAGCCCTCAACCACCCCATCACCCAAAAATGGTTCA
3-79(Gbscaffold10496.11.0 HZAU        351 GCTAATCAAAATGGGTATTGATCCCATGACTCACAAGCCCTCAACCACCCCATCACCCAAAAATGGTTCA
Shixiya1(Ga12G1198.1 CRI)             351 GCTAATCAAAATGGGTATTGATCCCATGACTCACAAGCCCTCAACCTCCCCATCACCCAAAAATGGTTCA

TM-1(this study)                      421 AATTTAAGCCACATGGCCCAGTGGGAGAGTGCACGTCTACAGGCTGAAGCCAGGTTGGTCCGTGAGTCAA
JZ-Wild                               421 AATTTAAGCCACATGGCCCAGTGGGAGAGTGCACGTCTACAGGCTGAAGCCAGGTTGGTCCGTGAGTCAA
TM-1(CotAD_16205 BGI)                 421 AATTTAAGCCACATGGCCCAGTGGGAGAGTGCACGTCTACAGGCTGAAGCCAGGTTGGTCCGTGAGTCAA
TM-1(Gh_A12G1504 NAU)                 421 AATTTAAGCCACATGGCCCAGTGGGAGAGTGCACGTCTACAGGCTGAAGCCAGGTTGGTCCGTGAGTCAA
n2                                    421 AATTTAAGCCACATGGCCCAGTGGGAGAGTGCACGTCTACAGGCTGAAGCCAGGTTGGTCCGTGAGTCAA
081925 fl                             421 AATTTAAGCCACATGGCCCAGTGGGAGAGTGCACGTCTACAGGCTGAAGCCAGGTTGGTCCGTGAGTCAA
JZ-fl                                 421 AATTTAAGCCACATGGCCCAGTGGGAGAGTGCACGTCTACAGGCTGAAGCCAGGTTGGTCCGTGAGTCAA
Xu142 fl                              421 AATTTAAGCCACATGGCCCAGTGGGAGAGTGCACGTCTACAGGCTGAAGCCAGGTTGGTCCGTGAGTCAA
11452GZ                               421 AATTTAAGCCACATGGCCCAGTGGGAGAGTGCACGTCTACAGGCTGAAGCCAGGTTGGTCCGTGAGTCAA
SL1-7-1                               421 AATTTAAGCCACATGGCCCAGTGGGAGAGTGCACGTCTACAGGCTGAAGCCAGGTTGGTCCGTGAGTCAA
Xinhai21(GOBAR_AA37559 NAU)           421 AATTTAAGCCACATGGCCCAGTGGGAGAGTGCACGTCTACAGGCTGAAGCCAGGTTGGTCCGTGAGTCAA
3-79(Gbscaffold10496.11.0 HZAU        421 AATTTAAGCCACATGGCCCAGTGGGAGAGTGCACGTCTACAGGCTGAAGCCAGGTTGGTCCGTGAGTCAA
Shixiya1(Ga12G1198.1 CRI)             421 AATTTAAGCCACATGGCCCAGTGGGAGAGTGCACGTCTACAGGCTGAAGCCAGGTTGGTCCGTGAGTCAA

TM-1(this study)                      491 AACAGGTTGTCCCAAATCTTACCACCCGCCCCACTAGGAGGAGTCAACTCACCAGAAGCAGTCCCAGGTG
JZ-Wild                               491 AACAGGTTGTCCCAAATCTTACCACCCGCCCCACTAGGAGGAGTCAACTCACCAGAAGCAGTCCCAGGTG
TM-1(CotAD_16205 BGI)                 491 AACAGGTTGTCCCAAATCTTACCACCCGCCCCACTAGGAGGAGTCAACTCACCAGAAGCAGTCCCAGGTG
TM-1(Gh_A12G1504 NAU)                 491 AACAGGTTGTCCCAAATCTTACCACCCGCCCCACTAGGAGGAGTCAACTCACCAGAAGCAGTCCCAGGTG
n2                                    491 AACAGGTTGTCCCAAATCTTACCACCTGCCCCACTAGGAGGAGTCAACTCACCAGAAGCAGTCCCAGGTG
081925 fl                             491 AACAGGTTGTCCCAAATCTTACCACCTGCCCCACTAGGAGGAGTCAACTCACCAGAAGCAGTCCCAGGTG
JZ-fl                                 491 AACAGGTTGTCCCAAATCTTACCACCTGCCCCACTAGGAGGAGTCAACTCACCAGAAGCAGTCCCAGGTG
Xu142 fl                              491 AACAGGTTGTCCCAAATCTTACCACCTGCCCCACTAGGAGGAGTCAACTCACCAGAAGCAGTCCCAGGTG
11452GZ                               491 AACAGGTTGTCCCAAATCTTACCACCTGCCCCACTAGGAGGAGTCAACTCACCAGAAGCAGTCCCAGGTG
```

```
SL1-7-1                              491 AACAGGTTGTCCCAAATCTTACCACCTGCCCCACTAGGAGGAGTCAACTCACCAGAAGCAGTCCCAGGTG
Xinhai21(GOBAR_AA37559 NAU)          491 AACAGGTTGTCCCAAATCTTACCACCCGCCCCACTAGGAGGAGTCAACTCACCAGAAGCAGTCCCAGATG
3-79(Gbscaffold10496.11.0 HZAU       491 AACAGGTTGTCCCAAATCTTACCACCCGCCCCACTAGGAGGAGTCAACTCACCAGAAGCAGTCCCAGATG
Shixiya1(Ga12G1198.1 CRI)            491 AACAGGTTGTCCCAAATCTTACCACCCGCCCCACTAGGAGGAGTCAACTCACCAGAAGCAGTCCCAGGTG

TM-1(this study)                     561 CCTTGACATACTCAAAGCCTGGCAAGGTGTAGTTGCCGGGATGTTCGTTTTCTCCACCCAGGATCCCAGG
JZ-Wild                              561 CCTTGACATACTCAAAGCCTGGCAAGGTGTAGTTGCCGGGATGTTCGTTTTCTCCACCCAGGATCCCAGG
TM-1(CotAD_16205 BGI)                561 CCTTGACATACTCAAAGCCTGGCAAGGTGTAGTTGCCGGGATGTTCGTTTTCTCCACCCAGGATCCCAGG
TM-1(Gh_A12G1504 NAU)                561 CCTTGACATACTCAAAGCCTGGCAAGGTGTAGTTGCCGGGATGTTCGTTTTCTCCACCCAGGATCCCAGG
n2                                   561 CCTTGACATACTCAAAGCCTGGCAAGGTGTAGTTGCCGGGATGTTCGTTTTCTCCACCCAGGATCCCAGG
081925 fl                            561 CCTTGACATACTCAAAGCCTGGCAAGGTGTAGTTGCCGGGATGTTCGTTTTCTCCACCCAGGATCCCAGG
JZ-fl                                561 CCTTGACATACTCAAAGCCTGGCAAGGTGTAGTTGCCGGGATGTTCGTTTTCTCCACCCAGGATCCCAGG
Xu142 fl                             561 CCTTGACATACTCAAAGCCTGGCAAGGTGTAGTTGCCGGGATGTTCGTTTTCTCCACCCAGGATCCCAGG
11452GZ                              561 CCTTGACATACTCAAAGCCTGGCAAGGTGTAGTTGCCGGGATGTTCGTTTTCTCCACCCAGGATCCCAGG
SL1-7-1                              561 CCTTGACATACTCAAAGCCTGGCAAGGTGTAGTTGCCGGGATGTTCGTTTTCTCCACCCAGGATCCCAGG
Xinhai21(GOBAR_AA37559 NAU)          561 CCTTGACGTACTCAAAGCCTGGCAAGGTGTAGTTGCCGGGATGTTCGTTTTCTCCACCCAGGATCCCAGG
3-79(Gbscaffold10496.11.0 HZAU       561 CCTTGACGTACTCAAAGCCTGGCAAGGTGTAGTTGCCGGGATGTTCGTTTTCTCCACCCAGGATCCCAGG
Shixiya1(Ga12G1198.1 CRI)            561 CCTTGACGTACTCAAAGCCTGGCAAGGTGTAGTTGCCGGGATGTTTGTTTTCTCCACCCAGGATCCCAGG

TM-1(this study)                     631 TCCCTAACAACCTCAACTCTTCGTTTCCCTTCAGCTGGATGGGGAGAAGCTGAGGAATGGCGGGGCCAGG
JZ-Wild                              631 TCCCTAACAACCTCAACTCTTCGTTTCCCTTCAGCTGGATGGGGAGAAGCTGAGGAATGGCGGGGCCAGG
TM-1(CotAD_16205 BGI)                631 TCCCTAACAACCTCAACTCTTCGTTTCCCTTCAGCTGGATGGGGAGAAGCTGAGGAATGGCGGGGCCAGG
TM-1(Gh_A12G1504 NAU)                631 TCCCTAACAACCTCAACTCTTCGTTTCCCTTCAGCTGGATGGGGAGAAGCTGAGGAATGGCGGGGCCAGG
n2                                   631 TCCCTAACAACCTCAACTCTTCGTTTCCCTTCAGCTGGATGGGGAGAAGCTGAGGAATGGCGGGGCCAGG
081925 fl                            631 TCCCTAACAACCTCAACTCTTCGTTTCCCTTCAGCTGGATGGGGAGAAGCTGAGGAATGGCGGGGCCAGG
JZ-fl                                631 TCCCTAACAACCTCAACTCTTCGTTTCCCTTCAGCTGGATGGGGAGAAGCTGAGGAATGGCGGGGCCAGG
Xu142 fl                             631 TCCCTAACAACCTCAACTCTTCGTTTCCCTTCAGCTGGATGGGGAGAAGCTGAGGAATGGCGGGGCCAGG
11452GZ                              631 TCCCTAACAACCTCAACTCTTCGTTTCCCTTCAGCTGGATGGGGAGAAGCTGAGGAATGGCGGGGCCAGG
SL1-7-1                              631 TCCCTAACAACCTCAACTCTTCGTTTCCCTTCAGCTGGATGGGGAGAAGCTGAGGAATGGCGGGGCCAGG
Xinhai21(GOBAR_AA37559 NAU)          631 TCCCTAACAACCTCAACTCTTCGTTTCCCTTCAGCTGGATGGGGAGAAGCTGAGGAATGGCGGGGCCAGG
3-79(Gbscaffold10496.11.0 HZAU       631 TCCCTAACAACCTCAACTCTTCGTTTCCCTTCAGCTGGATGGGGAGAAGCTGAGGAATGGCGGGGCCAGG
Shixiya1(Ga12G1198.1 CRI)            631 TCCCTAACAACCTCAACTCTTCGTTTCCCTTCAGCTGGATGGGGAGAAGCTGAGGAATGGCGGGGCCAGG

TM-1(this study)                     701 GAATGAAGGGTTCCAGCGATGCTGATGATGCATGGTTTGAGGAGGACTCAGTCATACTACACAGTCTACC
JZ-Wild                              701 GAATGAAGGGTTCCAGCGATGCTGATGATGCATGGTTTGAGGAGGACTCAGTCATACTACACAGTCTACC
TM-1(CotAD_16205 BGI)                701 GAATGAAGGGTTCCAGCGATGCTGATGATGCATGGTTTGAGGAGGACTCAGTCATACTACACAGTCTACC
TM-1(Gh_A12G1504 NAU)                701 GAATGAAGGGTTCCAGCGATGCTGATGATGCATGGTTTGAGGAGGACTCAGTCATACTACACAGTCTACC
n2                                   701 GAATGAAGGGTTCCAGCGATGCTGATGATGCATGGTTTGAGGAGGACTCAGTCATACTACACAGTCTACC
081925 fl                            701 GAATGAAGGGTTCCAGCGATGCTGATGATGCATGGTTTGAGGAGGACTCAGTCATACTACACAGTCTACC
JZ-fl                                701 GAATGAAGGGTTCCAGCGATGCTGATGATGCATGGTTTGAGGAGGACTCAGTCATACTACACAGTCTACC
Xu142 fl                             701 GAATGAAGGGTTCCAGCGATGCTGATGATGCATGGTTTGAGGAGGACTCAGTCATACTACACAGTCTACC
11452GZ                              701 GAATGAAGGGTTCCAGCGATGCTGATGATGCATGGTTTGAGGAGGACTCAGTCATACTACACAGTCTACC
SL1-7-1                              701 GAATGAAGGGTTCCAGCGATGCTGATGATGCATGGTTTGAGGAGGACTCAGTCATACTACACAGTCTACC
Xinhai21(GOBAR_AA37559 NAU)          701 GAATGAAGGGTTCCAGCGATGCTGATGATGCATGGTTTGAGGAGGACTCAGTCATACTACACAGTCTACC
3-79(Gbscaffold10496.11.0 HZAU       701 GAATGAAGGGTTCCAGCGATGCTGATGATGCATGGTTTGAGGAGGACTCAGTCATACTACACAGTCTACC
Shixiya1(Ga12G1198.1 CRI)            701 GAATGAAGGGTTCCAGCGATGCTGATGATGCATGGTTTGAGGAGGACTCAGTCATACTACACAGTCTACC

TM-1(this study)                     771 TATTGAAAATATAATGGAAGGTTTGTCGGATGCTTTTATTTTGAATTCATGGATGGGTGTCGACAAATCA
JZ-Wild                              771 TATTGAAAATATAATGGAAGGTTTGTCGGATGCTTTTATTTTGAATTCATGGATGGGTGTCGACAAATCA
TM-1(CotAD_16205 BGI)                771 TATTGAAAATATAATGGAAGGTTTGTCGGATGCTTTTATTTTGAATTCATGGATGGGTGTCGACAAATCA
TM-1(Gh_A12G1504 NAU)                771 TATTGAAAATATAATGGAAGGTTTGTCGGATGCTTTTATTTTGAATTCATGGATGGGTGTCGACAAATCA
n2                                   771 TATTGAAAATATAATGGAAGGTTTGTCGGATGCTTTTATTTTGAATTCATGGATGGGTGTCGACAAATCA
081925 fl                            771 TATTGAAAATATAATGGAAGGTTTGTCGGATGCTTTTATTTTGAATTCATGGATGGGTGTCGACAAATCA
JZ-fl                                771 TATTGAAAATATAATGGAAGGTTTGTCGGATGCTTTTATTTTGAATTCATGGATGGGTGTCGACAAATCA
Xu142 fl                             771 TATTGAAAATATAATGGAAGGTTTGTCGGATGCTTTTATTTTGAATTCATGGATGGGTGTCGACAAATCA
11452GZ                              771 TATTGAAAATATAATGGAAGGTTTGTCGGATGCTTTTATTTTGAATTCATGGATGGGTGTCGACAAATCA
SL1-7-1                              771 TATTGAAAATATAATGGAAGGTTTGTCGGATGCTTTTATTTTGAATTCATGGATGGGTGTCGACAAATCA
Xinhai21(GOBAR_AA37559 NAU)          771 TATTGAAAATATAATGGAAGGTTTATCGGATGCTTTTATTTTGAATTCATGGATGGGTGTCGACAAATCA
```

```
3-79(Gbscaffold10496.11.0 HZAU    771 TATTGAAAATATAATGGAAGGTTTATCGGATGCTTTTATTTTGAATTCATGGATGGGTGTCGACAAATCA
Shixiya1(Ga12G1198.1 CRI)         771 TATTGAAAATATAATGGAAGGTTTGTCGGATGCTTTTATTTTGAATTCATGGATGGGTGTCGACGAATCA

TM-1(this study)                   841 ACAGATGAAAACATTGTAAAGGAGAATGGTCAATTGCTGGGATAGCGTACTCAACTTGTTGAGTTCTACA
JZ-Wild                            841 ACAGATGAAAACATTGTAAAGGAGAATGGTCAATTGCTGGGATAGCGTACTCAACTTGTTGAGTTCTACA
TM-1(CotAD_16205 BGI)              841 ACAGATGAAAACATTGTAAAGGAGAATGGTCAATTGCTGGGATAGCGTACTCAACTTGTTGAGTTCTACA
TM-1(Gh_A12G1504 NAU)              841 ACAGATGAAAACATTGTAAAGGAGAATGGTCAATTGCTGGGATAGCGTACTCAACTTGTTGAGTTCTACA
n2                                 841 ACAGATGAAAACATTGTAAAGGAGAATGGT-AATTGCTGGGATAGCGTACTCAACTTGTTGAGTTCTACA
081925 fl                          841 ACAGATGAAAACATTGTAAAGGAGAATGGT-AATTGCTGGGATAGCGTACTCAACTTGTTGAGTTCTACA
JZ-fl                              841 ACAGATGAAAACATTGTAAAGGAGAATGGT-AATTGCTGGGATAGCGTACTCAACTTGTTGAGTTCTACA
Xu142 fl                           841 ACAGATGAAAACATTGTAAAGGAGAATGGT-AATTGCTGGGATAGCGTACTCAACTTGTTGAGTTCTACA
11452GZ                            841 ACAGATGAAAACATTGTAAAGGAGAATGGT-AATTGCTGGGATAGCGTACTCAACTTGTTGAGTTCTACA
SL1-7-1                            841 ACAGATGAAAACACTGTAATGGAGAATGGT-AATTGCTGGGATAGCGTACTCAACTTCTTGAATTCTTCA
Xinhai21(GOBAR_AA37559 NAU)        841 ACAGATGAAAACACTGTAAAGGAGAATGGT-AATTGCTGGGATAGCGTACTCAACTTGTTGAGTTCTACA
3-79(Gbscaffold10496.11.0 HZAU    841 ACAGATGAAAACACTGTAAAGGAGAATGGT-AATTGCTGGGATAGCGTACTCAACTTGTTGAGTTCTACA
Shixiya1(Ga12G1198.1 CRI)         841 ACAGATGAAAACACTGTAAAGGAGAATGGT-AATTGCTGGGATAGCGTACTCAACTTGTTGAGTTCTACA

TM-1(this study)                   911 CCATGTGGTTCGCCTGTGTTGGGATAA
JZ-Wild                            911 CCATGTGGTTCGCCTGTGTTGGGATAA
TM-1(CotAD_16205 BGI)              911 CCATGTGGTTCGCCTGTGTTGGGATAA
TM-1(Gh_A12G1504 NAU)              911 CCATGTGGTTCGCCTGTGTTGGGATAA
n2                                 910 CCATGTGGTTCGCCTGTGTTGGGATAA
081925 fl                          910 CCATGTGGTTCGCCTGTGTTGGGATAA
JZ-fl                              910 CCATGTGGTTCGCCTGTGTTGGGATAA
Xu142 fl                           910 CCATGTGGTTCGCCTGTGTTGGGATAA
11452GZ                            910 CCATGTGGTTCGCCTGTGTTGGGATAA
SL1-7-1                            910 CCATGTTGTTCGCCTGTGTTGGGATAA
Xinhai21(GOBAR_AA37559 NAU)        910 CCATGTGGTTCGCCTGTGTTGGGATAA
3-79(Gbscaffold10496.11.0 HZAU    910 CCATGTGGTTCGCCTGTGTTGGGATAA
Shixiya1(Ga12G1198.1 CRI)         910 CCATGTGGTTCGCCTGTGTTGGGATAA
```
