# Supplementary material for: A Retrotransposon Insertion in GhMML3_D12 Is Likely Responsible for the Lintless Locus li3 of Tetraploid Cotton
Source: Front Plant Sci. 2020 Nov 26;11:593679. doi: 10.3389/fpls.2020.593679 (PMC7725795; doi:10.3389/fpls.2020.593679)
Supplement: Supplementary file 1 [file Data_Sheet_1.zip › Fig S1-Fig S8 and Table S1-S11/Fig S4.pdf]

Fig. S4. Alignment of the coding sequences of *MML3_A12* from normal lines and mutants. Normal lines: TM-1; fuzzless mutants: T586, GZNn2-1, 11452GZ, TaoGZ, gznn1-1, n2; Gb lines: 3-79, Hai7124, 9078N, Xinhai18, Xinhai21, Jizha45; fiberless mutants: 081925 fl, MD17, Xu142 fl, SL1-7-1; Ga lines: Shixiya1.

```
TM-1(Gh_A12G1503 NAU)          1   ATGCAGCAGTCTCCATGTAGCGACAAGGTGGGGTTGAAGAAAGGGCCATGGACTCCAGAAGAAGACCAAA
TM-1(CotAD_16204 BGI)          1   ATGCAGCAGTCTCCATGTAGCGACAAGGTGGGGTTGAAGAAAGGGCCATGGACTCCAGAAGAAGACCAAA
TM-1(this study)               1   ATGCAGCAGTCTCCATGTAGCGACAAGGTGGGGTTGAAGAAAGGGCCATGGACTCCAGAAGAAGACCAAA
SL1-7-1                        1   ATGCAGCAGTCTCCATGTAGCGACAAGGTGGGGTTGAAGAAAGGGCCATGGACTCCAGAAGAAGACCAAA
MD17                           1   ATGCAGCAGTCTCCATGTAGCGACAAGGTGGGGTTGAAGAAAGGGCCATGGACTCCAGAAGAAGACCAAA
T586                           1   ATGCAGCAGTCTCCATGTAGCGACAAGGTGGGGTTGAAGAAAGGGCCATGGACTCCAGAAGAAGACCAAA
11452GZ                        1   ATGCAGCAGTCTCCATGTAGCGACAAGGTGGGGTTGAAGAAAGGGCCATGGACTCCAGAAGAAGACCAAA
GZNn2-1                        1   ATGCAGCAGTCTCCATGTAGCGACAAGGTGGGGTTGAAGAAAGGGCCATGGACTCCAGAAGAAGACCAAA
081925 fl                      1   ATGCAGCAGTCTCCATGTAGCGACAAGGTGGGGTTGAAGAAAGGGCCATGGACTCCAGAAGAAGACCAAA
gznn1-1                        1   ATGCAGCAGTCTCCATGTAGCGACAAGGTGGGGTTGAAGAAAGGGCCATGGACTCCAGAAGAAGACCAAA
TaoGZ                          1   ATGCAGCAGTCTCCATGTAGCGACAAGGTGGGGTTGAAGAAAGGGCCATGGACTCCAGAAGAAGACCAAA
n2                             1   ATGCAGCAGTCTCCATGTAGCGACAAGGTGGGGTTGAAGAAAGGGCCATGGACTCCAGAAGAAGACCAAA
N1                             1   ATGCAGCAGTCTCCATGTAGCGACAAGGTGGGGTTGAAGAAAGGGCCATGGACTCCAGAAGAAGACCAAA
JZ-fl                          1   ATGCAGCAGTCTCCATGTAGCGACAAGGTGGGGTTGAAGAAAGGGCCATGGACTCCAGAAGAAGACCAAA
XZ142 fl                       1   ATGCAGCAGTCTCCATGTAGCGACAAGGTGGGGTTGAAGAAAGGGCCATGGACTCCAGAAGAAGACCAAA
Shixiya1(Cotton_A_06163 BGI)   1   ATGCAGCAGTCTCCATGTAGCGACAAGGTGGGGTTGAAGAAAGGGCCATGGACTCCAGAAGAAGACCAAA
Shixiya1(Ga12G1199.1 CRI)      1   ATGCAGCAGTCTCCATGTAGCGACAAGGTGGGGTTGAAGAAAGGGCCATGGACTCCAGAAGAAGACCAAA
Xinhai21(GOBAR_AA13136 NAU)    1   ATGCAGCAGTCTCCATGTAGCGACAAGGTGGGGTTGAAGAAAGGGCCATGGACTCCAGAAGAAGACCAAA
Jizha45                        1   ATGCAGCAGTCTCCATGTAGCGACAAGGTGGGGTTGAAGAAAGGGCCATGGACTCCAGAAGAAGACCAAA
Xinhai18                       1   ATGCAGCAGTCTCCATGTAGCGACAAGGTGGGGTTGAAGAAAGGGCCATGGACTCCAGAAGAAGACCAAA
9078N                          1   ATGCAGCAGTCTCCATGTAGCGACAAGGTGGGGTTGAAGAAAGGGCCATGGACTCCAGAAGAAGACCAAA
3-79                           1   ATGCAGCAGTCTCCATGTAGCGACAAGGTGGGGTTGAAGAAAGGGCCATGGACTCCAGAAGAAGACCAAA
Hai7124                        1   ATGCAGCAGTCTCCATGTAGCGACAAGGTGGGGTTGAAGAAAGGGCCATGGACTCCAGAAGAAGACCAAA
3-79(Gbscaffold6320.11.0 HZAU) 1   ATGCAGCAGTCTCCATGTAGCGACAAGGTGGGGTTGAAGAAAGGGCCATGGACTCCAGAAGAAGACCAAA

TM-1(Gh_A12G1503 NAU)          71  AACTCTTGTCTTATATTCAAGAACACGGCGGTGGAAGCTGGCGAGGCTTGCCCGCAAAAGCTGGACTTCA
TM-1(CotAD_16204 BGI)          71  AACTCTTGTCTTATATTCAAGAACACGGCGGTGGAAGCTGGCGAGGCTTGCCCGCAAAAGCTGGACTTCA
TM-1(this study)               71  AACTCTTGTCTTATATTCAAGAACACGGCGGTGGAAGCTGGCGAGGCTTGCCCGCAAAAGCTGGACTTCA
SL1-7-1                        71  AACTCTTGTCTTATATTCAAGAACACGGCGGTGGAAGCTGGCGAGGCTTGCCCGCAAAAGCTGGACTTCA
MD17                           71  AACTCTTGTCTTATATTCAAGAACACGGCGGTGGAAGCTGGCGAGGCTTGCCCGCAAAAGCTGGACTTCA
T586                           71  AACTCTTGTCTTATATTCAAGAACACGGCGGTGGAAGCTGGCGAGGCTTGCCCGCAAAAGCTGGACTTCA
11452GZ                        71  AACTCTTGTCTTATATTCAAGAACACGGCGGTGGAAGCTGGCGAGGCTTGCCCGCAAAAGCTGGACTTCA
GZNn2-1                        71  AACTCTTGTCTTATATTCAAGAACACGGCGGTGGAAGCTGGCGAGGCTTGCCCGCAAAAGCTGGACTTCA
081925 fl                      71  AACTCTTGTCTTATATTCAAGAACACGGCGGTGGAAGCTGGCGAGGCTTGCCCGCAAAAGCTGGACTTCA
gznn1-1                        71  AACTCTTGTCTTATATTCAAGAACACGGCGGTGGAAGCTGGCGAGGCTTGCCCGCAAAAGCTGGACTTCA
TaoGZ                          71  AACTCTTGTCTTATATTCAAGAACACGGCGGTGGAAGCTGGCGAGGCTTGCCCGCAAAAGCTGGACTTCA
n2                             71  AACTCTTGTCTTATATTCAAGAACACGGCGGTGGAAGCTGGCGAGGCTTGCCCGCAAAAGCTGGACTTCA
N1                             71  AACTCTTGTCTTATATTCAAGAACACGGCGGTGGAAGCTGGCGAGGCTTGCCCGCAAAAGCTGGACTTCA
JZ-fl                          71  AACTCTTGTCTTATATTCAAGAACACGGCGGTGGAAGCTGGCGAGGCTTGCCCGCAAAAGCTGGACTTCA
XZ142 fl                       71  AACTCTTGTCTTATATTCAAGAACACGGCGGTGGAAGCTGGCGAGGCTTGCCCGCAAAAGCTGGACTTCA
Shixiya1(Cotton_A_06163 BGI)   71  AACTCTTGTCTTATATTCAAGAACACGGCGGTGGAAGCTGGCGAGGCTTGCCCGCAAAAGCTGGACTTCA
Shixiya1(Ga12G1199.1 CRI)      71  AACTCTTGTCTTATATTCAAGAACACGGCGGTGGAAGCTGGCGAGGCTTGCCCGCAAAAGCTGGACTTCA
Xinhai21(GOBAR_AA13136 NAU)    71  AACTCTCGTCTTATATTCAAGAACACGGCGGTGGAAGCTGGCGAGGCTTGCCCGCAAAAGCTGGACTTCA
Jizha45                        71  AACTCTCGTCTTATATTCAAGAACACGGCGGTGGAAGCTGGCGAGGCTTGCCCGCAAAAGCTGGACTTCA
Xinhai18                       71  AACTCTCGTCTTATATTCAAGAACACGGCGGTGGAAGCTGGCGAGGCTTGCCCGCAAAAGCTGGACTTCA
9078N                          71  AACTCTCGTCTTATATTCAAGAACACGGCGGTGGAAGCTGGCGAGGCTTGCCCGCAAAAGCTGGACTTCA
3-79                           71  AACTCTCGTCTTATATTCAAGAACACGGCGGTGGAAGCTGGCGAGGCTTGCCCGCAAAAGCTGGACTTCA
Hai7124                        71  AACTCTCGTCTTATATTCAAGAACACGGCGGTGGAAGCTGGCGAGGCTTGCCCGCAAAAGCTGGACTTCA
```

3-79(Gbscaffold6320.11.0 HZAU)	71	AACTCTCGTCTTATATTCAAGAACACGGCGGTGGAAGCTGGCGAGGCTTGCCCGCAAAAGCTGGACTTCA
TM-1(Gh_A12G1503 NAU)	141	AAGATGTGGCAAGAGTTGTAGACTTAGGTGGATTAACTACTTAAGACCAGATATCAAAAGAGGAAAGTTC
TM-1(CotAD_16204 BGI)	141	AAGATGTGGCAAGAGTTGTAGACTTAGGTGGATTAACTACTTAAGACCAGATATCAAAAGAGGAAAGTTC
TM-1(this study)	141	AAGATGTGGCAAGAGTTGTAGACTTAGGTGGATTAACTACTTAAGACCAGATATCAAAAGAGGAAAGTTC
SL1-7-1	141	AAGATGTGGCAAGAGTTGTAGACTTAGGTGGATTAACTACTTAAGACCAGATATCAAAAGAGGAAAGTTC
MD17	141	AAGATGTGGCAAGAGTTGTAGACTTAGGTGGATTAACTACTTAAGACCAGATATCAAAAGAGGAAAGTTC
T586	141	AAGATGTGGCAAGAGTTGTAGACTTAGGTGGATTAACTACTTAAGACCAGATATCAAAAGAGGAAAGTTC
11452GZ	141	AAGATGTGGCAAGAGTTGTAGACTTAGGTGGATTAACTACTTAAGACCAGATATCAAAAGAGGAAAGTTC
GZNn2-1	141	AAGATGTGGCAAGAGTTGTAGACTTAGGTGGATTAACTACTTAAGACCAGATATCAAAAGAGGAAAGTTC
081925 fl	141	AAGATGTGGCAAGAGTTGTAGACTTAGGTGGATTAACTACTTAAGACCAGATATCAAAAGAGGAAAGTTC
gznn1-1	141	AAGATGTGGCAAGAGTTGTAGACTTAGGTGGATTAACTACTTAAGACCAGATATCAAAAGAGGAAAGTTC
TaoGZ	141	AAGATGTGGCAAGAGTTGTAGACTTAGGTGGATTAACTACTTAAGACCAGATATCAAAAGAGGAAAGTTC
n2	141	AAGATGTGGCAAGAGTTGTAGACTTAGGTGGATTAACTACTTAAGACCAGATATCAAAAGAGGAAAGTTC
N1	141	AAGATGTGGCAAGAGTTGTAGACTTAGGTGGATTAACTACTTAAGACCAGATATCAAAAGAGGAAAGTTC
JZ-fl	141	AAGATGTGGCAAGAGTTGTAGACTTAGGTGGATTAACTACTTAAGACCAGATATCAAAAGAGGAAAGTTC
XZ142 fl	141	AAGATGTGGCAAGAGTTGTAGACTTAGGTGGATTAACTACTTAAGACCAGATATCAAAAGAGGAAAGTTC
Shixiya1(Cotton_A_06163 BGI)	141	AAGATGTGGCAAGAGTTGTAGACTTAGGTGGATTAACTACTTAAGACCAGATATCAAAAGAGGAAAGTTC
Shixiya1(Ga12G1199.1 CRI)	141	AAGATGTGGCAAGAGTTGTAGACTTAGGTGGATTAACTACTTAAGACCAGATATCAAAAGAGGAAAGTTC
Xinhai21(GOBAR_AA13136 NAU)	141	AAGATGTGGCAAGAGTTGTAGACTTAGGTGGATTAACTACTTAAGACCAGATATCAAAAGAGGAAAGTTC
Jizha45	141	AAGATGTGGCAAGAGTTGTAGACTTAGGTGGATTAACTACTTAAGACCAGATATCAAAAGAGGAAAGTTC
Xinhai18	141	AAGATGTGGCAAGAGTTGTAGACTTAGGTGGATTAACTACTTAAGACCAGATATCAAAAGAGGAAAGTTC
9078N	141	AAGATGTGGCAAGAGTTGTAGACTTAGGTGGATTAACTACTTAAGACCAGATATCAAAAGAGGAAAGTTC
3-79	141	AAGATGTGGCAAGAGTTGTAGACTTAGGTGGATTAACTACTTAAGACCAGATATCAAAAGAGGAAAGTTC
Hai7124	141	AAGATGTGGCAAGAGTTGTAGACTTAGGTGGATTAACTACTTAAGACCAGATATCAAAAGAGGAAAGTTC
3-79(Gbscaffold6320.11.0 HZAU)	141	AAGATGTGGCAAGAGTTGTAGACTTAGGTGGATTAACTACTTAAGACCAGATATCAAAAGAGGAAAGTTC
TM-1(Gh_A12G1503 NAU)	211	AGTTCGCAGGAAGAACGAACCATCATTCAACTCCACGCCCTTCTTGGAAACAGGTGGTCGGCTATTGCGG
TM-1(CotAD_16204 BGI)	211	AGTTCGCAGGAAGAACGAACCATCATTCAACTCCACGCCCTTCTTGGAAACAGGTGGTCGGCTATTGCGG
TM-1(this study)	211	AGTTCGCAGGAAGAACGAACCATCATTCAACTCCACGCCCTTCTTGGAAACAGGTGGTCGGCTATTGCGG
SL1-7-1	211	AGTTCGCAGGAAGAACGAACCATCATTCAACTCCACGCCCTTCTTGGAAACAGGTGGTCGGCTATTGCGG
MD17	211	AGTTCGCAGGAAGAACGAACCATCATTCAACTCCACGCCCTTCTTGGAAACAGGTGGTCGGCTATTGCGG
T586	211	AGTTCGCAGGAAGAACGAACCATCATTCAACTCCACGCCCTTCTTGGAAACAGGTGGTCGGCTATTGCGG
11452GZ	211	AGTTCGCAGGAAGAACGAACCATCATTCAACTCCACGCCCTTCTTGGAAACAGGTGGTCGGCTATTGCGG
GZNn2-1	211	AGTTCGCAGGAAGAACGAACCATCATTCAACTCCACGCCCTTCTTGGAAACAGGTGGTCGGCTATTGCGG
081925 fl	211	AGTTCGCAGGAAGAACGAACCATCATTCAACTCCACGCCCTTCTTGGAAACAGGTGGTCGGCTATTGCGG
gznn1-1	211	AGTTCGCAGGAAGAACGAACCATCATTCAACTCCACGCCCTTCTTGGAAACAGGTGGTCGGCTATTGCGG
TaoGZ	211	AGTTCGCAGGAAGAACGAACCATCATTCAACTCCACGCCCTTCTTGGAAACAGGTGGTCGGCTATTGCGG
n2	211	AGTTCGCAGGAAGAACGAACCATCATTCAACTCCACGCCCTTCTTGGAAACAGGTGGTCGGCTATTGCGG
N1	211	AGTTCGCAGGAAGAACGAACCATCATTCAACTCCACGCCCTTCTTGGAAACAGGTGGTCGGCTATTGCGG
JZ-fl	211	AGTTCGCAGGAAGAACGAACCATCATTCAACTCCACGCCCTTCTTGGAAACAGGTGGTCGGCTATTGCGG
XZ142 fl	211	AGTTCGCAGGAAGAACGAACCATCATTCAACTCCACGCCCTTCTTGGAAACAGGTGGTCGGCTATTGCGG
Shixiya1(Cotton_A_06163 BGI)	211	AGTTCGCAGGAAGAACGAACCATCATTCAACTCCATGCCCTTCTTGGAAACAGGTGGTCGGCTATTGCGG
Shixiya1(Ga12G1199.1 CRI)	211	AGTTCGCAGGAAGAACGAACCATCATTCAACTCCATGCCCTTCTTGGAAACAGGTGGTCGGCTATTGCGG
Xinhai21(GOBAR_AA13136 NAU)	211	AGTTCGCAGGAAGAACGAACCATCATTCAACTCCATGCCCTTCTTGGAAACAGGTGGTCGGCTATTGCGG
Jizha45	211	AGTTCGCAGGAAGAACGAACCATCATTCAACTCCATGCCCTTCTTGGAAACAGGTGGTCGGCTATTGCGG
Xinhai18	211	AGTTCGCAGGAAGAACGAACCATCATTCAACTCCATGCCCTTCTTGGAAACAGGTGGTCGGCTATTGCGG
9078N	211	AGTTCGCAGGAAGAACGAACCATCATTCAACTCCATGCCCTTCTTGGAAACAGGTGGTCGGCTATTGCGG
3-79	211	AGTTCGCAGGAAGAACGAACCATCATTCAACTCCATGCCCTTCTTGGAAACAGGTGGTCGGCTATTGCGG
Hai7124	211	AGTTCGCAGGAAGAACGAACCATCATTCAACTCCATGCCCTTCTTGGAAACAGGTGGTCGGCTATTGCGG
3-79(Gbscaffold6320.11.0 HZAU)	211	AGTTCGCAGGAAGAACGAACCATCATTCAACTCCATGCCCTTCTTGGAAACAGGTGGTCGGCTATTGCGG

TM-1(Gh_A12G1503 NAU)	281	CTCATTTGCCAAAAAGAACAGACAATGAGATCAAGAACTACTGGAATACACAGTTGAAGAAAAGGTTGAC
TM-1(CotAD_16204 BGI)	281	CTCATTTGCCAAAAAGAACAGACAATGAGATCAAGAACTACTGGAATACACAGTTGAAGAAAAGGTTGAC
TM-1(this study)	281	CTCATTTGCCAAAAAGAACAGACAATGAGATCAAGAACTACTGGAATACACAGTTGAAGAAAAGGTTGAC
SL1-7-1	281	CTCATTTGCCAAAAAGAACAGACAATGAGATCATGAACTACTGGAATACACAGTTGAAGAAAAGGTTGAC
MD17	281	CTCATTTGCCAAAAAGAACAGACAATGAGATCATGAACTACTGGAATACACAGTTGAAGAAAAGGTTGAC
T586	281	CTCATTTGCCAAAAAGAACAGACAATGAGATCATGAACTACTGGAATACACAGTTGAAGAAAAGGTTGAC
11452GZ	281	CTCATTTGCCAAAAAGAACAGACAATGAGATCATGAACTACTGGAATACACAGTTGAAGAAAAGGTTGAC
GZNn2-1	281	CTCATTTGCCAAAAAGAACAGACAATGAGATCATGAACTACTGGAATACACAGTTGAAGAAAAGGTTGAC
081925 fl	281	CTCATTTGCCAAAAAGAACAGACAATGAGATCATGAACTACTGGAATACACAGTTGAAGAAAAGGTTGAC
gznn1-1	281	CTCATTTGCCAAAAAGAACAGACAATGAGATCATGAACTACTGGAATACACAGTTGAAGAAAAGGTTGAC
TaoGZ	281	CTCATTTGCCAAAAAGAACAGACAATGAGATCATGAACTACTGGAATACACAGTTGAAGAAAAGGTTGAC
n2	281	CTCATTTGCCAAAAAGAACAGACAATGAGATCATGAACTACTGGAATACACAGTTGAAGAAAAGGTTGAC
N1	281	CTCATTTGCCAAAAAGAACAGACAATGAGATCATGAACTACTGGAATACACAGTTGAAGAAAAGGTTGAC
JZ-fl	281	CTCATTTGCCAAAAAGAACAGACAATGAGATCATGAACTACTGGAATACACAGTTGAAGAAAAGGTTGAC
XZ142 fl	281	CTCATTTGCCAAAAAGAACAGACAATGAGATCATGAACTACTGGAATACACAGTTGAAGAAAAGGTTGAC
Shixiya1(Cotton_A_06163 BGI)	281	CTCATTTGCCAAAAAGAACAGACAATGAGATCAAGAACTACTGGAATACACAGTTGAAGAAAAGGTTGAC
Shixiya1(Ga12G1199.1 CRI)	281	CTCATTTGCCAAAAAGAACAGACAATGAGATCAAGAACTACTGGAATACACAGTTGAAGAAAAGGTTGAC
Xinhai21(GOBAR_AA13136 NAU)	281	CTCATTTGCCAAAAAGAACAGACAATGAGATCAAGAACTACTGGAATACACAGTTGAAGAAAAGGTTGAC
Jizha45	281	CTCATTTGCCAAAAAGAACAGACAATGAGATCAAGAACTACTGGAATACACAGTTGAAGAAAAGGTTGAC
Xinhai18	281	CTCATTTGCCAAAAAGAACAGACAATGAGATCAAGAACTACTGGAATACACAGTTGAAGAAAAGGTTGAC
9078N	281	CTCATTTGCCAAAAAGAACAGACAATGAGATCAAGAACTACTGGAATACACAGTTGAAGAAAAGGTTGAC
3-79	281	CTCATTTGCCAAAAAGAACAGACAATGAGATCAAGAACTACTGGAATACACAGTTGAAGAAAAGGTTGAC
Hai7124	281	CTCATTTGCCAAAAAGAACAGACAATGAGATCAAGAACTACTGGAATACACAGTTGAAGAAAAGGTTGAC
3-79(Gbscaffold6320.11.0 HZAU)	281	CTCATTTGCCAAAAAGAACAGACAATGAGATCAAGAACTACTGGAATACACAGTTGAAGAAAAGGTTGAC

TM-1(Gh_A12G1503 NAU)	351	GACGATAGGGATCGACCCTGCAACTCACAGGCCTAAAACCGATACCCTCGGTTCAACTCCCAAGGATGCC
TM-1(CotAD_16204 BGI)	351	GACGATAGGGATCGACCCTGCAACTCACAGGCCTAAAACCGATACCCTCGGTTCAACTCCCAAGGATGCC
TM-1(this study)	351	GACGATAGGGATCGACCCTGCAACTCACAGGCCTAAAACCGATACCCTCGGTTCAACTCCCAAGGATGCC
SL1-7-1	351	GACGATAGGGATCGACCCTGCAACTCACAGGCCTAAAACCGATACCCTCGGTTCAACTCCCAAGGATGCC
MD17	351	GACGATAGGGATCGACCCTGCAACTCACAGGCCTAAAACCGATACCCTCGGTTCAACTCCCAAGGATGCC
T586	351	GACGATAGGGATCGACCCTGCAACTCACAGGCCTAAAACCGATACCCTCGGTTCAACTCCCAAGGATGCC
11452GZ	351	GACGATAGGGATCGACCCTGCAACTCACAGGCCTAAAACCGATACCCTCGGTTCAACTCCCAAGGATGCC
GZNn2-1	351	GACGATAGGGATCGACCCTGCAACTCACAGGCCTAAAACCGATACCCTCGGTTCAACTCCCAAGGATGCC
081925 fl	351	GACGATAGGGATCGACCCTGCAACTCACAGGCCTAAAACCGATACCCTCGGTTCAACTCCCAAGGATGCC
gznn1-1	351	GACGATAGGGATCGACCCTGCAACTCACAGGCCTAAAACCGATACCCTCGGTTCAACTCCCAAGGATGCC
TaoGZ	351	GACGATAGGGATCGACCCTGCAACTCACAGGCCTAAAACCGATACCCTCGGTTCAACTCCCAAGGATGCC
n2	351	GACGATAGGGATCGACCCTGCAACTCACAGGCCTAAAACCGATACCCTCGGTTCAACTCCCAAGGATGCC
N1	351	GACGATAGGGATCGACCCTGCAACTCACAGGCCTAAAACCGATACCCTCGGTTCAACTCCCAAGGATGCC
JZ-fl	351	GACGATAGGGATCGACCCTGCAACTCACAGGCCTAAAACCGATACCCTCGGTTCAACTCCCAAGGATGCC
XZ142 fl	351	GACGATAGGGATCGACCCTGCAACTCACAGGCCTAAAACCGATACCCTCGGTTCAACTCCCAAGGATGCC
Shixiya1(Cotton_A_06163 BGI)	351	GACGATAGGGATCGACCCTGCAACTCACAGGCCTAAAACCGATACCCTCGGTTCAACTCCCAAGGATGCC
Shixiya1(Ga12G1199.1 CRI)	351	GACGATAGGGATCGACCCTGCAACTCACAGGCCTAAAACCGATACCCTCGGTTCAACTCCCAAGGATGCC
Xinhai21(GOBAR_AA13136 NAU)	351	GACGATAGGGATCGACCCTGCAACTCACAGGCCTAAAACCGATACCCTCGGTTCAACTCCCAAGGATGCC
Jizha45	351	GACGATAGGGATCGACCCTGCAACTCACAGGCCTAAAACCGATACCCTCGGTTCAACTCCCAAGGATGCC
Xinhai18	351	GACGATAGGGATCGACCCTGCAACTCACAGGCCTAAAACCGATACCCTCGGTTCAACTCCCAAGGATGCC
9078N	351	GACGATAGGGATCGACCCTGCAACTCACAGGCCTAAAACCGATACCCTCGGTTCAACTCCCAAGGATGCC
3-79	351	GACGATAGGGATCGACCCTGCAACTCACAGGCCTAAAACCGATACCCTCGGTTCAACTCCCAAGGATGCC
Hai7124	351	GACGATAGGGATCGACCCTGCAACTCACAGGCCTAAAACCGATACCCTCGGTTCAACTCCCAAGGATGCC
3-79(Gbscaffold6320.11.0 HZAU)	351	GACGATAGGGATCGACCCTGCAACTCACAGGCCTAAAACCGATACCCTCGGTTCAACTCCCAAGGATGCC

TM-1(Gh_A12G1503 NAU)	421	GCTAACCTTAGCCACATGGCTCAATGGGAGAGTGCTCGGTTAGAAGCTGAAGCTAGATTGGTGAGAGAGT
TM-1(CotAD_16204 BGI)	421	GCTAACCTTAGCCACATGGCTCAATGGGAGAGTGCTCGGTTAGAAGCTGAAGCTAGATTGGTGAGAGAGT
TM-1(this study)	421	GCTAACCTTAGCCACATGGCTCAATGGGAGAGTGCTCGGTTAGAAGCTGAAGCTAGATTGGTGAGAGAGT
SL1-7-1	421	GCTAACCTTAGCCACATGGCTCAATGGGAGAGTGCTCGGTTAGAAGCTGAAGCTAGATTGGTGAGAGAGT
MD17	421	GCTAACCTTAGCCACATGGCTCAATGGGAGAGTGCTCGGTTAGAAGCTGAAGCTAGATTGGTGAGAGAGT
T586	421	GCTAACCTTAGCCACATGGCTCAATGGGAGAGTGCTCGGTTAGAAGCTGAAGCTAGATTGGTGAGAGAGT

```
11452GZ                              421  GCTAACCTTAGCCACATGGCTCAATGGGAGAGTGCTCGGTTAGAAGCTGAAGCTAGATTGGTGAGAGAGT
GZNn2-1                              421  GCTAACCTTAGCCACATGGCTCAATGGGAGAGTGCTCGGTTAGAAGCTGAAGCTAGATTGGTGAGAGAGT
081925 fl                            421  GCTAACCTTAGCCACATGGCTCAATGGGAGAGTGCTCGGTTAGAAGCTGAAGCTAGATTGGTGAGAGAGT
gznn1-1                              421  GCTAACCTTAGCCACATGGCTCAATGGGAGAGTGCTCGGTTAGAAGCTGAAGCTAGATTGGTGAGAGAGT
TaoGZ                                421  GCTAACCTTAGCCACATGGCTCAATGGGAGAGTGCTCGGTTAGAAGCTGAAGCTAGATTGGTGAGAGAGT
n2                                   421  GCTAACCTTAGCCACATGGCTCAATGGGAGAGTGCTCGGTTAGAAGCTGAAGCTAGATTGGTGAGAGAGT
N1                                   421  GCTAACCTTAGCCACATGGCTCAATGGGAGAGTGCTCGGTTAGAAGCTGAAGCTAGATTGGTGAGAGAGT
JZ-fl                                421  GCTAACCTTAGCCACATGGCTCAATGGGAGAGTGCTCGGTTAGAAGCTGAAGCTAGATTGGTGAGAGAGT
XZ142 fl                             421  GCTAACCTTAGCCACATGGCTCAATGGGAGAGTGCTCGGTTAGAAGCTGAAGCTAGATTGGTGAGAGAGT
Shixiya1(Cotton_A_06163 BGI)         421  GCTAACCTTAGCCACATGGCTCAATGGGAGAGTGCTCGGTTAGAAGCTGAAGCTAGATTGGTGAGAGAGT
Shixiya1(Ga12G1199.1 CRI)            421  GCTAACCTTAGCCACATGGCTCAATGGGAGAGTGCTCGGTTAGAAGCTGAAGCTAGATTGGTGAGAGAGT
Xinhai21(GOBAR_AA13136 NAU)          421  GCTAACCTTAGCCACATGGCTCAATGGGAGAGTGCTCGGTTAGAAGCTGAAGCTAGATTGGTGAGAGAGT
Jizha45                              421  GCTAACCTTAGCCACATGGCTCAATGGGAGAGTGCTCGGTTAGAAGCTGAAGCTAGATTGGTGAGAGAGT
Xinhai18                             421  GCTAACCTTAGCCACATGGCTCAATGGGAGAGTGCTCGGTTAGAAGCTGAAGCTAGATTGGTGAGAGAGT
9078N                                421  GCTAACCTTAGCCACATGGCTCAATGGGAGAGTGCTCGGTTAGAAGCTGAAGCTAGATTGGTGAGAGAGT
3-79                                 421  GCTAACCTTAGCCACATGGCTCAATGGGAGAGTGCTCGGTTAGAAGCTGAAGCTAGATTGGTGAGAGAGT
Hai7124                              421  GCTAACCTTAGCCACATGGCTCAATGGGAGAGTGCTCGGTTAGAAGCTGAAGCTAGATTGGTGAGAGAGT
3-79(Gbscaffold6320.11.0 HZAU)       421  GCTAACCTTAGCCACATGGCTCAATGGGAGAGTGCTCGGTTAGAAGCTGAAGCTAGATTGGTGAGAGAGT

TM-1(Gh_A12G1503 NAU)                491  CGAAACGAGTTTCAAACCCTCCGCAAAACCAATTTAGGTTCACGTCTTCATCGGCTCCTCCACTGGTAAA
TM-1(CotAD_16204 BGI)                491  CGAAACGAGTTTCAAACCCTCCGCAAAACCAATTTAGGTTCACGTCTTCATCTGCTCCTCCACTGGTAAA
TM-1(this study)                     491  CGAAACGAGTTTCAAACCCTCCGCAAAACCAATTTAGGTTCACGTCTTCATCGGCTCCTCCACTGGTAAG
SL1-7-1                              491  CGAAACGAGTTTCAAACCCTTCGCAAAACCAATTTAGGTTCACGTCTTCATCGGCTCCTCCACTGGTAAG
MD17                                 491  CGAAACGAGTTTCAAACCCTTCGCAAAACCAATTTAGGTTCACGTCTTCATCGGCTCCTCCACTGGTAAG
T586                                 491  CGAAACGAGTTTCAAACCCTTCGCAAAACCAATTTAGGTTCACGTCTTCATCGGCTCCTCCACTGGTAAG
11452GZ                              491  CGAAACGAGTTTCAAACCCTTCGCAAAACCAATTTAGGTTCACGTCTTCATCGGCTCCTCCACTGGTAAG
GZNn2-1                              491  CGAAACGAGTTTCAAACCCTTCGCAAAACCAATTTAGGTTCACGTCTTCATCGGCTCCTCCACTGGTAAG
081925 fl                            491  CGAAACGAGTTTCAAACCCTTCGCAAAACCAATTTAGGTTCACGTCTTCATCGGCTCCTCCACTGGTAAG
gznn1-1                              491  CGAAACGAGTTTCAAACCCTTCGCAAAACCAATTTAGGTTCACGTCTTCATCGGCTCCTCCACTGGTAAG
TaoGZ                                491  CGAAACGAGTTTCAAACCCTTCGCAAAACCAATTTAGGTTCACGTCTTCATCGGCTCCTCCACTGGTAAG
n2                                   491  CGAAACGAGTTTCAAACCCTTCGCAAAACCAATTTAGGTTCACGTCTTCATCGGCTCCTCCACTGGTAAG
N1                                   491  CGAAACGAGTTTCAAACCCTTCGCAAAACCAATTTAGGTTCACGTCTTCATCGGCTCCTCCACTGGTAAG
JZ-fl                                491  CGAAACGAGTTTCAAACCCTTCGCAAAACCAATTTAGGTTCACGTCTTCATCGGCTCCTCCACTGGTAAG
XZ142 fl                             491  CGAAACGAGTTTCAAACCCTTCGCAAAACCAATTTAGGTTCACGTCTTCATCGGCTCCTCCACTGGTAAG
Shixiya1(Cotton_A_06163 BGI)         491  CGAAACGAGTTTCAAACCCTCCGCAAAACCAATTTAGGTTCACGTCTTCATCGGCTCCTCCACTGGTAAG
Shixiya1(Ga12G1199.1 CRI)            491  CGAAACGAGTTTCAAACCCTCCGCAAAACCAATTTAGGTTCACGTCTTCATCGGCTCCTCCACTGGTAAG
Xinhai21(GOBAR_AA13136 NAU)          491  CGAAACGAGTTTCAAACCCTCCGCAAAACCAATTTAGGTTCACGTCTTCATCGGCTCCTCCACTGGTAAG
Jizha45                              491  CGAAACGAGTTTCAAACCCTCCGCAAAACCAATTTAGGTTCACGTCTTCATCGGCTCCTCCACTGGTAAG
Xinhai18                             491  CGAAACGAGTTTCAAACCCTCCGCAAAACCAATTTAGGTTCACGTCTTCATCGGCTCCTCCACTGGTAAG
9078N                                491  CGAAACGAGTTTCAAACCCTCCGCAAAACCAATTTAGGTTCACGTCTTCATCGGCTCCTCCACTGGTAAG
3-79                                 491  CGAAACGAGTTTCAAACCCTCCGCAAAACCAATTTAGGTTCACGTCTTCATCGGCTCCTCCACTGGTAAG
Hai7124                              491  CGAAACGAGTTTCAAACCCTCCGCAAAACCAATTTAGGTTCACGTCTTCATCGGCTCCTCCACTGGTAAG
3-79(Gbscaffold6320.11.0 HZAU)       491  CGAAACGAGTTTCAAACCCTCCGCAAAACCAATTTAGGTTCACGTCTTCATCGGCTCCTCCACTGGTAAA

TM-1(Gh_A12G1503 NAU)                561  CAAAATTGATGTTGGTTTGGCTCATGCTACTAAACCGCAATGCCTCGATGTACTCAAAGCTTGGCAACGT
TM-1(CotAD_16204 BGI)                561  CAAAATTGATGTTGGTTTGGCTCATGCTACTAAACCGCAATGCCTCGATGTACTCAAAGCTTGGCAACGT
TM-1(this study)                     561  CAAAATTGATGTTGGTTTGGCTCATGCTACTAAACCGCAATGCCTCGATGTACTCAAAGCTTGGCAACGT
SL1-7-1                              561  CAAAATTGATGTTGGTTTGGCTCATGCTACTAAACCGCAATGCCTCGATGTACTCAAAGCTTGGCAACGT
MD17                                 561  CAAAATTGATGTTGGTTTGGCTCATGCTACTAAACCGCAATGCCTCGATGTACTCAAAGCTTGGCAACGT
T586                                 561  CAAAATTGATGTTGGTTTGGCTCATGCTACTAAACCGCAATGCCTCGATGTACTCAAAGCTTGGCAACGT
11452GZ                              561  CAAAATTGATGTTGGTTTGGCTCATGCTACTAAACCGCAATGCCTCGATGTACTCAAAGCTTGGCAACGT
GZNn2-1                              561  CAAAATTGATGTTGGTTTGGCTCATGCTACTAAACCGCAATGCCTCGATGTACTCAAAGCTTGGCAACGT
081925 fl                            561  CAAAATTGATGTTGGTTTGGCTCATGCTACTAAACCGCAATGCCTCGATGTACTCAAAGCTTGGCAACGT
gznn1-1                              561  CAAAATTGATGTTGGTTTGGCTCATGCTACTAAACCGCAATGCCTCGATGTACTCAAAGCTTGGCAACGT
TaoGZ                                561  CAAAATTGATGTTGGTTTGGCTCATGCTACTAAACCGCAATGCCTCGATGTACTCAAAGCTTGGCAACGT
n2                                   561  CAAAATTGATGTTGGTTTGGCTCATGCTACTAAACCGCAATGCCTCGATGTACTCAAAGCTTGGCAACGT
N1                                   561  CAAAATTGATGTTGGTTTGGCTCATGCTACTAAACCGCAATGCCTCGATGTACTCAAAGCTTGGCAACGT
JZ-fl                                561  CAAAATTGATGTTGGTTTGGCTCATGCTACTAAACCGCAATGCCTCGATGTACTCAAAGCTTGGCAACGT
```

```
XZ142 fl                              561  CAAAATTGATGTTGGTTTGGCTCATGCTACTAAACCGCAATGCCTCGATGTACTCAAAGCTTGGCAACGT
Shixiya1(Cotton_A_06163 BGI)          561  CAAAATTGATGTTGGTTTGGCTCATGCTACTAAACCGCAATGCCTCGATGTACTCAAAGCTTGGCAACGT
Shixiya1(Ga12G1199.1 CRI)             561  CAAAATTGATGTTGGTTTGGCTCATGCTACTAAACCGCAATGCCTCGATGTACTCAAAGCTTGGCAACGT
Xinhai21(GOBAR_AA13136 NAU)           561  CAAAATTGATGTTGGTTTGGCTCATGCTACTAAACCGCAATGCCTCGATGTACTCAAAGCTTGGCAACGT
Jizha45                               561  CAAAATTGATGTTGGTTTGGCTCATGCTACTAAACCGCAATGCCTCGATGTACTCAAAGCTTGGCAACGT
Xinhai18                              561  CAAAATTGATGTTGGTTTGGCTCATGCTACTAAACCGCAATGCCTCGATGTACTCAAAGCTTGGCAACGT
9078N                                 561  CAAAATTGATGTTGGTTTGGCTCATGCTACTAAACCGCAATGCCTCGATGTACTCAAAGCTTGGCAACGT
3-79                                  561  CAAAATTGATGTTGGTTTGGCTCATGCTACTAAACCGCAATGCCTCGATGTACTCAAAGCTTGGCAACGT
Hai7124                               561  CAAAATTGATGTTGGTTTGGCTCATGCTACTAAACCGCAATGCCTCGATGTACTCAAAGCTTGGCAACGT
3-79(Gbscaffold6320.11.0 HZAU)        561  CAAAATTGATGTTGGTTTGGCTCATGCTACTAAACCGCAATGCCTCGATGTACTCAAAGCTTGGCAACGT

TM-1(Gh_A12G1503 NAU)                 631  GTAGTCACTGGATTGTTCACTTTCAACACTGACAACCTCCAATCTCCAACATCGACGTCGAGCTTCACGG
TM-1(CotAD_16204 BGI)                 631  GTAGTCACTGGATTGTTCACTTTCAACACTGACAACCTCCAATCTCCAACATCGACGTCGAGCTTCACGG
TM-1(this study)                      631  GTAGTCACTGGATTGTTCACTTTCAACACTGACAACCTCCAATCTCCAACATCGACGTCGAGCTTCACGG
SL1-7-1                               631  GTAGTCACTGGATTGTTCACTTTCAACACTGACAACCTCCAATCTCCAACATCGACGTCGAGCTTCACGG
MD17                                  631  GTAGTCACTGGATTGTTCACTTTCAACACTGACAACCTCCAATCTCCAACATCGACGTCGAGCTTCACGG
T586                                  631  GTAGTCACTGGATTGTTCACTTTCAACACTGACAACCTCCAATCTCCAACATCGACGTCGAGCTTCACGG
11452GZ                               631  GTAGTCACTGGATTGTTCACTTTCAACACTGACAACCTCCAATCTCCAACATCGACGTCGAGCTTCACGG
GZNn2-1                               631  GTAGTCACTGGATTGTTCACTTTCAACACTGACAACCTCCAATCTCCAACATCGACGTCGAGCTTCACGG
081925 fl                             631  GTAGTCACTGGATTGTTCACTTTCAACACTGACAACCTCCAATCTCCAACATCGACGTCGAGCTTCACGG
gznn1-1                               631  GTAGTCACTGGATTGTTCACTTTCAACACTGACAACCTCCAATCTCCAACATCGACGTCGAGCTTCACGG
TaoGZ                                 631  GTAGTCACTGGATTGTTCACTTTCAACACTGACAACCTCCAATCTCCAACATCGACGTCGAGCTTCACGG
n2                                    631  GTAGTCACTGGATTGTTCACTTTCAACACTGACAACCTCCAATCTCCAACATCGACGTCGAGCTTCACGG
N1                                    631  GTAGTCACTGGATTGTTCACTTTCAACACTGACAACCTCCAATCTCCAACATCGACGTCGAGCTTCACGG
JZ-fl                                 631  GTAGTCACTGGATTGTTCACTTTCAACACTGACAACCTCCAATCTCCAACATCGACGTCGAGCTTCACGG
XZ142 fl                              631  GTAGTCACTGGATTGTTCACTTTCAACACTGACAACCTCCAATCTCCAACATCGACGTCGAGCTTCACGG
Shixiya1(Cotton_A_06163 BGI)          631  GTAGTCACTGGATTGTTCACTTTCAACACTGACAACCTCCAATCTCCAACATCGACGTCGAGCTTCACGG
Shixiya1(Ga12G1199.1 CRI)             631  GTAGTCACTGGATTGTTCACTTTCAACACTGACAACCTCCAATCTCCAACATCGACGTCGAGCTTCACGG
Xinhai21(GOBAR_AA13136 NAU)           631  GTAGTCACTGGATTGTTCACTTTCAACACTGACAACCTCCAATCTCCAACATCGACGTCGAGCTTCACGG
Jizha45                               631  GTAGTCACTGGATTGTTCACTTTCAACACTGACAACCTCCAATCTCCAACATCGACGTCGAGCTTCACGG
Xinhai18                              631  GTAGTCACTGGATTGTTCACTTTCAACACTGACAACCTCCAATCTCCAACATCGACGTCGAGCTTCACGG
9078N                                 631  GTAGTCACTGGATTGTTCACTTTCAACACTGACAACCTCCAATCTCCAACATCGACGTCGAGCTTCACGG
3-79                                  631  GTAGTCACTGGATTGTTCACTTTCAACACTGACAACCTCCAATCTCCAACATCGACGTCGAGCTTCACGG
Hai7124                               631  GTAGTCACTGGATTGTTCACTTTCAACACTGACAACCTCCAATCTCCAACATCGACGTCGAGCTTCACGG
3-79(Gbscaffold6320.11.0 HZAU)        631  GTAGTCACTGGATTGTTCACTTTCAACACTGACAACCTCCAATCTCCAACATCGACGTCGAGCTTCACGG

TM-1(Gh_A12G1503 NAU)                 701  AAAACACGTTACCAATCTCATCTGTCGGGTTCATTGACAGCTTTGTGGGGAACTCAAATAACAGCTGTTG
TM-1(CotAD_16204 BGI)                 701  AAAACACGTTACCAATCTCATCTGTCGGGTTCATTGACAGCTTTGTGGGGAACTCAAATAACAGCTGTTG
TM-1(this study)                      701  AAAACACGTTACCAATCTCATCTGTCGGGTTCATTGACAGCTTTGTGGGGAACTCAAATAACAGCTGTTG
SL1-7-1                               701  AAAACACGTTACCAATCTCATCTGTCGGGTTCATTGACAGCTTTGTGGGGAACTCAAATAACAGCTGTTG
MD17                                  701  AAAACACGTTACCAATCTCATCTGTCGGGTTCATTGACAGCTTTGTGGGGAACTCAAATAACAGCTGTTG
T586                                  701  AAAACACGTTACCAATCTCATCTGTCGGGTTCATTGACAGCTTTGTGGGGAACTCAAATAACAGCTGTTG
11452GZ                               701  AAAACACGTTACCAATCTCATCTGTCGGGTTCATTGACAGCTTTGTGGGGAACTCAAATAACAGCTGTTG
GZNn2-1                               701  AAAACACGTTACCAATCTCATCTGTCGGGTTCATTGACAGCTTTGTGGGGAACTCAAATAACAGCTGTTG
081925 fl                             701  AAAACACGTTACCAATCTCATCTGTCGGGTTCATTGACAGCTTTGTGGGGAACTCAAATAACAGCTGTTG
gznn1-1                               701  AAAACACGTTACCAATCTCATCTGTCGGGTTCATTGACAGCTTTGTGGGGAACTCAAATAACAGCTGTTG
TaoGZ                                 701  AAAACACGTTACCAATCTCATCTGTCGGGTTCATTGACAGCTTTGTGGGGAACTCAAATAACAGCTGTTG
n2                                    701  AAAACACGTTACCAATCTCATCTGTCGGGTTCATTGACAGCTTTGTGGGGAACTCAAATAACAGCTGTTG
N1                                    701  AAAACACGTTACCAATCTCATCTGTCGGGTTCATTGACAGCTTTGTGGGGAACTCAAATAACAGCTGTTG
JZ-fl                                 701  AAAACACGTTACCAATCTCATCTGTCGGGTTCATTGACAGCTTTGTGGGGAACTCAAATAACAGCTGTTG
XZ142 fl                              701  AAAACACGTTACCAATCTCATCTGTCGGGTTCATTGACAGCTTTGTGGGGAACTCAAATAACAGCTGTTG
Shixiya1(Cotton_A_06163 BGI)          701  AAAACACGTTACCAATCTCATCTGTCGGGTTCATTGACAGCTTTGTGGGGAACTCAAATAACAGCTGTTG
Shixiya1(Ga12G1199.1 CRI)             701  AAAACACGTTACCAATCTCATCTGTCGGGTTCATTGACAGCTTTGTGGGGAACTCAAATAACAGCTGTTG
Xinhai21(GOBAR_AA13136 NAU)           701  AAAACACGTTACCAATCTCATCTGTCGGGTTCATTGACAGCTTTGTGGGGAACTCAAATAACAGCTGTTG
Jizha45                               701  AAAACACGTTACCAATCTCATCTGTCGGGTTCATTGACAGCTTTGTGGGGAACTCAAATAACAGCTGTTG
Xinhai18                              701  AAAACACGTTACCAATCTCATCTGTCGGGTTCATTGACAGCTTTGTGGGGAACTCAAATAACAGCTGTTG
9078N                                 701  AAAACACGTTACCAATCTCATCTGTCGGGTTCATTGACAGCTTTGTGGGGAACTCAAATAACAGCTGTTG
3-79                                  701  AAAACACGTTACCAATCTCATCTGTCGGGTTCATTGACAGCTTTGTGGGGAACTCAAATAACAGCTGTTG
```

```
Hai7124                          701  AAAACACGTTACCAATCTCATCTGTCGGGTTCATTGACAGCTTTGTGGGGAACTCAAATAACAGCTGTTG
3-79(Gbscaffold6320.11.0 HZAU)   701  AAAACACGTTACCAATCTCATCTGTCGGGTTCATTGACAGCTTTGTGGGGAACTCAAATAACAGCTGTTG

TM-1(Gh_A12G1503 NAU)            771  CGGAAATAATTGGGAATGTGTGGAGAAATCGAGCCAAGTTGCTGAATTACAGGAAATATTGGATAACTCA
TM-1(CotAD_16204 BGI)            771  CGGAAATAATTGGGAATGTGTGGAGAAATCGAGCCAAGTTGCTGAATTACAGGAAATATTGGATAACTCA
TM-1(this study)                 771  CGGAAATAATTGGGAATGTGTGGAGAAATCGAGCCAAGTTGCTGAATTACAGGAAATATTGGATAACTCA
SL1-7-1                          771  CGGAAATAATTGGGAATGTGTGGAGAAATCGAGCCAAGTTGCTGAATTACAGGAAAGATTGGATAACTCA
MD17                             771  CGGAAATAATTGGGAATGTGTGGAGAAATCGAGCCAAGTTGCTGAATTACAGGAAAGATTGGATAACTCA
T586                             771  CGGAAATAATTGGGAATGTGTGGAGAAATCGAGCCAAGTTGCTGAATTACAGGAAAGATTGGATAACTCA
11452GZ                          771  CGGAAATAATTGGGAATGTGTGGAGAAATCGAGCCAAGTTGCTGAATTACAGGAAAGATTGGATAACTCA
GZNn2-1                          771  CGGAAATAATTGGGAATGTGTGGAGAAATCGAGCCAAGTTGCTGAATTACAGGAAAGATTGGATAACTCA
081925 fl                        771  CGGAAATAATTGGGAATGTGTGGAGAAATCGAGCCAAGTTGCTGAATTACAGGAAAGATTGGATAACTCA
gznn1-1                          771  CGGAAATAATTGGGAATGTGTGGAGAAATCGAGCCAAGTTGCTGAATTACAGGAAAGATTGGATAACTCA
TaoGZ                            771  CGGAAATAATTGGGAATGTGTGGAGAAATCGAGCCAAGTTGCTGAATTACAGGAAAGATTGGATAACTCA
n2                               771  CGGAAATAATTGGGAATGTGTGGAGAAATCGAGCCAAGTTGCTGAATTACAGGAAAGATTGGATAACTCA
N1                               771  CGGAAATAATTGGGAATGTGTGGAGAAATCGAGCCAAGTTGCTGAATTACAGGAAAGATTGGATAACTCA
JZ-fl                            771  CGGAAATAATTGGGAATGTGTGGAGAAATCGAGCCAAGTTGCTGAATTACAGGAAAGATTGGATAACTCA
XZ142 fl                         771  CGGAAATAATTGGGAATGTGTGGAGAAATCGAGCCAAGTTGCTGAATTACAGGAAAGATTGGATAACTCA
Shixiya1(Cotton_A_06163 BGI)     771  CGGAAATAATTGGGAATGTGTGGAGAAATCGAGCCAAGTTGCTGACTTACAGGAAAGATTGGATAACTCA
Shixiya1(Ga12G1199.1 CRI)        771  CGGAAATAATTGGGAATGTGTGGAGAAATCGAGCCAAGTTGCTGACTTACAGGAAAGATTGGATAACTCA
Xinhai21(GOBAR_AA13136 NAU)      771  CGGAAATAATTGGGAATGTGTGGAGAAATCGAGCCAAGTTGCTGAATTACAGGAAAGATTGGATAACTCA
Jizha45                          771  CGGAAATAATTGGGAATGTGTGGAGAAATCGAGCCAAGTTGCTGAATTACAGGAAAGATTGGATAACTCA
Xinhai18                         771  CGGAAATAATTGGGAATGTGTGGAGAAATCGAGCCAAGTTGCTGAATTACAGGAAAGATTGGATAACTCA
9078N                            771  CGGAAATAATTGGGAATGTGTGGAGAAATCGAGCCAAGTTGCTGAATTACAGGAAAGATTGGATAACTCA
3-79                             771  CGGAAATAATTGGGAATGTGTGGAGAAATCGAGCCAAGTTGCTGAATTACAGGAAAGATTGGATAACTCA
Hai7124                          771  CGGAAATAATTGGGAATGTGTGGAGAAATCGAGCCAAGTTGCTGAATTACAGGAAAGATTGGATAACTCA
3-79(Gbscaffold6320.11.0 HZAU)   771  CGGAAATAATTGGGAATGTGTGGAGAAATCGAGCCAAGTTGCTGAATTACAGGAAAGATTGGATAACTCA

TM-1(Gh_A12G1503 NAU)            841  ATGGGGTTGCATGACATATTGGATCTCTCCTCAGAAGATGTATGGTTTCAAGGCTCATACAGGGCGGAAA
TM-1(CotAD_16204 BGI)            841  ATGGGGTTGCATGACATATTGGATCTCTCCTCAGAAGATGTATGGTTTCAAGGCTCATACAGGGCGGAAA
TM-1(this study)                 841  ATGGGGTTGCATGACATATTGGATCTCTCCTCAGAAGATGTATGGTTTCAAGGCTCATACAGGGCGGAAA
SL1-7-1                          841  ATGGGGTTGCATGACATATTGGATCTCTCCTCAGAAGATGTATGGTTTCAAGGCTCATACAGGGCGGAAA
MD17                             841  ATGGGGTTGCATGACATATTGGATCTCTCCTCAGAAGATGTATGGTTTCAAGGCTCATACAGGGCGGAAA
T586                             841  ATGGGGTTGCATGACATATTGGATCTCTCCTCAGAAGATGTATGGTTTCAAGGCTCATACAGGGCGGAAA
11452GZ                          841  ATGGGGTTGCATGACATATTGGATCTCTCCTCAGAAGATGTATGGTTTCAAGGCTCATACAGGGCGGAAA
GZNn2-1                          841  ATGGGGTTGCATGACATATTGGATCTCTCCTCAGAAGATGTATGGTTTCAAGGCTCATACAGGGCGGAAA
081925 fl                        841  ATGGGGTTGCATGACATATTGGATCTCTCCTCAGAAGATGTATGGTTTCAAGGCTCATACAGGGCGGAAA
gznn1-1                          841  ATGGGGTTGCATGACATATTGGATCTCTCCTCAGAAGATGTATGGTTTCAAGGCTCATACAGGGCGGAAA
TaoGZ                            841  ATGGGGTTGCATGACATATTGGATCTCTCCTCAGAAGATGTATGGTTTCAAGGCTCATACAGGGCGGAAA
n2                               841  ATGGGGTTGCATGACATATTGGATCTCTCCTCAGAAGATGTATGGTTTCAAGGCTCATACAGGGCGGAAA
N1                               841  ATGGGGTTGCATGACATATTGGATCTCTCCTCAGAAGATGTATGGTTTCAAGGCTCATACAGGGCGGAAA
JZ-fl                            841  ATGGGGTTGCATGACATATTGGATCTCTCCTCAGAAGATGTATGGTTTCAAGGCTCATACAGGGCGGAAA
XZ142 fl                         841  ATGGGGTTGCATGACATATTGGATCTCTCCTCAGAAGATGTATGGTTTCAAGGCTCATACAGGGCGGAAA
Shixiya1(Cotton_A_06163 BGI)     841  ATGGGGTTGCATGACATATTGGATCTCTCCTCAGAAGATGTATGGTTTCAAGGCTCATACAGGGCGGAAA
Shixiya1(Ga12G1199.1 CRI)        841  ATGGG-TTGCATGACATATTGGATCTCTCCTCAGAAGATGTAT---------------------------
Xinhai21(GOBAR_AA13136 NAU)      841  ATGGGGTTGCATGACATATTGGATCTCTCCTCAGAAGATGTATGGTTTCAAGGCTCATACAGGGCGGAAA
Jizha45                          841  ATGGGGTTGCATGACATATTGGATCTCTCCTCAGAAGATGTATGGTTTCAAGGCTCATACAGGGCGGAAA
Xinhai18                         841  ATGGGGTTGCATGACATATTGGATCTCTCCTCAGAAGATGTATGGTTTCAAGGCTCATACAGGGCGGAAA
9078N                            841  ATGGGGTTGCATGACATATTGGATCTCTCCTCAGAAGATGTATGGTTTCAAGGCTCATACAGGGCGGAAA
3-79                             841  ATGGGGTTGCATGACATATTGGATCTCTCCTCAGAAGATGTATGGTTTCAAGGCTCATACAGGGCGGAAA
Hai7124                          841  ATGGGGTTGCATGACATATTGGATCTCTCCTCAGAAGATGTATGGTTTCAAGGCTCATACAGGGCGGAAA
3-79(Gbscaffold6320.11.0 HZAU)   841  ATGGGGTTGCATGACATATTGGATCTCTCCTCAGAAGATGTATGGTTTCAAGGCTCATACAGGGCGGAAA

TM-1(Gh_A12G1503 NAU)            911  ATATGATGGAAGGGTATTCGGACACGTTAATGGTTTGTGATTCTGGGGATCATCCGAAGAGTTTGTCAAT
TM-1(CotAD_16204 BGI)            911  ATATGATGGAAGGGTATTCGGACACGTTAATGGTTTGTGATTCTGGGGATCATCCGAAGAGTTTGTCAAT
TM-1(this study)                 911  ATATGATGGAAGGGTATTCGGACACGTTAATGGTTTGTGATTCTGGGGATCATCCGAAGAGTTTGTCAAT
SL1-7-1                          911  ATATGATGGAAGGGTATTCGGACACGTTAATGGTTTGTGATTCTGGGGATCATCCGAAGAGTTTGTCAAT
MD17                             911  ATATGATGGAAGGGTATTCGGACACGTTAATGGTTTGTGATTCTGGGGATCATCCGAAGAGTTTGTCAAT
```

```
T586                               911  ATATGATGGAAGGGTATTCGGACACGTTAATGGTTTGTGATTCTGGGGATCATCCGAAGAGTTTGTCAAT
11452GZ                            911  ATATGATGGAAGGGTATTCGGACACGTTAATGGTTTGTGATTCTGGGGATCATCCGAAGAGTTTGTCAAT
GZNn2-1                            911  ATATGATGGAAGGGTATTCGGACACGTTAATGGTTTGTGATTCTGGGGATCATCCGAAGAGTTTGTCAAT
081925 f1                          911  ATATGATGGAAGGGTATTCGGACACGTTAATGGTTTGTGATTCTGGGGATCATCCGAAGAGTTTGTCAAT
gznn1-1                            911  ATATGATGGAAGGGTATTCGGACACGTTAATGGTTTGTGATTCTGGGGATCATCCGAAGAGTTTGTCAAT
TaoGZ                              911  ATATGATGGAAGGGTATTCGGACACGTTAATGGTTTGTGATTCTGGGGATCATCCGAAGAGTTTGTCAAT
n2                                 911  ATATGATGGAAGGGTATTCGGACACGTTAATGGTTTGTGATTCTGGGGATCATCCGAAGAGTTTGTCAAT
N1                                 911  ATATGATGGAAGGGTATTCGGACACGTTAATGGTTTGTGATTCTGGGGATCATCCGAAGAGTTTGTCAAT
JZ-f1                              911  ATATGATGGAAGGGTATTCGGACACGTTAATGGTTTGTGATTCTGGGGATCATCCGAAGAGTTTGTCAAT
XZ142 f1                           911  ATATGATGGAAGGGTATTCGGACACGTTAATGGTTTGTGATTCTGGGGATCATCCGAAGAGTTTGTCAAT
Shixiya1(Cotton_A_06163 BGI)       911  ATATGATGGAAGGGTATTCGGACACGTTAATGGTTTGTGATTCTGGGGATCATCCGAAGAGTTTGTCAAT
Shixiya1(Ga12G1199.1 CRI)          882  -----------------TCGGACACGTTAATGGTTTGTGATTCTGGGGATCATCCGAAGAGTTTGTCAAT
Xinhai21(GOBAR_AA13136 NAU)        911  ATATGATGGAAGGGTATTCGGACACGTTAATGGTTTGTGATTCTGGGGATCATCCGAAGAGTTTGTCAAT
Jizha45                            911  ATATGATGGAAGGGTATTCGGACACGTTAATGGTTTGTGATTCTGGGGATCATCCGAAGAGTTTGTCAAT
Xinhai18                           911  ATATGATGGAAGGGTATTCGGACACGTTAATGGTTTGTGATTCTGGGGATCATCCGAAGAGTTTGTCAAT
9078N                              911  ATATGATGGAAGGGTATTCGGACACGTTAATGGTTTGTGATTCTGGGGATCATCCGAAGAGTTTGTCAAT
3-79                               911  ATATGATGGAAGGGTATTCGGACACGTTAATGGTTTGTGATTCTGGGGATCATCCGAAGAGTTTGTCAAT
Hai7124                            911  ATATGATGGAAGGGTATTCGGACACGTTAATGGTTTGTGATTCTGGGGATCATCCGAAGAGTTTGTCAAT
3-79(Gbscaffold6320.11.0 HZAU)     911  ATATGATGGAAGGGTATTCGGACACGTTAATGGTTTGTGATTCTGGGGATCATCAGAAGAGTTTGTCAAT

TM-1(Gh_A12G1503 NAU)              981  GGAGCCTAGACAAAACTTTAATGTTGGAACAAGTAATGCTAGTAGTTTCGAAGAAAACAAGAATTACTGG
TM-1(CotAD_16204 BGI)              981  GGAGCCTAGACAAAACTTTAATGTTGGAACAAGTAATGCTAGTAGTTTCGAAGAAAACAAGAATTACTGG
TM-1(this study)                   981  GGAGCCTAGACAAAACTTTAATGTTGGAACAAGTAATGCTAGTAGTTTCGAAGAAAACAAGAATTACTGG
SL1-7-1                            981  GGAGCCTAGACAAAACTTTAATGTTGGAACAAGTAATGCTAGTAGTTTCGAAGAAAACAAGAATTACTGG
MD17                               981  GGAGCCTAGACAAAACTTTAATGTTGGAACAAGTAATGCTAGTAGTTTCGAAGAAAACAAGAATTACTGG
T586                               981  GGAGCCTAGACAAAACTTTAATGTTGGAACAAGTAATGCTAGTAGTTTCGAAGAAAACAAGAATTACTGG
11452GZ                            981  GGAGCCTAGACAAAACTTTAATGTTGGAACAAGTAATGCTAGTAGTTTCGAAGAAAACAAGAATTACTGG
GZNn2-1                            981  GGAGCCTAGACAAAACTTTAATGTTGGAACAAGTAATGCTAGTAGTTTCGAAGAAAACAAGAATTACTGG
081925 f1                          981  GGAGCCTAGACAAAACTTTAATGTTGGAACAAGTAATGCTAGTAGTTTCGAAGAAAACAAGAATTACTGG
gznn1-1                            981  GGAGCCTAGACAAAACTTTAATGTTGGAACAAGTAATGCTAGTAGTTTCGAAGAAAACAAGAATTACTGG
TaoGZ                              981  GGAGCCTAGACAAAACTTTAATGTTGGAACAAGTAATGCTAGTAGTTTCGAAGAAAACAAGAATTACTGG
n2                                 981  GGAGCCTAGACAAAACTTTAATGTTGGAACAAGTAATGCTAGTAGTTTCGAAGAAAACAAGAATTACTGG
N1                                 981  GGAGCCTAGACAAAACTTTAATGTTGGAACAAGTAATGCTAGTAGTTTCGAAGAAAACAAGAATTACTGG
JZ-f1                              981  GGAGCCTAGACAAAACTTTAATGTTGGAACAAGTAATGCTAGTAGTTTCGAAGAAAACAAGAATTACTGG
XZ142 f1                           981  GGAGCCTAGACAAAACTTTAATGTTGGAACAAGTAATGCTAGTAGTTTCGAAGAAAACAAGAATTACTGG
Shixiya1(Cotton_A_06163 BGI)       981  GGAGCCTAGACAAAACTTTAATGTTGGAACAAGTAATGCTAGTAGTTTCGAAGAAAACAAGAATTACTGG
Shixiya1(Ga12G1199.1 CRI)          936  GGAGCCTAGACAAAACTTTAATGTTGGAACAAGTAATGCTAGTAGTTTCGAAGAAAACAAGAATTACTGG
Xinhai21(GOBAR_AA13136 NAU)        981  GGAGCCTAGACAAAACTTTAATGTTGGAACAAGTAATGCTAGTAGTTTCGAAGAAAACAAGAATTACTGG
Jizha45                            981  GGAGCCTAGACAAAACTTTAATGTTGGAACAAGTAATGCTAGTAGTTTCGAAGAAAACAAGAATTACTGG
Xinhai18                           981  GGAGCCTAGACAAAACTTTAATGTTGGAACAAGTAATGCTAGTAGTTTCGAAGAAAACAAGAATTACTGG
9078N                              981  GGAGCCTAGACAAAACTTTAATGTTGGAACAAGTAATGCTAGTAGTTTCGAAGAAAACAAGAATTACTGG
3-79                               981  GGAGCCTAGACAAAACTTTAATGTTGGAACAAGTAATGCTAGTAGTTTCGAAGAAAACAAGAATTACTGG
Hai7124                            981  GGAGCCTAGACAAAACTTTAATGTTGGAACAAGTAATGCTAGTAGTTTCGAAGAAAACAAGAATTACTGG
3-79(Gbscaffold6320.11.0 HZAU)     981  GGAGCCTAGACAAAACTTTAATGTTGGAACAAGTAATGCTAGTAGTTTCGAAGAAAACAAGAATTACTGG

TM-1(Gh_A12G1503 NAU)              1051 AACAACATCCTTAATTTTGCGAATGCTTCCCCTTCTGGTTCTTCTGTCTTTTGA
TM-1(CotAD_16204 BGI)              1051 AACAACATCCTTAATTTTGCGAATGCTTCCCCTTCTGGTTCTTCTGTCTTTTGA
TM-1(this study)                   1051 AACAACATCCTTAATTTTGCGAATGCTTCCCCTTCTGGTTCTTCTGTCTTTTGA
SL1-7-1                            1051 AACAACATCCTTAATTTTGCGAATGCTTCCCCTTCTGGTTCTTCTGTCTTTTGA
MD17                               1051 AACAACATCCTTAATTTTGCGAATGCTTCCCCTTCTGGTTCTTCTGTCTTTTGA
T586                               1051 AACAACATCCTTAATTTTGCGAATGCTTCCCCTTCTGGTTCTTCTGTCTTTTGA
11452GZ                            1051 AACAACATCCTTAATTTTGCGAATGCTTCCCCTTCTGGTTCTTCTGTCTTTTGA
GZNn2-1                            1051 AACAACATCCTTAATTTTGCGAATGCTTCCCCTTCTGGTTCTTCTGTCTTTTGA
081925 f1                          1051 AACAACATCCTTAATTTTGCGAATGCTTCCCCTTCTGGTTCTTCTGTCTTTTGA
gznn1-1                            1051 AACAACATCCTTAATTTTGCGAATGCTTCCCCTTCTGGTTCTTCTGTCTTTTGA
TaoGZ                              1051 AACAACATCCTTAATTTTGCGAATGCTTCCCCTTCTGGTTCTTCTGTCTTTTGA
n2                                 1051 AACAACATCCTTAATTTTGCGAATGCTTCCCCTTCTGGTTCTTCTGTCTTTTGA
N1                                 1051 AACAACATCCTTAATTTTGCGAATGCTTCCCCTTCTGGTTCTTCTGTCTTTTGA
```

JZ-fl	1051	AACAACATCCTTAATTTTGCGAATGCTTCCCCTTCTGGTTCTTCTGTCTTTTGA
XZ142 fl	1051	AACAACATCCTTAATTTTGCGAATGCTTCCCCTTCTGGTTCTTCTGTCTTTTGA
Shixiya1(Cotton_A_06163 BGI)	1051	AACAACATCCTTAATTTTGCGAATGCTTCCCCTTCTGGTTCTTCTGTCTTTTGA
Shixiya1(Ga12G1199.1 CRI)	1006	AACAACATCCTTAATTTTGCGAATGCTTCCCCTTCTGGTTCTTCTGTCTTTTGA
Xinhai21(GOBAR_AA13136 NAU)	1051	AACAACATCCTTAATTTTGCGAATGCTTCCCCTTCTGGTTCTTCTGTCTTTTGA
Jizha45	1051	AACAACATCCTTAATTTTGCGAATGCTTCCCCTTCTGGTTCTTCTGTCTTTTGA
Xinhai18	1051	AACAACATCCTTAATTTTGCGAATGCTTCCCCTTCTGGTTCTTCTGTCTTTTGA
9078N	1051	AACAACATCCTTAATTTTGCGAATGCTTCCCCTTCTGGTTCTTCTGTCTTTTGA
3-79	1051	AACAACATCCTTAATTTTGCGAATGCTTCCCCTTCTGGTTCTTCTGTCTTTTGA
Hai7124	1051	AACAACATCCTTAATTTTGCGAATGCTTCCCCTTCTGGTTCTTCTGTCTTTTGA
3-79(Gbscaffold6320.11.0 HZAU)	1051	AACAACATCCTTAATTTTGCGAATGCTTCCCCTTCTGGTTCTTCTGTCTTTTGA
