# Supplementary material for: A Retrotransposon Insertion in GhMML3_D12 Is Likely Responsible for the Lintless Locus li3 of Tetraploid Cotton
Source: Front Plant Sci. 2020 Nov 26;11:593679. doi: 10.3389/fpls.2020.593679 (PMC7725795; doi:10.3389/fpls.2020.593679)
Supplement: Supplementary file 1 [file Data_Sheet_1.zip › Fig S1-Fig S8 and Table S1-S11/Fig S5.pdf]

Fig. S5. Alignment of the protein sequences of *GhMML3_A12* and its orthologs from other plants. The mutation K105M was located in the second DNA binding motif of the MYB domain.

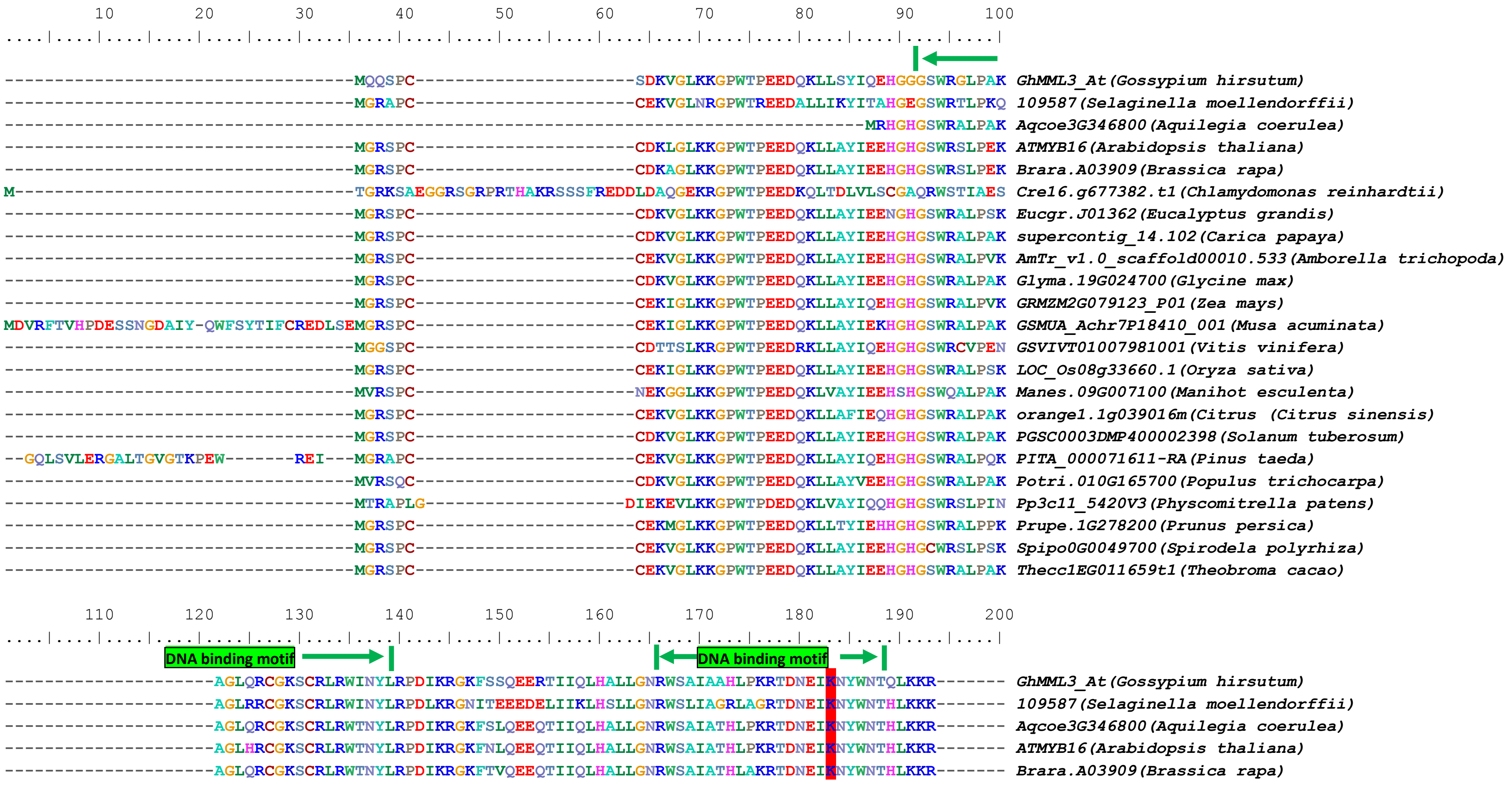

```
--------------------IPG--RSGKSCRLRWWNHLSPQVKKGPFSDFEDAVIVRSHEKYGNKWSVIAKLLPGRTDNAVKNRWNSTLKRKHTGNTLS Cre16.g677382.t1(Chlamydomonas reinhardtii)
---------------------AGLQRCGKSCRLRWTNYLRPDIKRGKFSLQEEQTIIQLHALLGNRWSAIATHLPKRTDNEIKNYWNTHLKKR------- Eucgr.J01362(Eucalyptus grandis)
---------------------AGLQRCGKSCRLRWTNYLRPDIKRGKFSLQEEQTIIQLHALLGNRWSAIATHLPKRTDNEIKNYWNTHLKKR------- supercontig_14.102(Carica papaya)
---------------------AGLLRCGKSCRLRWTNYLRPDIKRGKFSLQEEQTIIQLHALLGNRWSAIATHLPRRTDNEIKNYWNTHLKKR------- AmTr_v1.0_scaffold00010.533(Amborella trichopoda)
---------------------AGLQRCGKSCRLRWTNYLRPDIKRGKFSLQEEQTIIQLHALLGNRWSAIATHLPKRTDNEIKNYWNTHIKKR------- Glyma.19G024700(Glycine max)
AGEYSTIFPMNIVREISTNSLAGLQRCGKSCRLRWTNYLRPDIKRGKFSLQEEQTIIQLHALLGNRWSAIATHLPNRTDNEIKNYWNTHLKKR------- GRMZM2G079123_P01(Zea mays)
---------------------AGLQRCGKSCRLRWTNYLRPDIKRGEFSLQEEQTIIQLHALLGNRWSAIATHLPKRTDNEIKNYWNTHLKKR------- GSMUA_Achr7P18410_001(Musa acuminata)
---------------------AGLQRCGKSCRLRWTNYLRPDIKRGKFSLREEQTIIQLHALLGNRWSAIATHLPNRTDNEIKNYWNTHLKKR------- GSVIVT01007981001(Vitis vinifera)
---------------------AGLQRCGKSCRLRWTNYLRPDIKRGKFSLQEEQTIIQLHALLGNRWSAIATHLPKRTDNEIKNYWNTHLKKR------- LOC_Os08g33660.1(Oryza sativa)
---------------------AGLRRCGKSCRLRWINYLRPDIKRGKFSSQEEQTIIQLHALLGNKWSAIATHLPRRTDNEIKNYWNTHLKKR------- Manes.09G007100(Manihot esculenta)
---------------------AGLQRCGKSCRLRWINYLRPDIKRGKFSLQEEQTIIQLHALLGNRWSAIAAHLPKRTDNEIKNYWNTHLKKR------- orange1.1g039016m(Citrus (Citrus sinensis)
---------------------AGLQRCGKSCRLRWTNYLRPDIKRGKFTLQEEQTIIQLHALLGNRWSAIATHLPKRTDNEIKNYWNTHLKKR------- PGSC0003DMP400002398(Solanum tuberosum)
---------------------AGLLRCGKSCRLRWTNYLRPDIKRGKFNPQEEQTIIQLHALLGNRWSTIATHLPKRTDNEIKNYWNTHLKKR------- PITA_000071611-RA(Pinus taeda)
---------------------AGLQRCGKSCRLRWTNYLRPDIKRGKFSLQEEQTIIQLHALLGNRWSVIATHLPKRTDNEIKNYWNTHLKKR------- Potri.010G165700(Populus trichocarpa)
---------------------AGLQRCGKSCRLRWTNYLRPDIKRGRFSHEEDQMIVHLHAILGNRWSAIASHLPRRTDNEIKNYWNTHLKKR------- Pp3c11_5420V3(Physcomitrella patens)
---------------------AGLQRCGKSCRLRWTNYLRPDIKRGKFSLQEDQSIIQLHALIGNRWSAIATHLPKRTDNEIKNYWNTHLKKR------- Prupe.1G278200(Prunus persica)
---------------------AGLQRCGKSCRLRWTNYLRPDIKRGKFSVQEEQTIIQLHALLGNRWSAIASHLPKRTDNEIKNFWNTHLKKR------- Spipo0G0049700(Spirodela polyrhiza)
---------------------AGLQRCGKSCRLRWINYLRPDIKRGKFSLQEEQTIIQLHALLGNRWSAIATHLPKRTDNEIKNYWNTHLKKR------- Thecc1EG011659t1(Theobroma cacao)

          210       220       230       240       250       260       270       280       290       300
....|....|....|....|....|....|....|....|....|....|....|....|....|....|....|....|....|....|....|....|
---------LTTIGIDPATHRPKTDTLG---------------------------------------------------------------------STP GhMML3_At(Gossypium hirsutum)
---------LRSMGIDPHTHRPL----------------------------------------------------------------------------- 109587(Selaginella moellendorffii)
---------LSKLGIDPVTHKPKSDALS------------------------------------------------------------------SVDGQT Aqcoe3G346800(Aquilegia coerulea)
---------LVKMGIDPVTHKPKNETPL------------------------------------------------------------------SSLGLS ATMYB16(Arabidopsis thaliana)
---------LVKMGIDPVTHKHKNETLM------------------------------------------------------------------SSTGQS Brara.A03909(Brassica rapa)
NKFVDTYPELEPLMADPEASREAVEYSAGLEEAKAIMGAAAAEAAAGGHHMTMTGSEEEDHEHEEEDTMSEDEAEEGGSRKHAGSASVADLGPGSAAAAA Cre16.g677382.t1(Chlamydomonas reinhardtii)
---------LAKMGIDPVTHKPKNDALV------------------------------------------------------------------SSDGQS Eucgr.J01362(Eucalyptus grandis)
---------LAKMGIDPVTHKPKNEALL------------------------------------------------------------------SSDGQS supercontig_14.102(Carica papaya)
---------LAKMGIDPVTHKPKTDALC------------------------------------------------------------------LVDGQS AmTr_v1.0_scaffold00010.533(Amborella trichopoda)
---------LTKMGIDPVTHKPKNDALL------------------------------------------------------------------SSDGQS Glyma.19G024700(Glycine max)
---------LAKMGIDPVTHKSISGTRIG----------------------------------------------------------------TTNDKST GRMZM2G079123_P01(Zea mays)
---------LAKMGIDPCTHKAKIDTLS------------------------------------------------------------------SANGHP GSMUA_Achr7P18410_001(Musa acuminata)
---------LAKMGIDPVTHKPSHAVLT------------------------------------------------------------------SPNGDS GSVIVT01007981001(Vitis vinifera)
---------LAKMGIDPVTHKAINGTLNN----------------------------------------------------------------TAGDKSA LOC_Os08g33660.1(Oryza sativa)
---------LDRMGIDPMTHKPKADAFG------------------------------------------------------------------SGSSQY Manes.09G007100(Manihot esculenta)
---------LTKMGIDPVTHKPKTNPL----------------------------------------------------------------------GQP orange1.1g039016m(Citrus (Citrus sinensis)
---------LVKMGIDPVTHKPKNDALL------------------------------------------------------------------SNDGQS PGSC0003DMP400002398(Solanum tuberosum)
---------LVQMGIDPMTHKPKSESTI------------------------------------------------------------------AVGRQS PITA_000071611-RA(Pinus taeda)
---------LDKMGIDPMTHKPKADSFG------------------------------------------------------------------SGSGHS Potri.010G165700(Populus trichocarpa)
---------LMQMGIDPVTHKSTAAEELVH--------------------------------------------------------------YSIIPGLR Pp3c11_5420V3(Physcomitrella patens)
```

```
---------LTKLGIDPMTHKPRHHHG-------------------------------------------------------------------SGSGHA Prupe.1G278200(Prunus persica)
---------LAKMGIDPVTHKPRCDTLS-----------------------------------------------------------------PADSNVA Spipo0G0049700(Spirodela polyrhiza)
---------LTKMGIDPVTHKPKTDALG------------------------------------------------------------------SASGNP Thecc1EG011659t1(Theobroma cacao)

       310       320       330       340       350       360       370       380       390       400
....|....|....|....|....|....|....|....|....|....|....|....|....|....|....|....|....|....|....|....|
KD-AANLSHMAQ--------------------------------WESARLEAEARLVRESKRVSNPP------------------------QNQFRFTSS GhMML3_At(Gossypium hirsutum)
---------------------------------------------------------------------------------------------------- 109587(Selaginella moellendorffii)
KA-DSNLSHMAQ--------------------------------WESARLEAEARLVRESKLRSTSM------------------------NNQLALSGS Aqcoe3G346800(Aquilegia coerulea)
KN-AAILSHTAQ--------------------------------WESARLEAEARLARESKLL--------------------------HLQHYQTKTSS ATMYB16(Arabidopsis thaliana)
KSAAATLSHMAQ--------------------------------WESARLEAEARLARESKLL--------------------------HYQN------- Brara.A03909(Brassica rapa)
RR-AAAVALLPQRPLSTRKHTQSRNWRALEAADDATTSEDDDGAWEAEEAAEEQRIATIATSVGGAVVLCGTGSTSASPIAQSPALAALPAHLQSASSSA Cre16.g677382.t1(Chlamydomonas reinhardtii)
KS-AAKLSHLAQ--------------------------------WESARLEAEARLARESKLRSQSF------------------------QHHHSSSNS Eucgr.J01362(Eucalyptus grandis)
KN-AANLSHMAQ--------------------------------WESARLEAEARLVRESKLRSQSS----------------------FHQHHHHSNYN supercontig_14.102(Carica papaya)
KN-AATLSHMAQ--------------------------------WESARLEAEARLVRESKLRSAST------------------------NHSNSNNNK AmTr_v1.0_scaffold00010.533(Amborella trichopoda)
KT-AANLSHMAQ--------------------------------WESARLEAEARLVRESKIRSHSL------------------------QHQLGSSSS Glyma.19G024700(Glycine max)
KA-AASLSHMAQ--------------------------------WESARLEAEARLARESKIRTATP--------------------------------- GRMZM2G079123_P01(Zea mays)
KK-VANLSHMAQ--------------------------------WESARLEAEARLARESKLRAASNS--------------------TILPQQQQMGTSSS GSMUA_Achr7P18410_001(Musa acuminata)
KN-AANLSHMAQ--------------------------------WESARLEAEARLVKDSKLR----------------------------QTQHASASA GSVIVT01007981001(Vitis vinifera)
KV-TASLSHMAQ--------------------------------WESARLEAEARLARESKMRIAAS--------------------------------- LOC_Os08g33660.1(Oryza sativa)
KD-AANLSHAAQ--------------------------------WESARLEAEARLVRESK---------------------------RPLHKQFGFSSS Manes.09G007100(Manihot esculenta)
KD-TANLSHMAQ--------------------------------WESARLEAEARLVRESKLVSKPPP------------------ANHPHDHQLGLGSS orange1.1g039016m(Citrus (Citrus sinensis)
KN-AANLSHMAQ--------------------------------WESARLEAEARLARQSKLRSNSF------------------------QNSLASQEF PGSC0003DMP400002398(Solanum tuberosum)
CNGSSNLSHMAQ--------------------------------WESARLEAESRLARESKLRAQGL--------------------------------- PITA_000071611-RA(Pinus taeda)
KG-AAHLSHMAQ--------------------------------WESARLEAEARLVRESKVIIPNPP-----------------------PNRLGSTAS Potri.010G165700(Populus trichocarpa)
PVVSTNLTHMSQ--------------------------------WDSARAEAEARLSRQSSLTSPASDL-----------------AQTSLEHQKSNLST Pp3c11_5420V3(Physcomitrella patens)
KD-AANISHMAQ--------------------------------WESARLEAEARLVRESKLQVIST---------------------TNPNHLLISSSS Prupe.1G278200(Prunus persica)
KA-SASLSHMAQ--------------------------------WESARLEAEARLVREAKLLCFAS----------------------SSVASLGASSV Spipo0G0049700(Spirodela polyrhiza)
KD-AANLSHMAQ--------------------------------WESARLEAEARLVRESKLVSNPP------------------------QNQVGSSSS Thecc1EG011659t1(Theobroma cacao)

       410       420       430       440       450       460       470       480       490       500
....|....|....|....|....|....|....|....|....|....|....|....|....|....|....|....|....|....|....|....|
S----------------------------APPLVNKIDVGLAH-ATKP------QCLDVLK----------------------------AWQRV------ GhMML3_At(Gossypium hirsutum)
---------------------------------------------------------------------------------------------------- 109587(Selaginella moellendorffii)
GVDLGQQ---------------------------------LNK-ATSTSTQ--PHCLEVMK----------------------------AWQGVWSKPTK Aqcoe3G346800(Aquilegia coerulea)
QPHHHHG----------------FTHKSLLP----------------------------------------------------------NWTTK------ ATMYB16(Arabidopsis thaliana)
-------------------------NKAAAPNNC------LSHKASST-----------------------------------------NWTKP-----N Brara.A03909(Brassica rapa)
AAAAAANAHWLQQQQQQFGGSVDAADAAPTPPAK-RARCAVSAANASPLQGL-QPSLSQLQPQQPMLTASLHAACGYPAPYAAAGTPATAFTTT------ Cre16.g677382.t1(Chlamydomonas reinhardtii)
SQVYSAA--------------SALASTSAAPPG-------LVKKATAPLPPSSPQSLDTLK----------------------------AWTGSSWPSTK Eucgr.J01362(Eucalyptus grandis)
HHLVQLN------------------NNNPTP-------------GNTTTYSSSSSSHQLVNK--------------------------TAWNGS------ supercontig_14.102(Carica papaya)
NNYSKLL--------QPPLSFSATQIQQQQPKDC----------SGLP------LCLDVLK----------------------------VWQGGWQNKIM AmTr_v1.0_scaffold00010.533(Amborella trichopoda)
```

```
TFASSSSA---------STSASALNNNNKPE-------------AQRPPPPPSRSSLDVLK----------------------------AWNSGGWLESN Glyma.19G024700(Glycine max)
------------------TPTALHAQPTILP-------------ASAA-----SPCLDVLH----------------------------AWQGA------ GRMZM2G079123_P01(Zea mays)
---------------------------------------------------------------------------------------------------- GSMUA_Achr7P18410_001(Musa acuminata)
P----------------------------APAQL------LNKMATRLTP---PRRLDVLN----------------------------AWENV------ GSVIVT01007981001(Vitis vinifera)
------------------TPSKLHAQSTNPP-------------ASTP-----SPCFDVLN----------------------------AWQSA------ LOC_Os08g33660.1(Oryza sativa)
AASASSLH--LPKF---------------SPPSK----------ATAAALSVRPKCLDVLR----------------------------AWQGM------ Manes.09G007100(Manihot esculenta)
KAA--------------------------AP-------------AGRP------QCLDVLK----------------------------AWQGV------ orange1.1g039016m(Citrus (Citrus sinensis)
TAPSPS-----------------------SP-------------LSKPVMGP-ARCLNVLK----------------------------AWNGVWTKPIN PGSC0003DMP400002398(Solanum tuberosum)
-----------------------------WP-------------ASMRNPNVNSDILTRLQ---------------------------LAGQGF------ PITA_000071611-RA(Pinus taeda)
AQVSD--------------------KRSAAP-------------PARP------QCLDVLK----------------------------AWQGVVFSMLS Potri.010G165700(Populus trichocarpa)
KNDVNNQVA-SSNF---MSSWKAQVTETLRPNFG---VVELDKPPASP-----VNLQKFLQ----------------------------EWESS------ Pp3c11_5420V3(Physcomitrella patens)
DATHQGH------------------VINKAPPG--------------------LPCLDVLR----------------------------AWQGTWSTKPT Prupe.1G278200(Prunus persica)
SSAAASPFQ-LPLLVSDSSSSHVELHNESAA-------------SSAPAP---PPCFDVLR----------------------------AWQGIWRRGST Spipo0G0049700(Spirodela polyrhiza)
SAAPQSN-------------------NAVAP-------------ATRP------QCLDVLK----------------------------AWQGV------ Thecc1EG011659t1(Theobroma cacao)

         510       520       530       540       550       560       570       580       590       600
....|....|....|....|....|....|....|....|....|....|....|....|....|....|....|....|....|....|....|....|
------------VTGLF------------TFNTDNLQSPTSTSSFTE---NTL---PISSV---GFIDS------------------FVGNSNNSCCGN- GhMML3_At(Gossypium hirsutum)
---------------------------------------------------------------------------------------------------- 109587(Selaginella moellendorffii)
CGL---------IAGE------------GGSSGLDLESPTSTLCFSD---NSM---PVSTV---GFSDNP------------MSSLDLITNSGTFEG--- Aqcoe3G346800(Aquilegia coerulea)
----------------------------PHEDQQQLESPTSTVSFSEM-KESI-----------------------------PAKIEFVGSSTGV----- ATMYB16(Arabidopsis thaliana)
QGK----------------------------GDQQLESPTSTVTFSE---NLHLMIMPS-----GENNNESSE--------IQNMTEFALSSSTSSDV-- Brara.A03909(Brassica rapa)
----NAMCVGSGFAGEALACRPAQLTQQPSLQQQQLQLPVGLEAYGSSTSNTFTGVRVSIPA--NMQQQPQQQQPYQQVAVYPPQQCQVSQSLQQMEAAM Cre16.g677382.t1(Chlamydomonas reinhardtii)
SGE-GSGGAGGSANGIA----------G------DLESPTSTLTFSEP-MNVL-------------CENP------------KQMIEFVGSSGSSDSG-- Eucgr.J01362(Eucalyptus grandis)
-------------------------------NNIDLESPTSTLTFSE---NNAPGALTAGI---GLSSSP------------IPMIEFVGTSSGSSEAG- supercontig_14.102(Carica papaya)
RCN--------------------------GQDLAMLESPTSTLSFGGD--NNSSLPNPAAVGFGGENPAAGN--------------EFVENQMMLGPSDE AmTr_v1.0_scaffold00010.533(Amborella trichopoda)
EGN-GGIVSNVGVSG-------------------DLESPTSTLSFSE---NAP-PIMNGIG---GENNNNNDS--------AMPMIEFVGNSGNSSS--- Glyma.19G024700(Glycine max)
--------------------------------KIDLESPTSTLTFTGS--NSGTLPTPRTNRL-DVSESNCA---------------------------- GRMZM2G079123_P01(Zea mays)
--------------------------------SIDLESPTSTLSLAE----------------------------------------------------- GSMUA_Achr7P18410_001(Musa acuminata)
----GSKLK-IGSSG----------------SNRDIPSPTSTLSFLE---NVS----------------------------------------------- GSVIVT01007981001(Vitis vinifera)
--------------------------------KIDLESPTSTLTFAGS--NASMLPFSTTTAL-ELSESNSN---------------------------- LOC_Os08g33660.1(Oryza sativa)
------------VSGG------------------GLESPTSTLNFPE---NALLTPVVASI----------------------PQLQFPTCNITCKGG-- Manes.09G007100(Manihot esculenta)
------------VSGMF----------GAACRDNNLESPTSTLNFNE---HVLLSGPPAVG---LFSDN------------------LVGNSI------- orange1.1g039016m(Citrus (Citrus sinensis)
EGSIASASAGISVTGAL---------------ARDLESPTSTLGYFE---NAQ-HISSSGI---GASSNT-------------VLYEFVGNSSGSSEGG- PGSC0003DMP400002398(Solanum tuberosum)
----DSDKEGSKVLGL-----------------------NSTGGFERM---------------------------------------------------- PITA_000071611-RA(Pinus taeda)
AGC-----------------------------SDSLESPTSTLNFSE---NELAMPLV------GVQKNS------------ATTLAFATNNAPCNGGT- Potri.010G165700(Populus trichocarpa)
----------------------------LKAPQPEMEGPSIHDSITNI---------------PSLSSGTAS--------------ELVSTQYSPDVSSA Pp3c11_5420V3(Physcomitrella patens)
VRNVNSTI----ISG------------GSMMSMDDLESPTSTLNFPHI--NALNPPMQNAVNINGLNENP------------------------------ Prupe.1G278200(Prunus persica)
RGR-GEAQS-------------------SRDGKIDLESPTSTLTFSEA------VLPERAMGL-GSQHNSSS---------------IAHRCGGEGDM-- Spipo0G0049700(Spirodela polyrhiza)
------------VTGLF------------TFNSDNLQSPTSTLNFME---NTL---PISSV---GFNDN------------------FVGNSINPCEGD- Thecc1EG011659t1(Theobroma cacao)
```

```
         610       620       630       640       650       660       670       680       690       700
....|....|....|....|....|....|....|....|....|....|....|....|....|....|....|....|....|....|....|....|
---------------------------------------------------------------NWECVEKS--------SQVAE-LQEILD--------N GhMML3_At(Gossypium hirsutum)
---------------------------------------------------------------------------------------------------- 109587(Selaginella moellendorffii)
-------------------------TPVKEECDE-----------------------------EYKVYEKM--------NHMGE-YKSMVNSV-----AL Aqcoe3G346800(Aquilegia coerulea)
--------------------------TLMKEPEH-----------------------------DW----------------------------------I ATMYB16(Arabidopsis thaliana)
-----------------------------KDSDQ-----------------------------DW----------------------------------- Brara.A03909(Brassica rapa)
LMQQQQLLQQQQQQ----------QQQLQQQQEQQQQQLVQQQQQAAAMADCGTELEGPNDDLEWQFIMDT--------LDAAPAEQATFD-------DW Cre16.g677382.t1(Chlamydomonas reinhardtii)
--------------------------MIKEEGEQ-----------------------------EWKGLESS--------ITLPE-FKEGMGNNAVS---F Eucgr.J01362(Eucalyptus grandis)
--------------------------IVKEEGEQV---------------------------HDWKGLTNS--------THLSE-YKESIDHNSLS--FT supercontig_14.102(Carica papaya)
KEEYLPSLCDQDEGLKCGGVYSFLQQALRRSSSQ-----------------------------NFPLTANEEEEKEEVTRKLAGNRLEKVD-------GL AmTr_v1.0_scaffold00010.533(Amborella trichopoda)
--------------------------LVKEEAEQ-----------------------------EWKSTYDS----------------------SITT-TF Glyma.19G024700(Glycine max)
--------------------------VWHQRSDDLE-----------------------DEENDWQIFSKH---------QLLE--------------LD GRMZM2G079123_P01(Zea mays)
---------------------------------------------------------------NWLPIMASG-------TELVDGNAP----------AE GSMUA_Achr7P18410_001(Musa acuminata)
-------------------------TISRVESSE------------------------------------------------------------ITGILG GSVIVT01007981001(Vitis vinifera)
--------------------------VWQQRSDELE-----------------------GEESEWKFVSKQ---------QLQG--------------MH LOC_Os08g33660.1(Oryza sativa)
---------------------------IDEDASH--------------------------SENEWKSFEKS--------NQMAHQVKETID--------E Manes.09G007100(Manihot esculenta)
-----------------------------------------------------------------PCFEKS--------GQIIEGTKDNLDNSSM----- orange1.1g039016m(Citrus (Citrus sinensis)
-------------------------IMNNEESEE-----------------------------DWKEFGNSS------TGHLPQYNKDVINENSIS---F PGSC0003DMP400002398(Solanum tuberosum)
-----------------------------------------------------------KIEDKWDKF-------------------------------- PITA_000071611-RA(Pinus taeda)
-------------------------TAIEFNSGN-----------------------------QFECFEKL--------NEAAQ-VKQNVD--------S Potri.010G165700(Populus trichocarpa)
---------------------------------------------------------------SYRMPVSSMILPSP--AQISE-------------RLM Pp3c11_5420V3(Physcomitrella patens)
--------------------------VVPSEVLQ--------------------------------------------------------DNVNVTFGCM Prupe.1G278200(Prunus persica)
------------------------------EGEE---------------------------EVDWKYFPKP--------MQLFFGSRE----------RT Spipo0G0049700(Spirodela polyrhiza)
-------------------------GIVKDSGGN-----------------------------DWKCLEKS--------NQIPE-LKERLDNS------- Thecc1EG011659t1(Theobroma cacao)

         710       720       730       740       750       760       770       780       790       800
....|....|....|....|....|....|....|....|....|....|....|....|....|....|....|....|....|....|....|....|
SMGLHDILDLSSE-------------------------------------------DVWFQ--------------------------------GSYRAEN GhMML3_At(Gossypium hirsutum)
---------------------------------------------------------------------------------------------------- 109587(Selaginella moellendorffii)
NTGFHDMGFSTVE-------------------------------------------SPWAP---------ESSKVGNEH----------------IPVTN Aqcoe3G346800(Aquilegia coerulea)
NSTMHEF---------------------------------------------------------------ETTQ----------------------MGEG ATMYB16(Arabidopsis thaliana)
---MRQINCPTEG--------------------------------------------------------------------------------------- Brara.A03909(Brassica rapa)
TAGQQQQAQAQQTQPQQAQPMQVQQVLPVPVVAAQAAPAVAGVNCYSSCSSGSAA-ATWVSGP-------QQLKSTIVVSEAPVSLPPGDWATSAQKRQR Cre16.g677382.t1(Chlamydomonas reinhardtii)
TSTFPEMTMSLE--------------------------------------------GPWTP---------ESLRASSVH--------------GNVENND Eucgr.J01362(Eucalyptus grandis)
STTLHDLTMSMD--------------------------------------------GPWTAATPDHHHHHQSLRTSST------------------NNSP supercontig_14.102(Carica papaya)
FTSLQDMVFPADNS------------------------------------------GDFKA-----------------------------------GSGF AmTr_v1.0_scaffold00010.533(Amborella trichopoda)
SSGLHEFTMNME--------------------------------------------GTWAS---------ESLRTSG-------------------SHDI Glyma.19G024700(Glycine max)
SKEREEDFIGCE--------------------------------------------EPWFS-----------------------------------EMAG GRMZM2G079123_P01(Zea mays)
AAATHQEGFSMEA-------------------------------------FGSE--APWLS---------EPYTSQAGY-----------------SWGQ GSMUA_Achr7P18410_001(Musa acuminata)
TLGTEIDAFGTQQC---------------------------------------DINMTWTM-----------------------------------ESTD GSVIVT01007981001(Vitis vinifera)
```

```
GKETEEHFIGCE--------------------------------------------ESWFP-----------------------------------GTAN LOC_Os08g33660.1(Oryza sativa)
SNILHEMTMYISE-------------------------------------------NAWVY---------DSFRATASD-----------------IMGN Manes.09G007100(Manihot esculenta)
PIGLHEMTYATDGS-----------------------------------------VATWFQ---------DSFR----------------------TAEN orange1.1g039016m(Citrus (Citrus sinensis)
TSGLQDLTLPMD--------------------------------------------TTWTA---------ESLRSNTEQ----------------ISPAN PGSC0003DMP400002398(Solanum tuberosum)
----QDLSLGIKSD--------------------------------------------------------QSNSS---------------------ALGS PITA_000071611-RA(Pinus taeda)
SVALHDISPDASNH------------------------------------------NAWFDSATN-----EN------------------------APMG Potri.010G165700(Populus trichocarpa)
ASRHCDSLLPLPRID----------------------------------LAGS---NIFPA---------QSPSAT--------------------ESSF Pp3c11_5420V3(Physcomitrella patens)
DACAATLAWFVEE-------------------------------------------GTFSSGND------EEVGNVIMP--------------AG-VSNI Prupe.1G278200(Prunus persica)
TDGFTGATFPVDSSS----------------------------------LFGADSVASWLH---------ESAKIGPGST-------------AASAIGH Spipo0G0049700(Spirodela polyrhiza)
-MGLHEMAYSSE--------------------------------------------SAWFQ---------DSYR-----------------------AEN Thecc1EG011659t1(Theobroma cacao)

         810       820       830       840       850       860       870       880       890       900
....|....|....|....|....|....|....|....|....|....|....|....|....|....|....|....|....|....|....|....|
MMEGY----------SDTLMVCDSGDHPKSLSME-----PRQNFNVGTSN-------------------------------ASSFEE-NKNYWNN-ILNF GhMML3_At(Gossypium hirsutum)
---------------------------------------------------------------------------------------------------- 109587(Selaginella moellendorffii)
FIEGF-----------TNLLLGNSDDRNSLDN------------SSGDSSDNG----------------------DADATGCEEYEE-NKNYWNN-ILNL Aqcoe3G346800(Aquilegia coerulea)
IEEGF----------TGLLLGGDSIDRSFSG---------DKNETAGESS----------------------------GGDCNYYED-NKNYLDS-IFNF ATMYB16(Arabidopsis thaliana)
IEEGF----------TSLLLLGDSGDRSLSTGK------KDEETVAGAVE--------------------------VTESDYSYYED-NKNYWNS-ILNL Brara.A03909(Brassica rapa)
TLMGL----------TQVTSCSGSGSGNASGCAVGGGLLGTATSAAGLGSGASGLAGVGLGVSGLSLVQAAPVRTSSDFPWSATAQDLDVCFFPA-TAGL Cre16.g677382.t1(Chlamydomonas reinhardtii)
MEEGF-----------TSLLLNSSGDQSLS-------------DGGGESDKR-----------------------------SDYYED-NKNYWNS-ILNL Eucgr.J01362(Eucalyptus grandis)
MEEGF-----------TNLLLNDSIDKSLSA------------DDSGKDSDQNS--------------------HGSGGGGSDYYED-NKNYWNS-ILNL supercontig_14.102(Carica papaya)
NMEGFM---------SGDLLVVLADAHSEPENRLQ----SSNDQISGENARA-----------------------------NTTEED-HKSYWNN-IFNL AmTr_v1.0_scaffold00010.533(Amborella trichopoda)
VEEGF----------TNLLLKTNSDDPSLSS------------EDGGESKNGD----------------------GGGGTNSDFYED-NNNYWNS-ILNL Glyma.19G024700(Glycine max)
VGAGF-----------TGMLLGVSNEHDASEC--------WGESNNGQTER---------------------------SNQAS-DEE-DKNYWNG-VLDM GRMZM2G079123_P01(Zea mays)
LGAGF-----------TAMLLGDSGGQNTSDSCVQ----EEEEEATGLVER----------------------------------EG-NKTYWNT-ILNS GSMUA_Achr7P18410_001(Musa acuminata)
ENANF-----------VHLLLSRSGDGILTNG----------------------------------------------GRDGEELED-NKNYWND-IMNL GSVIVT01007981001(Vitis vinifera)
IGAGF-----------TGMLLDGSNMHDTSEC--------WDESSNGQDEQ---------------------------RSQVSEDAE-NKNYWNG-IFSM LOC_Os08g33660.1(Oryza sativa)
IVEGV-----------SDIMAYNNGEQNSSMA--------------GENVTT------------------------TSQSCCANLEDMQGNYWNSLLLNL Manes.09G007100(Manihot esculenta)
IMEGL----------SSDLMICDSVDNDHNSS---------IIINAAESS--------------------------TGNVGGNFDQE-NKNFWNG-LMNL orange1.1g039016m(Citrus (Citrus sinensis)
FVETF-----------TDLLLSNSGDGDLSEGG------GTESDNGGEGS--------------------------GSGNASENCED-NKNYWNS-IFNL PGSC0003DMP400002398(Solanum tuberosum)
SFRGY-----------NPVENDQSADRASNTSYLS--LLQQAMCPSAQGSKI------------------------LYNEAKKLMEN-QKNRQANATATL PITA_000071611-RA(Pinus taeda)
IIEGL-----------SEILVCTSQDHNASFD--------------GENIN---------------------------DSCGGNLEE-NGNYWNS-LLNL Potri.010G165700(Populus trichocarpa)
SFSGLCKVDNEHQYSPTSILHGPSGHDSSYSS------------PCGSSSSAYD--SVDILAQSFSLVHNDN-HQHSRATLAMNFSQ-EPSFWTQQQVAL Pp3c11_5420V3(Physcomitrella patens)
MMEDF-----------TDVLVYNS-------------------SLAGEIS----------------------------DNGGSHFEE-NRSYWDS-ILNL Prupe.1G278200(Prunus persica)
FGDGF-----------TEMLLNNADEHSSPGAADG---CTHEMADAGKPN--------------------------------SENEE-NRSYWNS-IFNL Spipo0G0049700(Spirodela polyrhiza)
MMEGY-----------SDIMVCDSVDQQNSS------------MTPGENL--------------------------TGTSYASSFEE-NKNYWNS-ILNL Thecc1EG011659t1(Theobroma cacao)

         910       920       930       940       950       960       970
....|....|....|....|....|....|....|....|....|....|....|....|....|....|
A-NASP------------SGSS-VF---------------------------------------------                               GhMML3_At(Gossypium hirsutum)
----------------------------------------------------------------------                               109587(Selaginella moellendorffii)
```

```
V-NSSP------------SDSP-TF---------------------------------------------    Aqcoe3G346800(Aquilegia coerulea)
V-DPSP------------SDSP-MF---------------------------------------------    ATMYB16(Arabidopsis thaliana)
V-DSTP------------SDSSTMF---------------------------------------------    Brara.A03909(Brassica rapa)
YGSQSV----LQQQQQQMQQLPPMFAQPPTVAQSSATAYMPCGPSVVSFTDFSRMAGPFNDPLA---FLV    Cre16.g677382.t1(Chlamydomonas reinhardtii)
V-NSSP------------SESP-MF---------------------------------------------    Eucgr.J01362(Eucalyptus grandis)
V-NSSP------------SDSPTMF---------------------------------------------    supercontig_14.102(Carica papaya)
V-NSPP------------AASP-IF---------------------------------------------    AmTr_v1.0_scaffold00010.533(Amborella trichopoda)
V-NSSP------------SHSP-MF---------------------------------------------    Glyma.19G024700(Glycine max)
VNSELT------------PKSPPFV---------------------------------------------    GRMZM2G079123_P01(Zea mays)
VNSSAS------------SNSPPVFYH-------------------------------------------    GSMUA_Achr7P18410_001(Musa acuminata)
M-TCQP------------SRSPNL----------------------------------------------    GSVIVT01007981001(Vitis vinifera)
VNSEQP------------PLQPPLL---------------------------------------------    LOC_Os08g33660.1(Oryza sativa)
VDGPVP------------FGSPVL----------------------------------------------    Manes.09G007100(Manihot esculenta)
V-SAST------------TWSP-VF---------------------------------------------    orange1.1g039016m(Citrus (Citrus sinensis)
VNNPSP------------SDSA-MF---------------------------------------------    PGSC0003DMP400002398(Solanum tuberosum)
YNVHQPHG--------NCSESKHL----------------------------------------------    PITA_000071611-RA(Pinus taeda)
V-DASP------------TGTSPVF---------------------------------------------    Potri.010G165700(Populus trichocarpa)
VDALQPESYFTHELGVNIAQKPPSHSFNLSVN------------SMV-----NQVGVPNNIPIPQFDYLC    Pp3c11_5420V3(Physcomitrella patens)
V-NASP------------SGSP-VF---------------------------------------------    Prupe.1G278200(Prunus persica)
VTSSSA------------SNSPDCVLV-------------------------------------------    Spipo0G0049700(Spirodela polyrhiza)
V-NASP------------SGSP-VF---------------------------------------------    Thecc1EG011659t1(Theobroma cacao)
```
