# Supplementary material for: A Retrotransposon Insertion in GhMML3_D12 Is Likely Responsible for the Lintless Locus li3 of Tetraploid Cotton
Source: Front Plant Sci. 2020 Nov 26;11:593679. doi: 10.3389/fpls.2020.593679 (PMC7725795; doi:10.3389/fpls.2020.593679)
Supplement: Supplementary file 1 [file Data_Sheet_1.zip › Fig S1-Fig S8 and Table S1-S11/Fig S6.pdf]

Fig. S6. Alignment of the coding sequences of *MML3_D12* from normal lines and mutants. Normal lines: TM-1, Caozao3; fuzzless mutants: 11452GZ, TaoGZ, SA27, Zhousuohongjijiaoye, gznn1-1, n2; Gb lines: 3-79, Hai7124, 9078N, Xinhai18, Jizha45; fiberless mutants: 081925 fl, MD17, Xu142 fl, SL1-7-1. The sequence of Ty1/*copia* LTR retrotransposon *Ghli3_ret* was removed from the sequence of Xu142 fl before alignment.

TM-1(this study)	1	ATGCAGCAGTCTCCATGTAGCGACAAGGTGGTGTTGAAGAAAGGGCCATGGACTCCAGAAGAAGACCAAA
11452GZ	1	ATGCAGCAGTCTCCATGTAGCGACAAGGTGGTGTTGAAGAAAGGGCCATGGACTCCAGAAGAAGACCAAA
TaoGZ	1	ATGCAGCAGTCTCCATGTAGCGACAAGGTGGTGTTGAAGAAAGGGCCATGGACTCCAGAAGAAGACCAAA
Xu142 fl	1	ATGCAGCAGTCTCCATGTAGCGACAAGGTGGTGTTGAAGAAAGGGCCATGGACTCCAGAAGAAGACCAAA
TM-1(CotAD_13206 BGI)	1	ATGCAGCAGTCTCCATGTAGCGACAAGGTGGTGTTGAAGAAAGGGCCATGGACTCCAGAAGAAGACCAAA
TM-1(Gh_D12G1628 NAU)	1	ATGCAGCAGTCTCCATGTAGCGACAAGGTGGTGTTGAAGAAAGGGCCATGGACTCCAGAAGAAGACCAAA
081925 fl	1	ATGCAGCAGTCTCCATGTAGCGACAAGGTGGTGTTGAAGAAAGGGCCATGGACTCCAGAAGAAGACCAAA
Caozao3	1	ATGCAGCAGTCTCCATGTAGCGACAAGGTGGTGTTGAAGAAAGGGCCATGGACTCCAGAAGAAGACCAAA
SL1-7-1	1	ATGCAGCAGTCTCCATGTAGCGACAAGGTGGTGTTGAAGAAAGGGCCATGGACTCCAGAAGAAGACCAAA
SA27	1	ATGCAGCAGTCTCCATGTAGCGACAAGGTGGTGTTGAAGAAAGGGCCATGGACTCCAGAAGAAGACCAAA
Zhousuohongjijiaoye	1	ATGCAGCAGTCTCCATGTAGCGACAAGGTGGTGTTGAAGAAAGGGCCATGGACTCCAGAAGAAGACCAAA
3-79(this study)	1	ATGCAGCAGTCTCCATGTAGCGACAAGGTGGTGTTGAAGAAAGGGCCATGGACTCCAGAAGAAGACCAAA
Hai7124	1	ATGCAGCAGTCTCCATGTAGCGACAAGGTGGTGTTGAAGAAAGGGCCATGGACTCCAGAAGAAGACCAAA
9078N	1	ATGCAGCAGTCTCCATGTAGCGACAAGGTGGTGTTGAAGAAAGGGCCATGGACTCCAGAAGAAGACCAAA
Xinhai18	1	ATGCAGCAGTCTCCATGTAGCGACAAGGTGGTGTTGAAGAAAGGGCCATGGACTCCAGAAGAAGACCAAA
Jizha45	1	ATGCAGCAGTCTCCATGTAGCGACAAGGTGGTGTTGAAGAAAGGGCCATGGACTCCAGAAGAAGACCAAA
gznn1-1	1	ATGCAGCAGTCTCCATGTAGCGACAAGGTGGTGTTGAAGAAAGGGCCATGGACTCCAGAAGAAGACCAAA
MD17	1	ATGCAGCAGTCTCCATGTAGCGACAAGGTGGTGTTGAAGAAAGGGCCATGGACTCCAGAAGAAGACCAAA
n2	1	ATGCAGCAGTCTCCATGTAGCGACAAGGTGGTGTTGAAGAAAGGGCCATGGACTCCAGAAGAAGACCAAA
3-79(Gbscaffold4699.3.0 HZAU)	1	ATGCAGCAGTCTCCATGTAGCGACAAGGTGGTGTTGAAGAAAGGGCCATGGACTCCAGAAGAAGACCAAA
Gorai.008G179600(JGI)	1	ATGCAGCAGTCTCCATGTAGCGACAAGGTGGGGTTGAAGAAAGGGCCATGGACTCCAGAAGAAGACCAAA
TM-1(this study)	71	AACTCCTGTCTTATATTCAAGAACACGGCGGTGGAAGCTGGCGCGGCTTGCCCGCAAAAGCTGGACTTCA
11452GZ	71	AACTCCTGTCTTATATTCAAGAACACGGCGGTGGAAGCTGGCGCGGCTTGCCCGCAAAAGCTGGACTTCA
TaoGZ	71	AACTCCTGTCTTATATTCAAGAACACGGCGGTGGAAGCTGGCGCGGCTTGCCCGCAAAAGCTGGACTTCA
Xu142 fl	71	AACTCCTGTCTTATATTCAAGAACACGGCGGTGGAAGCTGGCGCGGCTTGCCCGCAAAAGCTGGACTTCA
TM-1(CotAD_13206 BGI)	71	AACTCCTGTCTTATATTCAAGAACACGGCGGTGGAAGCTGGCGCGGCTTGCCCGCAAAAGCTGGACTTCA
TM-1(Gh_D12G1628 NAU)	71	AACTCCTGTCTTATATTCAAGAACACGGCGGTGGAAGCTGGCGCGGCTTGCCCGCAAAAGCTGGACTTCA
081925 fl	71	AACTCCTGTCTTATATTCAAGAACACGGCGGTGGAAGCTGGCGCGGCTTGCCCGCAAAAGCTGGACTTCA
Caozao3	71	AACTCCTGTCTTATATTCAAGAACACGGCGGTGGAAGCTGGCGCGGCTTGCCCGCAAAAGCTGGACTTCA
SL1-7-1	71	AACTCCTGTCTTATATTCAAGAACACGGCGGTGGAAGCTGGCGCGGCTTGCCCGCAAAAGCTGGACTTCA
SA27	71	AACTCCTGTCTTATATTCAAGAACACGGCGGTGGAAGCTGGCGCGGCTTGCCCGCAAAAGCTGGACTTCA
Zhousuohongjijiaoye	71	AACTCCTGTCTTATATTCAAGAACACGGCGGTGGAAGCTGGCGCGGCTTGCCCGCAAAAGCTGGACTTCA
3-79(this study)	71	AACTCTTGTCTTATATTCAAGAACACGGCGGTGGAAGCTGGCGCGGCTTGCCCGCAAAAGCTGGACTTCA
Hai7124	71	AACTCTTGTCTTATATTCAAGAACACGGCGGTGGAAGCTGGCGCGGCTTGCCCGCAAAAGCTGGACTTCA
9078N	71	AACTCTTGTCTTATATTCAAGAACACGGCGGTGGAAGCTGGCGCGGCTTGCCCGCAAAAGCTGGACTTCA
Xinhai18	71	AACTCTTGTCTTATATTCAAGAACACGGCGGTGGAAGCTGGCGCGGCTTGCCCGCAAAAGCTGGACTTCA
Jizha45	71	AACTCTTGTCTTATATTCAAGAACACGGCGGTGGAAGCTGGCGCGGCTTGCCCGCAAAAGCTGGACTTCA
gznn1-1	71	AACTCTTGTCTTATATTCAAGAACACGGCGGTGGAAGCTGGCGCGGCTTGCCCGCAAAAGCTGGACTTCA
MD17	71	AACTCTTGTCTTATATTCAAGAACACGGCGGTGGAAGCTGGCGCGGCTTGCCCGCAAAAGCTGGACTTCA
n2	71	AACTCTTGTCTTATATTCAAGAACACGGCGGTGGAAGCTGGCGCGGCTTGCCCGCAAAAGCTGGACTTCA
3-79(Gbscaffold4699.3.0 HZAU)	71	AACTCTTGTCTTATATTCAAGAACACGGCGGTGGAAGCTGGCGCGGCTTGCCCGCAAAAGCTGGACTTCA
Gorai.008G179600(JGI)	71	AACTCTTGTCTTATATTCAAGAACACGGCGGTGGAAGCTGGCGCGGCTTGCCCGCAAAAGCTGGACTTCA

```
TM-1(this study)                 141  AAGATGTGGCAAGAGTTGTAGACTTAGGTGGATTAACTACTTAAGACCAGATATCAAAAGAGGAAAGTTC
11452GZ                          141  AAGATGTGGCAAGAGTTGTAGACTTAGGTGGATTAACTACTTAAGACCAGATATCAAAAGAGGAAAGTTC
TaoGZ                            141  AAGATGTGGCAAGAGTTGTAGACTTAGGTGGATTAACTACTTAAGACCAGATATCAAAAGAGGAAAGTTC
Xu142 fl                         141  AAGATGTGGCAAGAGTTGTAGACTTAGGTGGATTAACTACTTAAGACCAGATATCAAAAGAGGAAAGTTC
TM-1(CotAD_13206 BGI)            141  AAGATGTGGCAAGAGTTGTAGACTTAGGTGGATTAACTACTTAAGACCAGATATCAAAAGAGGAAAGTTC
TM-1(Gh_D12G1628 NAU)            141  AAGATGTGGCAAGAGTTGTAGACTTAGGTGGATTAACTACTTAAGACCAGATATCAAAAGAGGAAAGTTC
081925 fl                        141  AAGATGTGGCAAGAGTTGTAGACTTAGGTGGATTAACTACTTAAGACCAGATATCAAAAGAGGAAAGTTC
Caozao3                          141  AAGATGTGGCAAGAGTTGTAGACTTAGGTGGATTAACTACTTAAGACCAGATATCAAAAGAGGAAAGTTC
SL1-7-1                          141  AAGATGTGGCAAGAGTTGTAGACTTAGGTGGATTAACTACTTAAGACCAGATATCAAAAGAGGAAAGTTC
SA27                             141  AAGATGTGGCAAGAGTTGTAGACTTAGGTGGATTAACTACTTAAGACCAGATATCAAAAGAGGAAAGTTC
Zhousuohongjijiaoye              141  AAGATGTGGCAAGAGTTGTAGACTTAGGTGGATTAACTACTTAAGACCAGATATCAAAAGAGGAAAGTTC
3-79(this study)                 141  AAGATGTGGCAAGAGTTGTAGACTTAGGTGGATTAACTACTTAAGACCAGATATCAAAAGAGGAAAGTTC
Hai7124                          141  AAGATGTGGCAAGAGTTGTAGACTTAGGTGGATTAACTACTTAAGACCAGATATCAAAAGAGGAAAGTTC
9078N                            141  AAGATGTGGCAAGAGTTGTAGACTTAGGTGGATTAACTACTTAAGACCAGATATCAAAAGAGGAAAGTTC
Xinhai18                         141  AAGATGTGGCAAGAGTTGTAGACTTAGGTGGATTAACTACTTAAGACCAGATATCAAAAGAGGAAAGTTC
Jizha45                          141  AAGATGTGGCAAGAGTTGTAGACTTAGGTGGATTAACTACTTAAGACCAGATATCAAAAGAGGAAAGTTC
gznn1-1                          141  AAGATGTGGCAAGAGTTGTAGACTTAGGTGGATTAACTACTTAAGACCAGATATCAAAAGAGGAAAGTTC
MD17                             141  AAGATGTGGCAAGAGTTGTAGACTTAGGTGGATTAACTACTTAAGACCAGATATCAAAAGAGGAAAGTTC
n2                               141  AAGATGTGGCAAGAGTTGTAGACTTAGGTGGATTAACTACTTAAGACCAGATATCAAAAGAGGAAAGTTC
3-79(Gbscaffold4699.3.0 HZAU)    141  AAGATGTGGCAAGAGTTGTAGACTTAGGTGGATTAACTACTTAAGACCAGATATCAAAAGAGGAAAGTTC
Gorai.008G179600(JGI)            141  AAGATGTGGCAAGAGTTGTAGACTTAGGTGGATTAACTACTTAAGACCAGATATCAAAAGAGGAAAGTTC

TM-1(this study)                 211  AGTTCGCAGGAAGAACGAACCATCATTCAACTCCATGCCCTTCTTGGAAACAGGTGGTCGGCTATTGCGG
11452GZ                          211  AGTTCGCAGGAAGAACGAACCATCATTCAACTCCATGCCCTTCTTGGAAACAGGTGGTCGGCTATTGCGG
TaoGZ                            211  AGTTCGCAGGAAGAACGAACCATCATTCAACTCCATGCCCTTCTTGGAAACAGGTGGTCGGCTATTGCGG
Xu142 fl                         211  AGTTCGCAGGAAGAACGAACCATCATTCAACTCCATGCCCTTCTTGGAAACAGGTGGTCGGCTATTGCGG
TM-1(CotAD_13206 BGI)            211  AGTTCGCAGGAAGAACGAACCATCATTCAACTCCATGCCCTTCTTGGAAACAGGTGGTCGGCTATTGCGG
TM-1(Gh_D12G1628 NAU)            211  AGTTCGCAGGAAGAACGAACCATCATTCAACTCCATGCCCTTCTTGGAAACAGGTGGTCGGCTATTGCGG
081925 fl                        211  AGTTCGCAGGAAGAACGAACCATCATTCAACTCCATGCCCTTCTTGGAAACAGGTGGTCGGCTATTGCGG
Caozao3                          211  AGTTCGCAGGAAGAACGAACCATCATTCAACTCCATGCCCTTCTTGGAAACAGGTGGTCGGCTATTGCGG
SL1-7-1                          211  AGTTCGCAGGAAGAACGAACCATCATTCAACTCCATGCCCTTCTTGGAAACAGGTGGTCGGCTATTGCGG
SA27                             211  AGTTCGCAGGAAGAACGAACCATCATTCAACTCCATGCCCTTCTTGGAAACAGGTGGTCGGCTATTGCGG
Zhousuohongjijiaoye              211  AGTTCGCAGGAAGAACGAACCATCATTCAACTCCATGCCCTTCTTGGAAACAGGTGGTCGGCTATTGCGG
3-79(this study)                 211  AGTTCGCAGGAAGAACGAACCATCATTCAACTCCATGCCCTTCTTGGAAACAGGTGGTCGGCTATTGCGG
Hai7124                          211  AGTTCGCAGGAAGAACGAACCATCATTCAACTCCATGCCCTTCTTGGAAACAGGTGGTCGGCTATTGCGG
9078N                            211  AGTTCGCAGGAAGAACGAACCATCATTCAACTCCATGCCCTTCTTGGAAACAGGTGGTCGGCTATTGCGG
Xinhai18                         211  AGTTCGCAGGAAGAACGAACCATCATTCAACTCCATGCCCTTCTTGGAAACAGGTGGTCGGCTATTGCGG
Jizha45                          211  AGTTCGCAGGAAGAACGAACCATCATTCAACTCCATGCCCTTCTTGGAAACAGGTGGTCGGCTATTGCGG
gznn1-1                          211  AGTTCGCAGGAAGAACGAACCATCATTCAACTCCATGCCCTTCTTGGAAACAGGTGGTCGGCTATTGCGG
MD17                             211  AGTTCGCAGGAAGAACGAACCATCATTCAACTCCATGCCCTTCTTGGAAACAGGTGGTCGGCTATTGCGG
n2                               211  AGTTCGCAGGAAGAACGAACCATCATTCAACTCCATGCCCTTCTTGGAAACAGGTGGTCGGCTATTGCGG
3-79(Gbscaffold4699.3.0 HZAU)    211  AGTTCGCAGGAAGAACGAACCATCATTCAACTCCATGCCCTTCTTGGAAACAGGTGGTCGGCTATTGCGG
Gorai.008G179600(JGI)            211  AGTTCGCAGGAAGAACGAACCATCATTCAACTCCATGCCCTTCTTGGAAACAGGTGGTCGGCTATTGCGG

TM-1(this study)                 281  CTCATTTGCCAAAAAGAACAGACAATGAGATCAAGAACTACTGGAATACACAGTTGAAGAAAAGATTGAC
11452GZ                          281  CTCATTTGCCAAAAAGAACAGACAATGAGATCAAGAACTACTGGAATACACAGTTGAAGAAAAGATTGAC
TaoGZ                            281  CTCATTTGCCAAAAAGAACAGACAATGAGATCAAGAACTACTGGAATACACAGTTGAAGAAAAGATTGAC
Xu142 fl                         281  CTCATTTGCCAAAAAGAACAGACAATGAGATCAAGAACTACTGGAATACACAGTTGAAGAAAAGATTGAC
TM-1(CotAD_13206 BGI)            281  CTCATTTGCCAAAAAGAACAGACAATGAGATCAAGAACTACTGGAATACACAGTTGAAGAAAAGATTGAC
TM-1(Gh_D12G1628 NAU)            281  CTCATTTGCCAAAAAGAACAGACAATGAGATCAAGAACTACTGGAATACACAGTTGAAGAAAAGATTGAC
081925 fl                        281  CTCATTTGCCAAAAAGAACAGACAATGAGATCAAGAACTACTGGAATACACAGTTGAAGAAAAGATTGAC
Caozao3                          281  CTCATTTGCCAAAAAGAACAGACAATGAGATCAAGAACTACTGGAATACACAGTTGAAGAAAAGATTGAC
SL1-7-1                          281  CTCATTTGCCAAAAAGAACAGACAATGAGATCAAGAACTACTGGAATACACAGTTGAAGAAAAGATTGAC
SA27                             281  CTCATTTGCCAAAAAGAACAGACAATGAGATCAAGAACTACTGGAATACACAGTTGAAGAAAAGATTGAC
Zhousuohongjijiaoye              281  CTCATTTGCCAAAAAGAACAGACAATGAGATCAAGAACTACTGGAATACACAGTTGAAGAAAAGATTGAC
3-79(this study)                 281  CTCATTTGCCAAAAAGAACAGACAATGAGATCAAGAACTACTGGAATACACAGTTGAAGAAAAGATTGAC
Hai7124                          281  CTCATTTGCCAAAAAGAACAGACAATGAGATCAAGAACTACTGGAATACACAGTTGAAGAAAAGATTGAC
```

```
9078N                              281  CTCATTTGCCAAAAAGAACAGACAATGAGATCAAGAACTACTGGAATACACAGTTGAAGAAAAGATTGAC
Xinhai18                           281  CTCATTTGCCAAAAAGAACAGACAATGAGATCAAGAACTACTGGAATACACAGTTGAAGAAAAGATTGAC
Jizha45                            281  CTCATTTGCCAAAAAGAACAGACAATGAGATCAAGAACTACTGGAATACACAGTTGAAGAAAAGATTGAC
gznn1-1                            281  CTCATTTGCCAAAAAGAACAGACAATGAGATCAAGAACTACTGGAATACACAGTTGAAGAAAAGATTGAC
MD17                               281  CTCATTTGCCAAAAAGAACAGACAATGAGATCAAGAACTACTGGAATACACAGTTGAAGAAAAGATTGAC
n2                                 281  CTCATTTGCCAAAAAGAACAGACAATGAGATCAAGAACTACTGGAATACACAGTTGAAGAAAAGATTGAC
3-79(Gbscaffold4699.3.0 HZAU)      281  CTCATTTGCCAAAAAGAACAGACAATGAGATCAAGAACTACTGGAATACACAGTTGAAGAAAAGATTGAC
Gorai.008G179600(JGI)              281  CTCATTTGCCAAAAAGAACAGACAATGAGATCAAGAACTACTGGAATACACAGTTGAAGAAAAGATTGAC

TM-1(this study)                   351  CAAGATAGGGATCGACCCTGCAACTCACAGGCCTAAAACCGATACCCTCGGTTCAACTCCCAAGGATGTC
11452GZ                            351  CAAGATAGGGATCGACCCTGCAACTCACAGGCCTAAAACCGATACCCTCGGTTCAACTCCCAAGGATGTC
TaoGZ                              351  CAAGATAGGGATCGACCCTGCAACTCACAGGCCTAAAACCGATACCCTCGGTTCAACTCCCAAGGATGTC
Xu142 fl                           351  CAAGATAGGGATCGACCCTGCAACTCACAGGCCTAAAACCGATACCCTCGGTTCAACTCCCAAGGATGTC
TM-1(CotAD_13206 BGI)              351  CAAGATAGGGATCGACCCTGCAACTCACAGGCCTAAAACCGATACCCTCGGTTCAACTCCCAAGGATGTC
TM-1(Gh_D12G1628 NAU)              351  CAAGATAGGGATCGACCCTGCAACTCACAGGCCTAAAACCGATACCCTCGGTTCAACTCCCAAGGATGCC
081925 fl                          351  CAAGATAGGGATCGACCCTGCAACTCACAGGCCTAAAACCGATACCCTCGGTTCAACTCCCAAGGATGTC
Caozao3                            351  CAAGATAGGGATCGACCCTGCAACTCACAGGCCTAAAACCGATACCCTCGGTTCAACTCCCAAGGATGTC
SL1-7-1                            351  CAAGATAGGGATCGACCCTGCAACTCACAGGCCTAAAACCGATACCCTCGGTTCAACTCCCAAGGATGTC
SA27                               351  CAAGATAGGGATCGACCCTGCAACTCACAGGCCTAAAACCGATACCCTCGGTTCAACTCCCAAGGATGTC
Zhousuohongjijiaoye                351  CAAGATAGGGATCGACCCTGCAACTCACAGGCCTAAAACCGATACCCTCGGTTCAACTCCCAAGGATGTC
3-79(this study)                   351  CAAGATAGGGATCGACCCTGCAACTCACAGGCCTAAAACCGATACCCTCGGTTCAACTCCCAAGGATGTC
Hai7124                            351  CAAGATAGGGATCGACCCTGCAACTCACAGGCCTAAAACCGATACCCTCGGTTCAACTCCCAAGGATGTC
9078N                              351  CAAGATAGGGATCGACCCTGCAACTCACAGGCCTAAAACCGATACCCTCGGTTCAACTCCCAAGGATGTC
Xinhai18                           351  CAAGATAGGGATCGACCCTGCAACTCACAGGCCTAAAACCGATACCCTCGGTTCAACTCCCAAGGATGTC
Jizha45                            351  CAAGATAGGGATCGACCCTGCAACTCACAGGCCTAAAACCGATACCCTCGGTTCAACTCCCAAGGATGTC
gznn1-1                            351  CAAGATAGGGATCGACCCTGCAACTCACAGGCCTAAAACCGATACCCTCGGTTCAACTCCCAAGGATGTC
MD17                               351  CAAGATAGGGATCGACCCTGCAACTCACAGGCCTAAAACCGATACCCTCGGTTCAACTCCCAAGGATGTC
n2                                 351  CAAGATAGGGATCGACCCTGCAACTCACAGGCCTAAAACCGATACCCTCGGTTCAACTCCCAAGGATGTC
3-79(Gbscaffold4699.3.0 HZAU)      351  CAAGATAGGGATCGACCCTGCAACTCACAGGCCTAAAACCGATACCCTCGGTTCAACTCCCAAGGATGTC
Gorai.008G179600(JGI)              351  CAAGATAGGGATCGACCCTGCAACTCACAGGCCTAAAACCGATACCCTCGGTTCAACTCCCAAGGATGCC

TM-1(this study)                   421  GCTAACCTTAGCCACATGGCTCAATGGGAGAGTGCTCGGTTAGAAGCTGAAGCTAGATTGGTGAGAGAGT
11452GZ                            421  GCTAACCTTAGCCACATGGCTCAATGGGAGAGTGCTCGGTTAGAAGCTGAAGCTAGATTGGTGAGAGAGT
TaoGZ                              421  GCTAACCTTAGCCACATGGCTCAATGGGAGAGTGCTCGGTTAGAAGCTGAAGCTAGATTGGTGAGAGAGT
Xu142 fl                           421  GCTAACCTTAGCCACATGGCTCAATGGGAGAGTGCTCGGTTAGAAGCTGAAGCTAGATTGGTGAGAGAGT
TM-1(CotAD_13206 BGI)              421  GCTAACCTTAGCCACATGGCTCAATGGGAGAGTGCTCGGTTAGAAGCTGAAGCTAGATTGGTGAGAGAGT
TM-1(Gh_D12G1628 NAU)              421  GCTAACCTTAGCCACATGGCTCAATGGGAGAGTGCTCGGTTAGAAGCTGAAGCTAGATTGGTGAGAGAGT
081925 fl                          421  GCTAACCTTAGCCACATGGCTCAATGGGAGAGTGCTCGGTTAGAAGCTGAAGCTAGATTGGTGAGAGAGT
Caozao3                            421  GCTAACCTTAGCCACATGGCTCAATGGGAGAGTGCTCGGTTAGAAGCTGAAGCTAGATTGGTGAGAGAGT
SL1-7-1                            421  GCTAACCTTAGCCACATGGCTCAATGGGAGAGTGCTCGGTTAGAAGCTGAAGCTAGATTGGTGAGAGAGT
SA27                               421  GCTAACCTTAGCCACATGGCTCAATGGGAGAGTGCTCGGTTAGAAGCTGAAGCTAGATTGGTGAGAGAGT
Zhousuohongjijiaoye                421  GCTAACCTTAGCCACATGGCTCAATGGGAGAGTGCTCGGTTAGAAGCTGAAGCTAGATTGGTGAGAGAGT
3-79(this study)                   421  GCTAACCTTAGCCACATGGCTCAATGGGAGAGTGCTCGGTTAGAAGCTGAAGCTAGATTGGTGAGAGAGT
Hai7124                            421  GCTAACCTTAGCCACATGGCTCAATGGGAGAGTGCTCGGTTAGAAGCTGAAGCTAGATTGGTGAGAGAGT
9078N                              421  GCTAACCTTAGCCACATGGCTCAATGGGAGAGTGCTCGGTTAGAAGCTGAAGCTAGATTGGTGAGAGAGT
Xinhai18                           421  GCTAACCTTAGCCACATGGCTCAATGGGAGAGTGCTCGGTTAGAAGCTGAAGCTAGATTGGTGAGAGAGT
Jizha45                            421  GCTAACCTTAGCCACATGGCTCAATGGGAGAGTGCTCGGTTAGAAGCTGAAGCTAGATTGGTGAGAGAGT
gznn1-1                            421  GCTAACCTTAGCCACATGGCTCAATGGGAGAGTGCTCGGTTAGAAGCTGAAGCTAGATTGGTGAGAGAGT
MD17                               421  GCTAACCTTAGCCACATGGCTCAATGGGAGAGTGCTCGGTTAGAAGCTGAAGCTAGATTGGTGAGAGAGT
n2                                 421  GCTAACCTTAGCCACATGGCTCAATGGGAGAGTGCTCGGTTAGAAGCTGAAGCTAGATTGGTGAGAGAGT
3-79(Gbscaffold4699.3.0 HZAU)      421  GCTAACCTTAGCCACATGGCTCAATGGGAGAGTGCTCGGTTAGAAGCTGAAGCTAGATTGGTGAGAGAGT
Gorai.008G179600(JGI)              421  GCTAACCTTAGCCACATGGCTCAATGGGAGAGTGCTCGGTTAGAAGCTGAAGCTAGATTGGTGAGAGAGT

TM-1(this study)                   491  CGAAACGAGTTTCAAACCCTCCGCAAAACCAATTTAGGTTCACGTCTTCATCTGCTCCTCCACTGGTAAA
11452GZ                            491  CGAAACGAGTTTCAAACCCTCCGCAAAACCAATTTAGGTTCACGTCTTCATCTGCTCCTCCACTGGTAAA
TaoGZ                              491  CGAAACGAGTTTCAAACCCTCCGCAAAACCAATTTAGGTTCACGTCTTCATCTGCTCCTCCACTGGTAAA
Xu142 fl                           491  CGAAACGAGTTTCAAACCCTCCGCAAAACCAATTTAGGTTCACGTCTTCATCTGCTCCTCCACTGGTAAA
TM-1(CotAD_13206 BGI)              491  CGAAACGAGTTTCAAACCCTCCGCAAAACCAATTTAGGTTCACGTCTTCATCTGCTCCTCCACTGGTAAA
```

```
TM-1(Gh_D12G1628 NAU)              491  CGAAACGAGTTTCAAACCCTCCGCAAAACCAATTTAGGTTCACGTCTTCATCGGCTCCTCCACTGGTAAA
081925 fl                          491  CGAAACGAGTTTCAAACCCTCCGCAAAACCAATTTAGGTTCACGTCTTCATCGGCTCCTCCACTGGTAAA
Caozao3                            491  CGAAACGAGTTTCAAACCCTCCGCAAAACCAATTTAGGTTCACGTCTTCATCGGCTCCTCCACTGGTAAA
SL1-7-1                            491  CGAAACGAGTTTCAAACCCTCCGCAAAACCAATTTAGGTTCACGTCTTCATCGGCTCCTCCACTGGTAAA
SA27                               491  CGAAACGAGTTTCAAACCCTCCGCAAAACCAATTTAGGTTCACGTCTTCATCGGCTCCTCCACTGGTAAA
Zhousuohongjijiaoye                491  CGAAACGAGTTTCAAACCCTCCGCAAAACCAATTTAGGTTCACGTCTTCATCGGCTCCTCCACTGGTAAA
3-79(this study)                   491  CGAAACGAGTTTCAAACCCTCCGCAAAACCAATTTAGGTTCACGTCTTCATCGGCTCCTCCACTGGTAAA
Hai7124                            491  CGAAACGAGTTTCAAACCCTCCGCAAAACCAATTTAGGTTCACGTCTTCATCGGCTCCTCCACTGGTAAA
9078N                              491  CGAAACGAGTTTCAAACCCTCCGCAAAACCAATTTAGGTTCACGTCTTCATCGGCTCCTCCACTGGTAAA
Xinhai18                           491  CGAAACGAGTTTCAAACCCTCCGCAAAACCAATTTAGGTTCACGTCTTCATCGGCTCCTCCACTGGTAAA
Jizha45                            491  CGAAACGAGTTTCAAACCCTCCGCAAAACCAATTTAGGTTCACGTCTTCATCGGCTCCTCCACTGGTAAA
gznn1-1                            491  CGAAACGAGTTTCAAACCCTCCGCAAAACCAATTTAGGTTCACGTCTTCATCGGCTCCTCCACTGGTAAA
MD17                               491  CGAAACGAGTTTCAAACCCTCCGCAAAACCAATTTAGGTTCACGTCTTCATCGGCTCCTCCACTGGTAAA
n2                                 491  CGAAACGAGTTTCAAACCCTCCGCAAAACCAATTTAGGTTCACGTCTTCATCGGCTCCTCCACTGGTAAA
3-79(Gbscaffold4699.3.0 HZAU)      491  CGAAACGAGTTTCAAACCCTCCGCAAAACCAATTTAGGTTCACGTCTTCATCGGCTCCTCCACTGGTAAA
Gorai.008G179600(JGI)              491  CGAAACGAGTTTCAAACCCTCCGCAAAACCAATTTAGGTTCACGTCTTCATCGGCTCCTCCACTGGTAAG

TM-1(this study)                   561  CAAAATTGATGTTGGTTTGGCTCATGCTACTAAACCGCAATGCCTCGATGTACTCAAAGCTTGGCAACGT
11452GZ                            561  CAAAATTGATGTTGGTTTGGCTCATGCTACTAAACCGCAATGCCTCGATGTACTCAAAGCTTGGCAACGT
TaoGZ                              561  CAAAATTGATGTTGGTTTGGCTCATGCTACTAAACCGCAATGCCTCGATGTACTCAAAGCTTGGCAACGT
Xu142 fl                           561  CAAAATTGATGTTGGTTTGGCTCATGCTACTAAACCGCAATGCCTCGATGTACTCAAAGCTTGGCAACGT
TM-1(CotAD_13206 BGI)              561  CAAAATTGATGTTGGTTTGGCTCATGCTACTAAACCGCAATGCCTCGATGTACTCAAAGCTTGGCAACGT
TM-1(Gh_D12G1628 NAU)              561  CAAAATTGATGTTGGTTTGGCTCATGCTACTAAACCGCAATGCCTCGATGTACTCAAAGCTTGGCAACGT
081925 fl                          561  CAAAATTGATGTTGGTTTGGCTCATGCTACTAAACCGCAATGCCTCGATGTACTCAAAGCTTGGCAACGT
Caozao3                            561  CAAAATTGATGTTGGTTTGGCTCATGCTACTAAACCGCAATGCCTCGATGTACTCAAAGCTTGGCAACGT
SL1-7-1                            561  CAAAATTGATGTTGGTTTGGCTCATGCTACTAAACCGCAATGCCTCGATGTACTCAAAGCTTGGCAACGT
SA27                               561  CAAAATTGATGTTGGTTTGGCTCATGCTACTAAACCGCAATGCCTCGATGTACTCAAAGCTTGGCAACGT
Zhousuohongjijiaoye                561  CAAAATTGATGTTGGTTTGGCTCATGCTACTAAACCGCAATGCCTCGATGTACTCAAAGCTTGGCAACGT
3-79(this study)                   561  CAAAATTGATGTTGGTTTGGCTCATGCTACTAAACCGCAATGCCTCGATGTACTCAAAGCTTGGCAACGT
Hai7124                            561  CAAAATTGATGTTGGTTTGGCTCATGCTACTAAACCGCAATGCCTCGATGTACTCAAAGCTTGGCAACGT
9078N                              561  CAAAATTGATGTTGGTTTGGCTCATGCTACTAAACCGCAATGCCTCGATGTACTCAAAGCTTGGCAACGT
Xinhai18                           561  CAAAATTGATGTTGGTTTGGCTCATGCTACTAAACCGCAATGCCTCGATGTACTCAAAGCTTGGCAACGT
Jizha45                            561  CAAAATTGATGTTGGTTTGGCTCATGCTACTAAACCGCAATGCCTCGATGTACTCAAAGCTTGGCAACGT
gznn1-1                            561  CAAAATTGATGTTGGTTTGGCTCATGCTACTAAACCGCAATGCCTCGATGTACTCAAAGCTTGGCAACGT
MD17                               561  CAAAATTGATGTTGGTTTGGCTCATGCTACTAAACCGCAATGCCTCGATGTACTCAAAGCTTGGCAACGT
n2                                 561  CAAAATTGATGTTGGTTTGGCTCATGCTACTAAACCGCAATGCCTCGATGTACTCAAAGCTTGGCAACGT
3-79(Gbscaffold4699.3.0 HZAU)      561  CAAAATTGATGTTGGTTTGGCTCATGCTACTAAACCGCAATGCCTCGATGTACTCAAAGCTTGGCAACGT
Gorai.008G179600(JGI)              561  CAAAATTGATGTTGGTTTGGCTCATGCTACTAAACCGCAATGCCTCGATGTACTCAAAGCTTGGCAACGT

TM-1(this study)                   631  GTAGTCACTGGATTGTTCACTTTCAACACTGACAACCTCCAATCTCCAACATCGACGTCGAGCTTCACGG
11452GZ                            631  GTAGTCACTGGATTGTTCACTTTCAACACTGACAACCTCCAATCTCCAACATCGACGTCGAGCTTCACGG
TaoGZ                              631  GTAGTCACTGGATTGTTCACTTTCAACACTGACAACCTCCAATCTCCAACATCGACGTCGAGCTTCACGG
Xu142 fl                           631  GTAGTCACTGGATTGTTCACTTTCAACACTGACAACCTCCAATCTCCAACATCGACGTCGAGCTTCACGG
TM-1(CotAD_13206 BGI)              631  GTAGTCACTGGATTGTTCACTTTCAACACTGACAACCTCCAATCTCCAACATCGACGTCGAGCTTCACGG
TM-1(Gh_D12G1628 NAU)              631  GTAGTCACTGGATTGTTCACTTTCAACACTGACAACCTCCAATCTCCAACATCGACGTCGAGCTTCACGG
081925 fl                          631  GTAGTCACTGGATTGTTCACTTTCAACACTGACAACCTCCAATCTCCAACATCGACGTCGAGCTTCACGG
Caozao3                            631  GTAGTCACTGGATTGTTCACTTTCAACACTGACAACCTCCAATCTCCAACATCGACGTCGAGCTTCACGG
SL1-7-1                            631  GTAGTCACTGGATTGTTCACTTTCAACACTGACAACCTCCAATCTCCAACATCGACGTCGAGCTTCACGG
SA27                               631  GTAGTCACTGGATTGTTCACTTTCAACACTGACAACCTCCAATCTCCAACATCGACGTCGAGCTTCACGG
Zhousuohongjijiaoye                631  GTAGTCACTGGATTGTTCACTTTCAACACTGACAACCTCCAATCTCCAACATCGACGTCGAGCTTCACGG
3-79(this study)                   631  GTAGTCACTGGATTGTTCACTTTCAACACTGACAACCTCCAATCTCCAACATCGACGTCGAGCTTCACGG
Hai7124                            631  GTAGTCACTGGATTGTTCACTTTCAACACTGACAACCTCCAATCTCCAACATCGACGTCGAGCTTCACGG
9078N                              631  GTAGTCACTGGATTGTTCACTTTCAACACTGACAACCTCCAATCTCCAACATCGACGTCGAGCTTCACGG
Xinhai18                           631  GTAGTCACTGGATTGTTCACTTTCAACACTGACAACCTCCAATCTCCAACATCGACGTCGAGCTTCACGG
Jizha45                            631  GTAGTCACTGGATTGTTCACTTTCAACACTGACAACCTCCAATCTCCAACATCGACGTCGAGCTTCACGG
gznn1-1                            631  GTAGTCACTGGATTGTTCACTTTCAACACTGACAACCTCCAATCTCCAACATCGACGTCGAGCTTCACGG
MD17                               631  GTAGTCACTGGATTGTTCACTTTCAACACTGACAACCTCCAATCTCCAACATCGACGTCGAGCTTCACGG
n2                                 631  GTAGTCACTGGATTGTTCACTTTCAACACTGACAACCTCCAATCTCCAACATCGACGTCGAGCTTCACGG
```

3-79(Gbscaffold4699.3.0 HZAU)	631	GTAGTCACTGGATTGTTCACTTTCAACACTGACAACCTCCAATCTCCAACATCGACGTCGAGCTTCACGG
Gorai.008G179600(JGI)	631	GTAGTCACTGGATTGTTCACTTTCAACACTGACAACCTCCAATCTCCAACATCGACGTCGAGCTTCACGG

TM-1(this study)	701	AAAACAGGTTACCAATCTCATCTGTCGGATTCATTGACAGCTTTGTGGGGAACTCAAATAACAGCTGTTG
11452GZ	701	AAAACAGGTTACCAATCTCATCTGTCGGATTCATTGACAGCTTTGTGGGGAACTCAAATAACAGCTGTTG
TaoGZ	701	AAAACAGGTTACCAATCTCATCTGTCGGATTCATTGACAGCTTTGTGGGGAACTCAAATAACAGCTGTTG
Xu142 fl	701	AAAACAGGTTACCAATCTCATCTGTCGGATTCATTGACAGCTTTGTGGGGAACTCAAATAACAGCTGTTG
TM-1(CotAD_13206 BGI)	701	AAAACAGGTTACCAATCTCATCTGTCGGATTCATTGACAGCTTTGTGGGGAACTCAAATAACAGCTGTTG
TM-1(Gh_D12G1628 NAU)	701	AAAACAGGTTACCAATCTCATCTGTCGGATTCATTGACAGCTTTGTGGGGAACTCAAATAACAGCTGTTG
081925 fl	701	AAAACAGGTTACCAATCTCATCTGTCGGATTCATTGACAGCTTTGTGGGGAACTCAAATAACAGCTGTTG
Caozao3	701	AAAACAGGTTACCAATCTCATCTGTCGGATTCATTGACAGCTTTGTGGGGAACTCAAATAACAGCTGTTG
SL1-7-1	701	AAAACAGGTTACCAATCTCATCTGTCGGATTCATTGACAGCTTTGTGGGGAACTCAAATAACAGCTGTTG
SA27	701	AAAACAGGTTACCAATCTCATCTGTCGGATTCATTGACAGCTTTGTGGGGAACTCAAATAACAGCTGTTG
Zhousuohongjijiaoye	701	AAAACAGGTTACCAATCTCATCTGTCGGATTCATTGACAGCTTTGTGGGGAACTCAAATAACAGCTGTTG
3-79(this study)	701	AAAACAGGTTACCAATCTCATCTGTCGGATTCATTGACAGCTTTGTGGGGAACTCAAATAACAGCTGTTG
Hai7124	701	AAAACAGGTTACCAATCTCATCTGTCGGATTCATTGACAGCTTTGTGGGGAACTCAAATAACAGCTGTTG
9078N	701	AAAACAGGTTACCAATCTCATCTGTCGGATTCATTGACAGCTTTGTGGGGAACTCAAATAACAGCTGTTG
Xinhai18	701	AAAACAGGTTACCAATCTCATCTGTCGGATTCATTGACAGCTTTGTGGGGAACTCAAATAACAGCTGTTG
Jizha45	701	AAAACAGGTTACCAATCTCATCTGTCGGATTCATTGACAGCTTTGTGGGGAACTCAAATAACAGCTGTTG
gznn1-1	701	AAAACAGGTTACCAATCTCATCTGTCGGATTCATTGACAGCTTTGTGGGGAACTCAAATAACAGCTGTTG
MD17	701	AAAACAGGTTACCAATCTCATCTGTCGGATTCATTGACAGCTTTGTGGGGAACTCAAATAACAGCTGTTG
n2	701	AAAACAGGTTACCAATCTCATCTGTCGGATTCATTGACAGCTTTGTGGGGAACTCAAATAACAGCTGTTG
3-79(Gbscaffold4699.3.0 HZAU)	701	AAAACACGTTACCAATCTCATCTGTCGGATTCATTGACAGCTTTGTGGGGAACTCAAATAACAGCTGTTG
Gorai.008G179600(JGI)	701	AAAACAGGTTACCAATCTCATCTGTCGGATTCATTGACAGCTTTTTGGGGAACTCAAATAACAGCTGTTG

TM-1(this study)	771	CGGAAATAATTGGGAATGTGTGGAGAAATCGAGCCAAGTTGCTGAGTTACAGGAAAGATTGGATAACTCA
11452GZ	771	CGGAAATAATTGGGAATGTGTGGAGAAATCGAGCCAAGTTGCTGAGTTACAGGAAAGATTGGATAACTCA
TaoGZ	771	CGGAAATAATTGGGAATGTGTGGAGAAATCGAGCCAAGTTGCTGAGTTACAGGAAAGATTGGATAACTCA
Xu142 fl	771	CGGAAATAATTGGGAATGTGTGGAGAAATCGAGCCAAGTTGCTGAGTTACAGGAAAGATTGGATAACTCA
TM-1(CotAD_13206 BGI)	771	CGGAAATAATTGGGAATGTGTGGAGAAATCGAGCCAAGTTGCTGAGTTACAGGAAAGATTGGATAACTCA
TM-1(Gh_D12G1628 NAU)	771	CGGAAATAATTGGGAATGTGTGGAGAAATCGAGCCAAGTTGCTGAGTTACAGGAAAGATTGGATAACTCA
081925 fl	771	CGGAAATAATTGGGAATGTGTGGAGAAATCGAGCCAAGTTGCTGAGTTACAGGAAAGATTGGATAACTCA
Caozao3	771	CGGAAATAATTGGGAATGTGTGGAGAAATCGAGCCAAGTTGCTGAGTTACAGGAAAGATTGGATAACTCA
SL1-7-1	771	CGGAAATAATTGGGAATGTGTGGAGAAATCGAGCCAAGTTGCTGAGTTACAGGAAAGATTGGATAACTCA
SA27	771	CGGAAATAATTGGGAATGTGTGGAGAAATCGAGCCAAGTTGCTGAGTTACAGGAAAGATTGGATAACTCA
Zhousuohongjijiaoye	771	CGGAAATAATTGGGAATGTGTGGAGAAATCGAGCCAAGTTGCTGAGTTACAGGAAAGATTGGATAACTCA
3-79(this study)	771	CGGAAATAATTGGGAATGTGTGGAGAAATCGAGCCAAGTTGCTGAGTTACAGGAAAGATTGGATAACTCA
Hai7124	771	CGGAAATAATTGGGAATGTGTGGAGAAATCGAGCCAAGTTGCTGAGTTACAGGAAAGATTGGATAACTCA
9078N	771	CGGAAATAATTGGGAATGTGTGGAGAAATCGAGCCAAGTTGCTGAGTTACAGGAAAGATTGGATAACTCA
Xinhai18	771	CGGAAATAATTGGGAATGTGTGGAGAAATCGAGCCAAGTTGCTGAGTTACAGGAAAGATTGGATAACTCA
Jizha45	771	CGGAAATAATTGGGAATGTGTGGAGAAATCGAGCCAAGTTGCTGAGTTACAGGAAAGATTGGATAACTCA
gznn1-1	771	CGGAAATAATTGGGAATGTGTGGAGAAATCGAGCCAAGTTGCTGAGTTACAGGAAAGATTGGATAACTCA
MD17	771	CGGAAATAATTGGGAATGTGTGGAGAAATCGAGCCAAGTTGCTGAGTTACAGGAAAGATTGGATAACTCA
n2	771	CGGAAATAATTGGGAATGTGTGGAGAAATCGAGCCAAGTTGCTGAGTTACAGGAAAGATTGGATAACTCA
3-79(Gbscaffold4699.3.0 HZAU)	771	CGGAAATAATTGGGAATGTGTGGAGAAATCGAGCCAAGTTGCTGAGTTACAGGAAAGATTGGATAACTCA
Gorai.008G179600(JGI)	771	CGGAAATAATTGGGAATGTGTGGAGAAATCGAGCCAAGTTGCCGAGTTACAGGAAAGATTGGATAACTCA

TM-1(this study)	841	ATGGGGTTGCATGACATATTGGATTTCTCCTCAGAAGATGTATGGTTTCAAGGCTCATACAGGGCGGAAA
11452GZ	841	ATGGGGTTGCATGACATATTGGATTTCTCCTCAGAAGATGTATGGTTTCAAGGCTCATACAGGGCGGAAA
TaoGZ	841	ATGGGGTTGCATGACATATTGGATTTCTCCTCAGAAGATGTATGGTTTCAAGGCTCATACAGGGCGGAAA
Xu142 fl	841	ATGGGGTTGCATGACATATTGGATTTCTCCTCAGAAGATGTATGGTTTCAAGGCTCATACAGGGCGGAAA
TM-1(CotAD_13206 BGI)	841	ATGGGGTTGCATGACATATTGGATTTCTCCTCAGAAGATGTATGGTTTCAAGGCTCATACAGGGCGGAAA
TM-1(Gh_D12G1628 NAU)	841	ATGGGGTTGCATGACATATTGGATTTCTCCTCAGAAGATGTATGGTTTCAAGGCTCATACAGGGCGGAAA
081925 fl	841	ATGGGGTTGCATGACATATTGGATTTCTCCTCAGAAGATGTATGGTTTCAAGGCTCATACAGGGCGGAAA
Caozao3	841	ATGGGGTTGCATGACATATTGGATTTCTCCTCAGAAGATGTATGGTTTCAAGGCTCATACAGGGCGGAAA
SL1-7-1	841	ATGGGGTTGCATGACATATTGGATTTCTCCTCAGAAGATGTATGGTTTCAAGGCTCATACAGGGCGGAAA
SA27	841	ATGGGGTTGCATGACATATTGGATTTCTCCTCAGAAGATGTATGGTTTCAAGGCTCATACAGGGCGGAAA
Zhousuohongjijiaoye	841	ATGGGGTTGCATGACATATTGGATTTCTCCTCAGAAGATGTATGGTTTCAAGGCTCATACAGGGCGGAAA

3-79(this study) 841 ATGGGGTTGCATGACATATTGGATTTCTCCTCAGAAGATGTATGGTTTCAAGGCTCATACAGGGCGGAAA
Hai7124 841 ATGGGGTTGCATGACATATTGGATTTCTCCTCAGAAGATGTATGGTTTCAAGGCTCATACAGGGCGGAAA
9078N 841 ATGGGGTTGCATGACATATTGGATTTCTCCTCAGAAGATGTATGGTTTCAAGGCTCATACAGGGCGGAAA
Xinhai18 841 ATGGGGTTGCATGACATATTGGATTTCTCCTCAGAAGATGTATGGTTTCAAGGCTCATACAGGGCGGAAA
Jizha45 841 ATGGGGTTGCATGACATATTGGATTTCTCCTCAGAAGATGTATGGTTTCAAGGCTCATACAGGGCGGAAA
gznn1-1 841 ATGGGGTTGCATGACATATTGGATTTCTCCTCAGAAGATGTATGGTTTCAAGGCTCATACAGGGCGGAAA
MD17 841 ATGGGGTTGCATGACATATTGGATTTCTCCTCAGAAGATGTATGGTTTCAAGGCTCATACAGGGCGGAAA
n2 841 ATGGGGTTGCATGACATATTGGATTTCTCCTCAGAAGATGTATGGTTTCAAGGCTCATACAGGGCGGAAA
3-79(Gbscaffold4699.3.0 HZAU) 841 ATGGGGTTGCATGACATATTGGATTTCTCCTCAGAAGATGTATGGTTTCAAGGCTCATACAGGGCGGAAA
Gorai.008G179600(JGI) 841 ATGGGGTTGCATGACATATTGGATTTCTCCTCAGACGATGTATGGTTTCAAGGCTCATACAGGGCGGAAA

TM-1(this study) 911 ATATGATGGAAGGGTATTCGGACACGTTAATGGTTTGTGATTCTGGGGATCATCAGAAGAGTTTGTCAAT
11452GZ 911 ATATGATGGAAGGGTATTCGGACACGTTAATGGTTTGTGATTCTGGGGATCATCAGAAGAGTTTGTCAAT
TaoGZ 911 ATATGATGGAAGGGTATTCGGACACGTTAATGGTTTGTGATTCTGGGGATCATCAGAAGAGTTTGTCAAT
Xu142 fl 911 ATATGATGGAAGGGTATTCGGACACGTTAATGGTTTGTGATTCTGGGGATCATCAGAAGAGTTTGTCAAT
TM-1(CotAD_13206 BGI) 911 ATATGATGGAAGGGTATTCGGACACGTTAATGGTTTGTGATTCTGGGGATCATCCGAAGAGTTTGTCAAT
TM-1(Gh_D12G1628 NAU) 911 ATATGATGGAAGGGTATTCGGACACGTTAATGGTTTGTGATTCTGGGGATCATCCGAAGAGTTTGTCAAT
081925 fl 911 ATATGATGGAAGGGTATTCGGACACGTTAATGGTTTGTGATTCTGGGGATCATCAGAAGAGTTTGTCAAT
Caozao3 911 ATATGATGGAAGGGTATTCGGACACGTTAATGGTTTGTGATTCTGGGGATCATCAGAAGAGTTTGTCAAT
SL1-7-1 911 ATATGATGGAAGGGTATTCGGACACGTTAATGGTTTGTGATTCTGGGGATCATCAGAAGAGTTTGTCAAT
SA27 911 ATATGATGGAAGGGTATTCGGACACGTTAATGGTTTGTGATTCTGGGGATCATCAGAAGAGTTTGTCAAT
Zhousuohongjijiaoye 911 ATATGATGGAAGGGTATTCGGACACGTTAATGGTTTGTGATTCTGGGGATCATCAGAAGAGTTTGTCAAT
3-79(this study) 911 ATATGATGGAAGGGTATTCGGACACGTTAATGGTTTGTGATTCTGGGGATCATCAGAAGAGTTTGTCAAT
Hai7124 911 ATATGATGGAAGGGTATTCGGACACGTTAATGGTTTGTGATTCTGGGGATCATCAGAAGAGTTTGTCAAT
9078N 911 ATATGATGGAAGGGTATTCGGACACGTTAATGGTTTGTGATTCTGGGGATCATCAGAAGAGTTTGTCAAT
Xinhai18 911 ATATGATGGAAGGGTATTCGGACACGTTAATGGTTTGTGATTCTGGGGATCATCAGAAGAGTTTGTCAAT
Jizha45 911 ATATGATGGAAGGGTATTCGGACACGTTAATGGTTTGTGATTCTGGGGATCATCAGAAGAGTTTGTCAAT
gznn1-1 911 ATATGATGGAAGGGTATTCGGACACGTTAATGGTTTGTGATTCTGGGGATCATCAGAAGAGTTTGTCAAT
MD17 911 ATATGATGGAAGGGTATTCGGACACGTTAATGGTTTGTGATTCTGGGGATCATCAGAAGAGTTTGTCAAT
n2 911 ATATGATGGAAGGGTATTCGGACACGTTAATGGTTTGTGATTCTGGGGATCATCAGAAGAGTTTGTCAAT
3-79(Gbscaffold4699.3.0 HZAU) 911 ATATGATGGAAGGGTATTCGGACACGTTAATGGTTTGTGATTCTGGGGATCATCCGAAGAGTTTGTCAAT
Gorai.008G179600(JGI) 911 ATATGATGGAAGGGTATTCGGACACGTTAATGGTTTGTGATTCTGGGGATCATCAGAAGAGTTTGTCAAT

TM-1(this study) 981 GGAGCCTAGACAAAACTTTAATGTTGGAACAAGTAATGCTAGTAGTTTCGAAGAAAACAAGAATTACTGG
11452GZ 981 GGAGCCTAGACAAAACTTTAATGTTGGAACAAGTAATGCTAGTAGTTTCGAAGAAAACAAGAATTACTGG
TaoGZ 981 GGAGCCTAGACAAAACTTTAATGTTGGAACAAGTAATGCTAGTAGTTTCGAAGAAAACAAGAATTACTGG
Xu142 fl 981 GGAGCCTAGACAAAACTTTAATGTTGGAACAAGTAATGCTAGTAGTTTCGAAGAAAACAAGAATTACTGG
TM-1(CotAD_13206 BGI) 981 GGAGCCTAGACAAAACTTTAATGTTGGAACAAGTAATGCTAGTAGTTTCGAAGAAAACAAGAATTACTGG
TM-1(Gh_D12G1628 NAU) 981 GGAGCCTAGACAAAACTTTAATGTTGGAACAAGTAATGCTAGTAGTTTCGAAGAAAACAAGAATTACTGG
081925 fl 981 GGAGCCTAGACAAAACTTTAATGTTGGAACAAGTAATGCTAGTAGTTTCGAAGAAAACAAGAATTACTGG
Caozao3 981 GGAGCCTAGACAAAACTTTAATGTTGGAACAAGTAATGCTAGTAGTTTCGAAGAAAACAAGAATTACTGG
SL1-7-1 981 GGAGCCTAGACAAAACTTTAATGTTGGAACAAGTAATGCTAGTAGTTTCGAAGAAAACAAGAATTACTGG
SA27 981 GGAGCCTAGACAAAACTTTAATGTTGGAACAAGTAATGCTAGTAGTTTCGAAGAAAACAAGAATTACTGG
Zhousuohongjijiaoye 981 GGAGCCTAGACAAAACTTTAATGTTGGAACAAGTAATGCTAGTAGTTTCGAAGAAAACAAGAATTACTGG
3-79(this study) 981 GGAGCCTAGACAAAACTTTAATGTTGGAACAAGTAATGCTAGTAGTTTCGAAGAAAACAAGAATTACTGG
Hai7124 981 GGAGCCTAGACAAAACTTTAATGTTGGAACAAGTAATGCTAGTAGTTTCGAAGAAAACAAGAATTACTGG
9078N 981 GGAGCCTAGACAAAACTTTAATGTTGGAACAAGTAATGCTAGTAGTTTCGAAGAAAACAAGAATTACTGG
Xinhai18 981 GGAGCCTAGACAAAACTTTAATGTTGGAACAAGTAATGCTAGTAGTTTCGAAGAAAACAAGAATTACTGG
Jizha45 981 GGAGCCTAGACAAAACTTTAATGTTGGAACAAGTAATGCTAGTAGTTTCGAAGAAAACAAGAATTACTGG
gznn1-1 981 GGAGCCTAGACAAAACTTTAATGTTGGAACAAGTAATGCTAGTAGTTTCGAAGAAAACAAGAATTACTGG
MD17 981 GGAGCCTAGACAAAACTTTAATGTTGGAACAAGTAATGCTAGTAGTTTCGAAGAAAACAAGAATTACTGG
n2 981 GGAGCCTAGACAAAACTTTAATGTTGGAACAAGTAATGCTAGTAGTTTCGAAGAAAACAAGAATTACTGG
3-79(Gbscaffold4699.3.0 HZAU) 981 GGAGCCTAGACAAAACTTTAATGTTGGAACAAGTAATGCTAGTAGTTTCGAAGAAAACAAGAATTACTGG
Gorai.008G179600(JGI) 981 GGAGCCTAGACAAAACTTTAATGTTGGAACAAGTAATGCTAGTAGTTTCGAAGAAAACAAGAATTACTGG

TM-1(this study) 1051 AACAACATCCTTAATTTTGCGAATGCTTCCCCATCTGGTTCTTCTGTCTTTTGA
11452GZ 1051 AACAACATCCTTAATTTTGCGAATGCTTCCCCATCTGGTTCTTCTGTCTTTTGA
TaoGZ 1051 AACAACATCCTTAATTTTGCGAATGCTTCCCCATCTGGTTCTTCTGTCTTTTGA

**Xu142 fl**	1051	AACAACATCCTTAATTTTGCGAATGCTTCCCCATCTGGTTCTTCTGTCTTTTGA
**TM-1(CotAD_13206 BGI)**	1051	AACAACATCCTTAATTTTGCGAATGCTTCCCCATCTGGTTCTTCTGTCTTTTGA
**TM-1(Gh_D12G1628 NAU)**	1051	AACAACATCCTTAATTTTGCGAATGCTTCCCCATCTGGTTCTTCTGTCTTTTGA
**081925 fl**	1051	AACAACATCCTTAATTTTGCGAATGCTTCCCCATCTGGTTCTTCTGTCTTTTGA
**Caozao3**	1051	AACAACATCCTTAATTTTGCGAATGCTTCCCCATCTGGTTCTTCTGTCTTTTGA
**SL1-7-1**	1051	AACAACATCCTTAATTTTGCGAATGCTTCCCCATCTGGTTCTTCTGTCTTTTGA
**SA27**	1051	AACAACATCCTTAATTTTGCGAATGCTTCCCCATCTGGTTCTTCTGTCTTTTGA
**Zhousuohongjijiaoye**	1051	AACAACATCCTTAATTTTGCGAATGCTTCCCCATCTGGTTCTTCTGTCTTTTGA
**3-79(this study)**	1051	AACAACATCCTTAATTTTGCGAATGCTTCCCCATCTGGTTCTTCTGTCTTTTGA
**Hai7124**	1051	AACAACATCCTTAATTTTGCGAATGCTTCCCCATCTGGTTCTTCTGTCTTTTGA
**9078N**	1051	AACAACATCCTTAATTTTGCGAATGCTTCCCCATCTGGTTCTTCTGTCTTTTGA
**Xinhai18**	1051	AACAACATCCTTAATTTTGCGAATGCTTCCCCATCTGGTTCTTCTGTCTTTTGA
**Jizha45**	1051	AACAACATCCTTAATTTTGCGAATGCTTCCCCATCTGGTTCTTCTGTCTTTTGA
**gznn1-1**	1051	AACAACATCCTTAATTTTGCGAATGCTTCCCCATCTGGTTCTTCTGTCTTTTGA
**MD17**	1051	AACAACATCCTTAATTTTGCGAATGCTTCCCCATCTGGTTCTTCTGTCTTTTGA
**n2**	1051	AACAACATCCTTAATTTTGCGAATGCTTCCCCATCTGGTTCTTCTGTCTTTTGA
**3-79(Gbscaffold4699.3.0 HZAU)**	1051	AACAACATCCTTAATTTTGCGAATGCTTCCCCTTCTGGTTCTTCTGTCTTTTGA
**Gorai.008G179600(JGI)**	1051	AACAACATCCTTAATTTTGCGAATGCTTCCCCATCTGGTTCTTCTGTCTTTTGA
