# Supplementary material for: A Retrotransposon Insertion in GhMML3_D12 Is Likely Responsible for the Lintless Locus li3 of Tetraploid Cotton
Source: Front Plant Sci. 2020 Nov 26;11:593679. doi: 10.3389/fpls.2020.593679 (PMC7725795; doi:10.3389/fpls.2020.593679)
Supplement: Supplementary file 1 [file Data_Sheet_1.zip › Fig S1-Fig S8 and Table S1-S11/Fig S7.pdf]

Fig. S7. Alignment of the promotor sequences of *MML3_D12* from normal lines and mutants. Normal lines: TM-1; fuzzless mutants: 11452GZ, T586, n2, SA27, Zhousuohongjijiaoye, TaoGZ; Gb lines: 3-79, Hai7124, Xinhai18, Jizha45; fiberless mutants: 081925 fl, MD17, Xu142 fl, SL1-7-1; Ga line: Shixiya1.

```
n2                            1    --------AACTACTTGAT-CCTTGTTTCTTATTTTA-----GCATATTTTATCACACTTCCTCTAATCT
3-79(this study)              1    --------AACTACTTGAT-CCTTGTTTCTTATTTTA-----GCATATTTTATCACACTTCCTCTAATCT
MD17                          1    --------AACTACTTGAT-CCTTGTTTCTTATTTTA-----GCATATTTTATCACACTTCCTCTAATCT
Hai7124                       1    -----TGGAACTACTTGAT-CCTTGTTTCTTATTTTA-----GCATATTTTATCACACTTCCTCTAATCT
Jizha45                       1    --------AACTACTTGAT-CCTTGTTTCTTATTTTA-----GCATATTTTATCACACTTCCTCTAATCT
Xinhai18                      1    --------AACTACTTGAT-CCTTGTTTCTTATTTTA-----GCATATTTTATCACACTTCCTCTAATCT
11452GZ                       1    --------AACTACTTGAT-CCTTGTTTCTTATTTTA-----GCATATTTTATCACACTTCCTCTAATCT
TM-1(BGI)                     1    --------AACTACTTGAT-CCTTGTTTCTTATTTTA-----GCATATTTTATCACACTTCCTCTAATCT
Zhousuohongjijiaoye           1    ---CTTGGAACTACTTGAT-CCTTGTTTCTTATTTTA-----GCATATTTTATCACACTTCCTCTAATCT
TM-1(this study)              1    --------AACTACTTGAT-CCTTGTTTCTTATTTTA-----GCATATTTTATCACACTTCCTCTAATCT
T586                          1    --------AACTACTTGAT-CCTTGTTTCTTATTTTA-----GCATATTTTATCACACTTCCTCTAATCT
SL1-7-1                       1    --------AACTACTTGAT-CCTTGTTTCTTATTTTA-----GCATATTTTATCACACTTCCTCTAATCT
SA27                          1    ------TGGACTACTTGAT-CCTTGTTTCTTATTTTA-----GCATATTTTATCACACTTCCTCTAATCT
081925 fl                     1    --------AACTACTTGAT-CCTTGTTTCTTATTTTA-----GCATATTTTATCACACTTCCTCTAATCT
Xu142 fl                      1    --------AACTACTTGAT-CCTTGTTTCTTATTTTA-----GCATATTTTATCACACTTCCTCTAATCT
TM-1(NAU)                     1    --------AACTACTTGAT-CCTTGTTTCTTATTTTA-----GCATATTTTATCACACTTCCTCTAATCT
Gorai.008G179600.1            1    AACTTGGAAACTACTTGAT-CATTGTTTCTTATTTTA-----GCATCTTTTATCACACTTCCTCTAATCT
Shixiaya1(Ga12G1199.1 CRI)    1    -----------TTTTTGTTGTTTTTTTTCTTTCTTTATATATACATATATTGTTAAAAAAAATTTTATCT

n2                            57   TTGAATCTGTCTATGTTCACTCTTGTTTTGTGCTACAATAGA----------------------------
3-79(this study)              57   TTGAATCTGTCTATGTTCACTCTTGTTTTGTGCTACAATAGA----------------------------
MD17                          57   TTGAATCTGTCTATGTTCACTCTTGTTTTGTGCTACAATAGA----------------------------
Hai7124                       60   TTGAATCTGTCTATGTTCACTCTTGTTTTGTGCTACAATAGA----------------------------
Jizha45                       57   TTGAATCTGTCTATGTTCACTCTTGTTTTGTGCTACAATAGA----------------------------
Xinhai18                      57   TTGAATCTGTCTATGTTCACTCTTGTTTTGTGCTACAATAGA----------------------------
11452GZ                       57   TTGAATCTGTCTATGTTCACTCTTGTTTTGTGCTACAATAGA----------------------------
TM-1(BGI)                     57   TTGAATCTGTCTATGTTCACTCTTGTTTTGTGCTACAATAGA----------------------------
Zhousuohongjijiaoye           62   TTGAATCTGTCTATGTTCACTCTTGTTTTGTGCTACAATAGA----------------------------
TM-1(this study)              57   TTGAATCTGTCTATGTTCACTCTTGTTTTGTGCTACAATAGA----------------------------
T586                          57   TTGAATCTGTCTATGTTCACTCTTGTTTTGTGCTACAATAGA----------------------------
SL1-7-1                       57   TTGAATCTGTCTATGTTCACTCTTGTTTTGTGCTACAATAGA----------------------------
SA27                          59   TTGAATCTGTCTATGTTCACTCTTGTTTTGTGCTACAATAGA----------------------------
081925 fl                     57   TTGAATCTGTCTATGTTCACTCTTGTTTTGTGCTACAATAGA----------------------------
Xu142 fl                      57   TTGAATCTGTCTATGTTCACTCTTGTTTTGTGCTACAATAGA----------------------------
TM-1(NAU)                     57   TTGAATCTGTCTATGTTCACTCTTGTTTTGTGCTACAATAGA----------------------------
Gorai.008G179600.1            65   TTGAATCTGTCTATGTTCACTCCTGTTTTGTGCTACAATAGA----------------------------
Shixiaya1(Ga12G1199.1 CRI)    60   TT---------TCCGGCCACTGTGGCGGTGGCCTGCGACGGGCAGCGGCCAACGATTGACCGGTAATCGG

n2                            98   ------CTT--CTAGAT----------------TTCTCTCATCTTTGAGTAGAATTTTTCTCTTTTACGT
3-79(this study)              98   ------CTT--CTAGAT----------------TTCTCTCATCTTTGAGTAGAATTTTTCTCTTTTACGT
MD17                          98   ------CTT--CTAGAT----------------TTCTCTCATCTTTGAGTAGAATTTTTCTCTTTTACGT
Hai7124                       101  ------CTT--CTAGAT----------------TTCTCTCATCTTTGAGTAGAATTTTTCTCTTTTACGT
Jizha45                       98   ------CTT--CTAGAT----------------TTCTCTCATCTTTGAGTAGAATTTTTCTCTTTTACGT
Xinhai18                      98   ------CTT--CTAGAT----------------TTCTCTCATCTTTGAGTAGAATTTTTCTCTTTTACGT
11452GZ                       98   ------CTT--CTAGAT----------------TTCTCTCATCTTTGAGTAGAACTTTTCTCTTTTACGT
TM-1(BGI)                     98   ------CTT--CTAGAT----------------TTCTCTCATCTTTGAGTAGAACTTTTCTCTTTTACGT
Zhousuohongjijiaoye           103  ------CTT--CTAGAT----------------TTCTCTCATCTTTGAGTAGAACTTTTCTCTTTTACGT
TM-1(this study)              98   ------CTT--CTAGAT----------------TTCTCTCATCTTTGAGTAGAACTTTTCTCTTTTACGT
T586                          98   ------CTT--CTAGAT----------------TTCTCTCATCTTTGAGTAGAACTTTTCTCTTTTACGT
SL1-7-1                       98   ------CTT--CTAGAT----------------TTCTCTCATCTTTGAGTAGAACTTTTCTCTTTTACGT
SA27                          100  ------CTT--CTAGAT----------------TTCTCTCATCTTTGAGTAGAACTTTTCTCTTTTACGT
```

```
081925 fl                          98   ------CTT--CTAGAT----------------TTCTCTCATCTTTGAGTAGAACTTTTCTCTTTTACGT
Xu142 fl                          98   ------CTT--CTAGAT----------------TTCTCTCATCTTTGAGTAGAACTTTTCTCTTTTACGT
TM-1(NAU)                          98   ------CTT--CTAGAT----------------TTCTCTCATCTTTGAGTAGAACTTTTCTCTTTTACGT
Gorai.008G179600.1                 106  ------GTT--CTAGAT----------------TTCTCTCATCTTTGAGTAGAATTTTTCTCTTTTAAGT
Shixiaya1(Ga12G1199.1 CRI)         121  CCCACCCTTGGCCGGATTTCCTTTCCCCCTCCCTTCTCTCTTCTTT-----CCCCCTTCTTCTTTT----

n2                                 145  GAAAGCTAAATTATTTATGGTTATCAT-TACATCTTAAATATTTTTATGGGCTGCTTGCTTCTCTTTTTT
3-79(this study)                   145  GAAAGCTAAATTATTTATGGTTATCAT-TACATCTTAAATATTTTTATGGGCTGCTTGCTTCTCTTTTTT
MD17                               145  GAAAGCTAAATTATTTATGGTTATCAT-TACATCTTAAATATTTTTATGGGCTGCTTGCTTCTCTTTTTT
Hai7124                            148  GAAAGCTAAATTATTTATGGTTATCAT-TACATCTTAAATATTTTTATGGGCTGCTTGCTTCTCTTTTTT
Jizha45                            145  GAAAGCTAAATTATTTATGGTTATCAT-TACATCTTAAATATTTTTATGGGCTGCTTGCTTCTCTTTTTT
Xinhai18                           145  GAAAGCTAAATTATTTATGGTTATCAT-TACATCTTAAATATTTTTATGGGCTGCTTGCTTCTCTTTTTT
11452GZ                            145  GAAAGCTAAATTATTTATGGTTATCAT-TACATCTTAAATATTTTTATGGGCTGCTTGCTTCTCTTTTTT
TM-1(BGI)                          145  GAAAGCTAAATTATTTATGGTTATCAT-TACATCTTAAATATTTTTATGGGCTGCTTGCTTCTCTTTTTT
Zhousuohongjijiaoye                150  GAAAGCTAAATTATTTATGGTTATCAT-TACATCTTAAATATTTTTATGGGCTGCTTGCTTCTCTTTTTT
TM-1(this study)                   145  GAAAGCTAAATTATTTATGGTTATCAT-TACATCTTAAATATTTTTATGGGCTGCTTGCTTCTCTTTTTT
T586                               145  GAAAGCTAAATTATTTATGGTTATCAT-TACATCTTAAATA-TTTTATGGGCTGCTTGCTTCTCTTTTTT
SL1-7-1                            145  GAAAGCTAAATTATTTATGGTTATCAT-TACATCTTAAATA-TTTTATGGGCTGCTTGCTTCTCTTTTTT
SA27                               147  GAAAGCTAAATTATTTATGGTTATCAT-TACATCTTAAATA-TTTTATGGGCTGCTTGCTTCTCTTTTTT
081925 fl                          145  GAAAGCTAAATTATTTATGGTTATCAT-TACATCTTAAATA-TTTTATGGGCTGCTTGCTTCTCTTTTTT
Xu142 fl                          145  GAAAGCTAAATTATTTATGGTTATCAT-TACATCTTAAATA-TTTTATGGGCTGCTTGCTTCTCTTTTTT
TM-1(NAU)                          145  GAAAGCTAAATTATTTATGGTTATCAT-TACATCTTAAATA-TTTTATGGGCTGCTTGCTTCTCTTTTTT
Gorai.008G179600.1                 153  GAAAGCTAAATTATTCATGGTTATCAT-TACATCTTAAATATTTTTATGGGCTGCTTACTTCTCTTTTTT
Shixiaya1(Ga12G1199.1 CRI)         181  ------TAGGTATTTTATATATATTATGTATATATTTTTTAATGCCATTAGTTATATATTTCTTATGTAT

n2                                 214  AGGGTCTTACA-ATCATTGCATTCAATGATGGACA---TCTGAATGCTA----------AGACT-TTACG
3-79(this study)                   214  AGGGTCTTACA-ATCATTGCATTCAATGATGGACA---TCTGAATGCTA----------AGACT-TTACG
MD17                               214  AGGGTCTTACA-ATCATTGCATTCAATGATGGACA---TCTGAATGCTA----------AGACT-TTACG
Hai7124                            217  AGGGTCTTACA-ATCATTGCATTCAATGATGGACA---TCTGAATGCTA----------AGACT-TTACG
Jizha45                            214  AGGGTCTTACA-ATCATTGCATTCAATGATGGACA---TCTGAATGCTA----------AGACT-TTACG
Xinhai18                           214  AGGGTCTTACA-ATCATTGCATTCAATGATGGACA---TCTGAATGCTA----------AGACT-TTACG
11452GZ                            214  AGGGTCTTACA-ATCATTGCATTCAATGATGGACA---TCTGAATGCTA----------AGACT-TTACG
TM-1(BGI)                          214  AGGGTCTTACA-ATCATTGCATTCAATGATGGACA---TCTGAATGCTA----------AGACT-TTACG
Zhousuohongjijiaoye                219  AGGGTCTTACA-ATCATTGCATTCAATGATGGACA---TCTGAATGCTA----------AGACT-TTACG
TM-1(this study)                   214  AGGGTCTTACA-ATCATTGCATTCAATGATGGACA---TCTGAATGCTA----------AGACT-TTACG
T586                               213  AGGGTCTTACA-ATCATTGCATTCAATGATGGACA---TCTGAATGCTA----------AGACT-TTACG
SL1-7-1                            213  AGGGTCTTACA-ATCATTGCATTCAATGATGGACA---TCTGAATGCTA----------AGACT-TTACG
SA27                               215  AGGGTCTTACA-ATCATTGCATTCAATGATGGACA---TCTGAATGCTA----------AGACT-TTACG
081925 fl                          213  AGGGTCTTACA-ATCATTGCATTCAATGATGGACA---TCTGAATGCTA----------AGACT-TTACG
Xu142 fl                          213  AGGGTCTTACA-ATCATTGCATTCAATGATGGACA---TCTGAATGCTA----------AGACT-TTACG
TM-1(NAU)                          213  AGGGTCTTACA-ATCATTGCATTCAATGATGGACA---TCTGAATGCTA----------AGACT-TTACG
Gorai.008G179600.1                 222  AGGGTCTTACA-ATCATTGCATTCAATGATGGACA---TCTGAATGCCA----------AGACT-TTACG
Shixiaya1(Ga12G1199.1 CRI)         246  ATATTCTTAAATACTATTATATATACATATATATATATTTTAAATACCATATTGTGTATATATTATTATT

n2                                 269  GGAATTTTTTAGTCTTGGTCCAACTTTTGTAGTGATGAACTTTATTTGGAAATATATGTCATTCATATAG
3-79(this study)                   269  GGAATTTTTTAGTCTTGGTCCAACTTTTGTAGTGATGAACTTTATTTGGAAATATATGTCATTCATATAG
MD17                               269  GGAATTTTTTAGTCTTGGTCCAACTTTTGTAGTGATGAACTTTATTTGGAAATATATGTCATTCATATAG
Hai7124                            272  GGAATTTTTTAGTCTTGGTCCAACTTTTGTAGTGATGAACTTTATTTGGAAATATATGTCATTCATATAG
Jizha45                            269  GGAATTTTTTAGTCTTGGTCCAACTTTTGTAGTGATGAACTTTATTTGGAAATATATGTCATTCATATAG
Xinhai18                           269  GGAATTTTTTAGTCTTGGTCCAACTTTTGTAGTGATGAACTTTATTTGGAAATATATGTCATTCATATAG
11452GZ                            269  GGAATTTTTTAGTCTTGGTCCAACTTTTGTAGTGATGAACTTTATTTGGAAATATATGTCATTCATATAG
TM-1(BGI)                          269  GGAATTTTTTAGTCTTGGTCCAACTTTTGTAGTGATGAACTTTATTTGGAAATATATGTCATTCATATAG
Zhousuohongjijiaoye                274  GGAATTTTTTAGTCTTGGTCCAACTTTTGTAGTGATGAACTTTATTTGGAAATATATGTCATTCATATAG
TM-1(this study)                   269  GGAATTTTTTAGTCTTGGTCCAACTTTTGTAGTGATGAACTTTATTTGGAAATATATGTCATTCATATAG
T586                               268  GGAATTTTTTAGTCTTGGTCCAACTTTTGTAGTGATGAACTTTATTTGGAAATATATGTCATTCATATAG
SL1-7-1                            268  GGAATTTTTTAGTCTTGGTCCAACTTTTGTAGTGATGAACTTTATTTGGAAATATATGTCATTCATATAG
SA27                               270  GGAATTTTTTAGTCTTGGTCCAACTTTTGTAGTGATGAACTTTATTTGGAAATATATGTCATTCATATAG
081925 fl                          268  GGAATTTTTTAGTCTTGGTCCAACTTTTGTAGTGATGAACTTTATTTGGAAATATATGTCATTCATATAG
```

```
Xu142 fl                        268  GGAATTTTTTAGTCTTGGTCCAACTTTTGTAGTGATGAACTTTATTTGGAAATATATGTCATTCATATAG
TM-1(NAU)                       268  GGAATTTTTTAGTCTTGGTCCAACTTTTGTAGTGATGAACTTTATTTGGAAATATATGTCATTCATATAG
Gorai.008G179600.1              277  GGAAGTTTTTAGTCTTGGTCTAACTTTTGTAGTGATGAACTTTATTTGGAAATATATGACATTCATTTAG
Shixiaya1(Ga12G1199.1 CRI)      316  ACAAATTATTACTATTGCTAGTATTATTATAACTATTA---TTATATTAGTGTATATTTTATGTACATAT

n2                              339  GTTTCAGTAAATAACTTGTTCTACCTCAAGCCTTATGCCTCTCTTCCATTCTCTTTTTATCTTCTCACAG
3-79(this study)                339  GTTTCAGTAAATAACTTGTTCTACCTCAAGCCTTATGCCTCTCTTCCATTCTCTTTTTATCTTCTCACAG
MD17                            339  GTTTCAGTAAATAACTTGTTCTACCTCAAGCCTTATGCCTCTCTTCCATTCTCTTTTTATCTTCTCACAG
Hai7124                         342  GTTTCAGTAAATAACTTGTTCTACCTCAAGCCTTATGCCTCTCTTCCATTCTCTTTTTATCTTCTCACAG
Jizha45                         339  GTTTCAGTAAATAACTTGTTCTACCTCAAGCCTTATGCCTCTCTTCCATTCTCTTTTTATCTTCTCACAG
Xinhai18                        339  GTTTCAGTAAATAACTTGTTCTACCTCAAGCCTTATGCCTCTCTTCCATTCTCTTTTTATCTTCTCACAG
11452GZ                         339  GTTTCAGTAAATAACTTGTTCTACCTCAAGCCTTATGCCTCTCTTCCATTCTCTTTTTATCTTCTCACAG
TM-1(BGI)                       339  GTTTCAGTAAATAACTTGTTCTACCTCAAGCCTTATGCCTCTCTTCCATTCTCTTTTTATCTTCTCACAG
Zhousuohongjijiaoye             344  GTTTCAGTAAATAACTTGTTCTACCTCAAGCCTTATGCCTCTCTTCCATTCTCTTTTTATCTTCTCACAG
TM-1(this study)                339  GTTTCAGTAAATAACTTGTTCTACCTCAAGCCTTATGCCTCTCTTCCATTCTCTTTTTATCTTCTCACAG
T586                            338  GTTTCAGTAAATAACTTGTTCTACCTCAAGCCTTATGCCTCTCTTCCATTCTCTTTTTATCTTCTCACAG
SL1-7-1                         338  GTTTCAGTAAATAACTTGTTCTACCTCAAGCCTTATGCCTCTCTTCCATTCTCTTTTTATCTTCTCACAG
SA27                            340  GTTTCAGTAAATAACTTGTTCTACCTCAAGCCTTATGCCTCTCTTCCATTCTCTTTTTATCTTCTCACAG
081925 fl                       338  GTTTCAGTAAATAACTTGTTCTACCTCAAGCCTTATGCCTCTCTTCCATTCTCTTTTTATCTTCTCACAG
Xu142 fl                        338  GTTTCAGTAAATAACTTGTTCTACCTCAAGCCTTATGCCTCTCTTCCATTCTCTTTTTATCTTCTCACAG
TM-1(NAU)                       338  GTTTCAGTAAATAACTTGTTCTACCTCAAGCCTTATGCCTCTCTTCCATTCTCTTTTTATCTTCTCACAG
Gorai.008G179600.1              347  GTTCCAGTAAATAACTTGTTCTACCTCAAGCCTTATGCCTCTCTTCCATTCTCTTTTTATCTTCTCACAG
Shixiaya1(Ga12G1199.1 CRI)      383  GTATATATATATATATTTTTGTACAT---------------------ATCATTATTTTATATTATTGTTG

n2                              409  CAATTCACAGAATTCAACCCCTGTTTCTTTATTTTCAAAGTACAAGAAGAGTCCAACTAGACGAACCAAG
3-79(this study)                409  CAATTCACAGAATTCAACCCCTGTTTCTTTATTTTCAAAGTACAAGAAGAGTCCAACTAGACGAACCAAG
MD17                            409  CAATTCACAGAATTCAACCCCTGTTTCTTTATTTTCAAAGTACAAGAAGAGTCCAACTAGACGAACCAAG
Hai7124                         412  CAATTCACAGAATTCAACCCCTGTTTCTTTATTTTCAAAGTACAAGAAGAGTCCAACTAGACGAACCAAG
Jizha45                         409  CAATTCACAGAATTCAACCCCTGTTTCTTTATTTTCAAAGTACAAGAAGAGTCCAACTAGACGAACCAAG
Xinhai18                        409  CAATTCACAGAATTCAACCCCTGTTTCTTTATTTTCAAAGTACAAGAAGAGTCCAACTAGACGAACCAAG
11452GZ                         409  CAATTCACAGAATTCAACCCCTGTTTCTTTATTTTCAAAGTACAAGAAGAGTCCAACTAGACGAACCAAG
TM-1(BGI)                       409  CAATTCACAGAATTCAACCCCTGTTTCTTTATTTTCAAAGTACAAGAAGAGTCCAACTAGACGAACCAAG
Zhousuohongjijiaoye             414  CAATTCACAGAATTCAACCCCTGTTTCTTTATTTTCAAAGTACAAGAAGAGTCCAACTAGACGAACCAAG
TM-1(this study)                409  CAATTCACAGAATTCAACCCCTGTTTCTTTATTTTCAAAGTACAAGAAGAGTCCAACTAGACGAACCAAG
T586                            408  CAATTCACAGAATTCAACCCCTGTTTCTTTATTTTCAAAGTACAAGAAGAGTCCAACTAGACGAACCAAG
SL1-7-1                         408  CAATTCACAGAATTCAACCCCTGTTTCTTTATTTTCAAAGTACAAGAAGAGTCCAACTAGACGAACCAAG
SA27                            410  CAATTCACAGAATTCAACCCCTGTTTCTTTATTTTCAAAGTACAAGAAGAGTCCAACTAGACGAACCAAG
081925 fl                       408  CAATTCACAGAATTCAACCCCTGTTTCTTTATTTTCAAAGTACAAGAAGAGTCCAACTAGACGAACCAAG
Xu142 fl                        408  CAATTCACAGAATTCAACCCCTGTTTCTTTATTTTCAAAGTACAAGAAGAGTCCAACTAGACGAACCAAG
TM-1(NAU)                       408  CAATTCACAGAATTCAACCCCTGTTTCTTTATTTTCAAAGTACAAGAAGAGTCCAACTAGACGAACCAAG
Gorai.008G179600.1              417  CAATTCACAGAATTCAACCCCTGTTTCTTTATTTTCAAAGTACAAGAAGAGTCCAACTAGACGAACCAAG
Shixiaya1(Ga12G1199.1 CRI)      432  TAA-------ATTTTTATGTACGTTTCCTAATATTT----------------------------------

n2                              479  AGCAGGTGGGAGCCTTTAGAGGAGAAATCAGTTGACAAATTGGGTTCTCTCAACAATTATGTTGTAAAAT
3-79(this study)                479  AGCAGGTGGGAGCCTTTAGAGGAGAAATCAGTTGACAAATTGGGTTCTCTCAACAATTATGTTGTAAAAT
MD17                            479  AGCAGGTGGGAGCCTTTAGAGGAGAAATCAGTTGACAAATTGGGTTCTCTCAACAATTATGTTGTAAAAT
Hai7124                         482  AGCAGGTGGGAGCCTTTAGAGGAGAAATCAGTTGACAAATTGGGTTCTCTCAACAATTATGTTGTAAAAT
Jizha45                         479  AGCAGGTGGGAGCCTTTAGAGGAGAAATCAGTTGACAAATTGGGTTCTCTCAACAATTATGTTGTAAAAT
Xinhai18                        479  AGCAGGTGGGAGCCTTTAGAGGAGAAATCAGTTGACAAATTGGGTTCTCTCAACAATTATGTTGTAAAAT
11452GZ                         479  AGCAGGTGGGAGCCTTTAGAGGAGAAATCAGTTGACAAATTGGGTTCTCTCAACAATTATGTTGTAAAAT
TM-1(BGI)                       479  AGCAGGTGGGAGCCTTTAGAGGAGAAATCAGTTGACAAATTGGGTTCTCTCAACAATTATGTTGTAAAAT
Zhousuohongjijiaoye             484  AGCAGGTGGGAGCCTTTAGAGGAGAAATCAGTTGACAAATTGGGTTCTCTCAACAATTATGTTGTAAAAT
TM-1(this study)                479  AGCAGGTGGGAGCCTTTAGAGGAGAAATCAGTTGACAAATTGGGTTCTCTCAACAATTATGTTGTAAAAT
T586                            478  AGCAGGTGGGAGCCTTTAGAGGAGAAATCAGTTGACAAATTGGGTTCTCTCAACAATTATGTTGTAAAAT
SL1-7-1                         478  AGCAGGTGGGAGCCTTTAGAGGAGAAATCAGTTGACAAATTGGGTTCTCTCAACAATTATGTTGTAAAAT
SA27                            480  AGCAGGTGGGAGCCTTTAGAGGAGAAATCAGTTGACAAATTGGGTTCTCTCAACAATTATGTTGTAAAAT
081925 fl                       478  AGCAGGTGGGAGCCTTTAGAGGAGAAATCAGTTGACAAATTGGGTTCTCTCAACAATTATGTTGTAAAAT
Xu142 fl                        478  AGCAGGTGGGAGCCTTTAGAGGAGAAATCAGTTGACAAATTGGGTTCTCTCAACAATTATGTTGTAAAAT
```

```
TM-1(NAU)                        478  AGCAGGTGGGAGCCTTTAGAGGAGAAATCAGTTGACAAATTGGGTTCTCTCAACAATTATGTTGTAAAAT
Gorai.008G179600.1               487  AGCAGGTGGGAGCCTTTAGAGGAGAAATCAGTTGACAAATTGGGTTCTCTCAACAATTATGTTGTAAAAT
Shixiaya1(Ga12G1199.1 CRI)       460  ------------TCTTTATTGTAGATATTATTATTATTATTACATACTTTT----ATTATATGCATATAT

n2                               549  ATAGTAGCTGAGTTCCTCTTGATGAAAAGGACAGAAAATTCTTCTTTTATATGTATGTTACAGTTGTGTT
3-79(this study)                 549  ATAGTAGCTGAGTTCCTCTTGATGAAAAGGACAGAAAATTCTTCTTTTATATGTATGTTACAGTTGTGTT
MD17                             549  ATAGTAGCTGAGTTCCTCTTGATGAAAAGGACAGAAAATTCTTCTTTTATATGTATGTTACAGTTGTGTT
Hai7124                          552  ATAGTAGCTGAGTTCCTCTTGATGAAAAGGACAGAAAATTCTTCTTTTATATGTATGTTACAGTTGTGTT
Jizha45                          549  ATAGTAGCTGAGTTCCTCTTGATGAAAAGGACAGAAAATTCTTCTTTTATATGTATGTTACAGTTGTGTT
Xinhai18                         549  ATAGTAGCTGAGTTCCTCTTGATGAAAAGGACAGAAAATTCTTCTTTTATATGTATGTTACAGTTGTGTT
11452GZ                          549  ATAGTAGCTGAGTTCCTCTTGATGAAAAGGACAGAAAATTCTTCTTTTATATGTATGTTACAGTTGTGTT
TM-1(BGI)                        549  ATAGTAGCTGAGTTCCTCTTGATGAAAAGGACAGAAAATTCTTCTTTTATATGTATGTTACAGTTGTGTT
Zhousuohongjijiaoye              554  ATAGTAGCTGAGTTCCTCTTGATGAAAAGGACAGAAAATTCTTCTTTTATATGTATGTTACAGTTGTGTT
TM-1(this study)                 549  ATAGTAGCTGAGTTCCTCTTGATGAAAAGGACAGAAAATTCTTCTTTTATATGTATGTTACAGTTGTGTT
T586                             548  ATAGTAGCTGAGTTCCTCTTGATGAAAAGGACAGAAAATTCTTCTTTTATATGTATGTTACAGTTGTGTT
SL1-7-1                          548  ATAGTAGCTGAGTTCCTCTTGATGAAAAGGACAGAAAATTCTTCTTTTATATGTATGTTACAGTTGTGTT
SA27                             550  ATAGTAGCTGAGTTCCTCTTGATGAAAAGGACAGAAAATTCTTCTTTTATATGTATGTTACAGTTGTGTT
081925 fl                        548  ATAGTAGCTGAGTTCCTCTTGATGAAAAGGACAGAAAATTCTTCTTTTATATGTATGTTACAGTTGTGTT
Xu142 fl                         548  ATAGTAGCTGAGTTCCTCTTGATGAAAAGGACAGAAAATTCTTCTTTTATATGTATGTTACAGTTGTGTT
TM-1(NAU)                        548  ATAGTAGCTGAGTTCCTCTTGATGAAAAGGACAGAAAATTCTTCTTTTATATGTATGTTACAGTTGTGTT
Gorai.008G179600.1               557  ATAGTAGCTAAGTTCCTCTTGATGAAAAGGACAGAAAATTCTTCTTTTATATGTATGTTACAGTTGTGTT
Shixiaya1(Ga12G1199.1 CRI)       515  AT----------------------------ATATATATATGTATTTTTATGTACATATTATTATTTTATA

n2                               619  CTATAATAAATACATGTCCGGTTCTTTTCAGTT-------------TGGTTTGGTTTTTACCGATTTTTT
3-79(this study)                 619  CTATAATAAATACATGTCCGGTTCTTTTCAGTT-------------TGGTTTGGTTTTTACCGATTTTTT
MD17                             619  CTATAATAAATACATGTCCGGTTCTTTTCAGTT-------------TGGTTTGGTTTTTACCGATTTTTT
Hai7124                          622  CTATAATAAATACATGTCCGGTTCTTTTCAGTT-------------TGGTTTGGTTTTTACCGATTTTTT
Jizha45                          619  CTATAATAAATACATGTCCGGTTCTTTTCAGTT-------------TGGTTTGGTTTTTACCGATTTTTT
Xinhai18                         619  CTATAATAAATACATGTCCGGTTCTTTTCAGTT-------------TGGTTTGGTTTTTACCGATTTTTT
11452GZ                          619  CTATAATAAATACATGTCCGGTTCTTTTCAATT-------------TGGTTTGGTTTTTACCGATTTTTT
TM-1(BGI)                        619  CTATAATAAATACATGTCCGGTTCTTTTCAATT-------------TGGTTTGGTTTTTACCGATTTTTT
Zhousuohongjijiaoye              624  CTATAATAAATACATGTCCGGTTCTTTTCAATT-------------TGGTTTGGTTTTTACCGATTTTTT
TM-1(this study)                 619  CTATAATAAATACATGTCCGGTTCTTTTCAATT-------------TGGTTTGGTTTTTACCGATTTTTT
T586                             618  CTATAATAAATACATGTCCAGTTCTTTTCAATT-------------TGGTTTGGTTTTTACCGATTTTTT
SL1-7-1                          618  CTATAATAAATACATGTCCAGTTCTTTTCAATT-------------TGGTTTGGTTTTTACCGATTTTTT
SA27                             620  CTATAATAAATACATGTCCAGTTCTTTTCAATT-------------TGGTTTGGTTTTTACCGATTTTTT
081925 fl                        618  CTATAATAAATACATGTCCAGTTCTTTTCAATT-------------TGGTTTGGTTTTTACCGATTTTTT
Xu142 fl                         618  CTATAATAAATACATGTCCAGTTCTTTTCAATT-------------TGGTTTGGTTTTTACCGATTTTTT
TM-1(NAU)                        618  CTATAATAAATACATGTCCAGTTCTTTTCAATT-------------TGGTTTGGTTTTTACCGATTTTTT
Gorai.008G179600.1               627  CTATAATAAATACATGTCCGGTTCTTTTCAGTT-------------TGGTTTGGTTTTTACCGATTTTTT
Shixiaya1(Ga12G1199.1 CRI)       557  TTATTATTACCAAATATATCATTTTTCCTATTTTATTATTAGTACATGATTTGTTTTT-----ATTTTGT

n2                               676  ATCTGTCGTAGTTTGTACTGATTTGATTCTAGACTATAACTAATCGAACGT--ATATTTGTTATTGATG-
3-79(this study)                 676  ATCTGTCGTAGTTTGTACTGATTTGATTCTAGACTATAACTAATCGAACGT--ATATTTGTTATTGATG-
MD17                             676  ATCTGTCGTAGTTTGTACTGATTTGATTCTAGACTATAACTAATCGAACGT--ATATTTGTTATTGATG-
Hai7124                          679  ATCTGTCGTAGTTTGTACTGATTTGATTCTAGACTATAACTAATCGAACGT--ATATTTGTTATTGATG-
Jizha45                          676  ATCTGTCGTAGTTTGTACTGATTTGATTCTAGACTATAACTAATCGAACGT--ATATTTGTTATTGATG-
Xinhai18                         676  ATCTGTCGTAGTTTGTACTGATTTGATTCTAGACTATAACTAATCGAACGT--ATATTTGTTATTGATG-
11452GZ                          676  ATCTGCCGTAGTTTGTACTGATTTGATTCTAGACTATAACTAATCGAACGT--ATATTTGTTATTGATG-
TM-1(BGI)                        676  ATCTGCCGTAGTTTGTACTGATTTGATTCTAGACTATAACTAATCGAACGT--ATATTTGTTATTGATG-
Zhousuohongjijiaoye              681  ATCTGCCGTAGTTTGTACTGATTTGATTCTAGACTATAACTAATCGAACGT--ATATTTGTTATTGATG-
TM-1(this study)                 676  ATCTGCCGTAGTTTGTACTGATTTGATTCTAGACTATAACTAATCGAACGT--ATATTTGTTATTGATG-
T586                             675  ATCTGCCGTAGTTTGTACTGATTTGATTCTAGACTATAACTAATCGAACGT--ATATTTGTTATTGATG-
SL1-7-1                          675  ATCTGCCGTAGTTTGTACTGATTTGATTCTAGACTATAACTAATCGAACGT--ATATTTGTTATTGATG-
SA27                             677  ATCTGCCGTAGTTTGTACTGATTTGATTCTAGACTATAACTAATCGAACGT--ATATTTGTTATTGATG-
081925 fl                        675  ATCTGCCGTAGTTTGTACTGATTTGATTCTAGACTATAACTAATCGAACGT--ATATTTGTTATTGATG-
Xu142 fl                         675  ATCTGCCGTAGTTTGTACTGATTTGATTCTAGACTATAACTAATCGAACGT--ATATTTGTTATTGATG-
TM-1(NAU)                        675  ATCTGCCGTAGTTTGTACTGATTTGATTCTAGACTATAACTAATCGAACGT--ATATTTGTTATTGATG-
```

```
Gorai.008G179600.1              684  ATCTGTCGTAGTTTGTACTGATTTGATTCTAGACTATAACTAATCGAATGT--ATATTTGTTATTGATG-
Shixiaya1(Ga12G1199.1 CRI)      622  AAATATCATTATTATTGTTATTCTAATATTCTAGTATTCC-------ATGTTAATATTTTTCATTAATGA

n2                              743  TATTGTATTTGTACGGAGGAGTTTTAGATGAATGGTTGGTGTTGATTTATCTATGATTTAT-ATTTTTGA
3-79(this study)                743  TATTGTATTTGTACGGAGGAGTTTTAGATGAATGGTTGGTGTTGATTTATCTATGATTTAT-ATTTTTGA
MD17                            743  TATTGTATTTGTACGGAGGAGTTTTAGATGAATGGTTGGTGTTGATTTATCTATGATTTAT-ATTTTTGA
Hai7124                         746  TATTGTATTTGTACGGAGGAGTTTTAGATGAATGGTTGGTGTTGATTTATCTATGATTTAT-ATTTTTGA
Jizha45                         743  TATTGTATTTGTACGGAGGAGTTTTAGATGAATGGTTGGTGTTGATTTATCTATGATTTAT-ATTTTTGA
Xinhai18                        743  TATTGTATTTGTACGGAGGAGTTTTAGATGAATGGTTGGTGTTGATTTATCTATGATTTAT-ATTTTTGA
11452GZ                         743  TATTGTATTTGTACGGAGGAGTTTTAGATGAATGGTTGGTGTTGATTTATCTATGATTTAT-ATTTTTGA
TM-1(BGI)                       743  TATTGTATTTGTACGGAGGAGTTTTAGATGAATGGTTGGTGTTGATTTATCTATGATTTAT-ATTTTTGA
Zhousuohongjijiaoye             748  TATTGTATTTGTACGGAGGAGTTTTAGATGAATGGTTGGTGTTGATTTATCTATGATTTAT-ATTTTTGA
TM-1(this study)                743  TATTGTATTTGTACGGAGGAGTTTTAGATGAATGGTTGGTGTTGATTTATCTATGATTTAT-ATTTTTGA
T586                            742  TATTGTATTTGTACGGAGGAGTTTTAGATGAATGGTTGGTGTTGATTTATCTATGATTTAT-ATTTTTGA
SL1-7-1                         742  TATTGTATTTGTACGGAGGAGTTTTAGATGAATGGTTGGTGTTGATTTATCTATGATTTAT-ATTTTTGA
SA27                            744  TATTGTATTTGTACGGAGGAGTTTTAGATGAATGGTTGGTGTTGATTTATCTATGATTTAT-ATTTTTGA
081925 fl                       742  TATTGTATTTGTACGGAGGAGTTTTAGATGAATGGTTGGTGTTGATTTATCTATGATTTAT-ATTTTTGA
Xu142 fl                        742  TATTGTATTTGTACGGAGGAGTTTTAGATGAATGGTTGGTGTTGATTTATCTATGATTTAT-ATTTTTGA
TM-1(NAU)                       742  TATTGTATTTGTACGGAGGAGTTTTAGATGAATGGTTGGTGTTGATTTATCTATGATTTAT-ATTTTTGA
Gorai.008G179600.1              751  TATTGTATTTGTACGGAGGAGTTTTAGATGAATGGTTGGTGTTGATTTATCTATGATTTAT-ATTTTTGA
Shixiaya1(Ga12G1199.1 CRI)      685  TATCATTTT------------TTTTG----------CGTTCTTTATCTATTTTTAATTTCTCACTTTAGG

n2                              812  ACTATTTTGTC----AATTTAATTTATATATGTAGTAATAATTGAATACATAAA-TTAACATGACTTTTA
3-79(this study)                812  ACTATTTTGTC----AATTTAATTTATATATGTAGTAATAATTGAATACATAAA-TTAACATGACTTTTA
MD17                            812  ACTATTTTGTC----AATTTAATTTATATATGTAGTAATAATTGAATACATAAA-TTAACATGACTTTTA
Hai7124                         815  ACTATTTTGTC----AATTTAATTTATATATGTAGTAATAATTGAATACATAAA-TTAACATGACTTTTA
Jizha45                         812  ACTATTTTGTC----AATTTAATTTATATATGTAGTAATAATTGAATACATAAA-TTAACATGACTTTTA
Xinhai18                        812  ACTATTTTGTC----AATTTAATTTATATATGTAGTAATAATTGAATACATAAA-TTAACATGACTTTTA
11452GZ                         812  ACTATTTTGTC----AATTTAATTTATATATGTAGTAATAATTGAATACATAAA-TTAACATGACTTTTA
TM-1(BGI)                       812  ACTATTTTGTC----AATTTAATTTATATATGTAGTAATAATTGAATACATAAA-TTAACATGACTTTTA
Zhousuohongjijiaoye             817  ACTATTTTGTC----AATTTAATTTATATATGTAGTAATAATTGAATACATAAA-TTAACATGACTTTTA
TM-1(this study)                812  ACTATTTTGTC----AATTTAATTTATATATGTAGTAATAATTGAATACATAAA-TTAACATGACTTTTA
T586                            811  ACTATTTTGTC----AATTTAATTTATATATGTAGTAATAATTGAATACATAAA-TTAACATGACTTTTA
SL1-7-1                         811  ACTATTTTGTC----AATTTAATTTATATATGTAGTAATAATTGAATACATAAA-TTAACATGACTTTTA
SA27                            813  ACTATTTTGTC----AATTTAATTTATATATGTAGTAATAATTGAATACATAAA-TTAACATGACTTTTA
081925 fl                       811  ACTATTTTGTC----AATTTAATTTATATATGTAGTAATAATTGAATACATAAA-TTAACATGACTTTTA
Xu142 fl                        811  ACTATTTTGTC----AATTTAATTTATATATGTAGTAATAATTGAATACATAAA-TTAACATGACTTTTA
TM-1(NAU)                       811  ACTATTTTGTC----AATTTAATTTATATATGTAGTAATAATTGAATACATAAA-TTAACATGACTTTTA
Gorai.008G179600.1              820  ACTATTTTGTC----AATTTAATTTATATATGTAGTAATAATTGAATACATAAA-TTAACATGACTTTTA
Shixiaya1(Ga12G1199.1 CRI)      733  AATATTTTTTCTCATGTTTTAATTAAT---TTCAAAAATAAGGCAATGTACCGATTTAATATGA-----A

n2                              877  GCGA-CGCTTTTATTAAAAACGCCGCTAAAGAAAATGACATTTAGCGGCGATTGTGGGGAAA-GCGCCAC
3-79(this study)                877  GCGA-CGCTTTTATTAAAAACGCCGCTAAAGAAAATGACATTTAGCGGCGATTGTGGGGAAA-GCGCCAC
MD17                            877  GCGA-CGCTTTTATTAAAAACGCCGCTAAAGAAAATGACATTTAGCGGCGATTGTGGGGAAA-GCGCCAC
Hai7124                         880  GCGA-CGCTTTTATTAAAAACGCCGCTAAAGAAAATGACATTTAGCGGCGATTGTGGGGAAA-GCGCCAC
Jizha45                         877  GCGA-CGCTTTTATTAAAAACGCCGCTAAAGAAAATGACATTTAGCGGCGATTGTGGGGAAA-GCGCCAC
Xinhai18                        877  GCGA-CGCTTTTATTAAAAACGCCGCTAAAGAAAATGACATTTAGCGGCGATTGTGGGGAAA-GCGCCAC
11452GZ                         877  GCGA-CGCTTTTACTAAAAACGCCGCTAAAGAAAATGACATTTAGCGGCGATTGTGGGGAAA-GCGCCAC
TM-1(BGI)                       877  GCGA-CGCTTTTACTAAAAACGCCGCTAAAGAAAATGACATTTAGCGGCGATTGTGGGGAAA-GCGCCAC
Zhousuohongjijiaoye             882  GCGA-CGCTTTTACTAAAAACGCCGCTAAAGAAAATGACATTTAGCGGCGATTGTGGGGAAA-GCGCCAC
TM-1(this study)                877  GCGA-CGCTTTTACTAAAAACGCCGCTAAAGAAAATGACATTTAGCGGCGATTGTGGGGAAA-GCGCCAC
T586                            876  GCGA-CGCTTTTACTAAAAACGCCGCTAAAGAAAATGATATTTAGCGGCGATTGTGGGGAAA-GCGCCAC
SL1-7-1                         876  GCGA-CGCTTTTACTAAAAACGCCGCTAAAGAAAATGATATTTAGCGGCGATTGTGGGGAAA-GCGCCAC
SA27                            878  GCGA-CGCTTTTACTAAAAACGCCGCTAAAGAAAATGATATTTAGCGGCGATTGTGGGGAAA-GCGCCAC
081925 fl                       876  GCGA-CGCTTTTACTAAAAACGCCGCTAAAGAAAATGATATTTAGCGGCGATTGTGGGGAAA-GCGCCAC
Xu142 fl                        876  GCGA-CGCTTTTACTAAAAACGCCGCTAAAGAAAATGATATTTAGCGGCGATTGTGGGGAAA-GCGCCAC
TM-1(NAU)                       876  GCGA-CGCTTTTACTAAAAACGCCGCTAAAGAAAATGATATTTAGCGGCGATTGTGGGGAAA-GCGCCAC
Gorai.008G179600.1              885  GCGA-CGCTTTTACTAAAAACGCCGCTAAAGAAAATGACCTTTAGCGGCATTTGTGGGGAAA--------
```

```
Shixiaya1(Ga12G1199.1 CRI)  795  GCCATCGATTTCAT---------CGCTATGTTGGGTGAAATTCATCGGCTTGTGTTAAAAAACGGGACAC

n2                          945  T----------AAAGGTCATGTTCTATAG-----CGTTTGTTGG-GGAAGCGCTGCTAA-----------
3-79(this study)            945  T----------AAAGGTCATGTTCTATAG-----CGTTTGTTGG-GGAAGCGCTGCTAA-----------
MD17                        945  T----------AAAGGTCATGTTCTATAG-----CGTTTGTTGG-GGAAGCGCTGCTAA-----------
Hai7124                     948  T----------AAAGGTCATGTTCTATAG-----CGTTTGTTGG-GGAAGCGCTGCTAA-----------
Jizha45                     945  T----------AAAGGTCATGTTCTATAG-----CGTTTGTTGG-GGAAGCGCTGCTAA-----------
Xinhai18                    945  T----------AAAGGTCATGTTCTATAG-----CGTTTGTTGG-GGAAGCGCTGCTAA-----------
11452GZ                     945  T----------AAAGGTCATGTTCTATAG-----CGTTTGTTGG-GGAAGCGCTGCTAA-----------
TM-1(BGI)                   945  T----------AAAGGTCATGTTCTATAG-----CGTTTGTTGG-GGAAGCGCTGCTAA-----------
Zhousuohongjijiaoye         950  T----------AAAGGTCATGTTCTATAG-----CGTTTGTTGG-GGAAGCGCTGCTAA-----------
TM-1(this study)            945  T----------AAAGGTCATGTTCTATAG-----CGTTTGTTGG-GGAAGCGCTGCTAA-----------
T586                        944  T----------AAAGGTCATGTTCTATAG-----CGTTTGTTGG-GGAAGCGCTGCTAA-----------
SL1-7-1                     944  T----------AAAGGTCATGTTCTATAG-----CGTTTGTTGG-GGAAGCGCTGCTAA-----------
SA27                        946  T----------AAAGGTCATGTTCTATAG-----CGTTTGTTGG-GGAAGCGCTGCTAA-----------
081925 fl                   944  T----------AAAGGTCATGTTCTATAG-----CGTTTGTTGG-GGAAGCGCTGCTAA-----------
Xu142 fl                    944  T----------AAAGGTCATGTTCTATAG-----CGTTTGTTGG-GGAAGCGCTGCTAA-----------
TM-1(NAU)                   944  T----------AAAGGTCATGTTCTATAG-----CGTTTGTTGG-GGAAGCGCTGCTAA-----------
Gorai.008G179600.1          945  -------------------------------------------------GTGCCGCTAA-----------
Shixiaya1(Ga12G1199.1 CRI)  856  CCTTTCTAAAAAAAAATCGAAAACTAAAAATTCTCATTTTTCAATTGAATCACGATTAAATATTATATTG

n2                          988  AAGTCATGTTCTTTAGCGGC------------GTTTATGGG--------------------GAAAGCGCC
3-79(this study)            988  AAGTCATGTTCTTTAGCGGC------------GTTTATGGG--------------------GAAAGCGCC
MD17                        988  AAGTCATGTTCTTTAGCGGC------------GTTTATGGG--------------------GAAAGCGCC
Hai7124                     991  AAGTCATGTTCTTTAGCGGC------------GTTTATGGG--------------------GAAAGCGCC
Jizha45                     988  AAGTCATGTTCTTTAGCGGC------------GTTTATGGG--------------------GAAAGCGCC
Xinhai18                    988  AAGTCATGTTCTTTAGCGGC------------GTTTATGGG--------------------GAAAGCGCC
11452GZ                     988  AAGTCATGTTCTTTAGCGGC------------GTTTAGGGG--------------------GAAAGCGCC
TM-1(BGI)                   988  AAGTCATGTTCTTTAGCGGC------------GTTTAGGGG--------------------GAAAGCGCC
Zhousuohongjijiaoye         993  AAGTCATGTTCTTTAGCGGC------------GTTTAGGGG--------------------GAAAGCGCC
TM-1(this study)            988  AAGTCATGTTCTTTAGCGGC------------GTTTAGGGG--------------------GAAAGCGCC
T586                        987  AAGTCATGTTCTTTAGCGGC------------GTTTAGGGG--------------------GAAAGCGCC
SL1-7-1                     987  AAGTCATGTTCTTTAGCGGC------------GTTTAGGGG--------------------GAAAGCGCC
SA27                        989  AAGTCATGTTCTTTAGCGGC------------GTTTAGGGG--------------------GAAAGCGCC
081925 fl                   987  AAGTCATGTTCTTTAGCGGC------------GTTTAGGGG--------------------GAAAGCGCC
Xu142 fl                    987  AAGTCATGTTCTTTAGCGGC------------GTTTAGGGG--------------------GAAAGCGCC
TM-1(NAU)                   987  AAGTCATGTTCTTTAGCGGC------------GTTTAGGGG--------------------GAAAGCGCC
Gorai.008G179600.1          956  AGGTCATGTTTTCTAGCGGC------------GTTTATAGG--------------------GAAAGCGCC
Shixiaya1(Ga12G1199.1 CRI)  926  AACTCATATTTTTGAAAATCAAGTCAACACATGTTTATGAGATACCAATTTTGGGCGTCGCGAGGGTGCT

n2                          1026 ACTAA-----------------AGGTCATGTTCTATAGCGTTTGTGGGGG-----AAGCGCTGCTAAAAG
3-79(this study)            1026 ACTAA-----------------AGGTCATGTTCTATAGCGTTTGTGGGGG-----AAGCGCTGCTAAAAG
MD17                        1026 ACTAA-----------------AGGTCATGTTCTATAGCGTTTGTGGGGG-----AAGCGCTGCTAAAAG
Hai7124                     1029 ACTAA-----------------AGGTCATGTTCTATAGCGTTTGTGGGGG-----AAGCGCTGCTAAAAG
Jizha45                     1026 ACTAA-----------------AGGTCATGTTCTATAGCGTTTGTGGGGG-----AAGCGCTGCTAAAAG
Xinhai18                    1026 ACTAA-----------------AGGTCATGTTCTATAGCGTTTGTGGGGG-----AAGCGCTGCTAAAAG
11452GZ                     1026 ACTAA-----------------AGGTCATGTTCTATAGCGTTTGTGGGGG-----AAGCGCTGTTAAAAG
TM-1(BGI)                   1026 ACTAA-----------------AGGTCATGTTCTATAGCGTTTGTGGGGG-----AAGCGCTGTTAAAAG
Zhousuohongjijiaoye         1031 ACTAA-----------------AGGTCATGTTCTATAGCGTTTGTGGGGG-----AAGCGCTGTTAAAAG
TM-1(this study)            1026 ACTAA-----------------AGGTCATGTTCTATAGCGTTTGTGGGGG-----AAGCGCTGTTAAAAG
T586                        1025 ACTAA-----------------AGGTCATGTTCTATAGCGTTTGTGGGGG-----AAGCGCTGTTAAAAG
SL1-7-1                     1025 ACTAA-----------------AGGTCATGTTCTATAGCGTTTGTGGGGG-----AAGCGCTGTTAAAAG
SA27                        1027 ACTAA-----------------AGGTCATGTTCTATAGCGTTTGTGGGGG-----AAGCGCTGTTAAAAG
081925 fl                   1025 ACTAA-----------------AGGTCATGTTCTATAGCGTTTGTGGGGG-----AAGCGCTGTTAAAAG
Xu142 fl                    1025 ACTAA-----------------AGGTCATGTTCTATAGCGTTTGTGGGGG-----AAGCGCTGTTAAAAG
TM-1(NAU)                   1025 ACTAA-----------------AGGTCATGTTCTATAGCGTTTGTGGGGG-----AAGCGCTGTTAAAAG
Gorai.008G179600.1          994  ACTAA-----------------AGGTCATGTTCTATAGCGTTTGTGGGGG-----AAGCGCTGCTAAAAG
Shixiaya1(Ga12G1199.1 CRI)  996  AATAACTTCCTCGCGCGTAACTAACTCCCGAACCCCAATTTTTCTCTGGACTTTCACGTAGACCTAAATT
```

```
n2                             1074 TCATGTTCTTTAGCGGCGTTTGTGGCAATAGTGGTGTTTATGGGTA---AAGCACCACTAAAGGTCATGT
3-79(this study)               1074 TCATGTTCTTTAGCGGCGTTTGTGGCAATAGTGGTGTTTATGGGTA---AAGCACCACTAAAGGTCATGT
MD17                           1074 TCATGTTCTTTAGCGGCGTTTGTGGCAATAGTGGTGTTTATGGGTA---AAGCACCACTAAAGGTCATGT
Hai7124                        1077 TCATGTTCTTTAGCGGCGTTTGTGGCAATAGTGGTGTTTATGGGTA---AAGCACCACTAAAGGTCATGT
Jizha45                        1074 TCATGTTCTTTAGCGGCGTTTGTGGCAATAGTGGTGTTTATGGGTA---AAGCACCACTAAAGGTCATGT
Xinhai18                       1074 TCATGTTCTTTAGCGGCGTTTGTGGCAATAGTGGTGTTTATGGGTA---AAGCACCACTAAAGGTCATGT
11452GZ                        1074 TCATGTTCTTTAGCGGCGTTTGTGGCAATAGTGGTGTTTATGGGTA---AAGCACCACTAAAGGTCATGT
TM-1(BGI)                      1074 TCATGTTCTTTAGCGGCGTTTGTGGCAATAGTGGTGTTTATGGGTA---AAGCACCACTAAAGGTCATGT
Zhousuohongjijiaoye            1079 TCATGTTCTTTAGCGGCGTTTGTGGCAATAGTGGTGTTTATGGGTA---AAGCACCACTAAAGGTCATGT
TM-1(this study)               1074 TCATGTTCTTTAGCGGCGTTTGTGGCAATAGTGGTGTTTATGGGTA---AAGCACCACTAAAGGTCATGT
T586                           1073 TCATGTTCTTTAGCGGCGTTTGTGGCAATAGTGGTGTTTATGGGTA---AAGCACCACTAAAGGTCATGT
SL1-7-1                        1073 TCATGTTCTTTAGCGGCGTTTGTGGCAATAGTGGTGTTTATGGGTA---AAGCACCACTAAAGGTCATGT
SA27                           1075 TCATGTTCTTTAGCGGCGTTTGTGGCAATAGTGGTGTTTATGGGTA---AAGCACCACTAAAGGTCATGT
081925 fl                      1073 TCATGTTCTTTAGCGGCGTTTGTGGCAATAGTGGTGTTTATGGGTA---AAGCACCACTAAAGGTCATGT
Xu142 fl                       1073 TCATGTTCTTTAGCGGCGTTTGTGGCAATAGTGGTGTTTATGGGTA---AAGCACCACTAAAGGTCATGT
TM-1(NAU)                      1073 TCATGTTCTTTAGCGGCGTTTGTGGCAATAGTGGTGTTTATGGGTA---AAGCACCACTAAAGGTCATGT
Gorai.008G179600.1             1042 TCATGTTCTTTAGTGGCGTTTGTGGCAATAGTAGTGTTTATGGGTA---AAGCACCGCTAAAGGTCATGT
Shixiaya1(Ga12G1199.1 CRI)     1066 TAGCCTTCTT--------TTTGTTTTAA-AATAATTTTATTAGGTGTCCGATCACAACTATAAAAAAGGA

n2                             1141 TTTATACCGGCGTTTTTCCACATAAACGCCGCAAA-------------ATTTAATGG--CGTTATTTATA
3-79(this study)               1141 TTTATACCGGCGTTTTTCCACATAAACGCCGCAAA-------------ATTTAATGG--CGTTATTTATA
MD17                           1141 TTTATACCGGCGTTTTTCCACATAAACGCCGCAAA-------------ATTTAATGG--CGTTATTTATA
Hai7124                        1144 TTTATACCGGCGTTTTTCCACATAAACGCCGCAAA-------------ATTTAATGG--CGTTATTTATA
Jizha45                        1141 TTTATACCGGCGTTTTTCCACATAAACGCCGCAAA-------------ATTTAATGG--CGTTATTTATA
Xinhai18                       1141 TTTATACCGGCGTTTTTCCACATAAACGCCGCAAA-------------ATTTAATGG--CGTTATTTATA
11452GZ                        1141 TTTATACCGGCGTTTTTCCACATAAACGCCGCAAA-------------ATTTAATGG--CGTTATTTATA
TM-1(BGI)                      1141 TTTATACCGGCGTTTTTCCACATAAACGCCGCAAA-------------ATTTAATGG--CGTTATTTATA
Zhousuohongjijiaoye            1146 TTTATACCGGCGTTTTTCCACATAAACGCCGCAAA-------------ATTTAATGG--CGTTATTTATA
TM-1(this study)               1141 TTTATACCGGCGTTTTTCCACATAAACGCCGCAAA-------------ATTTAATGG--CGTTATTTATA
T586                           1140 TTTATACCGGCGTTTTTCCACATAAACGCCGCAAA-------------ATTTAATGG--CGTTATTTATA
SL1-7-1                        1140 TTTATACCGGCGTTTTTCCACATAAACGCCGCAAA-------------ATTTAATGG--CGTTATTTATA
SA27                           1142 TTTATACCGGCGTTTTTCCACATAAACGCCGCAAA-------------ATTTAATGG--CGTTATTTATA
081925 fl                      1140 TTTATACCGGCGTTTTTCCACATAAACGCCGCAAA-------------ATTTAATGG--CGTTATTTATA
Xu142 fl                       1140 TTTATACCGGCGTTTTTCCACATAAACGCCGCAAA-------------ATTTAATGG--CGTTATTTATA
TM-1(NAU)                      1140 TTTATACCGGCGTTTTTCCACATAAACGCCGCAAA-------------ATTTAATGG--CGTTATTTATA
Gorai.008G179600.1             1109 TCTGTACCGGCGTTTTTCCACATAAACGCCGCAAA-------------ATTTAGCGG--CGTTATTTATA
Shixiaya1(Ga12G1199.1 CRI)     1127 TCGGTGGCGACTCCCTTTGTTAATAAAATCGAAAGTTTGTTTTCAAATGTTTAATAAATCGCCACAATTA

n2                             1196 GCGGC----------------GTTTTTTACGGCGCTTATCAAAACGCCGCAAATATTTTAGCGGCGCTTA
3-79(this study)               1196 GCGGC----------------GTTTTTTACGGCGCTTATCAAAACGCCGCAAATATTTTAGCGGCGCTTA
MD17                           1196 GCGGC----------------GTTTTTTACGGCGCTTATCAAAACGCCGCAAATATTTTAGCGGCGCTTA
Hai7124                        1199 GCGGC----------------GTTTTTTACGGCGCTTATCAAAACGCCGCAAATATTTTAGCGGCGCTTA
Jizha45                        1196 GCGGC----------------GTTTTTTACGGCGCTTATCAAAACGCCGCAAATATTTTAGCGGCGCTTA
Xinhai18                       1196 GCGGC----------------GTTTTTTACGGCGCTTATCAAAACGCCGCAAATATTTTAGCGGCGCTTA
11452GZ                        1196 GCGGC----------------GTTTTTTACGACGCTTATCAAAACGCCGCAAATATTTTAGCGGCGCTTA
TM-1(BGI)                      1196 GCGGC----------------GTTTTTTACGACGCTTATCAAAACGCCGCAAATATTTTAGCGGCGCTTA
Zhousuohongjijiaoye            1201 GCGGC----------------GTTTTTTACGACGCTTATCAAAACGCCGCAAATATTTTAGCGGCGCTTA
TM-1(this study)               1196 GCGGC----------------GTTTTTTACGACGCTTATCAAAACGCCGCAAATATTTTAGCGGCGCTTA
T586                           1195 GCGGC----------------GTTTTTTACGACGCTTATCAAAACGCCGCAAATATTTTAGCGGCGCTTA
SL1-7-1                        1195 GCGGC----------------GTTTTTTACGACGCTTATCAAAACGCCGCAAATATTTTAGCGGCGCTTA
SA27                           1197 GCGGC----------------GTTTTTTACGACGCTTATCAAAACGCCGCAAATATTTTAGCGGCGCTTA
081925 fl                      1195 GCGGC----------------GTTTTTTACGACGCTTATCAAAACGCCGCAAATATTTTAGCGGCGCTTA
Xu142 fl                       1195 GCGGC----------------GTTTTTTACGACGCTTATCAAAACGCCGCAAATATTTTAGCGGCGCTTA
TM-1(NAU)                      1195 GCGGC----------------GTTTTTTACGACGCTTATCAAAACGCCGCAAATATTTTAGCGGCGCTTA
Gorai.008G179600.1             1164 GCGGC----------------GTTTTTTACGGCGCTTATCAAAACGCCGCAAATGTTTTAGCGGCGCTTA
Shixiaya1(Ga12G1199.1 CRI)     1197 GCGACCAAGCAAAACAAAAATTTTTTTTACGTCGC-TACAAGAACATT-TAAATATTTT---GCTATTTA
```

```
n2                               1250 TAGA---AAAAA-----------------AATGCCGCTAAA-----AACCTG------TTTTGC------
3-79(this study)                 1250 TAGA---AAAAA-----------------AATGCCGCTAAA-----AACCTG------TTTTGC------
MD17                             1250 TAGA---AAAAA-----------------AATGCCGCTAAA-----AACCTG------TTTTGC------
Hai7124                          1253 TAGG--AAAAAA-----------------AATGCCGCTAAA-----AACCTG------TTTTGC------
Jizha45                          1250 TAGA---AAAAA-----------------AATGCCGCTAAA-----AACCTG------TTTTGC------
Xinhai18                         1250 TAGA---AAAAA-----------------AATGCCGCTAAA-----AACCTG------TTTTGC------
11452GZ                          1250 TAGG---AAAAA-----------------AACGCCGCTAAA-----AACCTG------TTTTGCTGTAG-
TM-1(BGI)                        1250 TAGG---AAAAA-----------------AACGCCGCTAAA-----AACCTG------TTTTGCTGTAG-
Zhousuohongjijiaoye              1255 TAGG---AAAAA-----------------AACGCCGCTAAA-----AACCTG------TTTTGCTGTAG-
TM-1(this study)                 1250 TAGG---AAAAA-----------------AACGCCGCTAAA-----AACCTG------TTTTGCTGTAG-
T586                             1249 TAGG---AAAAA-----------------AACGCCGCTAAA-----AACCTG------TTTTGCTGTAG-
SL1-7-1                          1249 TAGG---AAAAA-----------------AACGCCGCTAAA-----AACCTG------TTTTGCTGTAG-
SA27                             1251 TAGG---AAAAA-----------------AACGCCGCTAAA-----AACCTG------TTTTGCTGTAG-
081925 fl                        1249 TAGG---AAAAA-----------------AACGCCGCTAAA-----AACCTG------TTTTGCTGTAG-
Xu142 fl                         1249 TAGG---AAAAA-----------------AACGCCGCTAAA-----AACCTG------TTTTGCTGTAG-
TM-1(NAU)                        1249 TAGG---AAAAA-----------------AACGCCGCTAAA-----AACCTG------TTTTGCTGTAG-
Gorai.008G179600.1               1218 TAGGCCAAAAAA-----------------AACGCCGCTAAA-----AACCTG------TTTTGCTGTAG-
Shixiaya1(Ga12G1199.1 CRI)       1262 TATATTTAAAAACAATAAAAAATCATTTTAACCTCAATAAATTTATAATTTGATATCTTTTTATTACAAC

n2                               1282 --TGATATAA-TTTTTTTTATTCTACCCTTAATCATAAATATCAATTTTATTTAAACTAAAAAAAGTAAG
3-79(this study)                 1282 --TGATATAA-TTTTTTTTATTCTACCCTTAATCATAAATATCAATTTTATTTAAACTAAAAAAAGTAAG
MD17                             1282 --TGATATAA-TTTTTTTTATTCTACCCTTAATCATAAATATCAATTTTATTTAAACTAAAAAAAGTAAG
Hai7124                          1286 --TGATATAA-TTTTTTTTATTCTACCCTTAATCATAAATATCAATTTTATTTAAACTAAAAAAAGTAAG
Jizha45                          1282 --TGATATAA-TTTTTTTTATTCTACCCTTAATCATAAATATCAATTTTATTTAAACTAAAAAAAGTAAG
Xinhai18                         1282 --TGATATAA-TTTTTTTTATTCTACCCTTAATCATAAATATCAATTTTATTTAAACTAAAAAAAGTAAG
11452GZ                          1287 --TGATATAATTTTTTTTTATTCTACCCTTAATCATAAATATCAATTTTATTTAAACTAAAAAAAGTAAG
TM-1(BGI)                        1287 --TGATATAATTTTTTTTTATTCTACCCTTAATCATAAATATCAATTTTATTTAAACTAAAAAAAGTAAG
Zhousuohongjijiaoye              1292 --TGATATAATTTTTTTTTATTCTACCCTTAATCATAAATATCAATTTTATTTAAACTAAAAAAAGTAAG
TM-1(this study)                 1287 --TGATATAATTTTTTTTTATTCTACCCTTAATCATAAATATCAATTTTATTTAAACTAAAAAAAGTAAG
T586                             1286 --TGATATAATTTTTTTTTATTCTACCCTTAATCATAAATATCAATTTTATTTAAACTAAAAAAAGTAAG
SL1-7-1                          1286 --TGATATAATTTTTTTTTATTCTACCCTTAATCATAAATATCAATTTTATTTAAACTAAAAAAAGTAAG
SA27                             1288 --TGATATAATTTTTTTTTATTCTACCCTTAATCATAAATATCAATTTTATTTAAACTAAAAAAAGTAAG
081925 fl                        1286 --TGATATAATTTTTTTTTATTCTACCCTTAATCATAAATATCAATTTTATTTAAACTAAAAAAAGTAAG
Xu142 fl                         1286 --TGATATAATTTTTTTTTATTCTACCCTTAATCATAAATATCAATTTTATTTAAACTAAAAAAAGTAAG
TM-1(NAU)                        1286 --TGATATAATTTTTTTTTATTCTACCCTTAATCATAAATATCAATTTTATTTAAACTAAAAAAAGTAAG
Gorai.008G179600.1               1258 --TGATATAA-TTTTTTTTATTCTACCCTTAATCATAAATATCAATTTTATTTAAACTAAAAAAAGTAAG
Shixiaya1(Ga12G1199.1 CRI)       1332 CTCGTTATAA-TTTTTTTTACTCTATCCTTTATCATAAATATC---------------------------

n2                               1350 GGAAGTTGAGTATGTATTGCTTTCTTTTAAAAGTTAATTTTTATTTATTTATCCCAATTCAAGAGAAGTT
3-79(this study)                 1350 GGAAGTTGAGTATGTATTGCTTTCTTTTAAAAGTTAATTTTTATTTATTTATCCCAATTCAAGAGAAGTT
MD17                             1350 GGAAGTTGAGTATGTATTGCTTTCTTTTAAAAGTTAATTTTTATTTATTTATCCCAATTCAAGAGAAGTT
Hai7124                          1354 GGAAGTTGAGTATGTATTGCTTTCTTTTAAAAGTTAATTTTTATTTATTTATCCCAATTCAAGAGAAGTT
Jizha45                          1350 GGAAGTTGAGTATGTATTGCTTTCTTTTAAAAGTTAATTTTTATTTATTTATCCCAATTCAAGAGAAGTT
Xinhai18                         1350 GGAAGTTGAGTATGTATTGCTTTCTTTTAAAAGTTAATTTTTATTTATTTATCCCAATTCAAGAGAAGTT
11452GZ                          1356 GGAAGTTGAGTATCTATTGCTTTCTTTTAAAAGTTAATTTTTATTTATTTATCCCAATTCAAGAGAAGTT
TM-1(BGI)                        1356 GGAAGTTGAGTATCTATTGCTTTCTTTTAAAAGTTAATTTTTATTTATTTATCCCAATTCAAGAGAAGTT
Zhousuohongjijiaoye              1361 GGAAGTTGAGTATCTATTGCTTTCTTTTAAAAGTTAATTTTTATTTATTTATCCCAATTCAAGAGAAGTT
TM-1(this study)                 1356 GGAAGTTGAGTATCTATTGCTTTCTTTTAAAAGTTAATTTTTATTTATTTATCCCAATTCAAGAGAAGTT
T586                             1355 GGAAGTTGAGTATCTATTGCTTTCTTTTAAAAGTTAATTTTTATTTATTTATCCCAATTCAAGAGAAGTT
SL1-7-1                          1355 GGAAGTTGAGTATCTATTGCTTTCTTTTAAAAGTTAATTTTTATTTATTTATCCCAATTCAAGAGAAGTT
SA27                             1357 GGAAGTTGAGTATCTATTGCTTTCTTTTAAAAGTTAATTTTTATTTATTTATCCCAATTCAAGAGAAGTT
081925 fl                        1355 GGAAGTTGAGTATCTATTGCTTTCTTTTAAAAGTTAATTTTTATTTATTTATCCCAATTCAAGAGAAGTT
Xu142 fl                         1355 GGAAGTTGAGTATCTATTGCTTTCTTTTAAAAGTTAATTTTTATTTATTTATCCCAATTCAAGAGAAGTT
TM-1(NAU)                        1355 GGAAGTTGAGTATCTATTGCTTTCTTTTAAAAGTTAATTTTTATTTATTTATCCCAATTCAAGAGAAGTT
Gorai.008G179600.1               1326 GGAAGTTGAGTATCTCTTGCTTTCTTTTAAAAGTTAATTTTTATTTATTTATCCCAATTCGAGAGAAGTT
Shixiaya1(Ga12G1199.1 CRI)       1373 --AAATTGAGTATCTCTTGCTTTCCTTGAAAAGTTAAATTT--TTTATTTATCCCGATTCAAGAGAAGTT

n2                               1420 AAAGAAAGG-GAAAAAAAAGGAAATCAAAACAAAACAATTTTTAAGATGTAAAATGTCTTTTTTGTCTAC
```

```
3-79(this study)                1420 AAAGAAAGG-GAAAAAAAAGGAAATCAAAACAAAACAATTTTTAAGATGTAAAATGTCTTTTTTGTCTAC
MD17                            1420 AAAGAAAGG-GAAAAAAAAGGAAATCAAAACAAAACAATTTTTAAGATGTAAAATGTCTTTTTTGTCTAC
Hai7124                         1424 AAAGAAAGG-GAAAAAAAAGGAAATCAAAACAAAACAATTTTTAAGATGTAAAATGTCTTTTTTGTCTAC
Jizha45                         1420 AAAGAAAGG-GAAAAAAAAGGAAATCAAAACAAAACAATTTTTAAGATGTAAAATGTCTTTTTTGTCTAC
Xinhai18                        1420 AAAGAAAGG-GAAAAAAAAGGAAATCAAAACAAAACAATTTTTAAGATGTAAAATGTCTTTTTTGTCTAC
11452GZ                         1426 AAAGAAAGG-GAAAAAAAAGGAAATCAAAACAAAACAATTTTTAAGATGTAAAATGTCTTTTTTGTCTAC
TM-1(BGI)                       1426 AAAGAAAGG-GAAAAAAAAGGAAATCAAAACAAAACAATTTTTAAGATGTAAAATGTCTTTTTTGTCTAC
Zhousuohongjijiaoye             1431 AAAGAAAGG-GAAAAAAAAGGAAATCAAAACAAAACAATTTTTAAGATGTAAAATGTCTTTTTTGTCTAC
TM-1(this study)                1426 AAAGAAAGG-GAAAAAAAAGGAAATCAAAACAAAACAATTTTTAAGATGTAAAATGTCTTTTTTGTCTAC
T586                            1425 AAAGAAAGG-GAAAAAAAAGGAAATCAAAACAAAACAATTTTTAAGATGTAAAATGTCTTTTTTGTCTAC
SL1-7-1                         1425 AAAGAAAGG-GAAAAAAAAGGAAATCAAAACAAAACAATTTTTAAGATGTAAAATGTCTTTTTTGTCTAC
SA27                            1427 AAAGAAAGG-GAAAAAAAAGGAAATCAAAACAAAACAATTTTTAAGATGTAAAATGTCTTTTTTGTCTAC
081925 f1                       1425 AAAGAAAGG-GAAAAAAAAGGAAATCAAAACAAAACAATTTTTAAGATGTAAAATGTCTTTTTTGTCTAC
Xu142 f1                        1425 AAAGAAAGG-GAAAAAAAAGGAAATCAAAACAAAACAATTTTTAAGATGTAAAATGTCTTTTTTGTCTAC
TM-1(NAU)                       1425 AAAGAAAGG-GAAAAAAAAGGAAATCAAAACAAAACAATTTTTAAGATGTAAAATGTCTTTTTTGTCTAC
Gorai.008G179600.1              1396 AAAGAAAGGAAAAAAAAAAGGAAATCTAAACAAAACAATTTTTAAGATGTAAAATGTCTTTTTTGTCGAC
Shixiaya1(Ga12G1199.1 CRI)      1440 AAAGAAAGG-----AAAAAGAAAATCAAAACAAAACAACTTTTAAGATGTATAATGTC-TTTTTGTCAAC

n2                              1488 -----TTTTTTTTTATAAATAATTTTATTATGATGTTATAATTGTTAAAACTTTATTATGAACAATCA--
3-79(this study)                1488 -----TTTTTTTTTATAAATAATTTTATTATGATGTTATAATTGTTAAAACTTTATTATGAACAATCA--
MD17                            1488 -----TTTTTTTTTATAAATAATTTTATTATGATGTTATAATTGTTAAAACTTTATTATGAACAATCA--
Hai7124                         1492 -----TTTTTTTTTATAAATAATTTTATTATGATGTTATAATTGTTAAAACTTTATTATGAACAATCA--
Jizha45                         1488 -----TTTTTTTTTATAAATAATTTTATTATGATGTTATAATTGTTAAAACTTTATTATGAACAATCA--
Xinhai18                        1488 -----TTTTTTTTTATAAATAATTTTATTATGATGTTATAATTGTTAAAACTTTATTATGAACAATCA--
11452GZ                         1494 -----TTTTTTTTTATAAATAATTTTATTATGATGTTATAATTGTTAAAACTTTATTATGAACAATCA--
TM-1(BGI)                       1494 -----TTTTTTTTTATAAATAATTTTATTATGATGTTATAATTGTTAAAACTTTATTATGAACAATCA--
Zhousuohongjijiaoye             1499 -----TTTTTTTTTATAAATAATTTTATTATGATGTTATAATTGTTAAAACTTTATTATGAACAATCA--
TM-1(this study)                1494 -----TTTTTTTTTATAAATAATTTTATTATGATGTTATAATTGTTAAAACTTTATTATGAACAATCA--
T586                            1493 -----TTTTTTTTTATAAATAATTTTATTATGATGTTATAATTGTTAAAACTTTATTATGAACAATCA--
SL1-7-1                         1493 -----TTTTTTTTTATAAATAATTTTATTATGATGTTATAATTGTTAAAACTTTATTATGAACAATCA--
SA27                            1495 -----TTTTTTTTTATAAATAATTTTATTATGATGTTATAATTGTTAAAACTTTATTATGAACAATCA--
081925 f1                       1493 -----TTTTTTTTTATAAATAATTTTATTATGATGTTATAATTGTTAAAACTTTATTATGAACAATCA--
Xu142 f1                        1493 -----TTTTTTTTTATAAATAATTTTATTATGATGTTATAATTGTTAAAACTTTATTATGAACAATCA--
TM-1(NAU)                       1493 -----TTTTTTTTTATAAATAATTTTATTATGATGTTATAATTGTTAAAACTTTATTATGAACAATCA--
Gorai.008G179600.1              1465 -----TTTTTTTTTATAAATAATTTTATTATGATGTTATAATTG-TAAAACTTTATTATGAACAATCA--
Shixiaya1(Ga12G1199.1 CRI)      1504 TTTTTTTTTTTTTTATAAATAATTTTATGATGACGTTATAATTG-TAAAACTTTATTATGAACAATCATT

n2                              1551 -CCTTTGCATATTCTATCCAAACAAAATAAATAAAATTACTTAATTTTCTTTTCCTTCACTACTTTTTAG
3-79(this study)                1551 -CCTTTGCATATTCTATCCAAACAAAATAAATAAAATTACTTAATTTTCTTTTCCTTCACTACTTTTTAG
MD17                            1551 -CCTTTGCATATTCTATCCAAACAAAATAAATAAAATTACTTAATTTTCTTTTCCTTCACTACTTTTTAG
Hai7124                         1555 -CCTTTGCATATTCTATCCAAACAAAATAAATAAAATTACTTAATTTTCTTTTCCTTCACTACTTTTTAG
Jizha45                         1551 -CCTTTGCATATTCTATCCAAACAAAATAAATAAAATTACTTAATTTTCTTTTCCTTCACTACTTTTTAG
Xinhai18                        1551 -CCTTTGCATATTCTATCCAAACAAAATAAATAAAATTACTTAATTTTCTTTTCCTTCACTACTTTTTAG
11452GZ                         1557 -CCTTTGCATATTCTATCCAAACAAAATAAATAAAATTACTTAATTTTCTTTTCCTTCACTACTTTTTAG
TM-1(BGI)                       1557 -CCTTTGCATATTCTATCCAAACAAAATAAATAAAATTACTTAATTTTCTTTTCCTTCACTACTTTTTAG
Zhousuohongjijiaoye             1562 -CCTTTGCATATTCTATCCAAACAAAATAAATAAAATTACTTAATTTTCTTTTCCTTCACTACTTTTTAG
TM-1(this study)                1557 -CCTTTGCATATTCTATCCAAACAAAATAAATAAAATTACTTAATTTTCTTTTCCTTCACTACTTTTTAG
T586                            1556 -CCTTTGCATATTCTATCCAAACAAAATAAATAAAATTACTTAATTTTCTTTTCCTTCACTACTTTTTAG
SL1-7-1                         1556 -CCTTTGCATATTCTATCCAAACAAAATAAATAAAATTACTTAATTTTCTTTTCCTTCACTACTTTTTAG
SA27                            1558 -CCTTTGCATATTCTATCCAAACAAAATAAATAAAATTACTTAATTTTCTTTTCCTTCACTACTTTTTAG
081925 f1                       1556 -CCTTTGCATATTCTATCCAAACAAAATAAATAAAATTACTTAATTTTCTTTTCCTTCACTACTTTTTAG
Xu142 f1                        1556 -CCTTTGCATATTCTATCCAAACAAAATAAATAAAATTACTTAATTTTCTTTTCCTTCACTACTTTTTAG
TM-1(NAU)                       1556 -CCTTTGCATATTCTATCCAAACAAAATAAATAAAATTACTTAATTTTCTTTTCCTTCACTACTTTTTAG
Gorai.008G179600.1              1527 -CCTTTGCATATTCTATCCAAACAAAATAAATAAAATTACTTAATTTTCTTTTCCTTCACTACTTTTTAG
Shixiaya1(Ga12G1199.1 CRI)      1573 TCCTTTGCATATTCTATCCAAACAAAATAAATAAAATTACTTAATTTTCTTTTCCTTCACTAATTTTTAG
```

7- bp target site duplication

n2	1621	TGTTTAAATAAGATAATAAATATTATTTACTTTATTTTTTCTTTGCTTTCCTATCTTTAATTCAAACATA
3-79(this study)	1621	TGTTTAAATAAGATAATAAATATTATTTACTTTATTTTTTCTTTGCTTTCCTATCTTTAATTCAAACATA
MD17	1621	TGTTTAAATAAGATAATAAATATTATTTACTTTATTTTTTCTTTGCTTTCCTATCTTTAATTCAAACATA
Hai7124	1625	TGTTTAAATAAGATAATAAATATTATTTACTTTATTTTTTCTTTGCTTTCCTATCTTTAATTCAAACATA
Jizha45	1621	TGTTTAAATAAGATAATAAATATTATTTACTTTATTTTTTCTTTGCTTTCCTATCTTTAATTCAAACATA
Xinhai18	1621	TGTTTAAATAAGATAATAAATATTATTTACTTTATTTTTTCTTTGCTTTCCTATCTTTAATTCAAACATA
11452GZ	1627	TGTTTAAATAAGATAATAAATATTATTTACTTTATTTTTTCTTTGCTTTCCTATCTTTAATTCACACATA
TM-1(BGI)	1627	TGTTTAAATAAGATAATAAATATTATTTACTTTATTTTTTCTTTGCTTTCCTATCTTTAATTCACACATA
Zhousuohongjijiaoye	1632	TGTTTAAATAAGATAATAAATATTATTTACTTTATTTTTTCTTTGCTTTCCTATCTTTAATTCACACATA
TM-1(this study)	1627	TGTTTAAATAAGATAATAAATATTATTTACTTTATTTTTTCTTTGCTTTCCTATCTTTAATTCACACATA
T586	1626	TGTTTAAATAAGATAATAAATATTATTTACTTTATTTTTTCTTTGCTTTCCTATCTTTAATTCACACATA
SL1-7-1	1626	TGTTTAAATAAGATAATAAATATTATTTACTTTATTTTTTCTTTGCTTTCCTATCTTTAATTCACACATA
SA27	1628	TGTTTAAATAAGATAATAAATATTATTTACTTTATTTTTTCTTTGCTTTCCTATCTTTAATTCACACATA
081925 fl	1626	TGTTTAAATAAGATAATAAATATTATTTACTTTATTTTTTCTTTGCTTTCCTATCTTTAATTCACACATA
Xu142 fl	1626	TGTTTAAATAAGATAATAAATATTATTTACTTTATTTTTTCTTTGCTTTCCTATCTTTAATTCACACATA
TM-1(NAU)	1626	TGTTTAAATAAGATAATAAATATTATTTACTTTATTTTTTCTTTGCTTTCCTATCTTTAATTCACACATA
Gorai.008G179600.1	1597	TGTTTAAATAAGATAATAAATATTATTTACTTTCTTTTTTCTTTGCTTTCCTATCTTTAATTCAA-----
Shixiaya1(Ga12G1199.1 CRI)	1643	TGTTTAAATAAGATAATAAATATTTTTTACTTTCTTTTTTCTTTGCTTTCCTATCTTTAATTCAA-----

n2	1691	GTAGAGGTGTTTATGGGTTGGGCGGCTCGGCCCGGCCCAATAGCCCGCCGAAAATATGGAAGGATTTTGG
3-79(this study)	1691	GTAGAGGTGTTTATGGGTTGGGCGGCTCGGCCCGGCCCAATAGCCCGCCGAAAATATGGAAGGATTTTGG
MD17	1691	GTAGAGGTGTTTATGGGTTGGGCGGCTCGGCCCGGCCCAATAGCCCGCCGAAAATATGGAAGGATTTTGG
Hai7124	1695	GTAGAGGTGTTTATGGGTTGGGCGGCTCGGCCCGGCCCAATAGCCCGCCGAAAATATGGAAGGATTTTGG
Jizha45	1691	GTAGAGGTGTTTATGGGTTGGGCGGCTCGGCCCGGCCCAATAGCCCGCCGAAAATATGGAAGGATTTTGG
Xinhai18	1691	GTAGAGGTGTTTATGGGTTGGGCGGCTCGGCCCGGCCCAATAGCCCGCCGAAAATATGGAAGGATTTTGG
11452GZ	1697	GTAGAGGTGTTTATGGGTTGGGCGGCTCGGCCCGGCCCAACAGCCCGCCCGAAATATGGAAGGG-TTTGG
TM-1(BGI)	1697	GTAGAGGTGTTTATGGGTTGGGCGGCTCGGCCCGGCCCAACAGCCCGCCCGAAATATGGAAGGG-TTTGG
Zhousuohongjijiaoye	1702	GTAGAGGTGTTTATGGGTTGGGCGGCTCGGCCCGGCCCAACAGCCCGCCCGAAATATGGAAGGG-TTTGG
TM-1(this study)	1697	GTAGAGGTGTTTATGGGTTGGGCGGCTCGGCCCGGCCCAACAGCCCGCCCGAAATATGGAAGGG-TTTGG
T586	1696	GTAGAGGTGTTTATGGGTTGGGCGGCTCGGCCCGGCCCAACAGCCCGCCCGAAATATGGAAGGG-TTTGG
SL1-7-1	1696	GTAGAGGTGTTTATGGGTTGGGCGGCTCGGCCCGGCCCAACAGCCCGCCCGAAATATGGAAGGG-TTTGG
SA27	1698	GTAGAGGTGTTTATGGGTTGGGCGGCTCGGCCCGGCCCAACAGCCCGCCCGAAATATGGAAGGG-TTTGG
081925 fl	1696	GTAGAGGTGTTTATGGGTTGGGCGGCTCGGCCCGGCCCAACAGCCCGCCCGAAATATGGAAGGG-TTTGG
Xu142 fl	1696	GTAGAGGTGTTTATGGGTTGGGCGGCTCGGCCCGGCCCAACAGCCCGCCCGAAATATGGAAGGG-TTTGG
TM-1(NAU)	1696	GTAGAGGTGTTTATGGGTTGGGCGGCTCGGCCCGGCCCAACAGCCCGCCCGAAATATGGAAGGG-TTTGG
Gorai.008G179600.1	1659	--
Shixiaya1(Ga12G1199.1 CRI)	1705	--

n2	1761	ATAAAAATATAGGCCCGAAATATGGGCTCGGGCACCACTTTTTTTGCCCGAGCCCGGCCCGGCCCGATAT
3-79(this study)	1761	ATAAAAATATAGGCCCGAAATATGGGCTCGGGCACCACTTTTTTTGCCCGAGCCCGGCCCGGCCCGATAT
MD17	1761	ATAAAAATATAGGCCCGAAATATGGGCTCGGGCACCACTTTTTTTGCCCGAGCCCGGCCCGGCCCGATAT
Hai7124	1765	ATAAAAATATAGGCCCGAAATATGGGCTCGGGCACCACTTTTTTTGCCCGAGCCCGGCCCGGCCCGATAT
Jizha45	1761	ATAAAAATATAGGCCCGAAATATGGGCTCGGGCACCACTTTTTTTGCCCGAGCCCGGCCCGGCCCGATAT
Xinhai18	1761	ATAAAAATATAGGCCCGAAATATGGGCTCGGGCACCACTTTTTTTGCCCGAGCCCGGCCCGGCCCGATAT
11452GZ	1766	ATAAAAATATAGGCCCGAAATATGGGCTCGGGCACCACTTTTTTTGCCTGAGCCCGGCCCGGCCCGATAT
TM-1(BGI)	1766	ATAAAAATATAGGCCCGAAATATGGGCTCGGGCACCACTTTTTTTGCCTGAGCCCGGCCCGGCCCGATAT
Zhousuohongjijiaoye	1771	ATAAAAATATAGGCCCGAAATATGGGCTCGGGCACCACTTTTTTTGCCTGAGCCCGGCCCGGCCCGATAT
TM-1(this study)	1766	ATAAAAATATAGGCCCGAAATATGGGCTCGGGCACCACTTTTTTTGCCTGAGCCCGGCCCGGCCCGATAT
T586	1765	ATAAAAATATAGGCCCGAAATATGGGCTCGGGCACCACTTTTTTTGCCTGAGCCCGGCCCGGCCCGATAT
SL1-7-1	1765	ATAAAAATATAGGCCCGAAATATGGGCTCGGGCACCACTTTTTTTGCCTGAGCCCGGCCCGGCCCGATAT
SA27	1767	ATAAAAATATAGGCCCGAAATATGGGCTCGGGCACCACTTTTTTTGCCTGAGCCCGGCCCGGCCCGATAT
081925 fl	1765	ATAAAAATATAGGCCCGAAATATGGGCTCGGGCACCACTTTTTTTGCCTGAGCCCGGCCCGGCCCGATAT
Xu142 fl	1765	ATAAAAATATAGGCCCGAAATATGGGCTCGGGCACCACTTTTTTTGCCTGAGCCCGGCCCGGCCCGATAT
TM-1(NAU)	1765	ATAAAAATATAGGCCCGAAATATGGGCTCGGGCACCACTTTTTTTGCCTGAGCCCGGCCCGGCCCGATAT
Gorai.008G179600.1	1659	--
Shixiaya1(Ga12G1199.1 CRI)	1705	--

**n2**	1831	AATAAATATATTTATTTTTTAAATTTTTTTTAATTTTAAAATATTTTTAAAATACTTTTTTATTATTTTT
**3-79(this study)**	1831	AATAAATATATTTATTTTTTAAATTTTTTTTAATTTTAAAATATTTTTAAAATACTTTTTTATTATTTTT
**MD17**	1831	AATAAATATATTTATTTTTTAAATTTTTTTTAATTTTAAAATATTTTTAAAATACTTTTTTATTATTTTT
**Hai7124**	1835	AATAAATATATTTATTTTTTAAATTTTTTTTAATTTTAAAATATTTTTAAAATACTTTTTTATTATTTTT
**Jizha45**	1831	AATAAATATATTTATTTTTTAAATTTTTTTTAATTTTAAAATATTTTTAAAATACTTTTTTATTATTTTT
**Xinhai18**	1831	AATAAATATATTTATTTTTTAAATTTTTTTTAATTTTAAAATATTTTTAAAATACTTTTTTATTATTTTT
**11452GZ**	1836	AATACATATATTTATTTTTTAAAATTTTTTTAATTTTAAAAT-TTTTTAAAATACTTTTTTATTATTTTT
**TM-1(BGI)**	1836	AATACATATATTTATTTTTTAAAATTTTTTTAATTTTAAAAT-TTTTTAAAATACTTTTTTATTATTTTT
**Zhousuohongjijiaoye**	1841	AATACATATATTTATTTTTTAAAATTTTTTTAATTTTAAAAT-TTTTTAAAATACTTTTTTATTATTTTT
**TM-1(this study)**	1836	AATACATATATTTATTTTTTAAAATTTTTTTAATTTTAAAAT-TTTTTAAAATACTTTTTTATTATTTTT
**T586**	1835	AATAAATATATTTATTTTTTAAAATTTTTTTAATTTTAAAATATTTTTAAAATACTTTTTTATTATTTTT
**SL1-7-1**	1835	AATAAATATATTTATTTTTTAAAATTTTTTTAATTTTAAAATATTTTTAAAATACTTTTTTATTATTTTT
**SA27**	1837	AATAAATATATTTATTTTTTAAAATTTTTTTAATTTTAAAATATTTTTAAAATACTTTTTTATTATTTTT
**081925 fl**	1835	AATAAATATATTTATTTTTTAAAATTTTTTTAATTTTAAAATATTTTTAAAATACTTTTTTATTATTTTT
**Xu142 fl**	1835	AATAAATATATTTATTTTTTAAAATTTTTTTAATTTTAAAATATTTTTAAAATACTTTTTTATTATTTTT
**TM-1(NAU)**	1835	AATAAATATATTTATTTTTTAAAATTTTTTTAATTTTAAAATATTTTTAAAATACTTTTTTATTATTTTT
**Gorai.008G179600.1**	1659	--
**Shixiaya1(Ga12G1199.1 CRI)**	1705	--

**n2**	1901	TAATTTTAAAATTTTTTTAAAATACTTTTTTAAATTTTTAAAATAAATTTTTGGTATTTATTAAAAAATG
**3-79(this study)**	1901	TAATTTTAAAATTTTTTTAAAATACTTTTTTAAATTTTTAAAATAAATTTTTGGTATTTATTAAAAAATG
**MD17**	1901	TAATTTTAAAATTTTTTTAAAATACTTTTTTAAATTTTTAAAATAAATTTTTGGTATTTATTAAAAAATG
**Hai7124**	1905	TAATTTTAAAATTTTTTTAAAATACTTTTTTAAATTTTTAAAATAAATTTTTGGTATTTATTAAAAAATG
**Jizha45**	1901	TAATTTTAAAATTTTTTTAAAATACTTTTTTAAATTTTTAAAATAAATTTTTGGTATTTATTAAAAAATG
**Xinhai18**	1901	TAATTTTAAAATTTTTTTAAAATACTTTTTTAAATTTTTAAAATAAATTTTTGGTATTTATTAAAAAATG
**11452GZ**	1905	TAATTTTAAAATTTTTTTAAAATACTTTTTTTAATTTTTAAAATAAATTTTTGGTATTTATTAAAAAATG
**TM-1(BGI)**	1905	TAATTTTAAAATTTTTTTAAAATACTTTTTTTAATTTTTAAAATAAATTTTTGGTATTTATTAAAAAATG
**Zhousuohongjijiaoye**	1910	TAATTTTAAAATTTTTTTAAAATACTTTTTTTAATTTTTAAAATAAATTTTTGGTATTTATTAAAAAATG
**TM-1(this study)**	1905	TAATTTTAAAATTTTTTTAAAATACTTTTTTTAATTTTTAAAATAAATTTTTGGTATTTATTAAAAAATG
**T586**	1905	TAATTTTAAATTTTTTTTAAAATACTTTTTTTAATTTTTAAAATAAATTTTTGGTATTTATTAAAAAATG
**SL1-7-1**	1905	TAATTTTAAATTTTTTTTAAAATACTTTTTTTAATTTTTAAAATAAATTTTTGGTATTTATTAAAAAATG
**SA27**	1907	TAATTTTAAATTTTTTTTAAAATACTTTTTTTAATTTTTAAAATAAATTTTTGGTATTTATTAAAAAATG
**081925 fl**	1905	TAATTTTAAATTTTTTTTAAAATACTTTTTTTAATTTTTAAAATAAATTTTTGGTATTTATTAAAAAATG
**Xu142 fl**	1905	TAATTTTAAATTTTTTTTAAAATACTTTTTTTAATTTTTAAAATAAATTTTTGGTATTTATTAAAAAATG
**TM-1(NAU)**	1905	TAATTTTAAATTTTTTTTAAAATACTTTTTTTAATTTTTAAAATAAATTTTTGGTATTTATTAAAAAATG
**Gorai.008G179600.1**	1659	--
**Shixiaya1(Ga12G1199.1 CRI)**	1705	--

**n2**	1971	GGCCGAGTCGAGCCAGGCCTGGGCTTATGCTTTTTTCCCGGGCCGGGCCTGGGCAAAATTTTAGGCCCAT
**3-79(this study)**	1971	GGCCGAGTCGAGCCAGGCCTGGGCTTATGCTTTTTTCCCGGGCCGGGCCTGGGCAAAATTTTAGGCCCAT
**MD17**	1971	GGCCGAGTCGAGCCAGGCCTGGGCTTATGCTTTTTTCCCGGGCCGGGCCTGGGCAAAATTTTAGGCCCAT
**Hai7124**	1975	GGCCGAGTCGAGCCAGGCCTGGGCTTATGCTTTTTTCCCGGGCCGGGCCTGGGCAAAATTTTAGGCCCAT
**Jizha45**	1971	GGCCGAGTCGAGCCAGGCCTGGGCTTATGCTTTTTTCCCGGGCCGGGCCTGGGCAAAATTTTAGGCCCAT
**Xinhai18**	1971	GGCCGAGTCGAGCCAGGCCTGGGCTTATGCTTTTTTCCCGGGCCGGGCCTGGGCAAAATTTTAGGCCCAT
**11452GZ**	1975	GGCCGAGTCGGGCCAGGCCTGGGCTTATGCTTTTTTCCCGGGCCGGGCCTGGGCAAAATTTTAGGCCCAT
**TM-1(BGI)**	1975	GGCCGAGTCGGGCCAGGCCTGGGCTTATGCTTTTTTCCCGGGCCGGGCCTGGGCAAAATTTTAGGCCCAT
**Zhousuohongjijiaoye**	1980	GGCCGAGTCGGGCCAGGCCTGGGCTTATGCTTTTTTCCCGGGCCGGGCCTGGGCAAAATTTTAGGCCCAT
**TM-1(this study)**	1975	GGCCGAGTCGGGCCAGGCCTGGGCTTATGCTTTTTTCCCGGGCCGGGCCTGGGCAAAATTTTAGGCCCAT
**T586**	1975	GGCCGAGTCGGGCCAGGCCTGGGCTTATGCTTTTTTCCCGGGCCGGGCCTGGGCAAAATTTTAGGCCCAT
**SL1-7-1**	1975	GGCCGAGTCGGGCCAGGCCTGGGCTTATGCTTTTTTCCCGGGCCGGGCCTGGGCAAAATTTTAGGCCCAT
**SA27**	1977	GGCCGAGTCGGGCCAGGCCTGGGCTTATGCTTTTTTCCCGGGCCGGGCCTGGGCAAAATTTTAGGCCCAT
**081925 fl**	1975	GGCCGAGTCGGGCCAGGCCTGGGCTTATGCTTTTTTCCCGGGCCGGGCCTGGGCAAAATTTTAGGCCCAT
**Xu142 fl**	1975	GGCCGAGTCGGGCCAGGCCTGGGCTTATGCTTTTTTCCCGGGCCGGGCCTGGGCAAAATTTTAGGCCCAT
**TM-1(NAU)**	1975	GGCCGAGTCGGGCCAGGCCTGGGCTTATGCTTTTTTCCCGGGCCGGGCCTGGGCAAAATTTTAGGCCCAT
**Gorai.008G179600.1**	1659	--
**Shixiaya1(Ga12G1199.1 CRI)**	1705	--

```
n2                                2041 ATTTTGGGCCGGGCCGGGGCCGGGCCTAGGACCCGGGCCAAATTTTT----------------------A
3-79(this study)                  2041 ATTTTGGGCCGGGCCGGGGCCGGGCCTAGGACCCGGGCCAAATTTTT----------------------A
MD17                              2041 ATTTTGGGCCGGGCCGGGGCCGGGCCTAGGACCCGGGCCAAATTTTT----------------------A
Hai7124                           2045 ATTTTGGGCCGGGCCGGGGCCGGGCCTAGGACCCGGGCCAAATTTTT----------------------A
Jizha45                           2041 ATTTTGGGCCGGGCCGGGGCCGGGCCTAGGACCCGGGCCAAATTTTT----------------------A
Xinhai18                          2041 ATTTTGGGCCGGGCCGGGGCCGGGCCTAGGACCCGGGCCAAATTTTT----------------------A
11452GZ                           2045 ATTTTGGGCCGGGCCGGGGCCGGGCCTAGGACT-------------------------------------
TM-1(BGI)                         2045 ATTTTGGGCCGGGCCGGGGCCGGGCCTAGGACT-------------------------------------
Zhousuohongjijiaoye               2050 ATTTTGGGCCGGGCCGGGGCCGGGCCTAGGACT-------------------------------------
TM-1(this study)                  2045 ATTTTGGGCCGGGCCGGGGCCGGGCCTAGGACT-------------------------------------
T586                              2045 ATTTTGGGCCGGGCCGGGGCCGGGCCTAGGACTCGGGCCAAATTTTTTATGGGCCCGGCCCAAACCCAGC
SL1-7-1                           2045 ATTTTGGGCCGGGCCGGGGCCGGGCCTAGGACTCGGGCCAAATTTTTTATGGGCCCGGCCCAAACCCAGC
SA27                              2047 ATTTTGGGCCGGGCCGGGGCCGGGCCTAGGACTCGGGCCAAATTTTTTATGGGCCCGGCCCAAACCCAGC
081925 fl                         2045 ATTTTGGGCCGGGCCGGGGCCGGGCCTAGGACTCGGGCCAAATTTTTTATGGGCCCGGCCCAAACCCAGC
Xu142 fl                          2045 ATTTTGGGCCGGGCCGGGGCCGGGCCTAGGACTCGGGCCAAATTTTTTATGGGCCCGGCCCAAACCCAGC
TM-1(NAU)                         2045 ATTTTGGGCCGGGCCGGGGCCGGGCCTAGGACTCGGGCCAAATTTTTTATGGGCCCGGCCCAAACCCAGC
Gorai.008G179600.1                1659 ----------------------------------------------------------------------
Shixiaya1(Ga12G1199.1 CRI)        1705 ----------------------------------------------------------------------

n2                                2089 TGGGCCCGGCCCAAACCCAGCCCGGCCTGGCCCGAACCCGGCCCGGCCCATGAGCACCTCTAAAACATAG
3-79(this study)                  2089 TGGGCCCGGCCCAAACCCAGCCCGGCCTGGCCCGAACCCGGCCCGGCCCATGAGCACCTCTAAAACATAG
MD17                              2089 TGGGCCCGGCCCAAACCCAGCCCGGCCTGGCCCGAACCCGGCCCGGCCCATGAGCACCTCTAAAACATAG
Hai7124                           2093 TGGGCCCGGCCCAAACCCAGCCCGGCCTGGCCCGAACCCGGCCCGGCCCATGAGCACCTCTAAAACATAG
Jizha45                           2089 TGGGCCCGGCCCAAACCCAGCCCGGCCTGGCCCGAACCCGGCCCGGCCCATGAGCACCTCTAAAACATAG
Xinhai18                          2089 TGGGCCCGGCCCAAACCCAGCCCGGCCTGGCCCGAACCCGGCCCGGCCCATGAGCACCTCTAAAACATAG
11452GZ                           2077 -------------------------------------------CGGCCCATGAGCACCTCTAAAACATAG
TM-1(BGI)                         2077 -------------------------------------------CGGCCCATGAGCACCTCTAAAACATAG
Zhousuohongjijiaoye               2082 -------------------------------------------CGGCCCATGAGCACCTCTAAAACATAG
TM-1(this study)                  2077 -------------------------------------------CGGCCCATGAGCACCTCTAAAACATAG
T586                              2115 CCGGCCTGGCCCAAACCCGGCCCGGCCTGGCCCGAACCCGGCCCGGCCCATGAGCACCTCTAAAACATAG
SL1-7-1                           2115 CCGGCCTGGCCCAAACCCGGCCCGGCCTGGCCCGAACCCGGCCCGGCCCATGAGCACCTCTAAAACATAG
SA27                              2117 CCGGCCTGGCCCAAACCCGGCCCGGCCTGGCCCGAACCCGGCCCGGCCCATGAGCACCTCTAAAACATAG
081925 fl                         2115 CCGGCCTGGCCCAAACCCGGCCCGGCCTGGCCCGAACCCGGCCCGGCCCATGAGCACCTCTAAAACATAG
Xu142 fl                          2115 CCGGCCTGGCCCAAACCCGGCCCGGCCTGGCCCGAACCCGGCCCGGCCCATGAGCACCTCTAAAACATAG
TM-1(NAU)                         2115 CCGGCCTGGCCCAAACCCGGCCCGGCCTGGCCCGAACCCGGCCCGGCCCATGAGCACCTCTAAAACATAG
Gorai.008G179600.1                1659 ----------------------------------------------------------------ACATAG
Shixiaya1(Ga12G1199.1 CRI)        1705 ----------------------------------------------------------------ACATAG

n2                                2159 TGTTAGAAAAGAAAATTAAATTCTTAAAGAAGGGTATAATGGTCATATAGGCTTAAGTAATATTTATTAG
3-79(this study)                  2159 TGTTAGAAAAGAAAATTAAATTCTTAAAGAAGGGTATAATGGTCATATAGGCTTAAGTAATATTTATTAG
MD17                              2159 TGTTAGAAAAGAAAATTAAATTCTTAAAGAAGGGTATAATGGTCATATAGGCTTAAGTAATATTTATTAG
Hai7124                           2163 TGTTAGAAAAGAAAATTAAATTCTTAAAGAAGGGTATAATGGTCATATAGGCTTAAGTAATATTTATTAG
Jizha45                           2159 TGTTAGAAAAGAAAATTAAATTCTTAAAGAAGGGTATAATGGTCATATAGGCTTAAGTAATATTTATTAG
Xinhai18                          2159 TGTTAGAAAAGAAAATTAAATTCTTAAAGAAGGGTATAATGGTCATATAGGCTTAAGTAATATTTATTAG
11452GZ                           2105 TGTTAGAAAAGAAAATTAAATTCTTAAAGAAGGGTATAATGGTCATATAGGCTTAAGTAATATTTATTAG
TM-1(BGI)                         2105 TGTTAGAAAAGAAAATTAAATTCTTAAAGAAGGGTATAATGGTCATATAGGCTTAAGTAATATTTATTAG
Zhousuohongjijiaoye               2110 TGTTAGAAAAGAAAATTAAATTCTTAAAGAAGGGTATAATGGTCATATAGGCTTAAGTAATATTTATTAG
TM-1(this study)                  2105 TGTTAGAAAAGAAAATTAAATTCTTAAAGAAGGGTATAATGGTCATATAGGCTTAAGTAATATTTATTAG
T586                              2185 TGTTAGAAAAGAAAATTAAATTCTTAAAGAAGGGTATAATGGTCATATAGGCTTAAGTAATATTTATTAG
SL1-7-1                           2185 TGTTAGAAAAGAAAATTAAATTCTTAAAGAAGGGTATAATGGTCATATAGGCTTAAGTAATATTTATTAG
SA27                              2187 TGTTAGAAAAGAAAATTAAATTCTTAAAGAAGGGTATAATGGTCATATAGGCTTAAGTAATATTTATTAG
081925 fl                         2185 TGTTAGAAAAGAAAATTAAATTCTTAAAGAAGGGTATAATGGTCATATAGGCTTAAGTAATATTTATTAG
Xu142 fl                          2185 TGTTAGAAAAGAAAATTAAATTCTTAAAGAAGGGTATAATGGTCATATAGGCTTAAGTAATATTTATTAG
TM-1(NAU)                         2185 TGTTAGAAAAGAAAATTAAATTCTTAAAGAAGGGTATAATGGTCATATAGGCTTAAGTAATATTTATTAG
Gorai.008G179600.1                1668 TGTTAGAAAAGAAAATTATATTCTTAAACAAGGGTATAATGGTCATATAGGCTTAAGTAATATTTATTAC
Shixiaya1(Ga12G1199.1 CRI)        1714 TGTTAGAAAAGAAAATTAAATTCTTAAAGAAGGGTATAATGGTCATATAGGCTTATGCAATATTTATTAA

n2                                2228 -ACTGTAAGCCTATAAAACGCACTTATATATCTACTTTAAATTAACCAATTCTACCCACATTTTCGTTGC
```

```
3-79(this study)               2228 -ACTGTAAGCCTATAAAACGCACTTATATATCTACTTTAAATTAACCAATTCTACCCACATTTTCGTTGC
MD17                           2228 -ACTGTAAGCCTATAAAACGCACTTATATATCTACTTTAAATTAACCAATTCTACCCACATTTTCGTTGC
Hai7124                        2232 -ACTGTAAGCCTATAAAACGCACTTATATATCTACTTTAAATTAACCAATTCTACCCACATTTTCGTTGC
Jizha45                        2228 -ACTGTAAGCCTATAAAACGCACTTATATATCTACTTTAAATTAACCAATTCTACCCACATTTTCGTTGC
Xinhai18                       2228 -ACTGTAAGCCTATAAAACGCACTTATATATCTACTTTAAATTAACCAATTCTACCCACATTTTCGTTGC
11452GZ                        2174 -ACTGTAAGCCTATAAAACGCACTTATATATCTACTTTAAATTAACCAATTCTACCCACATTTTCGTTGC
TM-1(BGI)                      2174 -ACTGTAAGCCTATAAAACGCACTTATATATCTACTTTAAATTAACCAATTCTACCCACATTTTCGTTGC
Zhousuohongjijiaoye            2179 -ACTGTAAGCCTATAAAACGCACTTATATATCTACTTTAAATTAACCAATTCTACCCACATTTTCGTTGC
TM-1(this study)               2174 -ACTGTAAGCCTATAAAACGCACTTATATATCTACTTTAAATTAACCAATTCTACCCACATTTTCGTTGC
T586                           2254 -ACTGTAAGCCTATAAAACGCACTTATATATCTACTTTAAATTAACCAATTCTACCCACATTTTCGTTGC
SL1-7-1                        2254 -ACTGTAAGCCTATAAAACGCACTTATATATCTACTTTAAATTAACCAATTCTACCCACATTTTCGTTGC
SA27                           2256 -ACTGTAAGCCTATAAAACGCACTTATATATCTACTTTAAATTAACCAATTCTACCCACATTTTCGTTGC
081925 fl                      2254 -ACTGTAAGCCTATAAAACGCACTTATATATCTACTTTAAATTAACCAATTCTACCCACATTTTCGTTGC
Xu142 fl                       2254 -ACTGTAAGCCTATAAAACGCACTTATATATCTACTTTAAATTAACCAATTCTACCCACATTTTCGTTGC
TM-1(NAU)                      2254 -ACTGTAAGCCTATAAAACGCACTTATATATCTACTTTAAATTAACCAATTCTACCCACATTTTCGTTGC
Gorai.008G179600.1             1737 -ACTGTAAGCCTATAAAACGCACTTATATATCTACTTTAAATTAACCAACTCTACCCACATTTTCGTTGC
Shixiaya1(Ga12G1199.1 CRI)     1784 CACTGTAAGCCTATAAAACGCAGTTATATATCTACTTTAAATTAACCAACTCTACCCACATTTTCGTTGC

n2                             2298 TTTCAACTGCGTATAATATCTC--TCTCTATCATCTCTTTATCATTGTCTGTTTTCAAATTAAGGAACAA
3-79(this study)               2298 TTTCAACTGCGTATAATATCTC--TCTCTATCATCTCTTTATCATTGTCTGTTTTCAAATTAAGGAACAA
MD17                           2298 TTTCAACTGCGTATAATATCTC--TCTCTATCATCTCTTTATCATTGTCTGTTTTCAAATTAAGGAACAA
Hai7124                        2302 TTTCAACTGCGTATAATATCTC--TCTCTATCATCTCTTTATCATTGTCTGTTTTCAAATTAAGGAACAA
Jizha45                        2298 TTTCAACTGCGTATAATATCTC--TCTCTATCATCTCTTTATCATTGTCTGTTTTCAAATTAAGGAACAA
Xinhai18                       2298 TTTCAACTGCGTATAATATCTC--TCTCTATCATCTCTTTATCATTGTCTGTTTTCAAATTAAGGAACAA
11452GZ                        2244 TTTCAACTGCGTATAATATCTC--TCTCTATCATCTATTTATCATTGTCTGTTTTCAAATTAAGGAACAA
TM-1(BGI)                      2244 TTTCAACTGCGTATAATATCTC--TCTCTATCATCTATTTATCATTGTCTGTTTTCAAATTAAGGAACAA
Zhousuohongjijiaoye            2249 TTTCAACTGCGTATAATATCTC--TCTCTATCATCTATTTATCATTGTCTGTTTTCAAATTAAGGAACAA
TM-1(this study)               2244 TTTCAACTGCGTATAATATCTC--TCTCTATCATCTATTTATCATTGTCTGTTTTCAAATTAAGGAACAA
T586                           2324 TTTCAACTGCGTATAATATCTC--TCTCTATCATCTATTTATCATTGTCTGTTTTCAAATTAAGGAACAA
SL1-7-1                        2324 TTTCAACTGCGTATAATATCTC--TCTCTATCATCTATTTATCATTGTCTGTTTTCAAATTAAGGAACAA
SA27                           2326 TTTCAACTGCGTATAATATCTC--TCTCTATCATCTATTTATCATTGTCTGTTTTCAAATTAAGGAACAA
081925 fl                      2324 TTTCAACTGCGTATAATATCTC--TCTCTATCATCTATTTATCATTGTCTGTTTTCAAATTAAGGAACAA
Xu142 fl                       2324 TTTCAACTGCGTATAATATCTC--TCTCTATCATCTATTTATCATTGTCTGTTTTCAAATTAAGGAACAA
TM-1(NAU)                      2324 TTTCAACTGCGTATAATATCTC--TCTCTATCATCTATTTATCATTGTCTGTTTTCAAATTAAGGAACAA
Gorai.008G179600.1             1807 TTTCAACTGCGTATAATATC----TCTCTATCATCTCTTTATCATTGTCTGTTTTCAAATTAAGGAACAA
Shixiaya1(Ga12G1199.1 CRI)     1854 TTTCAACTGCGTATAATCTCTCTTTCTCTATCATCTCTTTATCATTGTCTGTTTTCAAATTAAGGAACCA

                                                                          Stat codon of of MML3_Dt
n2                             2366 AATTCATTGAGGATTCGGCCTGGCTTGACTTCAAAGAACATGCAGCAGTCTCCA
3-79(this study)               2366 AATTCATTGAGGATTCGGCCTGGCTTGACTTCAAAGAACATGCAGCAGTCTCCA
MD17                           2366 AATTCATTGAGGATTCGGCCTGGCTTGACTTCAAAGAACATGCAGCAGTCTCCA
Hai7124                        2370 AATTCATTGAGGATTCGGCCTGGCTTGACTTCAAAGAACATGCAGCAGTCTCCA
Jizha45                        2366 AATTCATTGAGGATTCGGCCTGGCTTGACTTCAAAGAACATGCAGCAGTCTCCA
Xinhai18                       2366 AATTCATTGAGGATTCGGCCTGGCTTGACTTCAAAGAACATGCAGCAGTCTCCA
11452GZ                        2312 AATTCATTGAGGATTCGGCCTGGCTTGACTTCAAAGAACATGCAGCAGTCTCCA
TM-1(BGI)                      2312 AATTCATTGAGGATTCGGCCTGGCTTGACTTCAAAGAACATGCAGCAGTCTCCA
Zhousuohongjijiaoye            2317 AATTCATTGAGGATTCGGCCTGGCTTGACTTCAAAGAACATGCAGCAGTCTCCA
TM-1(this study)               2312 AATTCATTGAGGATTCGGCCTGGCTTGACTTCAAAGAACATGCAGCAGTCTCCA
T586                           2392 AATTCATTGAGGATTCGGCCTGGCTTGACTTCAAAGAACATGCAGCAGTCTCCA
SL1-7-1                        2392 AATTCATTGAGGATTCGGCCTGGCTTGACTTCAAAGAACATGCAGCAGTCTCCA
SA27                           2394 AATTCATTGAGGATTCGGCCTGGCTTGACTTCAAAGAACATGCAGCAGTCTCCA
081925 fl                      2392 AATTCATTGAGGATTCGGCCTGGCTTGACTTCAAAGAACATGCAGCAGTCTCCA
Xu142 fl                       2392 AATTCATTGAGGATTCGGCCTGGCTTGACTTCAAAGAACATGCAGCAGTCTCCA
TM-1(NAU)                      2392 AATTCATTGAGGATTCGGCCTGGCTTGACTTCAAAGAACATGCAGCAGTCTCCA
Gorai.008G179600.1             1873 AATTCATTGAGGATTCGGCCTGGCTTGACTTCAAAGAACATGCAGCAGTCTCCA
Shixiaya1(Ga12G1199.1 CRI)     1924 AATTCATTGAGGATTCGGCCTGGCTTGA-TTCAAAGAACATGCAGCAGTCTCCA
```
