# Supplementary material for: A Retrotransposon Insertion in GhMML3_D12 Is Likely Responsible for the Lintless Locus li3 of Tetraploid Cotton
Source: Front Plant Sci. 2020 Nov 26;11:593679. doi: 10.3389/fpls.2020.593679 (PMC7725795; doi:10.3389/fpls.2020.593679)
Supplement: Supplementary file 1 [file Data_Sheet_1.zip › Fig S1-Fig S8 and Table S1-S11/Fig S8.pdf]

Fig. S8. Alignment of the coding sequences of *MML4_D12* from normal lines and mutants. Normal lines: TM-1, Ma85, JZ-Wild; fuzzless mutants: gznn1-1, n2, TaoGZ, T586, GZNn2-1, 11452GZ; Gb lines: 3-79, Xinhai21 ; fiberless mutants: 081925 fl, MD17, Xu142 fl, SL1-7-1, JZ-fl.

```
n2                               1   ATGCTGAGCAAAATGCGGCCACCGTCTCCGAACAGAAAGAAAGAGGTGAGATTGAAGAGAGGGCCATGGA
gznn1_1                          1   ATGCTGAGCAAAATGCGGCCACCGTCTCCGAACAGAAAGAAAGAGGTGAGATTGAAGAGAGGGCCATGGA
Xinhai21(GOBAR_DD14076.1 NAU)    1   ATGCTGAGCAAAATGCGGCCACCGTCTCCGAACAGAAAGAAAGAGGTGAGATTGAAGAGAGGGCCATGGA
3-79(Gbscaffold4699.2.0 HZAU)    1   ATGCTGAGCAAAATGCGGCCACCGTCTCCGAACAGAAAGAAAGAGGTGAGATTGAAGAGAGGGCCATGGA
MD17                             1   ATGCTGAGCAAAATGCGGCCACCGTCTCCGAACAGAAAGAAAGAGGTGAGATTGAAGAGAGGGCCATGGA
Ma85                             1   ATGCTGAGCAAAATGCGGCCACCGTCTCCGAACAGAAAGAAAGAGGTGAGATTGAAGAGAGGGCCATGGA
JZ-Wild                          1   ATGCTGAGCAAAATGCGGCCACCGTCTCCGAACAGAAAGAAAGAGGTGAGATTGAAGAGAGGGCCATGGA
TaoGZ                            1   ATGCTGAGCAAAATGCGGCCACCGTCTCCGAACAGAAAGAAAGAGGTGAGATTGAAGAGAGGGCCATGGA
GZNn2_1                          1   ATGCTGAGCAAAATGCGGCCACCGTCTCCGAACAGAAAGAAAGAGGTGAGATTGAAGAGAGGGCCATGGA
TM-1(this study)                 1   ATGCTGAGCAAAATGCGGCCACCGTCTCCGAACAGAAAGAAAGAGGTGAGATTGAAGAGAGGGCCATGGA
11452GZ                          1   ATGCTGAGCAAAATGCGGCCACCGTCTCCGAACAGAAAGAAAGAGGTGAGATTGAAGAGAGGGCCATGGA
SL1-7-1                          1   ATGCTGAGCAAAATGCGGCCACCGTCTCCGAACAGAAAGAAAGAGGTGAGATTGAAGAGAGGGCCATGGA
TM-1(Gh_D12G1629 NAU)            1   ATGCTGAGCAAAATGCGGCCACCGTCTCCGAACAGAAAGAAAGAGGTGAGATTGAAGAGAGGGCCATGGA
JZ-fl                            1   ATGCTGAGCAAAATGCGGCCACCGTCTCCGAACAGAAAGAAAGAGGTGAGATTGAAGAGAGGGCCATGGA
Xu142_fl                         1   ATGCTGAGCAAAATGCGGCCACCGTCTCCGAACAGAAAGAAAGAGGTGAGATTGAAGAGAGGGCCATGGA
081925_fl                        1   ATGCTGAGCAAAATGCGGCCACCGTCTCCGAACAGAAAGAAAGAGGTGAGATTGAAGAGAGGGCCATGGA
TM-1(CotAD_16205.1 BGI)          1   ATGCTGAGCAAAATGCGGCCACCGTCTCCGAACAGAAAGAAAGAGGTGAGATTGAAGAGAGGGCCATGGA
Gorai.008G179800.1               1   ATGCTAAGTAAAATGCGGCCACCGTCTCCGAACAGAAAGAAAGAGGTGAGATTGAAGAGAGGGCCATGGA
```

```
n2                               71  CAGCTGAAGAAGACAAATTACTGACGGCTTACATTCAAAAACATGGCTATGGTAGCTGGGGTTCCTTGCC
gznn1_1                          71  CAGCTGAAGAAGACAAATTACTGACGGCTTACATTCAAAAACATGGCTATGGTAGCTGGGGTTCCTTGCC
Xinhai21(GOBAR_DD14076.1 NAU)    71  CAGCTGAAGAAGACAAATTACTGACGGCTTACATTCAAAAACATGGCTATGGTAGCTGGGGTTCCTTGCC
3-79(Gbscaffold4699.2.0 HZAU)    71  CAGCTGAAGAAGACAAATTACTGACGGCTTACATTCAAAAACATGGCTATGGTAGCTGGGGTTCCTTGCC
MD17                             71  CAGCTGAAGAAGACAAATTACTGACGGCTTACATTCAAAAACATGGCTATGGTAGCTGGGGTTCCTTGCC
Ma85                             71  CAGCTGAAGAAGACAAATTACTGACGGCTTACATTCAAAAACATGGCTATGGTAGCTGGGGTTCCTTGCC
JZ-Wild                          71  CAGCTGAAGAAGACAAATTACTGACGGCTTACATTCAAAAACATGGCTATGGTAGCTGGGGTTCCTTGCC
TaoGZ                            71  CAGCTGAAGAAGACAAATTACTGACGGCTTACATTCAAAAACATGGCTATGGTAGCTGGGGTTCCTTGCC
GZNn2_1                          71  CAGCTGAAGAAGACAAATTACTGACGGCTTACATTCAAAAACATGGCTATGGTAGCTGGGGTTCCTTGCC
TM-1(this study)                 71  CAGCTGAAGAAGACAAATTACTGACGGCTTACATTCAAAAACATGGCTATGGTAGCTGGGGTTCCTTGCC
11452GZ                          71  CAGCTGAAGAAGACAAATTACTGACGGCTTACATTCAAAAACATGGCTATGGTAGCTGGGGTTCCTTGCC
SL1-7-1                          71  CAGCTGAAGAAGACAAATTACTGACGGCTTACATTCAAAAACATGGCTATGGTAGCTGGGGTTCCTTGCC
TM-1(Gh_D12G1629 NAU)            71  CAGCTGAAGAAGACAAATTACTGACGGCTTACATTCAAAAACATGGCTATGGTAGCTGGGGTTCCTTGCC
JZ-fl                            71  CAGCTGAAGAAGACAAATTACTGACGGCTTACATTCAAAAACATGGCTATGGTAGCTGGGGTTCCTTGCC
Xu142_fl                         71  CAGCTGAAGAAGACAAATTACTGACGGCTTACATTCAAAAACATGGCTATGGTAGCTGGGGTTCCTTGCC
081925_fl                        71  CAGCTGAAGAAGACAAATTACTGACGGCTTACATTCAAAAACATGGCTATGGTAGCTGGGGTTCCTTGCC
TM-1(CotAD_16205.1 BGI)          71  CAGCTGAAGAAGACAAATTACTGACGGCTTACATTCAAAAACATGGCTATGGCAGCAGGGGTTCCTTGCC
Gorai.008G179800.1               71  CAGCTGAAGAAGACAAATTACTGACGGCTTACATTCAAAAACATGGCTATGGTAGCTGGGGTTCCTTGCC
```

```
n2                                  141 TCACAAAGCTGGACTTGAACGATGTGGGAAGAGCTGCCGACTGAGATGGATTAACTACTTAAGACCTTAT
gznn1_1                             141 TCACAAAGCTGGACTTGAACGATGTGGGAAGAGCTGCCGACTGAGATGGATTAACTACTTAAGACCTTAT
Xinhai21(GOBAR_DD14076.1 NAU)       141 TCACAAAGCTGGACTTGAACGATGTGGGAAGAGCTGCCGACTGAGATGGATTAACTACTTAAGACCTTAT
3-79(Gbscaffold4699.2.0 HZAU)       141 TCACAAAGCTGGACTTGAACGATGTGGGAAGAGCTGCCGACTGAGATGGATTAACTACTTAAGACCTTAT
MD17                                141 TCACAAAGCTGGACTTGAACGATGTGGGAAGAGCTGCCGACTGAGATGGATTAACTACTTAAGACCTTAT
Ma85                                141 TCACAAAGCTGGACTTGAACGATGTGGGAAGAGCTGCCGACTGAGATGGATTAACTACTTAAGACCTTAT
JZ-Wild                             141 TCACAAAGCTGGACTTGAACGATGTGGGAAGAGCTGCCGACTGAGATGGATTAACTACTTAAGACCTTAT
TaoGZ                               141 TCACAAAGCTGGACTTGAACGATGTGGGAAGAGCTGCCGACTGAGATGGATTAACTACTTAAGACCTTAT
GZNn2_1                             141 TCACAAAGCTGGACTTGAACGATGTGGGAAGAGCTGCCGACTGAGATGGATTAACTACTTAAGACCTTAT
TM-1(this study)                    141 TCACAAAGCTGGACTTGAACGATGTGGGAAGAGCTGCCGACTGAGATGGATTAACTACTTAAGACCTTAT
11452GZ                             141 TCACAAAGCTGGACTTGAACGATGTGGGAAGAGCTGCCGACTGAGATGGATTAACTACTTAAGACCTTAT
SL1-7-1                             141 TCACAAAGCTGGACTTGAACGATGTGGGAAGAGCTGCCGACTGAGATGGATTAACTACTTAAGACCTTAT
TM-1(Gh_D12G1629 NAU)               141 TCACAAAGCTGGACTTGAACGATGTGGGAAGAGCTGCCGACTGAGATGGATTAACTACTTAAGACCTTAT
JZ-fl                               141 TCACAAAGCTGGACTTGAACGATGTGGGAAGAGCTGCCGACTGAGATGGATTAACTACTTAAGACCTTAT
Xu142_fl                            141 TCACAAAGCTGGACTTGAACGATGTGGGAAGAGCTGCCGACTGAGATGGATTAACTACTTAAGACCTTAT
081925_fl                           141 TCACAAAGCTGGACTTGAACGATGTGGGAAGAGCTGCCGACTGAGATGGATTAACTACTTAAGACCTTAT
TM-1(CotAD_16205.1 BGI)             141 TCACAAAGCTGGACTTGAACGATGTGGAAAGAGCTGCCGACTGAGATGGATTAACTACTTAAGACCTGAT
Gorai.008G179800.1                  141 TCACAAAGCTGGACTTGAACGATGTGGGAAGAGCTGCCGACTGAGATGGATTAACTACTTAAGACCTGAT

n2                                  211 ATCAAAAGAGGAAAGTTTAGTTTAGAGGAAGAACAGACCATCATTCAACTCCATGCCTTTCTTGGAAACA
gznn1_1                             211 ATCAAAAGAGGAAAGTTTAGTTTAGAGGAAGAACAGACCATCATTCAACTCCATGCCTTTCTTGGAAACA
Xinhai21(GOBAR_DD14076.1 NAU)       211 ATCAAAAGAGGAAAGTTTAGTTTAGAGGAAGAACAGACCATCATTCAACTCCATGCCTTTCTTGGAAACA
3-79(Gbscaffold4699.2.0 HZAU)       211 ATCAAAAGAGGAAAGTTTAGTTTAGAGGAAGAACAGACCATCATTCAACTCCATGCCTTTCTTGGAAACA
MD17                                211 ATCAAAAGAGGAAAGTTTAGTTTAGAGGAAGAACAGACCATCATTCAACTCCATGCCTTTCTTGGAAACA
Ma85                                211 ATCAAAAGAGGAAAGTTTAGTTTAGAGGAAGAACAGACCATCATTCAACTCCATGCCTTTCTTGGAAACA
JZ-Wild                             211 ATCAAAAGAGGAAAGTTTAGTTTAGAGGAAGAACAGACCATCATTCAACTCCATGCCTTTCTTGGAAACA
TaoGZ                               211 ATCAAAAGAGGAAAGTTTAGTTTAGAGGAAGAACAGACCATCATTCAACTCCATGCCTTTCTTGGAAACA
GZNn2_1                             211 ATCAAAAGAGGAAAGTTTAGTTTAGAGGAAGAACAGACCATCATTCAACTCCATGCCTTTCTTGGAAACA
TM-1(this study)                    211 ATCAAAAGAGGAAAGTTTAGTTTAGAGGAAGAACAGACCATCATTCAACTCCATGCCTTTCTTGGAAACA
11452GZ                             211 ATCAAAAGAGGAAAGTTTAGTTTAGAGGAAGAACAGACCATCATTCAACTCCATGCCTTTCTTGGAAACA
SL1-7-1                             211 ATCAAAAGAGGAAAGTTTAGTTTAGAGGAAGAACAGACCATCATTCAACTCCATGCCTTTCTTGGAAACA
TM-1(Gh_D12G1629 NAU)               211 ATCAAAAGAGGAAAGTTTAGTTTAGAGGAAGAACAGACCATCATTCAACTCCATGCCTTTCTTGGAAACA
JZ-fl                               211 ATCAAAAGAGGAAAGTTTAGTTTAGAGGAAGAACAGACCATCATTCAACTCCATGCCTTTCTTGGAAACA
Xu142_fl                            211 ATCAAAAGAGGAAAGTTTAGTTTAGAGGAAGAACAGACCATCATTCAACTCCATGCCTTTCTTGGAAACA
081925_fl                           211 ATCAAAAGAGGAAAGTTTAGTTTAGAGGAAGAACAGACCATCATTCAACTCCATGCCTTTCTTGGAAACA
TM-1(CotAD_16205.1 BGI)             211 ATCAAAAGAGGAAAGTTTAGTTTAGAGGAAGAACAGACCATCATTCAACTCCATGCCTTTCTTGGAAACA
Gorai.008G179800.1                  211 ATCAAAAGAGGAAAGTTTAGTTTAGAGGAAGAACAGACCATCATTCAACTCCATGCCTTTCTTGGAAACA

n2                                  281 GGTGGTCGGCAATAGCGGCACACTTGCCTAAGAGAACAGACAATGAGATCAAGAATCACTGGAACACACA
gznn1_1                             281 GGTGGTCGGCAATAGCGGCACACTTGCCTAAGAGAACAGACAATGAGATCAAGAATCACTGGAACACACA
Xinhai21(GOBAR_DD14076.1 NAU)       281 GGTGGTCGGCAATAGCGGCACACTTGCCTAAGAGAACAGACAATGAGATCAAGAATCACTGGAACACACA
3-79(Gbscaffold4699.2.0 HZAU)       281 GGTGGTCGGCAATAGCGGCACACTTGCCTAAGAGAACAGACAATGAGATCAAGAATCACTGGAACACACA
MD17                                281 GGTGGTCGGCAATAGCGGCACACTTGCCTAAGAGAACAGACAATGAGATCAAGAATCACTGGAACACACA
Ma85                                281 GGTGGTCGGCAATAGCGGCACACTTGCCTAAGAGAACAGACAATGAGATCAAGAATCACTGGAACACACA
```

JZ-Wild	281	GGTGGTCGGCAATAGCGGCACACTTGCCTAAGAGAACAGACAATGAGATCAAGAATCACTGGAACACACA
TaoGZ	281	GGTGGTCGGCAATAGCGGCACACTTGCCTAAGAGAACAGACAATGAGATCAAGAATCACTGGAACACACA
GZNn2_1	281	GGTGGTCGGCAATAGCGGCACACTTGCCTAAGAGAACAGACAATGAGATCAAGAATCACTGGAACACACA
TM-1(this study)	281	GGTGGTCGGCAATAGCGGCACACTTGCCTAAGAGAACAGACAATGAGATCAAGAATCACTGGAACACACA
11452GZ	281	GGTGGTCGGCAATAGCGGCACACTTGCCTAAGAGAACAGACAATGAGATCAAGAATCACTGGAACACACA
SL1-7-1	281	GGTGGTCGGCAATAGCGGCACACTTGCCTAAGAGAACAGACAATGAGATCAAGAATCACTGGAACACACA
TM-1(Gh_D12G1629 NAU)	281	GGTGGTCGGCAATAGCGGCACACTTGCCTAAGAGAACAGACAATGAGATCAAGAATCACTGGAACACACA
JZ-fl	281	GGTGGTCGGCAATAGCGGCACACTTGCCTAAGAGAACAGACAATGAGATCAAGAATCACTGGAACACACA
Xu142_fl	281	GGTGGTCGGCAATAGCGGCACACTTGCCTAAGAGAACAGACAATGAGATCAAGAATCACTGGAACACACA
081925_fl	281	GGTGGTCGGCAATAGCGGCACACTTGCCTAAGAGAACAGACAATGAGATCAAGAATCACTGGAACACACA
TM-1(CotAD_16205.1 BGI)	281	GGTGGTCGGCAATAGCGGCACGCTTGCCTAAGAGAACAGACAATGAGATCAAGAATCACTGGAACACACA
Gorai.008G179800.1	281	GGTGGTCGGCAATAGCGGCACACTTGCCTAAGAGAACAGACAATGAGATCAAGAATCACTGGAACACACA

n2	351	TCTAAAGAAAAGGCTAATCAAAATGGGTATTGATCCCATGACTCACAAGCCCTCAACCTCCCCATCACCC
gznn1_1	351	TCTAAAGAAAAGGCTAATCAAAATGGGTATTGATCCCATGACTCACAAGCCCTCAACCTCCCCATCACCC
Xinhai21(GOBAR_DD14076.1 NAU)	351	TCTAAAGAAAAGGCTAATCAAAATGGGTATTGATCCCATGACTCACAAGCCCTCAACCACCCCATCACCC
3-79(Gbscaffold4699.2.0 HZAU)	351	TCTAAAGAAAAGGCTAATCAAAATGGGTATTGATCCCATGACTCACAAGCCCTCAACCACCCCATCACCC
MD17	351	TCTAAAGAAAAGGCTAATCAAAATGGGTATTGATCCCATGACTCACAAGCCCTCAACCACCCCATCACCC
Ma85	351	TCTAAAGAAAAGGCTAATCAAAATGGGTATTGATCCCATGACTCACAAGCCCTCAACCTCCCCATCACCC
JZ-Wild	351	TCTAAAGAAAAGGCTAATCAAAATGGGTATTGATCCCATGACTCACAAGCCCTCAACCTCCCCATCACCC
TaoGZ	351	TCTAAAGAAAAGGCTAATCAAAATGGGTATTGATCCCATGACTCACAAGCCCTCAACCTCCCCATCACCC
GZNn2_1	351	TCTAAAGAAAAGGCTAATCAAAATGGGTATTGATCCCATGACTCACAAGCCCTCAACCTCCCCATCACCC
TM-1(this study)	351	TCTAAAGAAAAGGCTAATCAAAATGGGTATTGATCCCATGACTCACAAGCCCTCAACCTCCCCATCACCC
11452GZ	351	TCTAAAGAAAAGGCTAATCAAAATGGGTATTGATCCCATGACTCACAAGCCCTCAACCTCCCCATCACCC
SL1-7-1	351	TCTAAAGAAAAGGCTAATCAAAATGGGTATTGATCCCATGACTCACAAGCCCTCAACCTCCCCATCACCC
TM-1(Gh_D12G1629 NAU)	351	TCTAAAGAAAAGGCTAATCAAAATGGGTATTGATCCCATGACTCACAAGCCCTCAACCTCCCCATCACCC
JZ-fl	351	TCTAAAGAAAAGGCTAATCAAAATGGGTATTGATCCCATGACTCACAAGCCCTCAACCTCCCCATCACCC
Xu142_fl	351	TCTAAAGAAAAGGCTAATCAAAATGGGTATTGATCCCATGACTCACAAGCCCTCAACCTCCCCATCACCC
081925_fl	351	TCTAAAGAAAAGGCTAATCAAAATGGGTATTGATCCCATGACTCACAAGCCCTCAACCTCCCCATCACCC
TM-1(CotAD_16205.1 BGI)	351	TCTAAAGAAAAGGCTAATCAAAATGGGTATTGATCCCATGACTCACAAGCCCTCAACCACCCCATCACCC
Gorai.008G179800.1	351	TCTAAAGAAAAGGTTAATCAAAATGGGTATTGATCCCATGACTCACAAGCCCTCAACCTCCCCATCACCG

n2	421	AAAAATGGTTCAAATCTAAGCCATATGACCCAGGGGGAGAGTGCACGTCTCCAGGCTGAAGCCAGGTTGG
gznn1_1	421	AAAAATGGTTCAAATCTAAGCCATATGACCCAGGGGGAGAGTGCACGTCTCCAGGCTGAAGCCAGGTTGG
Xinhai21(GOBAR_DD14076.1 NAU)	421	AAAAATGGTTCAAATTTAAGCCACATGGCCCAGTGGGAGAGTGCACGTCTACAGGCTGAAGCCAGGTTGG
3-79(Gbscaffold4699.2.0 HZAU)	421	AAAAATGGTTCAAATCTAAGCCATATGACCCAGGGGGAGAGTGCACGTCTCCAGGCTGAAGCCAGGTTGG
MD17	421	AAAAATGGTTCAAATCTAAGCCATATGACCCAGGGGGAGAGTGCACGTCTCCAGGCTGAAGCCAGGTTGG
Ma85	421	AAAAATGGTTCAAATCTAAGCCATATGACCCAGTGGGAGAGTGCACGTCTCGAGGCTGAAGCCAGGTTGG
JZ-Wild	421	AAAAATGGTTCAAATCTAAGCCATATGACCCAGTGGGAGAGTGCACGTCTCGAGGCTGAAGCCAGGTTGG
TaoGZ	421	AAAAATGGTTCAAATCTAAGCCATATGACCCAGTGGGAGAGTGCACGTCTCGAGGCTGAAGCCAGGTTGG
GZNn2_1	421	AAAAATGGTTCAAATCTAAGCCATATGACCCAGTGGGAGAGTGCACGTCTCGAGGCTGAAGCCAGGTTGG
TM-1(this study)	421	AAAAATGGTTCAAATCTAAGCCATATGACCCAGTGGGAGAGTGCACGTCTCGAGGCTGAAGCCAGGTTGG
11452GZ	421	AAAAATGGTTCAAATCTAAGCCATATGACCCAGTGGGAGAGTGCACGTCTCGAGGCTGAAGCCAGGTTGG
SL1-7-1	421	AAAAATGGTTCAAATCTAAGCCATATGACCCAGTGGGAGAGTGCACGTCTCCAGGCTGAAGCCAGGTTGG
TM-1(Gh_D12G1629 NAU)	421	AAAAATGGTTCAAATCTAAGCCATATGACCCAGTGGGAGAGTGCACGTCTCCAGGCTGAAGCCAGGTTGG

JZ-fl	421	AAAAATGGTTCAAATCTAAGCCATATGACCCAGTGGGAGAGTGCACGTCTCCAGGCTGAAGCCAGGTTGG
Xu142_fl	421	AAAAATGGTTCAAATCTAAGCCATATGACCCAGTGGGAGAGTGCACGTCTCCAGGCTGAAGCCAGGTTGG
081925_fl	421	AAAAATGGTTCAAATCTAAGCCATATGACCCAGTGGGAGAGTGCACGTCTCCAGGCTGAAGCCAGGTTGG
TM-1(CotAD_16205.1 BGI)	421	AAAAATGGTTCAAATTTAAGCCACATGGCCCAGTGGGAGAGTGCACGTCTACAGGCTGAAGCCAGGTTGG
Gorai.008G179800.1	421	AAAAATGGTTCAAATCTAAGCCACATGACCCAGTGGGAGAGTGCACGTCTCCAGGCTGAAGCCAGGTTGG
n2	491	TCCGTGAGTCAAAACAGGTTGTCCCAAATCTTACCACCCGCCCCACTAGATGGAGTCAACTCACCAGAAG
gznn1_1	491	TCCGTGAGTCAAAACAGGTTGTCCCAAATCTTACCACCCGCCCCACTAGATGGAGTCAACTCACCAGAAG
Xinhai21(GOBAR_DD14076.1 NAU)	491	TCCGTGAGTCAAAACAGGTTGTCCCAAATCTTACCACCCGCCCCACTAGGAGGAGTCAACTCACCAGAAG
3-79(Gbscaffold4699.2.0 HZAU)	491	TCCGTGAGTCAAAACAGGTTGTCCCAAATCTTACCACCCGCCCCACTAGATGGAGTCAACTCACCAGAAG
MD17	491	TCCGTGAGTCAAAACAGGTTGTCCCAAATCTTACCACCCGCCCCACTAGATGGAGTCAACTCACCAGAAG
Ma85	491	TCCGTGAGTAAAAACAGGTTGTCCCAAATCTTACCACCCGCCCCACTAGGAGGAGTCAACTCACCAGAAG
JZ-Wild	491	TCCGTGAGTAAAAACAGGTTGTCCCAAATCTTACCACCCGCCCCACTAGGAGGAGTCAACTCACCAGAAG
TaoGZ	491	TCCGTGAGTAAAAACAGGTTGTCCCAAATCTTACCACCCGCCCCACTAGGAGGAGTCAACTCACCAGAAG
GZNn2_1	491	TCCGTGAGTAAAAACAGGTTGTCCCAAATCTTACCACCCGCCCCACTAGGAGGAGTCAACTCACCAGAAG
TM-1(this study)	491	TCCGTGAGTAAAAACAGGTTGTCCCAAATCTTACCACCCGCCCCACTAGGAGGAGTCAACTCACCAGAAG
11452GZ	491	TCCGTGAGTAAAAACAGGTTGTCCCAAATCTTACCACCCGCCCCACTAGGAGGAGTCAACTCACCAGAAG
SL1-7-1	491	TCCGTGAGTAAAAACAGGTTGTCCCAAATCTTACCACCCGCCCCACTAGGAGGAGTCAACTCACCAGAAG
TM-1(Gh_D12G1629 NAU)	491	TCCGTGAGTAAAAACAGGTTGTCCCAAATCTTACCACCCGCCCCACTAGGAGGAGTCAACTCACCAGAAG
JZ-fl	491	TCCGTGAGTAAAAACAGGTTGTCCCAAATCTTACCACCCGCCCCACTAGGAGGAGTCAACTCACCAGAAG
Xu142_fl	491	TCCGTGAGTAAAAACAGGTTGTCCCAAATCTTACCACCCGCCCCACTAGGAGGAGTCAACTCACCAGAAG
081925_fl	491	TCCGTGAGTAAAAACAGGTTGTCCCAAATCTTACCACCCGCCCCACTAGGAGGAGTCAACTCACCAGAAG
TM-1(CotAD_16205.1 BGI)	491	TCCGTGAGTCAAAACAGGTTGTCCCAAATCTTACCACCCGCCCCACTAGGAGGAGTCAACTCACCAGAAG
Gorai.008G179800.1	491	TCCGTGAGTCAAAACAGGTTGTCCCAAATCTTACCACCCGCCCCACTAGGAGGAGTCAACTCACCAGAAG
n2	561	CAGTCCCAGGTGCCTTGACGTACTCAAAGCCTGGCAAGGTATAGTTGCCGGCATGTTTGTTTTCTCCACC
gznn1_1	561	CAGTCCCAGGTGCCTTGACGTACTCAAAGCCTGGCAAGGTATAGTTGCCGGCATGTTTGTTTTCTCCACC
Xinhai21(GOBAR_DD14076.1 NAU)	561	CAGTCCCAGATGCCTTGACGTACTCAAAGCCTGGCAAGGTGTAGTTGCCGGGATGTTCGTTTTCTCCACC
3-79(Gbscaffold4699.2.0 HZAU)	561	CAGTCCCAGGTGCCTTGACGTACTCAAAGCCTGGCAAGGTATAGTTGCCGGCATGTTTGTTTTCTCCACC
MD17	561	CAGTCCCAGGTGCCTTGACGTACTCAAAGCCTGGCAAGGTATAGTTGCCGGCATGTTTGTTTTCTCCACC
Ma85	561	CAGTCCCAGGTGCCTTGACGTACTCAAAGCCTGGCAAGGTATAGTTGCCGGCATGTTTGTTTTCTCCACC
JZ-Wild	561	CAGTCCCAGGTGCCTTGACGTACTCAAAGCCTGGCAAGGTATAGTTGCCGGCATGTTTGTTTTCTCCACC
TaoGZ	561	CAGTCCCAGGTGCCTTGACGTACTCAAAGCCTGGCAAGGTATAGTTGCCGGCATGTTTGTTTTCTCCACC
GZNn2_1	561	CAGTCCCAGGTGCCTTGACGTACTCAAAGCCTGGCAAGGTATAGTTGCCGGCATGTTTGTTTTCTCCACC
TM-1(this study)	561	CAGTCCCAGGTGCCTTGACGTACTCAAAGCCTGGCAAGGTATAGTTGCCGGCATGTTTGTTTTCTCCACC
11452GZ	561	CAGTCCCAGGTGCCTTGACGTACTCAAAGCCTGGCAAGGTATAGTTGCCGGCATGTTTGTTTTCTCCACC
SL1-7-1	561	CAGTCCCAGGTGCCTTGACGTACTCAAAGCCTGGCAAGGTATAGTTGCCGGCATGTTTGTTTTCTCCACC
TM-1(Gh_D12G1629 NAU)	561	CAGTCCCAGGTGCCTTGACGTACTCAAAGCCTGGCAAGGTATAGTTGCCGGCATGTTTGTTTTCTCCACC
JZ-fl	561	CAGTCCCAGGTGCCTTGACGTACTCAAAGCCTGGCAAGGTATAGTTGCCGGCATGTTTGTTTTCTCCACC
Xu142_fl	561	CAGTCCCAGGTGCCTTGACGTACTCAAAGCCTGGCAAGGTATAGTTGCCGGCATGTTTGTTTTCTCCACC
081925_fl	561	CAGTCCCAGGTGCCTTGACGTACTCAAAGCCTGGCAAGGTATAGTTGCCGGCATGTTTGTTTTCTCCACC
TM-1(CotAD_16205.1 BGI)	561	CAGTCCCAGGTGCCTTGACATACTCAAAGCCTGGCAAGGTGTAGTTGCCGGGATGTTCGTTTTCTCCACC
Gorai.008G179800.1	561	CAGTCCCAGGTGCCTTGACGTACTCAAAGCCTGGCAAGGTATAGTTGCCGGCATGTTTGTTTTCTCCACC
n2	631	CAGGATCCCAGGTCCCTAACAACCTCAACTCTTCGTTTCCCTTCAGCTGGATGGGGAGAAGCTGAGGAAT

```
gznn1_1                          631 CAGGATCCCAGGTCCCTAACAACCTCAACTCTTCGTTTCCCTTCAGCTGGATGGGGAGAAGCTGAGGAAT
Xinhai21(GOBAR_DD14076.1 NAU)    631 CAGGATCCCAGGTCCCTAACAACCTCAACTCTTCGTTTCCCTTCAGCTGGATGGGGAGAAGCTGAGGAAT
3-79(Gbscaffold4699.2.0 HZAU)    631 CAGGATCCCAGGTCCCTAACAACCTCAACTCTTCGTTTCCCTTCAGCTGGATGGGGAGAAGCTGAGGAAT
MD17                             631 CAGGATCCCAGGTCCCTAACAACCTCAACTCTTCGTTTCCCTTCAGCTGGATGGGGAGAAGCTGAGGAAT
Ma85                             631 CAGGATCCCAGGTCCCTAACAACCTCAACTCTTCGTTTCCCTTCAGCTGGATGGGGAGAAGCTGAGGAAT
JZ-Wild                          631 CAGGATCCCAGGTCCCTAACAACCTCAACTCTTCGTTTCCCTTCAGCTGGATGGGGAGAAGCTGAGGAAT
TaoGZ                            631 CAGGATCCCAGGTCCCTAACAACCTCAACTCTTCGTTTCCCTTCAGCTGGATGGGGAGAAGCTGAGGAAT
GZNn2_1                          631 CAGGATCCCAGGTCCCTAACAACCTCAACTCTTCGTTTCCCTTCAGCTGGATGGGGAGAAGCTGAGGAAT
TM-1(this study)                 631 CAGGATCCCAGGTCCCTAACAACCTCAACTCTTCGTTTCCCTTCAGCTGGATGGGGAGAAGCTGAGGAAT
11452GZ                          631 CAGGATCCCAGGTCCCTAACAACCTCAACTCTTCGTTTCCCTTCAGCTGGATGGGGAGAAGCTGAGGAAT
SL1-7-1                          631 CAGGATCCCAGGTCCCTAACAACCTCAACTCTTCGTTTCCCTTCAGCTGGATGGGGAGAAGCTGAGGAAT
TM-1(Gh_D12G1629 NAU)            631 CAGGATCCCAGGTCCCTAACAACCTCAACTCTTCGTTTCCCTTCAGCTGGATGGGGAGAAGCTGAGGAAT
JZ-fl                            631 CAGGATCCCAGGTCCCTAACAACCTCAACTCTTCGTTTCCCTTCAGCTGGATGGGGAGAAGCTGAGGAAT
Xu142_fl                         631 CAGGATCCCAGGTCCCTAACAACCTCAACTCTTCGTTTCCCTTCAGCTGGATGGGGAGAAGCTGAGGAAT
081925_fl                        631 CAGGATCCCAGGTCCCTAACAACCTCAACTCTTCGTTTCCCTTCAGCTGGATGGGGAGAAGCTGAGGAAT
TM-1(CotAD_16205.1 BGI)          631 CAGGATCCCAGGTCCCTAACAACCTCAACTCTTCGTTTCCCTTCAGCTGGATGGGGAGAAGCTGAGGAAT
Gorai.008G179800.1               631 CAGGATCCCAGGTCCCTAACAACCTCAACTCTTCGTTTCCCT------GGATGGGTAGAAGCTGAGGAAT

n2                               701 GGCGGGGCCAGGGAAAGAAGGGTTCCAGCGAAGCTGATGATGCATGGTTTGAGGAGGACTCAGTCATACT
gznn1_1                          701 GGCGGGGCCAGGGAAAGAAGGGTTCCAGCGAAGCTGATGATGCATGGTTTGAGGAGGACTCAGTCATACT
Xinhai21(GOBAR_DD14076.1 NAU)    701 GGCGGGGCCAGGGAATGAAGGGTTCCAGCGATGCTGATGATGCATGGTTTGAGGAGGACTCAGTCATACT
3-79(Gbscaffold4699.2.0 HZAU)    701 GGCGGGGCCAGGGAAAGAAGGGTTCCAGCGAAGCTGATGATGCATGGTTTGAGGAGGACTCAGTCATACT
MD17                             701 GGCGGGGCCAGGGAAAGAAGGGTTCCAGCGAAGCTGATGATGCATGGTTTGAGGAGGACTCAGTCATACT
Ma85                             701 GGCGGGGCCAGGGAAAGAAGGGTTCCAGCGATGCTGATGATGCATGGTTTGAGGAGGACTCAGTCATACT
JZ-Wild                          701 GGCGGGGCCAGGGAAAGAAGGGTTCCAGCGATGCTGATGATGCATGGTTTGAGGAGGACTCAGTCATACT
TaoGZ                            701 GGCGGGGCCAGGGAAAGAAGGGTTCCAGCGATGCTGATGATGCATGGTTTGAGGAGGACTCAGTCATACT
GZNn2_1                          701 GGCGGGGCCAGGGAAAGAAGGGTTCCAGCGATGCTGATGATGCATGGTTTGAGGAGGACTCAGTCATACT
TM-1(this study)                 701 GGCGGGGCCAGGGAAAGAAGGGTTCCAGCGATGCTGATGATGCATGGTTTGAGGAGGACTCAGTCATACT
11452GZ                          701 GGCGGGGCCAGGGAAAGAAGGGTTCCAGCGATGCTGATGATGCATGGTTTGAGGAGGACTCAGTCATACT
SL1-7-1                          701 GGCGGGGCCAGGGAAAGAAGGGTTCCAGCGATGCTGATGATGCATGGTTTGAGGAGGACTCAGTCATACT
TM-1(Gh_D12G1629 NAU)            701 GGCGGGGCCAGGGAAAGAAGGGTTCCAGCGATGCTGATGATGCATGGTTTGAGGAGGACTCAGTCATACT
JZ-fl                            701 GGCGGGGCCAGGGAAAGAAGGGTTCCAGCGATGCTGATGATGCATGGTTTGAGGAGGACTCAGTCATACT
Xu142_fl                         701 GGCGGGGCCAGGGAAAGAAGGGTTCCAGCGATGCTGATGATGCATGGTTTGAGGAGGACTCAGTCATACT
081925_fl                        701 GGCGGGGCCAGGGAAAGAAGGGTTCCAGCGATGCTGATGATGCATGGTTTGAGGAGGACTCAGTCATACT
TM-1(CotAD_16205.1 BGI)          701 GGCGGGGCCAGGGAATGAAGGGTTCCAGCGATGCTGATGATGCATGGTTTGAGGAGGACTCAGTCATACT
Gorai.008G179800.1               695 GGCGGGGCCAGGGAAAGAAGGGTTCCAGCGATGCTGATGATGCATGGTTTGAGGAGGACTCAGTCATACT

n2                               771 ACACAGTCTACCTATTGCAAATATAATGGAAGGTTTGTCGGATGCTTTTATTTTGAATTCATGGATGGGT
gznn1_1                          771 ACACAGTCTACCTATTGCAAATATAATGGAAGGTTTGTCGGATGCTTTTATTTTGAATTCATGGATGGGT
Xinhai21(GOBAR_DD14076.1 NAU)    771 ACACAGTCTACCTATTGAAAATATAATGGAAGGTTTATCGGATGCTTTTATTTTGAATTCATGGATGGGT
3-79(Gbscaffold4699.2.0 HZAU)    771 ACACAGTCTACCTATTGCAAATATAATGGAAGGTTTGTCGGATGCTTTTATTTTGAATTCATGGATGGGT
MD17                             771 ACACAGTCTACCTATTGCAAATATAATGGAAGGTTTGTCGGATGCTTTTATTTTGAATTCATGGATGGGT
Ma85                             771 ACACAGTCTACCTATTGCAAATATAATGGAAGGTTTGTCGGATGCTTTTATTTTGAATTCATGGATGGGT
JZ-Wild                          771 ACACAGTCTACCTATTGCAAATATAATGGAAGGTTTGTCGGATGCTTTTATTTTGAATTCATGGATGGGT
TaoGZ                            771 ACACAGTCTACCTATTGCAAATATAATGGAAGGTTTGTCGGATGCTTTTATTTTGAATTCATGGATGGGT
```

```
GZNn2_1                          771 ACACAGTCTACCTATTGCAAATATAATGGAAGGTTTGTCGGATGCTTTTATTTTGAATTCATGGATGGGT
TM-1(this study)                 771 ACACAGTCTACCTATTGCAAATATAATGGAAGGTTTGTCGGATGCTTTTATTTTGAATTCATGGATGGGT
11452GZ                          771 ACACAGTCTACCTATTGCAAATATAATGGAAGGTTTGTCGGATGCTTTTATTTTGAATTCATGGATGGGT
SL1-7-1                          771 ACACAGTCTACCTATTGCAAATATAATGGAAGGTTTGTCGGATGCTTTTATTTTGAATTCATGGATGGGT
TM-1(Gh_D12G1629 NAU)            771 ACACAGTCTACCTATTGCAAATATAATGGAAGGTTTGTCGGATGCTTTTATTTTGAATTCATGGATGGGT
JZ-fl                            771 ACACAGTCTACCTATTGCAAATATAATGGAAGGTTTGTCGGATGCTTTTATTTTGAATTCATGGATGGGT
Xu142_fl                         771 ACACAGTCTACCTATTGCAAATATAATGGAAGGTTTGTCGGATGCTTTTATTTTGAATTCATGGATGGGT
081925_fl                        771 ACACAGTCTACCTATTGCAAATATAATGGAAGGTTTGTCGGATGCTTTTATTTTGAATTCATGGATGGGT
TM-1(CotAD_16205.1 BGI)          771 ACACAGTCTACCTATTGAAAATATAATGGAAGGTTTGTCGGATGCTTTTATTTTGAATTCATGGATGGGT
Gorai.008G179800.1               765 ACACAGTCTACCTATTGCAAATATAATGGAAGGTTTGTCGGATGCTTTTATTTTGAATTCATGGATGGGT

n2                               841 GTCGACAAATCAACAGATGAAAACACTGTAATGGAGAATGGT-AATTGCTGGGATAGCGTACTCAACTTC
gznn1_1                          841 GTCGACAAATCAACAGATGAAAACACTGTAATGGAGAATGGT-AATTGCTGGGATAGCGTACTCAACTTC
Xinhai21(GOBAR_DD14076.1 NAU)    841 GTCGACAAATCAACAGATGAAAACACTGTAAAGGAGAATGGT-AATTGCTGGGATAGCGTACTCAACTTG
3-79(Gbscaffold4699.2.0 HZAU)    841 GTCGACAAATCAACAGATGAAAACACTGTAATGGAGAATGGT-AATTGCTGGGATAGCGTACTCAACTTC
MD17                             841 GTCGACAAATCAACAGATGAAAACACTGTAATGGAGAATGGT-AATTGCTGGGATAGCGTACTCAACTTC
Ma85                             841 GTCGACAAATCAACAGATGAAAACACTGTAATGGAGAATGGT-AATTGCTGGGATAGCGTACTCAACTTC
JZ-Wild                          841 GTCGACAAATCAACAGATGAAAACACTGTAATGGAGAATGGT-AATTGCTGGGATAGCGTACTCAACTTC
TaoGZ                            841 GTCGACAAATCAACAGATGAAAACACTGTAATGGAGAATGGT-AATTGCTGGGATAGCGTACTCAACTTC
GZNn2_1                          841 GTCGACAAATCAACAGATGAAAACACTGTAATGGAGAATGGT-AATTGCTGGGATAGCGTACTCAACTTC
TM-1(this study)                 841 GTCGACAAATCAACAGATGAAAACACTGTAATGGAGAATGGT-AATTGCTGGGATAGCGTACTCAACTTC
11452GZ                          841 GTCGACAAATCAACAGATGAAAACACTGTAATGGAGAATGGT-AATTGCTGGGATAGCGTACTCAACTTC
SL1-7-1                          841 GTCGACAAATCAACAGATGAAAACACTGTAATGGAGAATGGT-AATTGCTGGGATAGCGTACTCAACTTC
TM-1(Gh_D12G1629 NAU)            841 GTCGACAAATCAACAGATGAAAACACTGTAATGGAGAATGGT-AATTGCTGGGATAGCGTACTCAACTTC
JZ-fl                            841 GTCGACAAATCAACAGATGAAAACACTGTAATGGAGAATGGT-AATTGCTGGGATAGCGTACTCAACTTC
Xu142_fl                         841 GTCGACAAATCAACAGATGAAAACACTGTAATGGAGAATGGT-AATTGCTGGGATAGCGTACTCAACTTC
081925_fl                        841 GTCGACAAATCAACAGATGAAAACACTGTAATGGAGAATGGT-AATTGCTGGGATAGCGTACTCAACTTC
TM-1(CotAD_16205.1 BGI)          841 GTCGACAAATCAACAGATGAAAACATTGTAAAGGAGAATGGTCAATTGCTGGGATAG-------------
Gorai.008G179800.1               835 GTCGACAAATCAACAGATGAAAACACTGTAATGGAGAATGGT-AATTGCTGGGATAGCGTACTCAACTTC

n2                               910 TTGAATTCTTCACCATGTGGTTCGCCTGTGTTGGGATAA
gznn1_1                          910 TTGAATTCTTCACCATGTGGTTCGCCTGTGTTGGGATAA
Xinhai21(GOBAR_DD14076.1 NAU)    910 TTGAGTTCTACACCATGTGGTTCGCCTGTGTTGGGATAA
3-79(Gbscaffold4699.2.0 HZAU)    910 TTGAATTCTTCACCATGTGGTTCGCCTGTGTTGGGATAA
MD17                             910 TTGAATTCTTCACCATGTGGTTCGCCTGTGTTGGGATAA
Ma85                             910 TTGAATTCTTCACCATGTTGTTCGCCTGTGTTGGGATAA
JZ-Wild                          910 TTGAATTCTTCACCATGTTGTTCGCCTGTGTTGGGATAA
TaoGZ                            910 TTGAATTCTTCACCATGTTGTTCGCCTGTGTTGGGATAA
GZNn2_1                          910 TTGAATTCTTCACCATGTTGTTCGCCTGTGTTGGGATAA
TM-1(this study)                 910 TTGAATTCTTCACCATGTTGTTCGCCTGTGTTGGGATAA
11452GZ                          910 TTGAATTCTTCACCATGTTGTTCGCCTGTGTTGGGATAA
SL1-7-1                          910 TTGAATTCTTCACCATGTTGTTCGCCTGTGTTGGGATAA
TM-1(Gh_D12G1629 NAU)            910 TTGAATTCTTCACCATGTTGTTCGCCTGTGTTGGGATAA
JZ-fl                            910 TTGAATTCTTCACCATGTTGTTCGCCTGTGTTGGGATAA
Xu142_fl                         910 TTGAATTCTTCACCATGTTGTTCGCCTGTGTTGGGATAA
```

**081925_fl**	910	TTGAATTCTTCACCATGTTGTTCGCCTGTGTTGGGATAA
**TM-1(CotAD_16205.1 BGI)**	897	---------------------------------------
**Gorai.008G179800.1**	904	TTGAATTCTTCACCATGTGGTTCGCCTGTGTTGGGATAA
